# Introducing a Novel Course-Based Undergraduate Research Experience Using Duckweed as a Model System

**DOI:** 10.1093/iob/obaf049

**Published:** 2025-12-19

**Authors:** J Daniels, M McCallum, N Neal, E Nkwocha, S R Machado, H Carter, E Lam, C Peng, A O’Brien, N Wei, M Frederickson, J Tan

**Affiliations:** Department of Biological Sciences, Louisiana State University, Baton Rouge, LA 70803, USA; Department of Biological Sciences, Louisiana State University, Baton Rouge, LA 70803, USA; Department of Botany, University of Wisconsin-Madison, Madison, WI 53706, USA; Department of Biological Sciences, Louisiana State University, Baton Rouge, LA 70803, USA; Department of Biological Sciences, Louisiana State University, Baton Rouge, LA 70803, USA; Department of Biological Sciences, Louisiana State University, Baton Rouge, LA 70803, USA; Department of Plant Biology, Rutgers University, New Brunswick, NJ 08901, USA; State Environmental Protection Key Laboratory of Environmental Risk Assessment and Control on Chemical Process, School of Resource and Environmental Engineering, East China University of Science and Technology, Shanghai 200237, China; Department of Biological Sciences, University of New Hampshire, Durham, NH 03824, USA; State Key Laboratory of Plant Diversity and Specialty Crops, Wuhan Botanical Garden, Chinese Academy of Sciences, Wuhan, Hubei 430074, China; State Key Laboratory of Lake and Watershed Science for Water Security, Wuhan Botanical Garden, Chinese Academy of Sciences, Wuhan 430074, China; Hubei Key Laboratory of Wetland Evolution & Ecological Restoration, Wuhan Botanical Garden, Chinese Academy of Sciences, Wuhan 430074, China; University of Chinese Academy of Sciences, Beijing 100049, China; Department of Ecology and Evolutionary Biology, University of Toronto, Toronto, ON M5S 3B2, Canada; Department of Biological Sciences, Louisiana State University, Baton Rouge, LA 70803, USA

## Abstract

Course-based undergraduate research experiences (CUREs) provide a scalable model for engaging students in authentic scientific inquiry, bridging core biological concepts with real-world environmental applications. We introduce a new CURE lab tailored for introductory biology students at the undergraduate level, utilizing duckweed as a model organism to investigate ecological interactions and environmental management. Our paper presents a curriculum that engages students in hands-on research with a focus on duckweed’s role in ecosystem dynamics, pollutant remediation, and its potential as a bioresource, along with scientific results from student projects that serve as tangible examples of the curriculum’s outcomes. Through experimentation, students explore how duckweed can be applied to address real-world environmental challenges, utilizing advanced laboratory techniques and data analysis tools. Successfully implemented with 192 students across three semesters at our institutions, this CURE lab has produced reliable duckweed growth data with high reproducibility. This curriculum addresses the gap between traditional laboratory exercises and authentic research experiences through introducing opportunities to conduct reproducible experiments, analyze real data, and communicate scientific findings in meaningful contexts.

## Introduction

The rising demand for science, technology, engineering, and mathematics (STEM) graduates highlights a challenge in higher education: providing an increasing number of students with meaningful, hands-on research experiences. Traditional research opportunities, such as lab internships or assistantships, are limited in availability and require instructors to provide significant time and resources per student. As a result, these opportunities are accessible to only a small fraction of students. This lack of accessibility to research experiences creates a gap in students’ ability to connect theoretical knowledge with practical applications, limiting their understanding of complex ecological and biological processes (Gilissen et al. 2021). Course-based undergraduate research experiences (CUREs) offer a promising bridge to this gap by integrating authentic research opportunities directly into the undergraduate curriculum (Auchincloss et al. 2014; Bangera and Brownell 2014; Estrada et al. 2018). CUREs have been known to contribute to developing a more scientifically literate population, prepared for careers in science and technology by making hands-on research experiences accessible to a broader range of students, and fostering critical thinking, data analysis skills, and scientific communication (Graham et al. 2013; Brownell and Kloser 2015; Corwin et al. 2015).

In this paper, we present both the scientific findings and the curriculum design of a CURE developed for introductory biology students at our institution, using two species of duckweed *(Lemna minor* and *Spirodela polyrhiza*) as a model system to explore ecological interactions. This curriculum is intended to engage students in research on the role of duckweed in environmental management, allowing them to explore concepts such as habitat fragmentation, microbial interactions, and the effects of temperature on plant growth. Through this CURE, students gain practical experience in experimental procedures, data collection, analysis, and hone skills in scientific writing and communication. In developing and implementing this CURE, our goal was not only to provide accessible research experiences for our own students, but also to offer a low-cost, adaptable curriculum that other instructors, particularly those with limited financial or institutional support, could use as a starting point for integrating authentic research into introductory biology courses.

### Significance of duckweed

Duckweeds are an ideal model organism for a semester-long CURE due to their unique combination of traits. They are among the smallest flowering plants, with rapid growth rates, simple structure, and broad environmental tolerance with the ability to form turions, specialized overwintering structures that sink to the bottom of water bodies to survive colder months (Acosta et al. 2021; Tan et al. 2024). These characteristics make duckweeds suitable for controlled experiments with many replicates, allowing students to study ecological dynamics in a manageable classroom setting.

Duckweeds have also gained attention for their potential ecological applications. They are known for their ability to bioremediate water, efficiently removing excess nutrients such as nitrogen and phosphorus, and helping to combat water pollution issues such as eutrophication (Cheng and Stomp 2009; O’Brien et al. 2020). Further, their high starch content positions them as a potential biofuel source, while duckweed’s ability to thrive in nutrient-rich waters and produce high protein plant biomass makes them a candidate for sustainable animal feed (Appenroth et al. 2017). The resilience of duckweeds and their numerous commercial applications allow students to explore a wide range of research questions related to environmental sustainability, making the experience relevant and impactful.

### Overview of the CURE curriculum

Our Duckweed CURE serves as an introductory biology laboratory course for first-year undergraduate biology majors in their first or second semester of college. The lab is a 1-credit hour course. It is offered to students enrolled in both the regular and honors sections. Each semester of the Duckweed CURE centers on a distinct ecological question predetermined by the instructor to ensure feasibility for an introductory lab while addressing real-world environmental challenges and developing key skills in experimental design and microbial techniques. These questions guide lab protocols and data collection, exposing students to different aspects of plant ecology and evolving each semester based on prior experimental successes and student outcomes (Compant et al. 2010; Cheng et al. 2019; Sharma et al. 2022; Laine and Leino 2025). As such, over the course of three semesters, students examine how habitat size influences plant–microbe interactions (Spring 2023), how temperature affects *L. minor* growth and microbes (Fall 2023), and how temperature influences turion germination and *S. polyrhiza* growth (Spring 2024). The research questions that drive the course content are designed to cultivate both conceptual understanding and practical research skills. Each semester, students engage in hands-on experimental lab work that includes sterile handling, microbial plating, and data analysis with statistical software while developing science literacy skills through scaffolded, iterative writing assignments and communication pieces, such as lab reports and scientific posters. Student learning is evaluated through a variety of assessment types, including low-stakes weekly assignments and high-stakes end-of-term assessments. In-class quizzes and exit assignments are designed to assess student understanding of each individual lab, while more lengthy take-home assignments are designed to build literacy and writing skills. Scaffolding of both in-class and take-home assignments, coupled with instructor feedback and iterative submissions, allows students to build upon their current skills and work toward high stakes assignments such as a full lab report and scientific poster.

By the end of the course, students are expected to achieve six goals: (1) develop scientific reading and writing skills, (2) cultivate critical thinking and evidence evaluation, (3) connect science to broader contexts and real-world applications, (4) strengthen science communication skills, (5) analyze biological data with statistical software and draw evidence-based conclusions; and (6) demonstrate a working knowledge of key ecological concepts, including plant–microbe interactions, habitat fragmentation, climate change, and environmental influences on plant growth.

## Materials and methods

### Research methods and instructor preparation for course

All iterations of the Duckweed CURE are conducted in a biosafety level 1 (BSL-1) laboratory, using nonpathogenic environmental microbes. The lab contains four benches with two stations each, and students worked in pairs or small groups. Each section enrolls approximately 24 students, managed by a teaching assistant responsible for two sections per semester. Students are required to wear personal protective equipment, such as gloves, lab aprons, and safety glasses, and provided with all materials needed for experiments. Across semesters, the course uses axenic duckweed cultures and bacterial monocultures prepared in advance to ensure experimental outcomes reflected only the intended species interactions. Axenic cultures are established by sterilizing duckweed fronds in 1–2% sodium hypochlorite for 15–30 s, followed by multiple sterile water rinses. For experiments involving turions, *S. polyrhiza* propagules are obtained from the Rutgers Duckweed Stock Cooperative. All cultures are maintained in one-tenth strength Hoagland’s medium (prepared as 100 mL of medium stock per liter of deionized water) under an 16 h light/8 h dark photoperiod at controlled temperatures.

In Spring 2023, we examined how habitat size influences duckweed–microbe interactions by simulating different habitat sizes using test tubes of varying diameters (10, 13, and 16 mm). Three microbial communities were introduced to their corresponding duckweed strains (see details in BiologicalMaterialsOriginMap file in the Supplemental Materials). Each student group worked with one strain and its associated community across all habitat sizes, along with two controls (duckweed only and microbes only). Each experimental block contained nine tubes per treatment with three replicates, totaling 27 samples per group.

In Fall 2023, students examined how temperature affects *L. minor* growth and microbial abundance under three treatments: 20°C (average annual temperature), 30°C (average summer), and 40°C (extreme heat). Each test tube contained 6 mL of Hoagland’s medium and was maintained under the same photoperiod as previous semesters. The 5-week experiment included 27 total samples (*n* = 9 per treatment). Weekly measurements of frond number and optical density (OD₆₀₀) were used to measure plant growth and microbial abundance.

In Spring 2024, students tested how temperature affects turion germination, growth, and survival in *S. polyrhiza*. Sterile turions were placed individually in 6-well plates containing 10 mL of Hoagland’s medium and incubated at 20, 30, or 40°C in controlled growth chambers. Each treatment included six replicate wells (*n* = 6 per temperature), totaling 18 samples per student group. Germination rate and frond growth were recorded weekly for four weeks, with percent surface coverage quantified from ImageJ image analyses. Axenic conditions were confirmed by measuring optical density at the start and end of the experiment.

Statistical analyses, including analysis of variance (ANOVA) and Tukey’s test, are chosen for their accessibility and educational value in an introductory course context, to allow for learning of variance partitioning, hypothesis testing, and interpretation of *P*-values in a manageable format. Students are introduced to the assumptions and limitations of these tests and are made aware that more advanced or non-parametric methods may be more appropriate for certain data types. The use of simplified analyses intends to support core learning goals, including experimental design, data visualization, and statistical reasoning (Bjornsdottir and Garfield 2012; Lerner and Gelman 2024).

### Course implementation

We implement this course by providing a structured, research-based learning experience that progressively builds students’ experimental, analytical, and science literacy skills through scaffolded assignments and research protocols. Students enrolled in the CURE labs are typically in their second semester of introductory biology and are expected to have a foundational understanding of basic biological concepts, including the scientific method, scientific writing, data collection, basic data analysis, and basic laboratory skills, such as general lab safety, pipette using, and microbial plating.

Prior to the start of each semester, we develop and refine all protocols in advance to ensure feasibility, clarity, and alignment with course learning objectives. Each procedure is tested by the instructional team and iteratively revised based on student feedback and observed challenges at the end of each semester. Instructors and teaching assistants hold weekly preparatory meetings to coordinate class activities, discuss potential troubleshooting steps, and align instructional goals. Each semester is structured to progressively build research skills, with early weeks emphasizing sterile techniques and later weeks focusing on data analysis and scientific communication.

We include a summary table (Table 1) outlining the weekly topics, lab activities, and major assignments. The duration of this course is approximately 13 weeks with the research project spanning the first 9 or 10 weeks and presentations of students' findings along with final exams during the final 3 weeks. The semester length does not include two breaks per semester: Spring Break and Mid-Semester Break in the spring semester, and Fall Break and Thanksgiving in the fall semester, which are also not reflected in Table 1. Each week includes 2 h and 50 min of classroom instruction and 1–3 h of independent study. Students are given all relevant materials prior to class time via the online course management system. Classroom sessions begin with a quiz, followed by a 30-min lecture, then lab exercises and an in-class assignment (ICA). Quizzes are designed to assess students’ understanding of safety protocols, previous content, techniques, and procedures before hands-on activities, ensuring that students understand what they have done prior to moving on to new material or procedures. The lectures review the previous week’s lab and assignments, and brief students on the current week’s learning objectives, theory, lab activities, and assignments. During the last weeks of class, each student completes an individual full lab report. Students then work in groups to develop a collaborative scientific poster, communicating their research project and findings to the class. A detailed list of the weekly materials and resources offered to the students can be found in the “Supplementary Materials.”

**Table 1:** Course schedule, not including break weeks

TIMELINE	TOPIC	LAB ACTIVITY	AUXILIARY MATERIAL	ASSESSMENT
Week 1	1.1 Introduction to the course 1.2 Syllabus	-Practice with pipettes	(1) Syllabus (2) Academic Integrity Agreement	**Quiz 1**
Week 2	2.1 Presentation of CURE project 2.2 Microscopy techniques 2.3 Introduction of *Lemna minor*	Practice of microscopy techniques: compound and stereoscopes Bleaching of Duckweed Draw and label duckweed plant	(1) Methods Summary Instructions (2) Microscope Instructions	**Quiz 2** **ICA**: Duckweed drawing **THA**: Methods Summary
Week 3	3.1 Introduction to sterile techniques 3.2 Introduction to aquatic microbial communities 3.3 Scientific literature search	- Plate aquatic microbial community	(1) Methods Summary Instructions (2) Protocol: Microbial Plating	**Quiz 3** **ICA**: Methods Summary **THA**: Finding and Reading a Paper
Week 4 Week 4 (Turion Version)	4. 1 How to setup an experiment 4.2 How to find, evaluate, and choose scientific papers 4.1a How to setup an experiment 4.2a How to find, evaluate, and choose scientific papers 4.3a How to use ImageJ to collect data	- Purify/isolate bacteria cultures by streaking - Selection of relevant papers - Annotate and collect data for 5 practice duckweed photos - Selection of relevant papers	(1) Methods Summary Instructions (2) Protocol: Microbial Streaking (1) Methods Summary Instructions Protocol: Photo Annotation	**Quiz 4** **ICA**: Methods Summary **THA**: Key Papers and Annotated Bibliography **Quiz 4** **ICA**: Methods Summary **THA**: Key Papers and Annotated Bibliography
Week 5	5.1 Introduction to scientific writing 5.2 Setting up an experiment 5.3 Collecting data 5.4 Sterile techniques	- Data collection (**Day 0**) - Discussion of key papers - Experiment Setup and Start Date	(1) Experiment Overview PDF (2) Protocol: Data Collection Day 0	**Quiz 5** **ICA**: Outlining of Introduction & Annotated Bibliography **FWA1**: Intro, Methods, References
Week 6	6.1 Science communication 6.2 Importance of feedback and peer-review 6.3 Collecting data 6.4 Sterile techniques	- Data collection (**Day 7**) - Peer-review anonymous FWAs	(1) Protocol: Data Collection Day 7 (2) Peer Review Instructions (3) Peer Review Comments (4) FWA Guide	**Quiz 6** **ICA**: Peer Reviews **FWA1**: Peer Reviews for FWA1
Week 7	7.1 Understanding scientific writing: methods 7.2 Collecting data 7.3 Sterile techniques	- Data collection (**Day 14**) - Adding data to spreadsheets - Outlining and description of methodology	(1) Protocol: Data Collection Day 14	**Quiz 7** **ICA**: Methodology Dissection and Development **THA**: Complete 2^nd^ draft of FWA1
Week 8	8.1 Understanding scientific writing: Results 8.2 Collecting data 8.3 Sterile techniques 8.4 Creating figures and captions	- Data collection (**Day 21**) - Data analysis using statistical software	(1) Protocol: Data Collection Day 21	**Quiz 8** **ICA**: Data Analysis and Trends—started in class and finished at home
Week 9	9.1 Understanding scientific writing: Discussion 9.2 Collecting data 9.3 Sterile techniques	- Data collection (**Day 28)** - Data analysis update - Reading and dissecting a relevant paper’s discussion section	(2) Protocol: Final Data Collection Day 28 (3) Protocol: Experiment Breakdown	**Quiz 9** **ICA:** Dissecting Results and Discussion **THA**: Develop Results and Discussion Paragraphs
Week 10	10.1 Zooplankton diversity of the LSU Lakes 10.2 Origin of study system 10.3 Fieldwork and sample collecting	- LSU lakes zooplankton microscopy	(1) Protocol: Lake Study (2) FWA2 (3) Microorganism Identification Guide	**Quiz 10** **ICA**: LSU lake study report and presentations **FWA2**: Full report
Week 11	11.1 Scientific communication: scientific posters 11.2 Reading scientific posters	- Poster Partner Contract - Poster workshop I	(1) Reading a Scientific Poster (2) Scientific Poster Guide	**Quiz 11** **ICA**: Hallway Poster Critiques
Week 12	11.1 Scientific communication: Elevator pitch 11.2 Creating a scientific poster	- Poster workshop II - Understanding Elevator Pitches	(1) Scientific Poster Guide (2) How to Write an Elevator Pitch	**Quiz 12** **ICA**: Poster Elevator Pitch **THA**: Poster presentation file submission
Week 13	15.1 Scientific communication: Communicating experiment to an audience	- Posters presentation and evaluations by group - Poster feedback for presenters - Final Exam Review	(1) Scientific Poster Guide	**ICA**: Poster Evaluations **THA**: Study Guide
Week 14	15.2 Final assessment of course 15.3 Scientific communication: Communicating experiment to an audience	Final Exam CURE Poster Session	(1) Scientific Poster Guide	CURE Poster Session—LSU Union Attendance Required

*Note:* ICA, in-class assignment; THA, take-home assignment; and FWA, formal writing assignment.

## Results

### Instructor reflections on student learning

In the Methods section above, we outlined how the course activities were designed to help students develop core scientific skills. In this section, we focus on how we track their progress and what we observe in relation to those goals. We use both formative and summative assessment tools to monitor learning progress (goals 1–5). To check for student understanding of laboratory techniques and concepts, we use weekly coursework, such as quizzes and ICAs, and students generally score well on these formative assessments. This provides us with information how students understand what has been done in lab and how it relates to the broader research project. Additionally, we use weekly iterative assignments that include reading or writing prompts to allow the graduate instructor to assess science literacy development among students. Early assignments, such as learning to read and evaluate scientific literature, provide students with the skills needed to organize and construct material for the introduction of their formal writing assignment. Certain summative assignments, such as “ICA: Introduction Outline” and “THA: Developing Results and Discussion,” provide the graduate instructor with intermittent checks of higher order understanding and critical thinking.

We would like to provide reflections of instructor observations, along with course artifacts generated as part of normal instructional activity, as examples of what our introductory biology students produced at the end of term, acknowledging that no formal data collection were conducted for research purposes in this course. From the graduate instructor’s perspective, whose dissertation research mirrors the course, student performance on final lab reports demonstrated gains in scientific reasoning, data interpretation, and clarity of written communication. Specifically, using the beginning of the semester as a baseline, students showed increasing abilities to incorporate scientific literature to support their arguments, suggesting growth in their ability to synthesize information from primary literature. By the end of the course, most students are able to form concise and critical explanations of their findings and relate it to broader contexts outside of the classroom. Similarly, most posters demonstrated students’ abilities to integrate data visualizations and appeared to foster a deeper understanding for scientific communication.

### Experimental findings

**Fig. 1:**
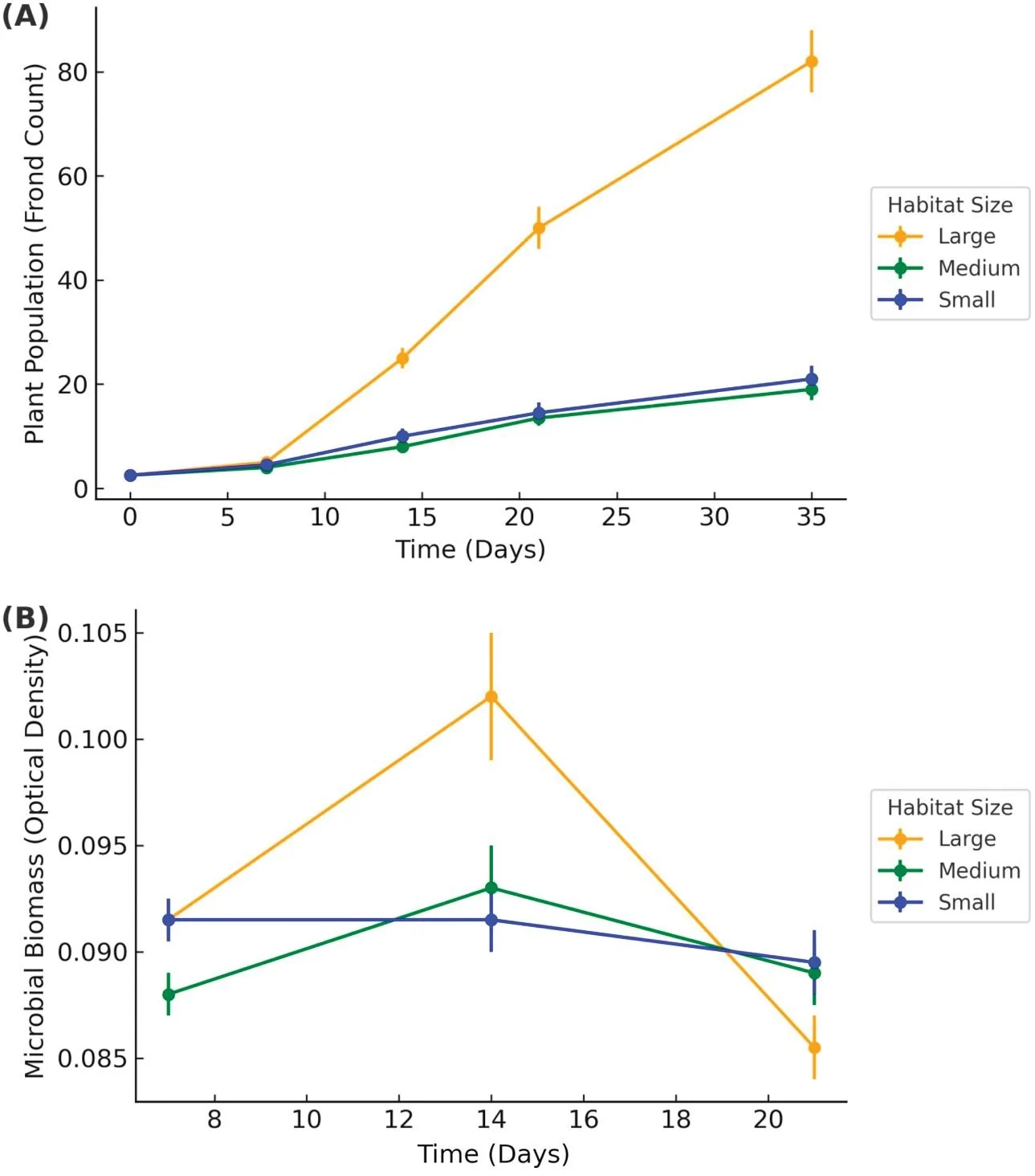
Artifact generated by student data from Spring 2023 showing **(A)** mean duckweed frond counts (*N* = 288) and **(B)** mean microbial optical density (OD600; *N* = 432) over time for three habitat sizes (10, 13, 16 mm); error bars represent standard error (SE). The data illustrates greater duckweed growth in larger tubes, while microbial density remains stable.

**Fig. 2:**
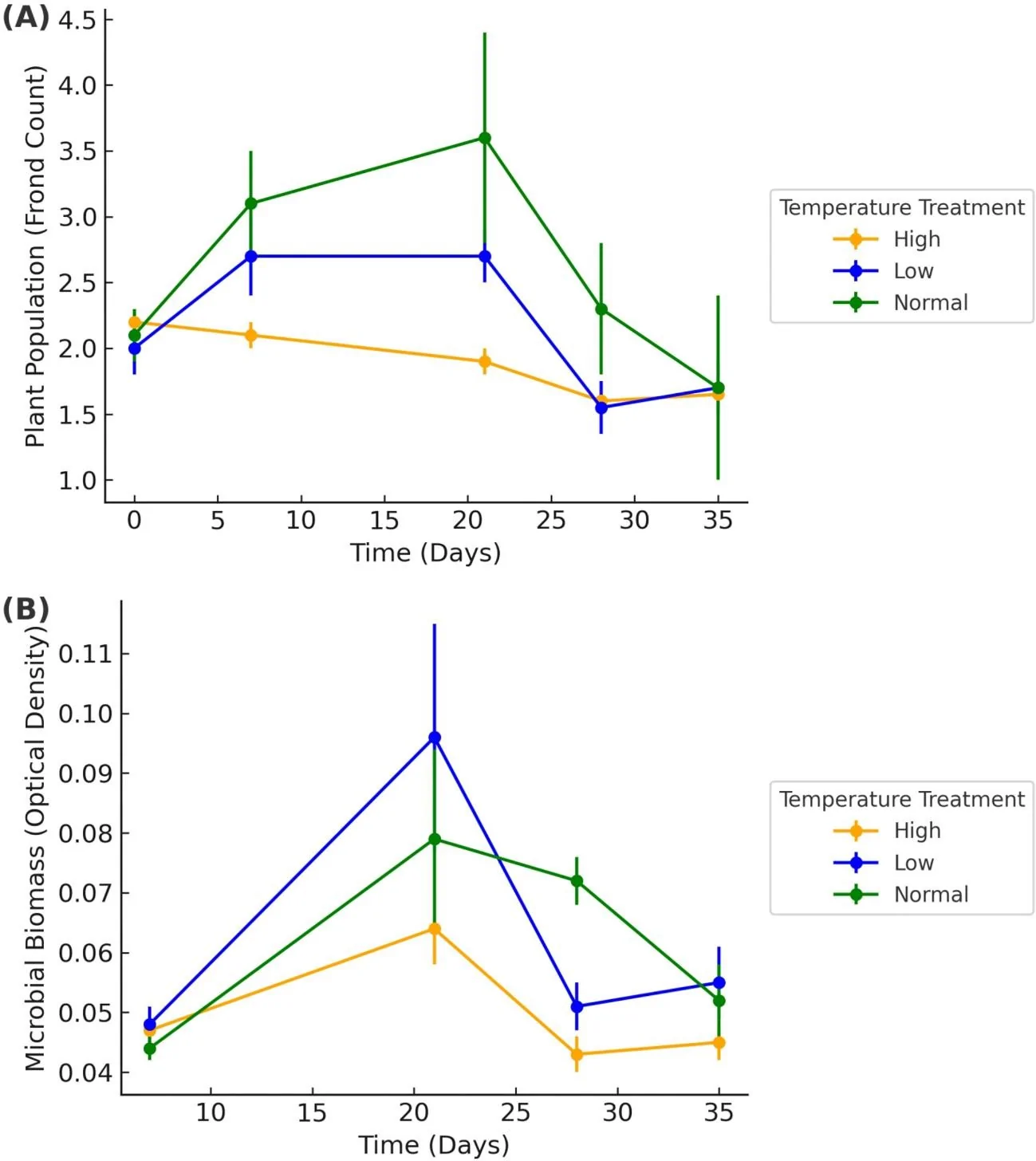
Artifact generated by student data from Fall 2023 showing **(A)** mean duckweed frond counts (*N* = 432) and **(B)** mean microbial optical density (OD600; *N* = 432) over time under three temperature treatments (20, 30, and 40°C); error bars represent SE. The data shows similar duckweed and microbial growth across temperatures.

**Fig. 3:**
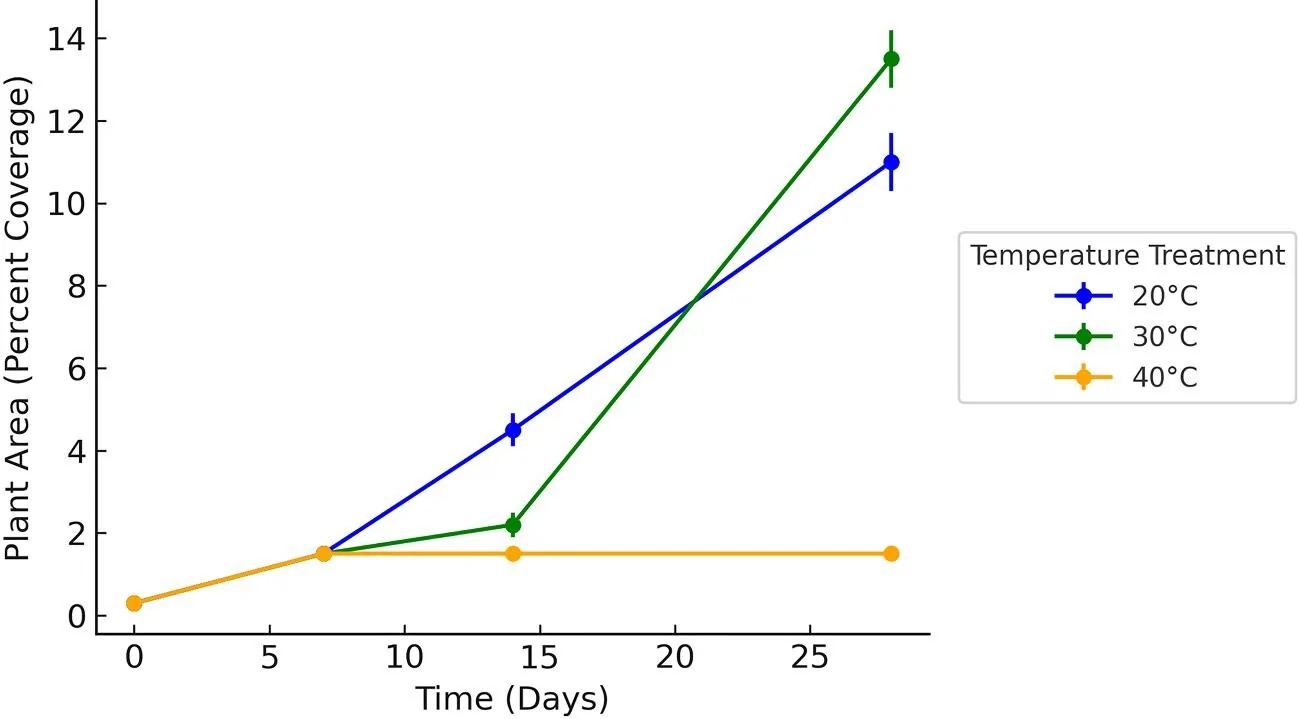
Artifact generated by student data from Spring 2024 showing mean percent coverage of *S. polyrhiza* turions over time for three temperature treatments (20, 30, and 40°C); error bars represent SE; *N* = 288. The data highlights faster germination and greater coverage at 20 and 30°C, with no growth observed at 40°C.

To demonstrate achievement of goal 6, we present the experimental results generated by students, which illustrate how environmental changes affect duckweeds and their associated microbiomes. Our experimental results revealed clear patterns demonstrating how environmental changes affect duckweeds and their associated microbiomes. In Spring 2023, habitat size significantly influenced duckweed growth, with larger habitats supporting greater frond production, while microbial density did not differ among treatments (frond count: ANOVA, followed by Tukey’s test: *F* = 23.97, *P* < 0.001; large > small (*P* < 0.001), large > medium (*P* < 0.001), small ≈ medium (*P* = 0.950); microbial density: *F* = 2.32, *P* = 0.102; Fig. 1). In Fall 2023, we examined the effects of temperature, an environmental variable known to directly influence biological processes (Parmesan 2006). Both duckweed and its microbial community displayed resilience to short-term temperature fluctuations, with no significant effects of temperature on frond count (ANOVA: *F* = 1.22, *P* = 0.322; Fig. 2A) or microbial density (*F* = 0.94, *P* = 0.419; Fig. 2B). This suggests that temperature alone may not fully expose organismal sensitivity, although high variability in the data also indicates limited power to detect subtle patterns, underscoring the need for future studies with greater replication. In Spring 2024, we investigated turion germination in *S. polyrhiza*. Turions are specialized dormant propagules that allow duckweed to survive unfavorable conditions such as cold or desiccation, playing a critical role in seasonal persistence and clonal reproduction (Kuehdorf et al. 2014). Temperature had a significant effect on turion germination rates and percent coverage of the fronds (ANOVA: *F* = 58.63, *P* < 0.001; Fig. 3). Germination and early growth were highest at 30°C, moderate at 20°C, and substantially reduced at 40°C, indicating strong thermal sensitivity during early life stages.

Together, these experiments provided students with hands-on opportunities to explore how environmental factors, such as habitat size and temperature, influence plant–microbe interactions. In doing so, students applied the skills developed earlier in the course, such as reading and interpreting scientific literature, analyzing data, and communicating their findings, thereby linking goal 6 to other learning goals. These student-generated datasets not only enhanced learning but also provided preliminary evidence that supports and extends current ecological understanding, demonstrating that CURE data can inform broader scientific questions about plant–microbiome interactions and environmental sensitivity.

## Discussion

### Challenges and successes in the execution of the curriculum design

The Duckweed CURE evolved considerably over three semesters as we refined both the scientific focus and instructional design. In Spring 2023, live microbial inoculations introduced challenges in maintaining sterile conditions since contamination and inconsistent inoculation rates were common among students handling cultures for the first time, often confounding ecological results. To improve consistency, Fall 2023 simplified the experiment to *L. minor* under controlled temperature treatments, removing the microbial component while retaining an ecological focus. By Spring 2024, we incorporated *S. polyrhiza* turions (dormant pre-sterilized propagules that minimized contamination risks) to standardize starting materials and enable observation of germination through growth within a single semester, while strengthening connections to temperature adaptation and environmental stress response.

Beyond these technical adjustments, a central challenge is managing the cognitive load for introductory students who are simultaneously learning foundational laboratory skills, navigating experimental techniques, and interpreting ecological data, often for the first time. Students frequently struggle to distinguish between natural experimental variability inherent to ecological research and procedural errors, which sometimes undermine their confidence in interpreting results and participating in scientific discourse. The optimization across semesters allows students to develop greater ownership over their data, as outcomes can be more readily attributed to experimental treatments rather than technical mishaps. These refinements also improve student engagement and experimental rigor through better pacing, scaffolding, and data recording standards.

We developed the Duckweed CURE, aiming to provide an accessible and meaningful research experience for introductory biology students at our institutions. We selected duckweed as the study system because it is easy to collect and maintain, and ecologically and economically important, making it an ideal model for engaging students in real-world research on environmental challenges (Cheng and Stomp 2009; Appenroth et al. 2017; O’Brien et al. 2020; Zhou et al. 2023). Traditional lab courses rarely allow students to engage in active inquiry, which can limit their ability to think critically about experimental design and scientific reasoning (Thompson et al. 2016; LaForge and Martin 2022; Lanclos et al. 2022). To address this, the Duckweed CURE was designed as a structured yet flexible framework in which students could explore authentic ecological questions through hands-on research, linking foundational laboratory skills to broader ecological and societal contexts. Moreover, in designing the curriculum, we seek to address broader gaps in STEM education, particularly the need for more inclusive research opportunities (Spell et al. 2014). CUREs are shown to support retention in STEM, especially for students from underrepresented backgrounds (Rodenbusch et al. 2016). By embedding research into a required course, we broaden participation in authentic scientific research, a practice shown to enhance engagement and retention in STEM by giving all students, regardless of prior experience, meaningful research opportunities (Hanauer et al. 2016). Finally, by focusing on topics like habitat fragmentation and climate change effects, we made the research questions both accessible and relevant, helping students see how their work could contribute to broader scientific understanding and societal needs (Vitousek et al. 1997; Parmesan 2006).

### Areas for improvement in duckweed CURE

One of the key limitations has been balancing the complexity of the experiments with the time frame of a single semester. For example, while students explore the effects of habitat size and temperature on duckweed and microbial communities, the short timeline means they can only observe short-term trends. Extending the course to include a longer experimental timeline or allowing cross-semester projects could provide students with the opportunity to collect longitudinal data, leading to a deeper understanding of temporal dynamics in ecological systems. Cross-cohort collaboration can also enhance the curriculum, with each cohort building on the results of previous groups (Pourmohammadali et al. 2020). The curriculum already aims to address gaps in STEM education by embedding inclusive research opportunities that connect student work to broader scientific and societal issues (Vitousek et al. 1997; Parmesan 2006; Spell et al. 2014; Hanauer et al. 2016; Rodenbusch et al. 2016). This continuity would provide a more holistic view of habitat fragmentation’s impact on ecosystems while fostering a sense of scientific contribution and community among students.

Another area for improvement is the integration of advanced molecular techniques. While the course introduced students to microbial culturing and plant growth metrics, it did not include methods such as traditional Sanger sequencing, quantitative polymerase chain reactions, and next-generation sequencing, which could offer more detailed insights into microbial community composition and plant physiological responses (Vallejo-Schmidt et al. 2024). Incorporating these methods into future versions of the course would not only enhance students’ understanding of microbial diversity but also prepare them for more advanced research in their later academic careers.

Additionally, while the Python-based data analysis component has been well received, some students struggle with retaining coding skills between sessions. This may be because pre-written notebooks can reduce opportunities for independent practice. Future iterations could focus more on building students’ coding skills incrementally throughout the semester, allowing them to write their own scripts for data analysis by the end of the course (Campbell et al. 2024). This change could better equip students for data-heavy research environments, an increasingly important skill in the biological sciences.

There is also potential to expand the interdisciplinary nature of the course. Collaboration with other university departments, such as Statistics, Coastal and Environmental Sciences, Renewable Natural Resources, and Environmental Engineering could provide students with the opportunity to apply their research in more practical contexts, such as monitoring water quality in local wetlands or studying the effects of pollution. These partnerships would not only enhance ecological inquiry but also expose students to diverse career pathways. Because not all participants will pursue research-focused careers, interdisciplinary collaboration can help students see how scientific inquiry connects to environmental management, policy, data analysis, and other applied fields, fostering wider appreciation of the relevance of science in multiple disciplines. In future iterations of the Duckweed CURE, we will integrate molecular approaches within the ecological framework and foster interdisciplinary collaboration, providing students with a comprehensive experience that connects modern research methods to practical applications across diverse scientific fields.

Lastly, while this paper focuses on presenting the curriculum design and implementation, we acknowledge that evaluating student satisfaction, perceptions of belonging, self-efficacy, and identity as a scientist is an important next step. This work represents an initial, practice-based reflection on student learning within the biology laboratory context. Future studies should build on these observations through IRB-approved research that systematically collects and analyzes student artifacts and perceptions to more rigorously evaluate the impact of these assignments on scientific literacy development and student retention in the sciences.

## Conclusions

This CURE curriculum offers a structured opportunity for biology undergraduates to engage in research experiences that connect theoretical concepts with practical applications. It is designed to be accessible, affordable, and ready for adoption by institutions aiming to incorporate a study of authentic research into their coursework. By providing an alternative to traditional laboratory course formats, this curriculum supports broader integration of research-based learning (Lanclos et al. 2022). While the curriculum has already made significant strides, it remains a work in progress with opportunities for refinement and growth. Our goal is to continue adapting the curriculum to better meet the needs of students, ensuring it remains an engaging way to explore ecological questions.

## Supplementary Material

obaf049_Supplemental_Files

## Data Availability

Data supporting this article is provided in the supplementary materials.
