# Supplementary material for: Introducing a Novel Course-Based Undergraduate Research Experience Using Duckweed as a Model System
Source: Integr Org Biol. 2025 Dec 19;8(1):obaf049. doi: 10.1093/iob/obaf049 (PMC12802901; doi:10.1093/iob/obaf049)
Supplement: obaf049_Supplemental_Files [file obaf049_supplemental_files.zip › 06 Supplementary Materials Content List 20251105.docx]

Appendix information content list for Supplementary Materials (See the .zip file).

**File-Naming Format:**

Each document title follows the format: NN_Week##_CATEGORY_TitleOfDocument

- **NN** – Two-digit sequential number showing the order of materials in the course packet.
- **Week##** – The instructional week to which the material belongs (if applicable). Some preparatory or reference files omit the week number because they are used throughout the course.
- **CATEGORY** – A code that identifies the type of material:
  - **PREPS** – Preparations or setup documents for instructors or students.
  - **RESOURCES** – Supplemental materials such as guides, tips, or templates provided to students.
  - **FORMS** – Contracts or administrative forms for student groups or presentations.
  - **PROTOCOL** – Step-by-step laboratory procedures or experimental methods.
  - **ICA** – In-Class Assignment used during scheduled lab sessions.
  - **THA** – Take-Home Assignment to be completed outside of class.
  - **ARTIFACT** - Student-generated materials that illustrate class activities and outcomes.
- **TitleOfDocument** – A brief descriptor of the specific activity, protocol, or resource.

| **Item** | **File Name** | **Type** | **Brief Description** |
| --- | --- | --- | --- |
| 00 | 00_PREPS_BiologicalMaterialsOriginMap.docx | .docx | Map of biological material origins. |
| 01 | 01_RESOURCES_LabSafety.pdf | .pdf | Lab safety instructions. |
| 02 | 02_PREPS_DuckweedFall23.docx | .docx | Background on duckweed for Fall 2023 course. |
| 03 | 03_PREPS_TurionsSpring24.docx | .docx | Background on Turion Experiments for Spring 2024. |
| 05 | 05_PREPS_R2A_Recipe.pdf | .pdf | R2A medium recipe. |
| 06 | 06_FORMS_LabPartnerContract.docx | .docx | Lab partner contract form. |
| 07 | 07_RESOURCES_Syllabus.docx | .docx | Syllabus used throughout course |
| 08 | 08_PREPS_WeeklyActivityDescriptions.docx | .docx | Summary of weekly lab activities. |
| 09 | 09_Week02_RESOURCES_MicroscopePartsAndSafety.docx | .docx | Student Handout labeling the parts of a microscope and explaining how to safely use one. |
| 10 | 10_Week02_PROTOCOL_CompoundMicroscopeStereoscopes.docx | .docx | Protocol for students to use the compound microscope to view duckweed. |
| 11 | 11_Week02_PROTOCOL_DuckweedBleaching.docx | .docx | Protocol for bleaching duckweed field samples. |
| 12 | 12_Week02_PROTOCOL_GrowthMeasurementsforStudents.docx | .docx | Protocol for measuring duckweed growth. |
| 13 | 13_Week03_PROTOCOL_MicrobialPlatingforStudents.docx | .docx | Protocol for microbial plating. |
| 14 | 14_Week03_PROTOCOL_MicrobialPlatingforStudents.docx | .docx | Alternate microbial plating protocol. |
| 15 | 15_Week03_THA_MethodsSummaryAssignmentGuide.docx | .docx | Take-home assignment: methods summary guide. |
| 16 | 16_Week03_ICA_MethodsSummaryAssignmentGuide.docx | .docx | In-class activity: methods summary. |
| 17 | 17_Week03_THA_FindingAndReadingAPaper.docx | .docx | Take-home assignment: locating and reading a paper. |
| 18 | 18_Week04_RESOURCES_ExperimentalDesign.docx | .docx | Resource for students on experimental design. |
| 19 | 19_Week04_RESOURCES_ExperimentalDesignOverview.pdf | .pdf | Overview of experimental design concepts. |
| 20 | 20_Week04_THA_ExperimentOverviewQuestions.docx | .docx | Take-home assignment: experiment overview questions. |
| 21 | 21_Week04_ICA_MethodsSummary AssignmentGuide.docx | .docx | In-class activity: methods summary guide. |
| 22 | 22_Week04_PROTOCOL_StreakingMicrobialColonies.docx | .docx | Protocol for streaking microbial colonies. |
| 23 | 23_Week04_RESOURCES_PhotoAnnotationExcelSheet.xlsx | .xlsx | Template for photo annotation and data collection. |
| 24 | 24_Week04_THA_KeyPapersStudentGuide.docx | .docx | Take-home assignment: guide to key papers. |
| 25 | 25_Week05_PROTOCOL_DataCollectionDay0.docx | .docx | Protocol for Day 0 data collection. |
| 26 | 26_Week05_PROTOCOL_InoculationAndDataCollectionDay0.docx | .docx | Protocol for inoculation and Day 0 data. |
| 27 | 27_Week05_ICA_IntroductionOutline.docx | .docx | In-class activity: introduction outline. |
| 28 | 28_Week05_RESOURCES_FWAWritingGuide.docx | .docx | Formal writing assignment guide. |
| 29 | 29_Week05_RESOURCES_DataCollectionTemplate.xlsx | .xlsx | Data collection template. |
| 30 | 30_Week05_RESOURES_TurionsDataSheet.xlsx | .xlsx | Turion data sheet template. |
| 31 | 31_Week05_ICA_AnnotatedBibliography.docx | .docx | In-class activity: annotated bibliography. |
| 32 | 32_Week06_PROTOCOL_MicrobialAdditionAndDataCollectionDay7.docx | .docx | Protocol for microbial addition and Day 7 data. |
| 33 | 33_Week06_PROTOCOL_DataCollectionDay7.docx | .docx | Protocol for Day 7 data collection. |
| 34 | 34_Week06_ICA_MethodologyDissectionDevelopment.docx | .docx | In-class activity: methodology dissection. |
| 35 | 35_Week06_ICA_PeerReviewGuidelinesAndPeerMark.docx | .docx | Peer-review guidelines and PeerMark instructions. |
| 36 | 36_Week07_PROTOCOL_DataCollectionDay14.docx | .docx | Protocol for Day 14 data collection. |
| 37 | 37_Week08_RESOURCES_ExcelStatstisticsTutorial.docx | .docx | Tutorial: statistical analysis in Excel. |
| 38 | 38_Week08_RESOURCES_JMPProtocolDataAnalysis.docx | .docx | Tutorial: data analysis in JMP. |
| 39 | 39_Week08_RESOURCES_TurionDataAnalysis.ipynb | .ipynb | Google Colab notebook for Turion data analysis. |
| 40 | 40_Week08_ICA_FiguresAndTables.docx | .docx | In-class activity: figures and tables. |
| 41 | 41_Week08_ICA_DataAnalysisReport.docx | .docx | In-class activity: data analysis report. |
| 42 | 42_Week09_PROTOCOL_DataCollectionDay28.docx | .docx | Protocol for Day 28 data collection. |
| 43 | 43_Week09_ICA_DissectingResultsAndDiscussion.docx | .docx | In-class activity: dissecting results and discussion. |
| 44 | 44_Week09_THA_DevelopingResultsAndDiscussion.docx | .docx | Take-home assignment: develop results & discussion. |
| 45 | 45_Week10_PROTOCOL_LakeStudy.docx | .docx | Protocol for lake-study fieldwork. |
| 46 | 46_Week10_ICA_PrintoutForLakeSamplingObservations.docx | .docx | In-class activity: lake-sampling observations. |
| 47 | 47_Week10_ICA_LakeStudyAlternativeAssignment.docx | .docx | Alternative assignment for lake study. |
| 48 | 48_Week11_FORMS_PosterPartnerContract.docx | .docx | Poster-presentation partner contract. |
| 49 | 49_Week11_RESOURCES_PosterTips.docx | .docx | Tips for preparing a scientific poster. |
| 50 | 50_Week11_RESOURCES_PosterTemplatesStandardSize.pptx | .pptx | Standard poster templates (PowerPoint). |
| 51 | 51_Week11_ICA_HallwayPosterCritiques.docx | .docx | In-class activity: hallway poster critiques. |
| 52 | 52_Week13_ICA_PeerEvaluationsIn-ClassPosterPresentations.docx | .docx | Peer-evaluation form for poster presentations. |
| 53 | 53_Week13_RUBRIC_In-ClassPosterPresentations.docx | .docx | Rubric for in-class poster presentations. |
| 54 | 54_ARTIFACT_FWA2Spring23.pdf | .pdf | Example student paper created from Spring 2023 Formal Writing Assignments |
| 55 | 55_ARTIFACT_FWA2Fall23.pdf | .pdf | Example student paper created from Fall 2023 Formal Writing Assignments |
| 56 | 56_ARTIFACT_FWA2Spring24.pdf | .pdf | Example student paper created from Spring 2024 Formal Writing Assignments |
| 57 | 57_ARTIFACT_PosterSpring23.pptx | .pptx | Representative student research poster from Spring 2023 |
| 58 | 58_ARTIFACT_PosterFall23.pptx | .pptx | Representative student research poster from Fall 2023 |
| 59 | 59_ARTIFACT_PosterSpring24.pptx | .pptx | Representative student research poster from Spring 2024 |
| 60 | 60_ARTIFACT_TableS1.docx | .docx | Student statistical table summary |
| 61 | 61_ARTIFACT_TableS2.docx | .docx | Student statistical table from each semester results |
