# Supplementary material for: Introducing a Novel Course-Based Undergraduate Research Experience Using Duckweed as a Model System
Source: Integr Org Biol. 2025 Dec 19;8(1):obaf049. doi: 10.1093/iob/obaf049 (PMC12802901; doi:10.1093/iob/obaf049)
Supplement: obaf049_Supplemental_Files [file obaf049_supplemental_files.zip › 07 Supplementary Materials/Supplementary Materials/00_PREPS_BiologicalMaterialsOriginMap.docx]

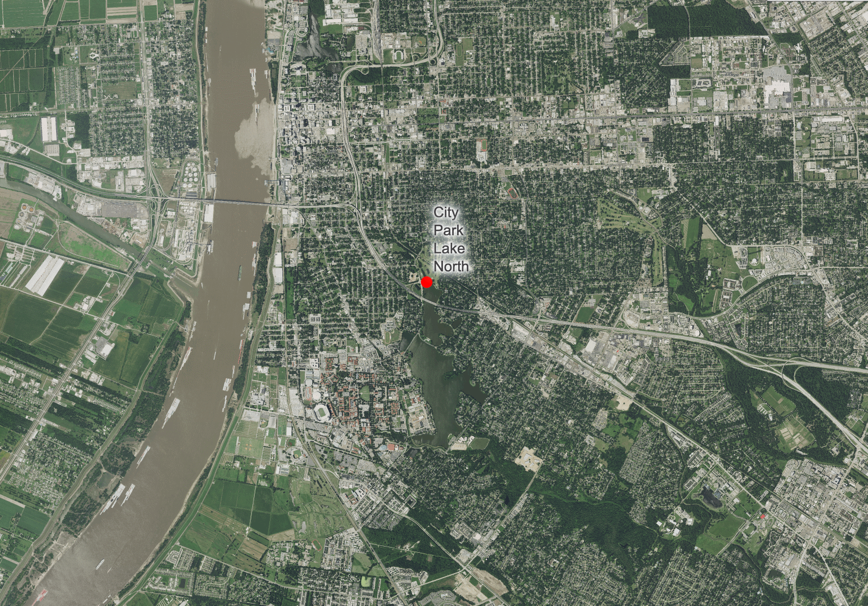

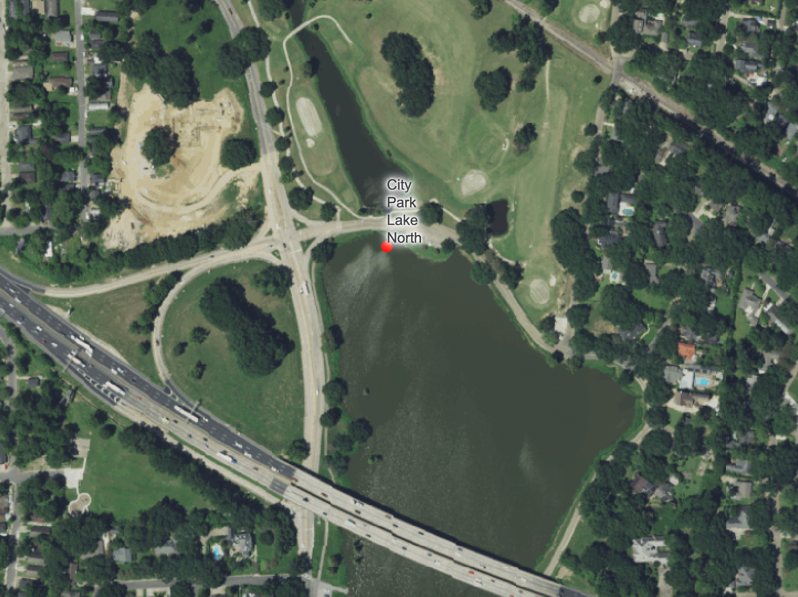


Location of source site for duckweed genotypes and associated microbes. We collected many *Lemna minor* plants from one water body in Baton Rouge, Louisiana during the summer of 2022, 2023 and 2024 and separately cultured the plants and their associated microbes for use during the semester. The coordinates are 30.42911, -91.16784. This site is a pond in a suburban park.
