# Supplementary material for: Introducing a Novel Course-Based Undergraduate Research Experience Using Duckweed as a Model System
Source: Integr Org Biol. 2025 Dec 19;8(1):obaf049. doi: 10.1093/iob/obaf049 (PMC12802901; doi:10.1093/iob/obaf049)
Supplement: obaf049_Supplemental_Files [file obaf049_supplemental_files.zip › 07 Supplementary Materials/Supplementary Materials/01_RESOURCES_LabSafety.pdf]

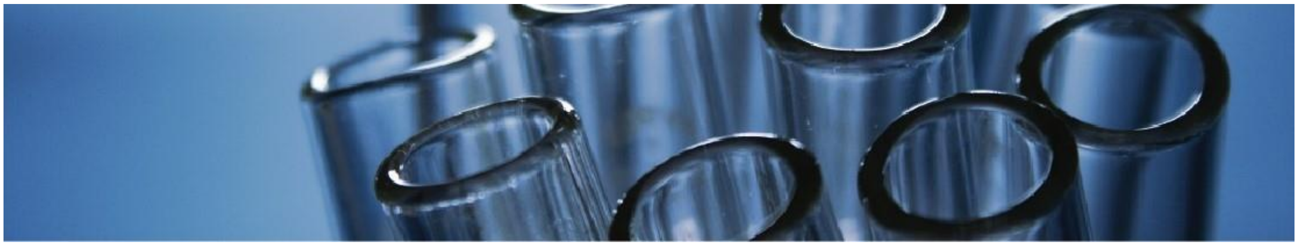

LABORATORY SAFETY

In case of emergency, have someone contact the appropriate people using the following emergency phone numbers:

- LSU Emergency (Fire, Police, Ambulance): 911 (on a campus phone)
- LSU Police; 578-3231
- Student Health Center: 578-6271

There is a phone in either the lab or the adjacent prep room.

General Background Information

Proper laboratory techniques and procedures are essential for creating a laboratory environment that is both productive and safe. Many of the laboratory exercises in this manual involve the use of materials or procedures that can be hazardous to you and others in the laboratory if not handled or performed correctly.

Thoroughly read the following information on laboratory safety equipment and procedures.

After reading the safety procedures, you must read and accept the "Contract to Follow the Laboratory Safety Procedures." You will not be allowed to attend the laboratory until you have read and acknowledged that you accept to follow the contract,

Knowing where laboratory safety equipment is and how to use it can be critical to effectively responding to a laboratory accident.

Covid-19 procedures:

1. If you have any signs of illness, do not come to class.
2. To protect all campus community members, the University requires everyone to wear facemasks/clo[illegible] on campus. Failure to do so is a violation of the code of student conduct.
3. Wash hands with soap and water or clean with sanitizer frequently, and refrain from touching your f[illegible]
4. If you must cough or sneeze unexpectedly, please be mindful of others nearby and cough or sneeze i[illegible] your elbow or shield yourself the best you can.
5. If you have been exposed to others who have tested positive for COVID-19, self-quarantine consiste[illegible] with current CDC guidelines.

Laboratory Safety: Procedures

1. Many laboratory accidents are the result of poor preparation. Carefully reading and understanding what you will be doing in the laboratory will allow you to avoid many possible accidents.

2. Your lab instructor is responsible for the safe and effective operation of the lab. If you have any conditions or special needs that may put you at greater risk in the laboratory (if you are pregnant, color-blind, allergic to any insects or latex, taking immunosuppressive drugs, or have a medical condition that may require special precautionary measures), you should noti%' the laboratory instructor before the laboratory begins.

3. Wearing the appropriate clothing is an important protection preparation for lab. Always wear closed-toe shoes in the laboratory. SANDALS ARE NOT ACCEPTABLE FOOTWEAR.

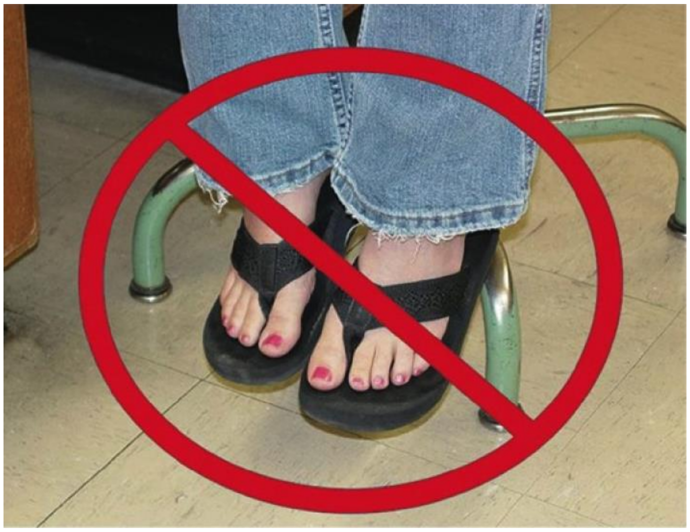

Figure I- I . An example of inappropriate footwear for the laboratory.

4. Exercising care for long hair can prevent many lab-related accidents. Long hair should be tied back to keep it out of your eyes and away from anything that could result in harm.

5. Avoiding certain behaviors in lab can help keep you safe. Do not eat, drink, smoke, or apply cosmetics in the laboratory. Food and drinks may never be brought into the laboratories.

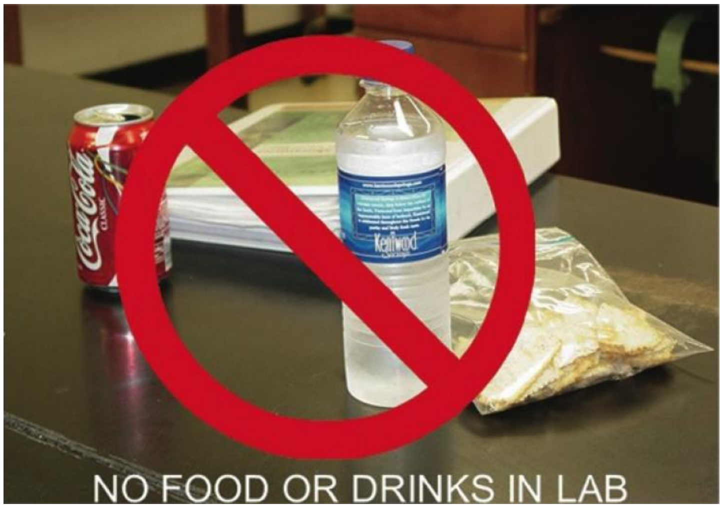

Figure 1-2. Food and drink should never be consumed in the laboratory.

6. Since your hands may come in contact with many things before, during, and after the lab, always wash your hands prior to or immediately after leaving the laboratory. This will prevent external sources of contamination that could alter the result of your experiment. It will also keep you safe from anything you have come in contact with during the lab.

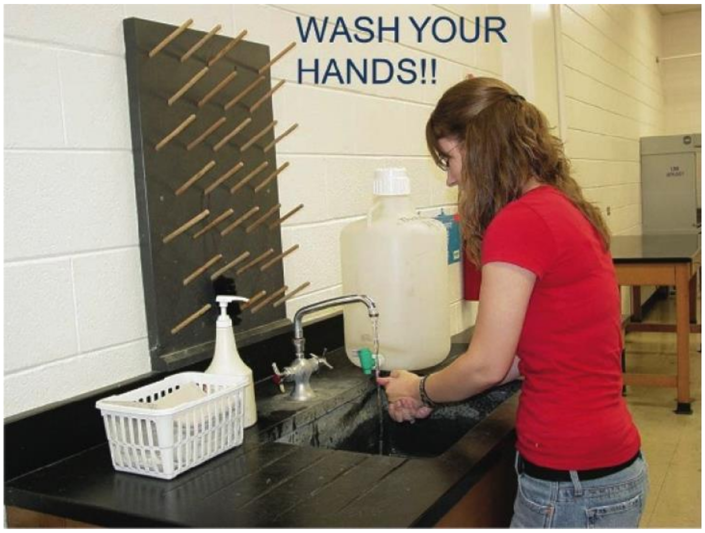

Figure 1-3. Be sure your hands are clean prior to and after completion of the experiments.

7. Come to lab prepared to do the work required and stay focused on the job. Any behavior that distracts you, your group members, or others in the lab could result in possible harm. This includes unauthorized use of cell phones.

Cell phones are on silent and put away!

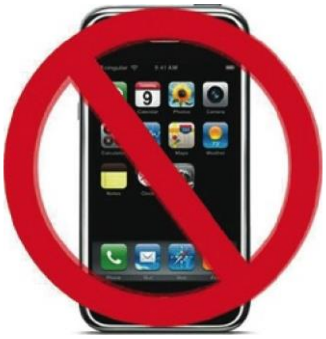

Figure 1-4. Devices like cell phones or mp3 players should not be used in lab unless authorized by the laboratory Instructor. ix

8. While you will often be working in groups, each member should be familiar with all aspects of the lab exercises. *Ihis not only may help keep you safe, but it will also help ensure your lab runs more smoothly and will likely be of benefit to earning a better grade in the course.

9. Accurate and complete notes should be kept in your laboratory notebook during each experiment. Flhis not only provides the basis for safety but can also aid in the completion of many of the post-laboratory events during the course.

10. Read the labels on containers prior to removing any materials from containers. NEVER RETURN MATERIALS TO THE STOCK CONTAINERS. Discard used chemicals and solutions in the appropriate manner. If you are not sure of how to dispose of any materials, be sure to ask your laboratory instructor prior to discarding materials.

I l. Discard broken glassware in the special container marked "Broken Glass." Please do not discard paper or non-glass materials (e.g., plastic pipettes) or biohazardous material in this container. This is important not only for the safety of people working in the lab, but also for the custodians that are responsible for cleaning the rooms.

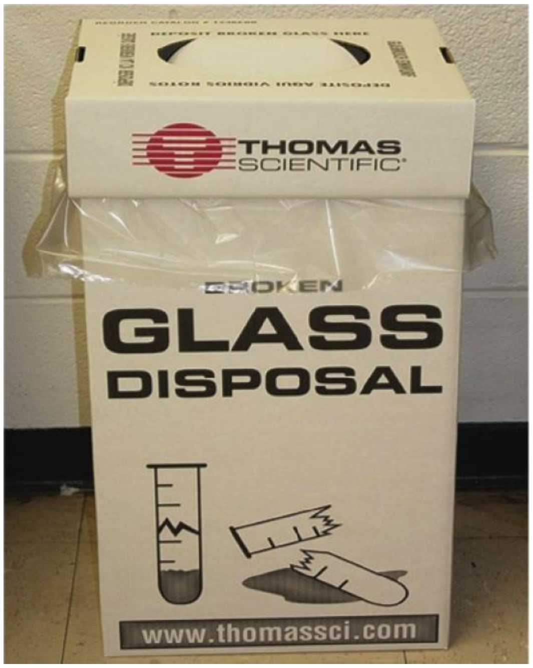

Figure 1-5. Glass disposal only in appropriate container.

12. All "Sharps" (small sharp glass, metal or plastic objects, e.g., coverslips, razor blades) must be placed in the "Sharps Containers" located in each room.
