# Supplementary material for: Introducing a Novel Course-Based Undergraduate Research Experience Using Duckweed as a Model System
Source: Integr Org Biol. 2025 Dec 19;8(1):obaf049. doi: 10.1093/iob/obaf049 (PMC12802901; doi:10.1093/iob/obaf049)
Supplement: obaf049_Supplemental_Files [file obaf049_supplemental_files.zip › 07 Supplementary Materials/Supplementary Materials/03_PREPS_DuckweedFall23.docx]

**Jada Daniels- CURE**

**Preps Spring 2023**

*Room setup: 8 stations across 4 benches (3 students each station); 24 students each section (48 students total)*

**TA: include all prep items along with total quantities that are needed each week. Be as specific as possible. If it is a prep that you will be providing, put your name in parentheses behind the item.**

***Every week:*** *labeling tape, sharpies, gloves, bleach soln 10%, ethanol 70%, discard beakers, biohazard bag*

*ORDER:*

- *R2A Agar*
- Murashige and Skoog Basal Salt Mixture

| **Week 1: Introduction and Practice** | |
| --- | --- |
| - PROTOCOL: Pipetting by Design Lab (24) - Micropipettes & tips (p10 & p100) - Colored water in flasks (8 each: red, blue, yellow) - Water flasks (8; 2 per bench) - 96 Well Plate (24) - Jada provides | - 10mL H2O serological pipettes (3/bench; 12 total) - 10mL green pi pimps (3/bench; 12 total) - Non-sterile test tubes (9/bench; 27 total) - Square test tube racks (3/bench; label tape A-C); |
|  |  |
| **Week 2: Duckweed Bleaching** | |
| - Sink drain strainers (133 & 133A) - Vortex (4) - Forceps (fine point) - Stereoscopes - Compound microscopes - Slides & coverslips (for wet mounts) - kimwipes - Sharpies – fine point - Test tubes (13mm) ( 5 per group, 120 total) - Test tube racks (12) - Duckweed in tubes – Jada providing | - Bunsen Burner (8 - Inoculation Loop (24) - 800 mL dH_2_O (10; 1 per group; pyrex with lid) - 600 mL 1:4 Bleach Solution (10; 1 per group; pyrex with lid) - 100 ml Beaker (30; 3 per group) - Petri Dishes (24; Any Size) - Liquid Discard - PROTOCOL: Microscopy of Duckweed (24) - PROTOCOL: Duckweed Cleaning (24) |
|  | |
| **Week 3: Microbe Plating** | |
| - Micropipettes p10 & p1000   - Sterile tips for both - Practice Cards - 10 ml Seros (30; sterile in wrap) - Sharpies - Test tube racks (12; on benches) - Disposable masks - Lab Coats - Jada will bring samples to plate | - Sterile water in 100 ml flasks (24; ~75 mL each) - Test tubes with caps(100; 50/section)   13mm with caps   - Petri dish holders for incubator - Petri Dishes with R2A (100) - Spreaders (24; sterile) - Bunsen Burner (8) - PROTOCOL: Microbial Plating (24) |
|  | |
| **Week 4: Duckweed Abundance and Microbe Streaking** | |
| - Colored label Tape - Disposable masks - Lab Coats | - Petri dish holders for incubator - Petri Dishes with R2A (100) - Sterile Inoculation loops (48) - Bunsen Burner (8) - PROTOCOL: Streaking Microbial Colonies (24) |
|  |  |
| **Week 5: Duckweed Abundance and Microbial Isolations** | |
| - Cuvettes / Microplates - Hoagland’s media (1:10, no sugar or carbon)   - Recipe: Posted in Week 5   - Volume per test tube - Test Tube racks - 16 mm test tubes w caps (432; sterile) - Sterilize: Hoagland's in tube, capped, autoclaved - Colored label Tape - PROTOCOL: Experimental Set-up: Day 0 (24) | - Inoculation loops (24) - Bunsen Burner (8) - Sterile pipette tips (p100 or p1000)- 2 boxes per bench - Micropipettes p10 & p1000 - Nutrient Broth (20 tubes)   - 8 ml each   - 16 mm test tubes - Disposable masks - Lab Coats - Bunsen burners |
|  |  |
| **Week 6: Microbial Addition** | |
| - Sterile pipette tips (p100 or p1000)- 2 boxes per bench - Micropipettes p10 & p1000 - Masks - Goggles - 96 well plates (Jada provide) - Bunsen burners - Microplate reader spec – Gen5 Jada Provide | - Protocol: Microbial Addition and Data Collection – Day 7 (25) - Kim wipes - Inoculation loops (24) |
|  | |
| Week 7: Fall Holiday  ** No Class ** | |
|  |  |
|  | |
| Week 8: Duckweed abundance and Microbial Enumeration | |
| - Microplate reader spec - Gen5 - Label Tape - Sharpies - **Masks** - **Goggles** - **PROTOCOL: Data Collection Day 21 (24)** | - Sterile pipette tips (p100 and p1000)- 2 boxes of each size per bench - P100 and P1000 pipette - 96 well plates (Jada provide) - Bunsen Burner (8) - Vortex on each bench |
|  | |
| **Week 9: Duckweed Abundance and Microbial Enumeration** | |
| - Microplate reader spec - Gen5 - Label Tape - Sharpies - **Masks** - **Goggles** - **PROTOCOL: Data Collection Day 28 (24)** | - Sterile pipette tips (p100 and p1000)- 2 boxes per bench - P100 and P1000 pipette - 96 well plates (Jada provide) - Bunsen burners |
|  | |
| **Week 10: Final Data Collection & Microbial Plating** | |
| - Microscopes - Ruler - Petri dish holders for incubator - Petri Dishes with R2A (100) - Spreaders (24; sterile) - Test tube return - Test tube cap retun | - Sterile pipette tips (p100 and p1000) - 2 boxes per bench - P100 and P1000 pipette - 96 well plates (Jada provides)   PROTOCOL: Data Collection Day 35 and Experiment Breakdown (24)  PROTOCOL: Microbial Plating W10 |
|  |  |
|  | |
| **Week 11: Lake Study** | |
| - **Microscopes** - **Microbe Field guides** - **Microbe Identification worksheet (24)** | - **Blank slides** - **Slide covers** - **DI Water** - **Dropper** |
|  | |
| **Week 12: Poster Preparations** | |
| - Clipboards (24) - Poster Critique - Elevator Pitches |  |
|  | |
| **Week 13: In Class Poster Presentations** | |
|  |  |
|  | |
| **Week 14: Thanksgiving Holiday** | |
|  |  |
|  | |
| **Week 15: Final Exam / CURE Poster Session** | |
| - Station 1: micropipettes   - P10   - P100   - p1000 - Station 2: Pipetting | - Station 3: Fronds   - Jada will provide duckweed and test tubes - Station 4: posters   - clipboards |
