# Supplementary material for: Introducing a Novel Course-Based Undergraduate Research Experience Using Duckweed as a Model System
Source: Integr Org Biol. 2025 Dec 19;8(1):obaf049. doi: 10.1093/iob/obaf049 (PMC12802901; doi:10.1093/iob/obaf049)
Supplement: obaf049_Supplemental_Files [file obaf049_supplemental_files.zip › 07 Supplementary Materials/Supplementary Materials/04_PREPS_TurionsSpring24.docx]

**Jada Daniels - CURE**

**Preps Spring 2023**

*Room setup: 8 stations across 4 benches (3 students each station); 24 students each section (48 students total)*

**TA: include all prep items along with total quantities that are needed each week. Be as specific as possible. If it is a prep that you will be providing, put your name in parentheses behind the item.**

***Every week:*** *labeling tape, sharpies, gloves, bleach soln 10%, ethanol 70%, discard beakers, biohazard bag*

*ORDER:*

- *R2A Agar*
- Hoaglands
- 6 well plates

| **Week 1: Introduction and Practice** | |
| --- | --- |
| ** No Class** |  |
| **Week 2: Pipetting Practice and Microscopy** | |
| - Micropipettes & tips (p10 & p100) - Colored water in flasks (8 each: red, blue, yellow) - Water flasks (8; 2 per bench) - 96 Well Plate (24) - Jada provides - Sink drain strainers (133 & 133A) - Vortex (4) - Forceps (fine point) - Stereoscopes - Compound microscopes - Slides & coverslips (for wet mounts) - kimwipes - Sharpies – fine point - Test tubes (13mm) ( 5 per group, 120 total) - Test tube racks (12) - Duckweed in tubes – Jada providing | - Bunsen Burner (8) - Inoculation Loop - nonsterile (24) - 800 mL dH_2_O (10; 1 per group; pyrex with lid) - Petri Dishes (24; Any Size) - Liquid Discard - 10mL H2O serological pipettes (3/bench; 12 total) - 10mL green pi pimps (3/bench; 12 total) - Non-sterile test tubes (9/bench; 27 total) - Square test tube racks (3/bench; label tape A-C); - PROTOCOL: Microscopy of Duckweed (24) - PROTOCOL: Pipetting by Design Lab (24) |
|  | |
| **Week 3: Microbe Plating** | |
| - Micropipettes p10 & p1000   - Sterile tips for both - Practice Cards - 10 ml Seros (20; sterile in wrap) - Sharpies - Test tube racks (8; on benches) - Disposable masks - Lab Coats - Jada will bring samples to plate - Test tube / test tube cap Discard - Spreader Discard | - Sterile water in 100 ml flasks (32; ~75 mL each) - Sterile Test tubes with caps (200 total; 50 per section)   13mm with caps   - Petri dish holders for incubator - Petri Dishes with R2A (50 per section, 200 in total) - Spreaders (64; sterile) - Bunsen Burner (8) - PROTOCOL: Microbial Plating (24; PRINT NEW Spring24) |
|  | |
| **Week 4: Photo Annotations** | |
| **Dry Lab** | PROTOCOL: Photo Annotations (24) |
|  |  |
| **Week 5: Mardi Gras Holiday** | |
| - **No Class** |  |
|  |  |
| **Week 6: Experimental Setup** | |
| Hoagland’s media (1:10, no sugar or carbon)   - - Recipe: Posted in Week 5   - Volume per test tube - Falcon tube purple Test Tube racks (12) - 6 well plates, Jada will bring - Sterilize Hoagland in batches. 32 batches total, 200 mL each - Colored label Tape - PROTOCOL: Experimental Set-up: Day 0 (24) | - Inoculation loops (24) - Bunsen Burner (8) - 10 mL Sterile Serological Pipettes (40) - Disposable masks - Lab Coats - Bunsen burners |
|  | |
| Week 7: Data Collection for Turion Germination and Coverage | |
| - Kim Wipes - Colored Label Tape   **Protocol: Data Collection Day 7 (24)** | - Sharpies - Bunsen Burner - Inoculation Loops – do not need to be sterile |
|  | |
| Week 8: Data Collection for Turion Germination and Coverage | |
| - Sharpies - Bunsen Burner - Inoculation Loops – do not need to be sterile | ****Use protocol from last week** |
|  | |
| **Week 9: **Spring Break**** | |
| - No Class |  |
|  |  |
| **Week 10: Final Data Collection & Microbial Plating** | |
| - Petri dish holders for incubator - Petri Dishes with R2A (300 total – 150 per section) - 80 packs of 10 cotton swabs in a bag per group. 800 total | - P10 pipettes – at least 8, one for each group. - Sterile P10 pipette tips (20 for each group, 32 groups total) -   ** each group will need a P10 pipette and their own set of pipette tips.  PROTOCOL: Data collection and experiment Breakdown (24) |
| \| **Week 11: Elevator Pitches and Poster Presentations** \| \| \| --- \| --- \| \| - EVALS PRINT Elevator Pitches (24 per section, 4 sections: 96 total) \|  \| | |
| **Week 12: Lake Study** | |
| - **Microscopes - Microbe Field guides** - **Microbe Identification worksheet (24)** | - **Blank slides** - **Slide covers** - **DI Water** - **Dropper** |
|  | |
| **Week 13: Elevator Pitches** | |
| - Hallway Critiques (48) |  |
|  | |
| **Week 14: Poster Workshop** | |
|  |  |
| \| **Week 15: In class Poster Presentations** \| \| \| --- \| --- \| \| - Poster Evaluations \|  \| | |
| **Week 16: Final Exam / CURE Poster Session** | |
| - Station 1: micropipettes   - P10   - P100   - p1000 - Station 2: Pipetting   - ....   ELAINE ONLY:   - 50 ml of bacterial culture - Plates with colonies from a 10-fold dilution (10^-1^ – 10^-10^ written on the plates) - 10 Test tubes containing 9 ml of sterile water labeled 10^-1^ – 10^-10.^ - Vortex Machine - P1000 micropipette, tips, and Micropipette Stand | - Station 3: Fronds   - Jada will provide duckweed and test tubes - Station 4: posters   - clipboards |
