# Supplementary material for: Introducing a Novel Course-Based Undergraduate Research Experience Using Duckweed as a Model System
Source: Integr Org Biol. 2025 Dec 19;8(1):obaf049. doi: 10.1093/iob/obaf049 (PMC12802901; doi:10.1093/iob/obaf049)
Supplement: obaf049_Supplemental_Files [file obaf049_supplemental_files.zip › 07 Supplementary Materials/Supplementary Materials/05_PREPS_R2A_Recipe.pdf]

R2A AGAR

INTENDED USE
Remel R2A Agar is a solid medium recommended for use in qualitative and quantitative procedures for performing heterotrophic plate counts of treated potable water.

SUMMARY AND EXPLANATION
R2A Agar was developed by Reasoner and Geldreich of the U.S. Environmental Protection Agency for the isolation and recovery of aerobic and facultative heterotrophic bacteria from treated potable water samples.[1] The investigators found R2A Agar to be superior to Plate Count Agar when using the pour plate, spread plate, or membrane filter techniques.[2] The use of a less nutritious medium, lower incubation temperatures, and longer incubation times stimulates the growth of stressed and chlorine-tolerant bacteria recovered from treated waters. Information on water quality, such as heterotrophic plate counts, may be used to evaluate the efficiency of treatment processes. R2A Agar is recommended by the American Public Health Association (APHA) in *Standard Methods for the Examination of Water and Wastewater*.[3]

PRINCIPLE
Peptone supplies essential amino acids and nitrogenous substances necessary for growth. Dextrose is added as a carbon source of energy. Yeast extract supplies B-complex vitamins and serves as a growth enhancer. Soluble starch is added as a neutralizer and sodium pyruvate stimulates the growth of stressed bacteria. Dipotassium phosphate and magnesium sulfate are buffering agents added to maintain osmotic equilibrium. The low nutrients in this medium give higher counts in treated potable waters than a highly nutritious medium.

REAGENTS (CLASSICAL FORMULA)*
Casein Hydrolysate	0.5 g
Dextrose	0.5 g
Soluble Starch	0.5 g
Yeast Extract	0.5 g
Dipotassium Phosphate	0.3 g
Sodium Pyruvate	0.3 g
Casein Peptone	0.25 g
Meat Peptone	0.25 g
Magnesium Sulfate	0.024 g
Agar	15.0 g
Demineralized Water	1000.0 ml

pH 7.2 ± 0.2 @ 25°C

*Adjusted as required to meet performance standards.

PRECAUTIONS
This product is For Laboratory Use only. It is not intended for use in the diagnosis of disease or other conditions.

PREPARATION OF DEHYDRATED CULTURE MEDIUM
1. Suspend 18.1 g of medium in 1000 ml of demineralized water.
2. Heat to boiling with agitation to completely dissolve.
3. Sterilize by autoclaving at 121°C for 15 minutes.
4. Dispense into appropriate containers.

PROCEDURE
1. Consult appropriate references for information regarding water sample preparation and inoculation.
2. Follow procedures outlined in *Standard Methods for the Examination of Water and Wastewater* for the pour plate, spread plate, or membrane filter technique utilizing R2A Agar.[3]
3. Incubate plate(s) according to established laboratory guidelines for processing water samples. The highest counts typically will be obtained from incubation at 20-28°C for 5-7 days. During incubation, maintain humidity within the incubator to prevent the agar from drying out.
4. Consult appropriate references for standard methods in computing and reporting counts. The heterotrophic plate count is determined by dividing the total number of colony-forming units (CFU) or average, in the case of duplicate plates, by the sample volume. The report should include the CFU per volume of sample, the medium and method used, as well as the incubation time and temperature.

Pour Tube: Melt the agar deep in a boiling water bath and cool to 45-50°C. Mix and dispense into a sterile petri dish and proceed with the instructions above.

QUALITY CONTROL
Each lot number of R2A Agar has been manufactured, packaged, and processed in accordance with current Good Manufacturing Practice regulations. All lot numbers have been tested using the following quality control organisms and have been found to be acceptable. Testing of control organisms should be performed in accordance with established laboratory quality control procedures.

CONTROL	INCUBATION	RESULTS
Escherichia coli ATCC® 8739	Aerobic, 18 - 24 h @ 35 - 37°C	Growth
Pseudomonas aeruginosa ATCC® 9027	Aerobic, 18 - 24 h @ 35 - 37°C	Growth

(Continued on back)

BIBLIOGRAPHY

1. Reasoner, D.J. and E.E. Geldreich. 1979. Abstract #N7. Abstracts of the 79th General Meeting of the American Society for Microbiology. ASM, Washington, D.C.
2. Reasoner, D.J. and E.E. Geldreich. 1985. Appl. Environ. Microbiol. 49:1-7.
3. Clesceri, L.S., A.E. Greenberg, and A.D. Eaton. 1998. Standard Methods for the Examination of Water and Wastewater. 20th ed. APHA, Washington, D.C.

Refer to the front of Remel *Technical Manual of Microbiological Media* for **General Information** regarding precautions, product storage and deterioration, specimen collection, storage and transportation, materials required, quality control, and limitations.

ATCC® is a registered trademark of American Type Culture Collection.

IFU 112543, Revised July 11, 2007 Printed in U.S.A.

remel

12076 Santa Fe Drive, Lenexa, KS 66215, USA

General Information: (800) 255-6730 Technical Service: (800) 447-3641 Order Entry: (800) 447-3635

Local/International Phone: (913) 888-0939 International Fax: (913) 895-4128

Website: www.remel.com Email: remel@remel.com
