# Supplementary material for: Introducing a Novel Course-Based Undergraduate Research Experience Using Duckweed as a Model System
Source: Integr Org Biol. 2025 Dec 19;8(1):obaf049. doi: 10.1093/iob/obaf049 (PMC12802901; doi:10.1093/iob/obaf049)
Supplement: obaf049_Supplemental_Files [file obaf049_supplemental_files.zip › 07 Supplementary Materials/Supplementary Materials/06_FORMS_LabPartnerContract.docx]

# **Lab Partner Contract**

# LSU CURE

Some assignments for this course may be completed in partners. These include some assignments related to data analysis, lab reports, and your final scientific posters. To work with a lab partner on these assignments, you must agree to the following:

- Lab partners must contribute equal work on all partner assignments.
- Individual contributions must be listed on all partner assignments.
- Lab partners are equally responsible for all work submitted for partner assignments.
- Lab partners must communicate at least once a week to stay on track with assignments.
- Lab partners must respond to communication with each other within 24 hours.
- Lab partners may not share answers on any individual assignments, including quizzes.
- Partner assignments must be completed using Google Drive or OneDrive to track each partner’s contributions.
- Partners who do not contribute equally may lose the privilege of working with others on large assignments, such as data analysis and the scientific poster.

By typing your name below, you acknowledge that you understand and will adhere to the policies listed above for working with your lab partner. If you fail to follow these rules, the instructor will forward the case to the Office of the Dean of Students for disciplinary action. The consequences for proven violations of the Code of Student Conduct are serious & can result in a failing grade in the class, a permanent transcript notation, suspension, or even expulsion from the University.

**This document must be electronically signed (name & date typed in the fields) & submitted through the Lab Partner Contract Turnitin link on Moodle by Monday, September 14^th^ by 11:59 PM CST.**

|  |  |  |
| --- | --- | --- |
| Name (electronic signature) |  | Date |
