# Supplementary material for: Introducing a Novel Course-Based Undergraduate Research Experience Using Duckweed as a Model System
Source: Integr Org Biol. 2025 Dec 19;8(1):obaf049. doi: 10.1093/iob/obaf049 (PMC12802901; doi:10.1093/iob/obaf049)
Supplement: obaf049_Supplemental_Files [file obaf049_supplemental_files.zip › 07 Supplementary Materials/Supplementary Materials/07_RESOURCES_Syllabus.docx]

# **Biology Laboratory for Science Majors II**

# **BIOL XXXX, Semester Year**

Instructor: **Name**

Department

Email:

Zoom ID:

Student Hours:

Administrator:

Class: Location

Section X: Day, Time

Section X: Day, Time

## ***Classroom Safety***

## Appropriate footwear and clothing are required to avoid cuts and burns. NO OPEN TOE SHOES or CROP TOPS.

## Upon removing your gloves and leaving the laboratory, please wash your hands.

## ***Email Etiquette***

College is a professional setting and students in this course are expected to hold that standard when communicating with their instructors. When corresponding with your instructor over email, you should include your course, section number, lab or assignment in question, and relevant documents. Prior to emailing, you should check Moodle resources and assignment instructions to answer your question. If these guidelines are not adhered to, you should not expect your instructor to respond. If you provide all pertinent information, you can expect a response within 24 hours, not including weekends. Refer to your syllabus lecture for an example of an appropriate email.

#### **Course Overview**

*The focus of this lab course is on you doing science.* The lab course centers around doing science through direct research with duckweed. Students will use duckweed as a model system to explore fundamental questions in plant biology and ecology.. Through hands-on experiments, you will gain experience in experimental design, ecological inquiry, and methods with applications across ecology, environmental science, biotechnology, and agriculture. Together, the class will pursue original research questions, contributing to the broader understanding of duckweed biology. **As a class, you will attempt to answer novel and important research questions that have not yet been answered – this is what makes this course particularly exciting!**

The lab will consist of lectures, group discussions, individual work, and controlled experiments emphasizing antibiotic resistance, mutagenesis, genetic engineering, and basic research protocols (experimental design, data collection, data analysis, and communication). When conducting experimental work, you will work in groups, but outside of class (i.e., writing assignments), you are to complete work independently. If you need help, seek assistance from your instructor first.

Finally, biology labs typically require more work per credit than lectures - this lab is no exception. The expectations and workloads will be challenging. Consider carefully now whether you are up to the task. If so, this lab will give you an informative introduction to conducting basic research in the biological sciences, including molecular biology, and an introduction to some of the natural history of antibiotic resistance and its implications for global health.

This section is a CURE (course-based undergraduate research experience). CURE is a unique program that allows the integration of authentic research projects into introductory biology laboratory courses. Students in CURE freshman biology labs focus on in-house research projects to better understand science and gain confidence in their abilities to do scientific research. CURE courses are communication-intensive with a strong focus on science literacy and will culminate in you designing and delivering a scientific poster about your semester-long research project. As a research-based course, you will collect scientific data and present your findings through communication pieces such as formal writing assignments and scientific posters. This data, along with your writing assignments and scientific posters may be used for publications by course administrators as deemed fit. In this case, all identifying information will be removed. Each section will select class representatives to present their posters at the **CURE Poster Presentation** on **DATE, TIME, LOCATION.** **Attendance is required for all students unless a valid excuse is documented**.

#### **Grading**

| **Assessment** | **Weighted Percentage** |
| --- | --- |
| Quizzes | 15 |
| In-class Assignments (ICAs) | 15 |
| Take-home Assignments (THAs) | 20 |
| Formal Writing Assignments (FWAs) | 25 |
| Scientific Poster | 10 |
| Final Exam | 15 |
| **Total** | **100** |

A+ 97-100% | A 93-96.99% | A- 90-92.99% | B+ 87-89.99% | B 83-86.99% | B- 80-82.99% |

C+ 77-79.99% | C 73-76.99% | C- 70-72.99% | D+ 67-69.99% | D 63-66.99% | D- 60-62.99% | F < 60%

*Quizzes:* You will be quizzed on readings (including the syllabus), lab lecture materials, graphing, homework assignments, etc. Quizzes are likely to cover material from both preceding and forthcoming labs.

*In-class Assignments:* These can consist of the completion of your lab notebook, questions, data analyses, graphs, presentations, peer reviews, or other in-class assignments.

*Take-home Assignments:* Assignments completed at home, such as writing drafts, outlining, literature reviews, etc.

*Formal Writing Assignments: There will be formal writing assignments during the semester, which will introduce reading and writing in a scientific style.* Feedback will be given to assist you in improving your written communication skills.

*Scientific Poster:* Towards the end of the semester, each group will create and ultimately present a conference-quality poster outlining their research project. Guidance will be provided on what makes a good poster.

*Final Exam:* The final exam will be comprehensive and will be based on topics covered during the lab portion of the class, including reading and understanding literature, experimental design, data analysis, presentation, and interpretation, your research throughout the semester, and practical techniques.

#### **Course Policies**

*Submitting assignments:*

You should save your assignments as **PDF** files and submit them as such. All assignments must be submitted with the abbreviated title followed by **your first and last names**. Please, see examples:

ICA_DilutionCalculations_JohnDoe

THA_ReadingPaper_JaneDoe

FWA1_JohnDoe

*Late Assignments:*

**Take home Assignments (THAs):** THAs are due by 11:59 pm on Mondays. Late submissions are not accepted for these asynchronous assignments. **Computer issues or blank submissions are not a valid reason for submission issues**.

Assignments other than THAs may be submitted late, up to 72 hours, with penalty. These late assignments will incur a 10% grade reduction for every 24-hour window that the assignment is late. For example, if an assignment is ten minutes late or 20 hours late, the penalty will be a 10% reduction in score. In addition to THAs, some other assignments, such as Formal Writing Assignments, PeerMark, and the final exam, cannot be accepted as late without a valid excuse. Internet and computer issues are not valid excuses for not submitting an assignment on time. Likewise, not monitoring your university email account for correspondence, whether from your instructor or lab partner, is not a valid excuse for missing information. Please plan accordingly if your internet or computer is not reliable. **Assignments submitted more than 72 hours late (3 days) will not be accepted.**

*Attendance and Makeup Work:*

Your presence and participation in the Lab are mandatory. There is limited opportunity to make up for a missed class. If you are unable to attend class for a university accepted excuse, you will be assigned makeup work. It is your responsibility to plan for missed classes - your instructor will not track you down. You must contact your instructor prior to your absence and provide documentation for your absence to be excused. Appointment confirmations may not suffice as documentation. If you miss class for any other reason (not University excused), you miss the points for that class. See university policy regarding absences. **No make-up work will be allowed for students missing more than two class meetings. Likewise, even with an excused absence, makeup work will not be accepted beyond ten days after the missed class.**

**Code of Misconduct**

*10.1 Academic Integrity/Plagiarism:*

It is your responsibility to refrain from plagiarizing the academic property of another and to utilize appropriate citation methods for all coursework. Ignorance of the citation method is not an excuse for academic misconduct. Remember, there is a difference between paraphrasing and quoting and how to properly cite each respectively. One tool available to assist you in writing correct citations is the “References” function in Microsoft Word. This program can generate a reference page according to the citation method you select for your document. A demonstration of how to use this tool is available online at the Student Advocacy & Accountability page. Although you will be working in groups in the laboratory, all written assignments are to be individually written. **Do not share your work with another student**, as the student who shares their work is just as guilty of plagiarism as the student who uses the shared work. Any assignment suspected of plagiarism will be submitted to the Student Advocacy & Accountability office for review.

*10.2 Behavioral Misconduct:*

To ensure a safe and productive learning environment in the biology lab, students are expected to be fully alert and in control of their faculties at all times. **If a student appears to be under the influence of alcohol or drugs, they will be asked to leave the lab immediately and will be reported to the Office of Student Accountability.** This includes, but is not limited to, signs such as impaired behavior, slurred speech, erratic movements, or other indications of intoxication or impairment. **If there is reasonable suspicion that a student possesses or has recently used alcohol or drugs—such as the odor of alcohol or marijuana on their person or belongings—this will also result in removal from the lab and a report to the appropriate office.** These measures are in place to protect the safety of all students and staff, as laboratory environments involve potentially hazardous materials and equipment. Please review and follow all university policies regarding substance use. Violations may result in disciplinary action.

*Disclaimer on Course Flexibility:*

The syllabus, the schedule listed below, and assignments may be changed at any point in the semester. In fact, because of weather and other logistical uncertainties related to the dynamic nature of field/experimental microbiology and molecular biology, you should expect changes. So, be flexible. Also, course instructors are given latitude to emphasize their own research and teaching strengths. Thus, the material covered, and the specific requirements of each assignment may differ among semesters. If you have any problems during the semester, please contact the course coordinator.
