# Supplementary material for: Introducing a Novel Course-Based Undergraduate Research Experience Using Duckweed as a Model System
Source: Integr Org Biol. 2025 Dec 19;8(1):obaf049. doi: 10.1093/iob/obaf049 (PMC12802901; doi:10.1093/iob/obaf049)
Supplement: obaf049_Supplemental_Files [file obaf049_supplemental_files.zip › 07 Supplementary Materials/Supplementary Materials/08_PREPS_WeeklyActivityDescriptions.docx]

***Weekly Activity Descriptions***

***Week 1: Introduction to the Course and Laboratory Techniques***
 In week 1, students learn about laboratory practices and techniques, such as pipetting skills, to assist with activities covered later in the course. The first week of instruction will be used to review the class syllabus, laboratory classroom safety rules, academic integrity, and course structure. The instructor will then introduce lab techniques and explain how to use and read a serological pipette and micropipette. The remainder of the lab time will be spent practicing pipette techniques. Students will be given a protocol with multiple versions of instructions, a micropipette, three colors of water, and a 96-well plate. Each version of instructions corresponds to an image that can be created within the 96-well plate using the dyed water. Students will then clean and dispose of their materials properly and are dismissed from class. After class, students will complete a syllabus quiz on the course website to review the syllabus.

***Week 2: Microscopy Techniques and Study System***
 In week 2, students will learn microscopy techniques and sterile handling. Before the class on week 2, the instructor will prepare axenic duckweed clusters (recommended 5-10 per student with varying frond counts), sterile water, 25% sodium hypochlorite solution, Bunsen burners, and inoculation loops. The instructor provides all axenic fronds, and students perform the process solely to understand the technique and practice sterile handling. In week 2, the class will commence with a lecture on the significance of microscopy and its practical applications. Following this, the instructor will introduce *Lemna minor*, explaining its ecological and scientific importance. Each student will receive a test tube containing one cluster of duckweed and will learn the techniques of using inoculation loops and Bunsen burners to ensure sterility while transferring duckweed to a petri dish and preparing a wet mount. Students will examine the duckweed under both dissecting and compound microscopes to appreciate the differences between these tools. For the in-class activity, students will sketch and label a duckweed cluster from their microscopic viewings. To minimize confusion during future data collection, students will receive instruction on how to identify and count individual duckweed fronds. During microscopy and sketching exercises, the instructor will review key features that distinguish fronds and provide examples of atypical cases, such as overlapping or newly budding fronds. This will help ensure consistency across lab groups when frond counts are used as a primary growth metric later in the semester. After the instructor approves their drawings, students will proceed to the bleaching process—a method to achieve an axenic state in field-collected aquatic plants. To accomplish this, students will first distribute 6 mL of sterile water into five test tubes using serological pipettes. Unless damaged during microscope viewing, the same duckweed specimens will be used. Students will rinse the duckweed in sterile water and, using a sterilized inoculation loop, transfer it to the bleach solution (1% sodium hypochlorite) and then rinse it again in sterile water. Throughout the process, students must practice sterile techniques, such as flaming the inoculation loop between uses. To prepare the samples for the study, each student will place one bleached duckweed cluster into a test tube of sterile water, ending up with five test tubes each. This exercise emphasizes sterile handling techniques and the preparation of biological specimens for laboratory analysis. For a take-home assignment, students will be given instructions on how to write a ‘Methods Summary’. Students will be given approximately 5 days to complete a methods summary of the bleaching process for submission on the course management system.

***Week 3: Plating Microbial Communities and Find Scientific Literature***
 In week 3, students will learn about microbial plating and literature searches. Before the class on week 3, the instructor will set up stations with required materials for microbial plating, including micropipettes with sterile tips, Bunsen burners, test tubes with caps, and petri dishes containing R2A agar. R2A (Reasoner's 2A) agar is a low-nutrient medium commonly used to cultivate heterotrophic bacteria from environmental samples. It is ideal for CURE experiments because its low nutrient content supports the growth of slow-growing environmental microbes, allowing students to observe a broad diversity of bacteria. Students will perform serial dilutions and microbial plating, focusing on maintaining sterile conditions throughout the process. Students will use prepared monocultures to plate alongside a sterile water sample as a control. After plating, the instructor will label, and group plates based on lab groups. All plates will be placed in the incubator at 30°C until the next class period. After properly disposing of all wet lab materials, students will be tasked with a literature search assignment. . They will identify relevant scientific literature related to their assigned research topic using Google Scholar and practice summarizing key findings. To support this process, the instructor provides a live demonstration of effective keyword selection and discusses strategies for identifying peer-reviewed sources. This will help develop their skills in locating and evaluating scientific literature pertinent to their project. This assignment may be started in class but will be finished at home and submitted via the course website. For the turion version of the course, microbial plating will be used to check the difference between wild, non-axenic duckweed plants and axenic duckweed plants, again using sterile water as a control for absence of microbes. In-class and Take-Home assignments are the same for both course versions.

***Week 4: Streaking Microbial Colonies and Evaluating Scientific Literature***
 In week 4, students will learn about microbial isolation and critical literature comprehension. During the fourth week, the instructor’s preparations will include materials necessary for microbial streaking procedures and choosing key papers relevant to their research project. Stations are prepared with sterile inoculating loops, Bunsen burners, petri dishes, and other requisite tools. Before the wet lab begins, the instructor will lecture on the importance of scientific literature and how to evaluate potential sources. The students will then walk through the experiment set-up and procedures. Students will then engage in streaking microbial colonies, practicing the transfer of bacteria from a single colony to a fresh agar plate to cultivate pure bacterial cultures. This process will emphasize proper laboratory techniques, requiring students to sterilize the inoculating loop between transfers and carefully label each petri dish to track bacterial growth. The in-class assignment will involve students summarizing the streaking protocol they practiced, emphasizing the importance of sterile techniques and methods writing in microbial research. For their take-home assignment, students will be tasked with reading and summarizing key papers provided by the instructor on the study topic. Students will also begin compiling their bibliography for Formal Writing Assignment 1 (FWA1) for submission via the course management system. Students will create citations based on the scientific papers they have read, summarizing each paper's relevance to their project, main findings, and the implications of these findings on their study. This task is designed to deepen their understanding of existing literature, help frame their research within the broader scientific context and provide examples of scientific writing to better understand literature structure. For the turion version of the course, students will use five provided photos of turions in a 6-well plate to practice collecting data using ImageJ. The instructor will demonstrate to students how to upload photos to ImageJ and how to use the select and measure tools to collect percent frond area coverage and count fronds as a measure of growth. The in-class assignment will include students summarizing the data collection protocol. The take-home assignment remains the same for both course versions.

***Week 5: Experimental Setup and Introduction to Scientific Writing***

In week 5, students will learn about experimental design and scientific writing. The instructor will need to prepare sterile growth media in test tubes of varying sizes to represent different habitat sizes, axenic duckweed, sterile R2A broth in test tubes, and a lab polyculture of duckweed-associated microbes. Students will first examine their streaked plates to determine purity of the bacteria cultures. Students will then follow the given protocol to create a monoculture using the colonies from the streaked plates and the R2A broth. Note that students will go through the process of collecting a field sample and preparing it for experimental use but will not use the duckweed or bacteria cultures created during the class time for the experiment. The instructor will provide new axenic duckweed and polycultures for the experimental setup to ensure standardization across lab groups and sections. Once students have completed creating their monocultures, the instructor will explain the importance of purifying the bacteria cultures for experimental use and the experiment overview. After cleaning up their lab stations, students will begin preparing to set up their experiment. For the plant-microbe version of this course, students will be given pre-sterilized glass test tubes of three varying sizes (10 mm, 13 mm, 16 mm), each filled with 8 mL of a 1:10 Hoagland’s plant growth medium solution and one glass test tube of axenic duckweed containing at least 30 clusters. Students will add one duckweed cluster to each test tube of growth media. Students will then record the number of fronds in each test tube for their first data collection. Once finished, the instructor will place all experimental test tubes on the plant rack. In experiments where temperature was not an experimental variable, duckweed cultures were maintained at a stable room temperature of approximately 22–24°C. Grow lights used for both versions of this curriculum are full spectrum fluorescent LED bulbs. For all experiments, grow lights were positioned approximately 15–20 cm above the top of the test tubes or well plates. Each light illuminated a group of 72 test tubes or 6 well plates. We used the GooingTop LED Grow Light 6000K Full Spectrum Clip Plant Growing Lamp with White Red LEDs for Indoor Plants.

For the turion version, students will be given one tube of turion pods, three 6-well plates, and 250 mL of sterile plant growth media. Students will use pipettes to transfer 8 mL of plant growth media into each well. Using sterile inoculation loops, students will add one turion to each well. Students will record the number of fronds and measure per cent area coverage of each well for their first data collection. Turions will be placed into the incubators, preset by the instructor with the temperature settings.

For the in-class assignment for week 5, students will outline the Introduction section for their semester paper. This outline should broadly discuss research questions and hypotheses about habitat fragmentation effects on plant-microbe interactions and introduce the biological and ecological significance of duckweed. The take-home assignment will involve continuing the development of an annotated bibliography. Students will also submit the first draft of their formal writing assignment (FWA1). The FWA1 is comprised of the Introduction, Methods, and Bibliography sections.

***Week 6: Collecting Data and Peer Reviews***

In week 6, students will learn about data collection and peer review. Before the sixth week class, the instructor will prepare the laboratory by ensuring all stations are equipped with sterile pipettes, Bunsen burners, and microplates necessary for microbial addition to the ongoing duckweed experiment. The instructor will also ensure that all materials required for data collection, including laptops for running ImageJ and equipment for measuring optical density, are ready for use.

For the plant-microbe version, students will be guided on how to add microbial solutions to their test tubes using sterile techniques to prevent contamination and ensure accurate experimental results. In the wet lab portion of the class, students will perform the microbial addition, carefully measuring and recording the volume of microbial solutions added to each sample tube. Students will then pipette a small amount of liquid from each test tube into a 96 well plate to measure optical density and count the number of fronds in each test tube. This will be week 1 of data collection, establishing the protocol that will be followed consistently throughout the 28-day experiment.

For the turion version, students will count the number of duckweed fronds and use ImageJ to measure the percentage area coverage of duckweed in their samples. This will be the second data collection event for students, and the standard method for data collection in the turion version of this course.

The in-class activity will involve a detailed examination and discussion of methodology based on primary literature. Students will dissect various methods used in key papers and color-code text segments according to the types of methodology (e.g., experimental design, data collection, data analysis) to better understand how to construct their methods sections for their own papers. For the take-home assignment, students will engage in peer review of their classmates' papers (which were submitted before class). This peer review process is designed to refine their analytical skills and improve their own writing based on the feedback they provide and receive. This activity encourages critical thinking and detailed examination of scientific writing, helping students enhance the clarity and impact of their own research papers.

***Week 7: Understanding Scientific Writing and Data Collection***

In week 7, students will learn about writing methodology sections and refining data collection techniques. The lecture will discuss the nuances of scientific writing with a specific focus on writing methods sections effectively. This discussion aims to equip students with the skills needed to describe their procedures in a manner that is both concise and replicable. During the wet lab, students will engage in another round of data collection, marking Day 14 of the ongoing experiment. For the in-class activity, students will dissect methodologies from key papers, analyzing the techniques and experimental designs used in similar studies to help shape their own methods writings. This exercise is intended to help them understand how to structure their own methods sections for their formal writing assignments. The take-home assignment for week 7 will involve students developing the second draft of their formal writing assignments (FWA1), particularly focusing on refining their methods sections based on the dissections, peer reviews, and discussions in class.

***Week 8: Writing Scientific Results and Data Analysis***

In week 8, students will learn about data analysis and presenting results. Before week 8, the instructor will ensure that all instructions for downloading statistical analysis tools, including Excel, JMP, and specific Python notebooks, are available on the course management system. The necessary documents and tutorials for statistical analysis using these tools will also be used to walk students through the process. The lecture will focus on interpreting statistical data and understanding the significance of results in scientific studies. This discussion will emphasize the importance of statistical tools in analyzing biological data. In the lab, students will engage in advanced data analysis of the results collected from their ongoing duckweed and microbial experiments. They will use Excel and JMP to perform statistical tests, including ANOVA and regression analyses, to determine the significance of their experimental results. For the turion version of this course, students will be provided with Python notebooks via the course management system to analyze data and visualize trends. For the in-class activity, students will create figures and tables that accurately represent their experimental data. They will use JMP (or Python) to generate line graphs and bar charts that illustrate the growth of duckweed and microbial concentrations, or percent coverage and frond count over time, ensuring each figure has a complete caption and is ready for inclusion in their formal writing assignments. The take-home assignment will involve students completing a data analysis report, which includes the beginning stages of a results section for their formal writing assignment 2 (FWA2). This report should include figures, tables, complete captions, and preliminary discussions on the trends observed in their experimental data up to 14 days. Students will use this data analysis to add data from future weeks for their final data analyses. This task aims to integrate their practical data analysis skills with scientific writing, preparing them for future scientific communications.

***Week 9: Developing Results and Discussion***

In week 9, students will learn about data interpretation and writing discussion sections. The week 9 lecture will focus on the analysis and interpretation of the complete dataset collected over the experiment. Students will be taught how to critically analyze data and discuss the implications of their findings in the context of existing scientific literature. In the wet lab, students will conduct the final data collection for their project, marking Day 28. Each student will record their data using a template provided on the course management system, ensuring accuracy and consistency in their final data set. For the in-class activity, students will dissect a scientific paper, focusing on how the results and discussion sections are structured and written. They will analyze how trends, significant values, and figure references are integrated into the narrative to support the study's conclusions.

The take-home assignment will involve students developing the Results and Discussion sections of their formal writing assignments. Students will use the feedback received on their data analysis assignment to refine their captions and trend sentences. Additionally, students will compile information for the Discussion section, with includes restating hypotheses, discussing whether the data supports or rejects these hypotheses, and interpreting their findings in a broader scientific context. This assignment helps students to articulate their research findings clearly and place their work within the larger framework of scientific knowledge.

***Week 10: Lake Study and Study System Origin***

In week 10, students will learn about field sampling and aquatic ecosystems through a visit to a local lake (University X Lakes for our course). While this week is not specific to the Duckweed curriculum, it is a crucial part of an ecology lab. For our university, this activity is scheduled in week 10 of the Fall semester, which works well in the Southeastern subtropical climate. However, in northern regions or colder climates, this timing may not be ideal, as week 10 often coincides with the onset of winter in Fall Terms. In such cases, instructors could adapt the activity to occur earlier in the semester, such as week 2, when conditions are more conducive. Before the week 10 class, the instructor will ensure that all necessary materials and equipment for the lake study are prepared and ready. This will include sterilization of supplies, personal protection equipment (PPE), such as gloves, goggles, lab coats, and collection tools like bottles and petri dishes. The lecture this week will focus on the importance of biodiversity in aquatic ecosystems, specifically discussing the roles and interactions of different organisms within lake ecosystems. Students will learn about the significance of biotic and abiotic factors in shaping these communities and the natural history behind their study system – *Lemna minor* or *Spirodela polyrhiza*. Students will be expected to be able to identify the importance of duckweed in natural ecosystems.

During the field study at University X Lakes, students will collect water samples. They will follow strict protocols to avoid contamination during sample collection and handling. The collection process will involve securing water samples that include vegetative particulates, which are likely habitats for microorganisms. In the wet lab, students will observe their samples under stereoscopes and compound microscopes. They will identify various microorganisms using identification guides from the iNaturalist ACC common pond water sample organisms online guide (https://www.inaturalist.org/guides/7609).

For the in-class activity, students will analyze their observations and create food chains based on the organisms they identify using the given identification guides. They will discuss how biotic, abiotic, and human influences affect their specific collection sites and the overall health of the ecosystem. The take-home assignment involves preparing a detailed report and presentation on their findings. Students will include photographs of their collection sites, detailed descriptions of observed organisms, and discuss the ecological health of the lake based on the diversity and roles of the organisms they identified. This assignment emphasizes the relevance of ecological studies to real-world environmental challenges and conservation efforts. Students will also be expected to revise FWA2 before submitting via the course website.

***Weeks 11 - 12: Creating and Reading Scientific Posters***

In week 11, students will learn about scientific communication and poster design. During the class session, students will work in teams to create their posters, utilizing the best elements from each partner’s formal writing assignments. They will be encouraged to integrate text and graphics seamlessly to create an engaging and informative poster. The session will be focused on collaboration, with students discussing and deciding on the content layout, design choices, and the division of work. For the in-class activity, students will engage in hallway critiques of existing scientific posters displayed around the building. Students will be guided in the lecture through examples of strong and weak scientific posters to help them critically evaluate design and content. This exercise will aim at sharpening their critical thinking and analytical skills by evaluating the effectiveness of various poster designs and content choices. They will be expected to rate the posters on several criteria, such as clarity, organization, and visual appeal, and consider these insights when designing their own posters. The tin-class assignment will be dedicated to helping student develop and finalize their posters, ensuring all elements are well-aligned, visually pleasing, and informative. Students will need to adhere to the design tips provided, such as appropriate use of colors, text readability, and maintaining a logical flow of information. This task will culminate the preparation for the poster presentation, where they will communicate their research findings from their experiments to engage with an audience, simulating a real-world scientific conference which they will present in class in the following weeks.

***Week 13 - 14: Scientific Communication and Semester Conclusion***

In weeks 12-14, students will learn about presenting research and peer feedback through activities like participating in poster workshops and giving presentations at the poster symposium. In week 13, student poster presentations are assessed in class using a specified rubric available to students via the course website. Notably, while an in-person poster symposium would be ideal, posters are not physically printed but instead projected for in-class presentations. Presentations will primarily focus on the discussion and future directions of the research, leveraging common experimental data but highlighting unique interpretations and connections to broader literature. This will allow students to communicate their science effectively in an oral or multimedia format. Following the presentations, the instructor conducts a final exam review and provides time for students to ask questions if needed. In preparation for week 14, the instructor readies the final exam, holds a final exam review session and optional practical stations, and then oversees the administration of the final exam. The poster symposium is scheduled after the exam.

Selected students from multiple CURE sections within the Department of Biological Sciences and the College of Science come together to present their research at the CURE Poster Symposium. Typically, 2–3 posters are selected from each lab section for formal printing and public presentation, based on criteria such as research quality, clarity, and design. The symposium provides a professional setting where students present their findings to peers, faculty, and other guests, simulating the experience of a scientific conference. Meanwhile, all students, including those whose posters are not printed, actively participate by attending, offering constructive feedback, and engaging with the presented research. This shared event helps to foster a sense of scientific community, encourages cross-group learning, and gives students the opportunity to practice both giving and receiving professional scientific feedback.
