# Supplementary material for: Introducing a Novel Course-Based Undergraduate Research Experience Using Duckweed as a Model System
Source: Integr Org Biol. 2025 Dec 19;8(1):obaf049. doi: 10.1093/iob/obaf049 (PMC12802901; doi:10.1093/iob/obaf049)
Supplement: obaf049_Supplemental_Files [file obaf049_supplemental_files.zip › 07 Supplementary Materials/Supplementary Materials/09_Week02_RESOURCES_MicroscopePartsAndSafety.docx]

# Microscope Parts & Safety

The microscope is a basic tool of biology that you will use throughout your academic career. In this laboratory you will work with a compound microscope. In order to derive the maximum benefit from this microscope, you should learn the names and functions of the various parts.

#### *Parts of the microscope*

- Base: The base supports the other parts.
- Arm: The C- or L-shaped arm supports the stage and body-tube.
- Stage: The stage is the horizontal platform which supports the object, usually a slide, to be examined. The slide may be held in place by the clips on the stage, or the clips may be removed.
- Body-tube: The body-tube bears the lenses of the microscope - the eyepiece or ocular lens at the top, and the objective lens just above the stage.
- Eyepiece: The eyepiece or ocular lens generally magnifies the image 10 times. It may contain a pointer that can be moved by rotating the eyepiece.
- Nosepiece: The nosepiece bears the objective lenses. To position an objective lens above a specimen, rotate the nosepiece until the lens clicks into position.
- Objective lenses: Most of the microscopes bear three objective lenses: scanning (4x), low power (10x), and high power (40x). *To calculate the total magnification of an object viewed through the microscope, multiply the eyepiece magnification times the objective lens magnification. As an example, if a cell is viewed through the low power (10x) objective, and the microscope has a 10x eyepiece, the total magnification of the cell is 10 x 10 = 100x.*
- Focus knobs: The image is focused by turning the large **coarse focus knob** and/or the small **fine focus knob**. The focus knobs raise or lower the stage to position the specimen at the point where the objective lens is focused. **The course focus knob should be used only when using the 4x objective**.
- Light source: The light is turned on and off by turning the switch near the left side of the base. Don't forget to plug in the cord!
- Condenser: The condenser collects light from the source to pass through the specimen. The condenser should be positioned about 1/8” below the stage.
- Iris diaphragm: The condenser iris diaphragm adjusts the intensity of the light passing through the sample. It is roughly equivalent to the contrast control on a television. Opening the diaphragm allows more light to pass through, which is critical in viewing dense objects or objects at high magnification. Closing the diaphragm, in addition to protecting your eyes from intense light, allows you to examine objects at low magnification with high contrast.

#### *Safety Tips*

- Always start on the lowest magnification and the stage all the way down.
- Coarse focus is used ONLY with the 4x objective lens. Fine focus can be used for all three objective lenses.
- Use the safety clip correctly. It holds the slide in place, it doesn’t go on top of the slide.
- Never touch the lens with your fingers and always clean them with lens paper.
- Be very careful when changing the objective lens! We don’t want to break the lens or the slide we are looking at by having the stage up too high and the lens hitting it.
