# Supplementary material for: Introducing a Novel Course-Based Undergraduate Research Experience Using Duckweed as a Model System
Source: Integr Org Biol. 2025 Dec 19;8(1):obaf049. doi: 10.1093/iob/obaf049 (PMC12802901; doi:10.1093/iob/obaf049)
Supplement: obaf049_Supplemental_Files [file obaf049_supplemental_files.zip › 07 Supplementary Materials/Supplementary Materials/10_Week02_PROTOCOL_CompoundMicroscopeStereoscopes.docx]

# LAB: Compound Microscopes & Stereoscopes

## EXERCISE: COMPOUND MICROSCOPE Basics

1. **Before you start, wipe the following parts with an alcohol pad: eyepieces, nosepiece, focus knobs.**
2. **Squirt EtOH into your gloved hands.**
3. Obtain an ‘e’ slide from the front table.
4. Lower the stage using the coarse focus knob and position the slide so that the ‘e’ is directly above the condenser.
5. Rotate the nosepiece to 4X objective lens and position it directly above the ‘e’.
6. Turn-on the light source and look through the eyepiece. Adjust the diaphragm to a comfortable light intensity.
7. Use the coarse adjustment knob to move the stage as close to the lens as it will go.
8. While looking through the ocular, use the coarse adjustment knob to move the stage away from the objective lens until the image is in focus.
9. Adjust the power intensity knob for the proper amount of light.
10. Adjust the fine focus knob to bring it into proper focus.
11. Slowly move up to the next level of magnification, 10X objective.
12. Adjust the power intensity, fine focus knob, and diaphragm accordingly until it focuses. **Note: do not use the coarse focus knob when using the 10x or 40x** objective lens, as it will break the lens and slide.
13. Slowly move up to the next level of magnification, 40X.
14. Adjust the power intensity, fine focus knob, and diaphragm accordingly. **Note**: **do not use the coarse focus here.**
15. Lab Notebook prompts:
    - *Did the letter appear in the same orientation when viewed through the microscope as viewed without the microscope?*
    - *When you move the slide to the right what direction does it appear to move under the microscope?*
    - *What happened to the image when you switched objectives?*

## Exercise: Preparing wet mounts of microscopic specimens

1. **Before you begin, wipe contact areas including the ocular lenses, nosepiece, and focus knobs.**
2. **Squirt EtOH into your gloved hands.**
3. Ensure microscope is in starting position: stage lowest, 4x objective lens in position.
4. Obtain a glass slide, cover slip, and transfer pipette from the supply bench.
5. Draw-up a sample specimen using a transfer pipette and place one drop at the center of the slide.
6. **At an angle**, place the cover slip against the slide over your specimen. Note: the slip is glass and will break.
7. Position the slide so that the sample is directly above the condenser using the x/y knobs located on the stage.
8. Turn-on the light source and look through the eyepiece. Adjust the diaphragm to a comfortable light intensity.
9. Use the coarse adjustment knob to move the stage as high as it will go.
10. While looking through the ocular, use the coarse adjustment to move the stage away until image is in focus.
11. Adjust power intensity knob for the best viewing and the fine focus knob to bring it into perfect focus.
12. Ensure that your specimen is directly in the center of your field of view using the x/y knob on the stage.
13. The y and x-axis knobs located on the stage can be used to move the slide to search for more specimens.
14. Slowly move up to the next level of magnification, 10X objective.
15. Adjust the power intensity, fine focus knob, and diaphragm accordingly until it focuses perfectly.
16. **Note:** **Do not use the coarse focus knob when using 10x or 40x objective lens;** it will break the lens and slide.
17. Slowly move up to the next level of magnification, 40X.
18. Adjust the power intensity, fine focus knob, and diaphragm accordingly. **Note: do not use the coarse focus.**

## Exercise: Stereoscope

1. **Before you begin, wipe contact areas including the ocular lenses, nosepiece, and focus knobs.**
2. **Squirt EtOH into your gloved hands.**
3. Place petri dish with specimen on stage. Include water in the dish if needed.
4. If doing wet mounts on slides:
   1. Obtain a glass slide, cover slip, and transfer pipette from the supply bench.
   2. Draw-up a sample specimen using a transfer pipette and place one drop at the center of the slide.
   3. **At an angle**, place the cover slip against the slide over your specimen. Note: the slip is glass and will break.
5. Place white or black paper underneath your dish; depends on which allows for better visibility of your specimen.
6. Adjust ocular lenses to fit your eyes.
7. Rotate knob on arm (moved body of scope up and down) until specimen becomes visible.
8. Adjust course zoom knob (side of nosepiece). There are four stops with varying magnification (7, 15, 25x)
9. Adjust left ocular lens for finer magnification if needed.

## CLEAN-UP PROCEDURE

1. Set the microscope back to its starting position: stage down, 4X objective lens in place
2. **Clean the benchtop well with EtOH and paper towels.**
3. Push chairs under bench.
4. Discard gloves in the biohazard bag.
5. **Wash your hands upon leaving classroom.**
