# Supplementary material for: Introducing a Novel Course-Based Undergraduate Research Experience Using Duckweed as a Model System
Source: Integr Org Biol. 2025 Dec 19;8(1):obaf049. doi: 10.1093/iob/obaf049 (PMC12802901; doi:10.1093/iob/obaf049)
Supplement: obaf049_Supplemental_Files [file obaf049_supplemental_files.zip › 07 Supplementary Materials/Supplementary Materials/11_Week02_PROTOCOL_DuckweedBleaching.docx]

**BIOL 1503**

**Cleaning Protocol**

**INTRODUCTION**

This protocol describes the process for cleaning the collected duckweed for them to reach an axenic state.

**MATERIALS**

**Consumables**

- Deionized water
- 0.25x Hoagland 0.1g/L ppm media solution
- Bleach

**Disposables**

- Gloves

**Equipment**

- Inoculating loop
- Bunsen burner
- Culture tubes
- Beakers
- Falcon tube
- Label
- Sharpie Pen

**PREPARATION**

- Your group will need:
  - 1 beaker of H20
  - 1 beaker of Bleach Solution
  - 1 petri dish
  - 2 inoculation loops
  - 1 Bunsen burner
  - 1 Stereoscope
  - 2 beakers (100 mL)
- Wash your hands thoroughly and don gloves.
- Clean your lab bench and prep additional materials.
- Spray ethanol onto the gloves before beginning in order to re-sterilize. Do not perform this step near an open flame. Set ethanol away from flame once completed.

**INITIAL CLEANING (01: To remove most of the microbes from the field)**

1. Pour 100 mL of DI water into the first beaker (A)
2. Pour 100 mL of the Bleach Solution into the second beaker (B)
3. Pour 100 mL of DI water into the third beaker (C)
4. Turn on your bunsen burner and sterilize the loop using the burner.
5. Transfer a large portion of the duckweeds from the falcon tube into the first water beaker (A)
6. Use the loop to stir the duckweed to manually remove any debris or unwanted substances.
7. Sterilize the loop using the burner and isolate one duckweed cluster from the beaker. Make sure the loop is cool or cool it using the water.
8. Place the duckweed into the bleach (Beaker B) for 10-20 seconds using aseptic techniques.
9. Immediately rinse the duckweed in water. Do this by transferring the duckweed from the bleach beaker to the third water beaker (C) using the loop.
10. Transfer the duckweed to a media tube.
11. Vortex briefly.
12. Label the tube with your bench number, the date, and your initials.
13. Repeat steps 12-16 until all duckweeds are transferred from 6 media tubes.
14. Place the rack on the lighted shelf and wait for growth.
15. Clean lab area
16. Dispose of bleach by pouring in the sink and flushing it with lots of running water.
17. Dispose of duckweeds by dumping large portions in the trash and pouring the water in the sink.
