# Supplementary material for: Introducing a Novel Course-Based Undergraduate Research Experience Using Duckweed as a Model System
Source: Integr Org Biol. 2025 Dec 19;8(1):obaf049. doi: 10.1093/iob/obaf049 (PMC12802901; doi:10.1093/iob/obaf049)
Supplement: obaf049_Supplemental_Files [file obaf049_supplemental_files.zip › 07 Supplementary Materials/Supplementary Materials/12_Week02_PROTOCOL_GrowthMeasurementsforStudents.docx]

# Protocol: Root Measuring and Frond Counting

**Introduction**

This protocol describes the process for rot measuring and frond counting involving *Spirodela* *polyrhiza* (Greater Duckweed). It is imperative that you practice sterile techniques during this lab. Be aware of what you and your equipment touch – do not allow pipette tips to contact anything other than what you are transferring. Sterilize your gloves often with 70% EtOH.

**Objective:**

To measure the root lengths and count the fronds of *Spirodela polyrhiza* to understand its growth characteristics.

Materials:

- *Spirodela polyrhiza* specimens
- Petri dishes or small shallow containers
- Distilled water
- Ruler or caliper (for measuring root lengths)
- Stereomicroscope or magnifying glass (optional, for ease of counting fronds)
- Lab notebook and pen for recording observations

Safety Precautions:

1. Wear lab coats and gloves.

2. Wash hands thoroughly after handling the plants.

3. Dispose of any waste material as per the lab's guidelines.

Procedure:

1. Preparation

1. Wash your hands thoroughly and don gloves and a mask.
2. Clean your lab bench and prep additional materials.
3. Spray ethanol onto the gloves before beginning in order to re-sterilize. Do not perform this step near an open flame. Set ethanol away from flame once completed.
4. Each group of students should collect five specimens of *Spirodela polyrhiza* from the instructor.
5. Place each specimen in a Petri dish with water to prevent dehydration.

2. Root Measurement

1. Carefully lift one specimen from the water using forceps.
2. Gently straighten the roots on a ruler and measure their length in millimeters.
3. Record the measurements in your notebook.
4. Repeat the process for all five specimens.

3. Frond Counting

1. Observe each specimen under a stereomicroscope or with a magnifying glass.
2. Count the number of fronds (leaves) on each plant.
3. Record the number of fronds in the lab notebook alongside the root measurements.

4. Data Analysis

1. Calculate the average root length and the average number of fronds per specimen.
2. Discuss any observed variations and potential reasons for these differences.

5. Cleanup

1. Return the specimens to their original containers.
2. Clean and store all equipment.
3. Dispose of any waste material according to lab guidelines.

Conclusion:

Students should write a brief report summarizing their findings, discussing potential factors influencing the growth of Spirodela polyrhiza, and reflecting on the importance of such plants in aquatic ecosystems. Students should also answer the following questions in their notebook along with a drawing of the duckweed plant. This will be checked before leaving class.

Label each term on your drawing: Frond, Root, Cluster,

**Questions:**

Why is it important to bleach/clean the duckweed before using it in experiments?

How do the two microscopes differ with your duckweed image?

What is the importance of burning your inoculation loop between each cluster of duckweed?
