# Supplementary material for: Introducing a Novel Course-Based Undergraduate Research Experience Using Duckweed as a Model System
Source: Integr Org Biol. 2025 Dec 19;8(1):obaf049. doi: 10.1093/iob/obaf049 (PMC12802901; doi:10.1093/iob/obaf049)
Supplement: obaf049_Supplemental_Files [file obaf049_supplemental_files.zip › 07 Supplementary Materials/Supplementary Materials/13_Week03_PROTOCOL_MicrobialPlatingforStudents.docx]

# Protocol: Microbial Plating

## **Introduction**

This protocol describes the process for serial dilution and microbe plating. It is imperative that you practice sterile techniques during this lab. Be aware of what you and your equipment touch – do not allow pipette tips to contact anything other than what you are transferring. Sterilize your gloves often with 70% EtOH.

## **Materials**

| - Deionized water (sterile) | - Water Sample | - Gloves |
| --- | --- | --- |
| - Test tubes (3, sterile) | - Bunsen burner | - Goggles |
| - Culture tubes | - Lab Coat | - Sterile pipette tips |
| - Vortex | - Petri Dishes | - Masks |
| - P1000 pipette | - P10 pipette | - Serological pipette |

## **Procedure**

#### Serial Dilutions

1. Wash your hands thoroughly and put on gloves and a mask.
2. Clean your lab bench and prep additional materials.
3. Spray ethanol onto the gloves before beginning to re-sterilize.
4. Turn on your Bunsen Burner. Do not perform this step near an open flame. Set ethanol away from flame once completed.
5. Using a sterile serological pipette, transfer 4.5 ml of sterile water into three test tubes. Be careful to not allow the pipette tip to become contaminated between transfers. This will be the start of your serial dilutions
6. Using the label tape, label the test tubes with 10 –1, 10 –2, 10 –3,
7. Extract 0.5 ml of water from the Falcon Tube sample.
8. Use the burner to sterilize the neck (or top) of the water tube and test tubes as you open and close the water tube and test tube caps.
9. Add the 0.5 ml to your first test tube of water. This is your -1 dilution. Mix the sample by pipetting the liquid up and down repeatedly 20 –30 times.
10. Next, transfer 0.5 ml of the -1 dilution to a new water tube. This will be your -2 dilution. Mix the sample by pipetting the liquid up and down repeatedly 20 –30 times.
11. Repeat steps until you reach –3 dilutions.

#### Microbial Plating

1. Label petri dishes accordingly: Initials, Date, Bench #, Sample Name, Dilution
   1. EX: JD 01/30/2024 Bench 1, 10 –1

JD 01/30/2024 Bench 1, 10 –2

JD 01/30/2024 Bench 1, 10 –3

JD 01/30/2024 Bench 1, Sample

- 1. Label one petri dish as your control
     1. JD 01/30/2024 Bench 1 Control

1. Using a micropipette and a sterile tip, transfer 10 ul of each dilution tube to the corresponding petri dish. **Beginning with the highest dilution series.**
2. Once all the samples are transferred, use the spreader to spread the sample in the dish, **beginning with the highest dilution series**.
   1. Make sure to sterilize the spreader before and after each use with ethanol located on the bench top.
   2. Be sure to press lightly or else you could puncture the agar.
3. Plate sample directly from the green falcon tube.
4. Plate the Control solution given to you by your instructor.
5. Give your petri dishes to your instructor to incubate.

## **Clean-up**

- Return all items or discard in their proper receptacle. Gloves go in the biohazard bag.
- Sterilize benchtops with EtOH and paper towels.
- Push chairs back under the bench when finished.
