# Supplementary material for: Introducing a Novel Course-Based Undergraduate Research Experience Using Duckweed as a Model System
Source: Integr Org Biol. 2025 Dec 19;8(1):obaf049. doi: 10.1093/iob/obaf049 (PMC12802901; doi:10.1093/iob/obaf049)
Supplement: obaf049_Supplemental_Files [file obaf049_supplemental_files.zip › 07 Supplementary Materials/Supplementary Materials/14_Week03_PROTOCOL_MicrobialPlatingforStudents.docx]

# Protocol: Microbial Plating

## **Introduction**

This protocol describes the process for serial dilution and microbe plating. It is imperative that you practice sterile techniques during this lab. Be cognizant of what you and your equipment touch – do not allow pipette tips to make contact with anything other than what you are transferring. Sterilize your gloves often with 70% EtOH.

## **Materials**

| Deionized water (sterile) | Inoculating loop | Gloves |
| --- | --- | --- |
| Test tubes (6, sterile) | Bunsen burner | Goggles |
| Culture tubes | Glass beads | Lab Coats |
| Vortex | Petri Dishes | Masks |

## **Procedure**

#### Serial Dilutions

1. Wash your hands thoroughly and don gloves and a mask.
2. Clean your lab bench and prep additional materials.
3. Spray ethanol onto the gloves before beginning in order to re-sterilize. Do not perform this step near an open flame. Set ethanol away from flame once completed.
4. Using a sterile serological pipette, transfer 4.5 ml of sterile water into four test tubes. Be careful to not allow the pipette tip to become contaminated between transfers.
5. Extract 0.5 ml of water from the Falcon Tube sample.
6. Turn on the burner and sterilize the water tube as you open and close the water tube caps.
7. Add the 0.5 ml to your first test tube of water. This is your -1 dilution.
8. Next, transfer 0.5 ml of the -1 dilution to a new water tube. This will be your -2 dilution.
9. Repeat step 4 until you reach –5 dilutions.

#### Microbial Plating

1. Label petri dishes accordingly.
   1. EX: JD 01/30/2023 Bench 1 WDRD

JD 01/30/2023 Bench 1 PNLK

JD 01/30/2023 Bench 1 MPSP

1. Add ~10 glass beads to each petri dish
2. Using the p10 micropipette and a sterile tip, transfer 10 ul of each dilution tube to the corresponding petri dish. Beginning with the highest dilution series.
3. Once all the samples are transferred, stack the petri dishes and shift the stacks using the glass beads to spread the sample in the dish.
   1. Usually, ~30 shifts in each direction will suffice.
4. Remove the beads and place them into the discard beaker.
5. Give your petri dishes to your instructor to incubate.

## **Clean-up**

- Return all items or discard in their proper receptacle. Gloves (only) go in the biohazard bag.
- Sterilize benchtops with EtOH and paper towels.
