# Supplementary material for: Introducing a Novel Course-Based Undergraduate Research Experience Using Duckweed as a Model System
Source: Integr Org Biol. 2025 Dec 19;8(1):obaf049. doi: 10.1093/iob/obaf049 (PMC12802901; doi:10.1093/iob/obaf049)
Supplement: obaf049_Supplemental_Files [file obaf049_supplemental_files.zip › 07 Supplementary Materials/Supplementary Materials/15_Week03_THA_MethodsSummaryAssignmentGuide.docx]

# Methods Summary Writing Guide

Begin by watching the Protocol Lectures and reading the Protocol sheet for the week. Make note of the key steps and pieces of information. You should have at least one paragraph (4-6 sentences) and no more than two paragraphs under each subsection (technique). This is to be written in paragraph format and not a bulleted list. Remember it’s a summary. When you begin writing, remember the following rules:

1. Speak in the past tense because methods are written after the experiment is performed. Use third person perspectives as if you are watching yourself perform the experiment. Utilize a passive voice/tone. Detach yourself from your writing. Do not use personal pronouns such as "I" or "we". (e.g. The test tube was capped and inverted to mix contents. Absorbance readings were taken....).
2. Ensure that the information written can allow other scientists to replicate your experiment. Remember that your audience is primarily scientists, and they should know how to perform commonly used techniques such as pipetting, serial dilutions, or basic operation of a spectrophotometer. Focus should be placed on things such as volumes, concentrations, conditions (eg. temperature and growth media composition for organisms), number of replicates, or measuring device and program settings (eg. wavelength value used on the spectrophotometer)
3. Reference primary literature (your TA, yourself and your group members are not referenceable sources). If a technique is thoroughly and effectively outlined in prior literature, very briefly summarize what was done and refer to the paper/papers that outline the protocol.
4. NEVER copy word for word from any source. A reference is NOT a free pass to plagiarize. Always use your own words. A great trick is to rewrite something you read multiple times in various ways and choose the best sentence/sentences.
5. Be concise and optimize your use of space on a page. Remember that you are summarizing all of your methods to cover the important details. Do not provide more information than is necessary. Compress sentence and paragraph structure where possible for the highest clarity and transparency but utilizing the fewest. If you can compress 2 sentences into one extremely clear and concise sentence, then always do so.
6. Use subheadings where necessary. Separate different preparations or phases of the methods when it is applicable. One obvious example is the separation of methods for data acquisition and data analysis. Data acquisition may have various phases that require sub sectioning (e.g. Preparation of diatom samples in growth media and growth conditions, measurement of results using spectrophotometry, etc.)

If you have any questions throughout this process, do not hesitate to ask for help. This style of writing can be very difficult at first but practicing each week will improve your proficiency, which will culminate in your poster preparation. You will also revise your methods summary each week for your lab report submissions.

When your summary is complete, you will submit it to the Turnitin link on Moodle before lab each week. These links will be located under the weekly topic heading they are associated with
