# Supplementary material for: Introducing a Novel Course-Based Undergraduate Research Experience Using Duckweed as a Model System
Source: Integr Org Biol. 2025 Dec 19;8(1):obaf049. doi: 10.1093/iob/obaf049 (PMC12802901; doi:10.1093/iob/obaf049)
Supplement: obaf049_Supplemental_Files [file obaf049_supplemental_files.zip › 07 Supplementary Materials/Supplementary Materials/17_Week03_THA_FindingAndReadingAPaper.docx]

# THA: Finding and Reading a Paper


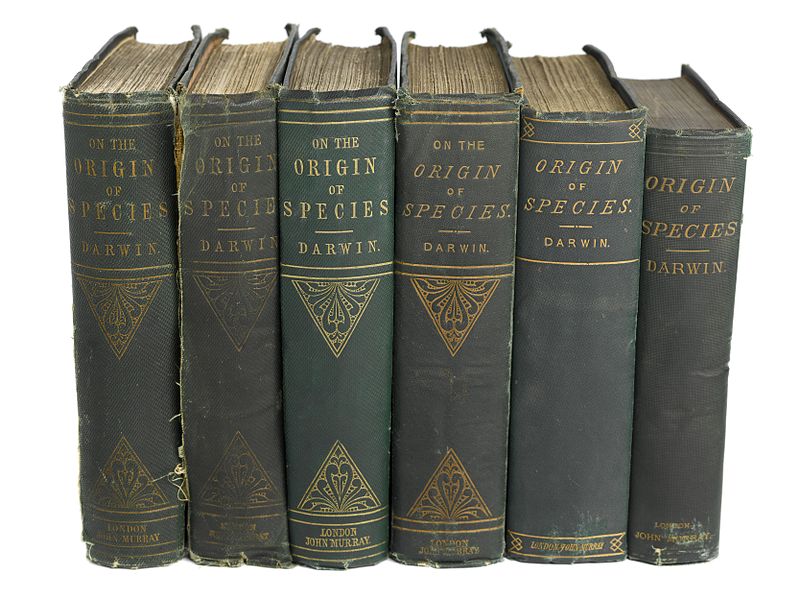
The first step of the scientific method is making observations. We observe things every day that pique our interest and lead us to ask questions. It could be something you see while walking down the street, playing with your pet, eating food, etc. Every day you participate in the first two steps of the scientific method without even realizing it; we are all scientists! The key to good science, however, is going further to seek answers to your questions. Many of you do this already by searching for an answer to your question on the internet or by asking someone who may know. But how do you know the information you find is accurate and credible? You could very well find information that is false or only part of the story.

Whether you plan to study science or simply aspire to know how to find answers to your questions, evaluating what you read is important for everyday life. The skill of finding and evaluating literature must be developed and practiced so you can be knowledgeable about the various topics you discuss with your friends, family, and colleagues.

In this student guide, you will develop a research question about something that piques your interest and work to find background information on that topic. You will utilize familiar search engines, such as Google and Bing, and you will also be exposed to an entirely different way to find more reliable and credible information on the internet. Once you learn how to access more credible scientific information, you will then learn how to read it, so you do not feel overwhelmed or lost.

**Read all instructions throughout the Student Guide so you do not lose points!** To make grading easier, please make sure all your answers are in burgundy.

## I. RESEARCH TOPICS ABOUT YOUR CURE PROJECT

*The first step of researching science literature is to narrow your research topics to be specific to your project. Since we are so early in the semester and you haven’t been introduced to the topics surrounding growing diatoms for biofuels and other bioactivities, you will find a list of ideas below. Choose one of the following topics to begin your search.*

- Plant –Microbe Symbiosis
- Habitat Fragmentation
- Duckweed as an enviornmental model

Chosen topic:

## II. Finding Literature

*When researching a topic, you must determine the keywords that produce the best search results. The best way to do this is to use a basic search engine. In Google, play around with wording to find which keywords are the most effective.*

1. List the best keywords (at least four) that you found on Google.

*Google offers a search engine for finding credible scientific information called Google Scholar. Type “google scholar” into your browser and go to this search engine. Use your best keywords to search for articles within this search engine. To access the full articles within this search engine, you need to be logged into your myLSU account in another browser tab.* Follow the directions below:

- *Sign in to myLSU in your web browser.*
- *Open a new tab and go to* [*Google Scholar*](https://scholar.google.com/)*.*
- *Follow* [*these steps*](https://guides.lib.lsu.edu/c.php?g=923391&p=6655139) *to set your Google Scholar libraries to LSU.*
- *Perform a keyword search.*
- *Click the link to the right of the article to access it via the LSU Library (see image in LSU Library link above).  Most articles will be accessible, but LSU does not have a subscription to every journal.*
- *You can also sometimes click the article title or below the article where it says “All # versions”.*

1. After using the keywords from Google, what other useful keywords did you discover in Google Scholar?

*As you noticed, Google Scholar is a search engine that houses published scientific literature. This type of literature is called* ***primary literature*** *because it is based on data that the authors collected or analyzed themselves. These authors are most often scientists, including research professors, doctors, data scientists, etc. The difference between articles found on Google versus Google Scholar is that primary literature is reviewed heavily by other scientists to make sure the data and article are based on good science.*

## III. Reading Literature

*Now that you have found a piece of primary literature for credibility, you will learn how to read this type of literature.* ***Be sure to choose a scientific paper that you can access the full PDF and that has 3+ figures in the results****.*

*** once you have gotten to this step, show your instructor the paper’s full PDF. Upon their approval, you are free to go and complete the rest at home ***

1. Find the important information to use for a citation by completing the table below.

| Author(s) |  |
| --- | --- |
| Date of publication |  |
| Title of article |  |
| Title of journal |  |
| Volume |  |
| Page number |  |
| Website |  |
| Date found (today’s date) |  |

1. Using the information in the table above, provide the full citation in APA format below. See the APA Format Guide on Moodle.

#### Read the abstract.

1. What is the purpose of this study?
2. What are the major results?
3. What are the keywords that the authors list for this paper? Note: They should be below the abstract.

#### Read the introduction.

1. Notice how the introduction begins with relevant background information that gives the reader important information to understand the study. What did you learn from reading it?
2. Outline the main topics discussed in the background information. Hint: Each paragraph is typically a new topic. Topics begin broad and end narrow.
3. Notice how the introduction ends by being very specific about the study at hand. What are the research questions addressed in this study? What hypotheses and/or predictions are made?
   - RQ:

- H/P:

#### Read the discussion and conclusions.

Check your knowledge and understanding by summarizing the article.

1. What are the main conclusions? (Hint: See abstract and concluding paragraphs.)
2. How does this paper advance scientific knowledge? (Hint: See abstract, introduction, and concluding paragraphs.)
3. What are the real-world applications? How is this study useful? (Hint: See discussion or concluding paragraphs.)
4. What are some limitations of the study? What are some other possible explanations for the findings? (Hint: See discussion or concluding paragraphs.)
5. What questions can be asked for future studies? Include suggestions from both the author(s) and you. (Hint: See discussion or concluding paragraphs.)

#### Find the results section.

The most important parts of the results section are the figures, tables, and images. In scientific writing, figures, along with their captions, should be easy to understand, even when you do not thoroughly read the paragraphs. Choose two figures and one table or image to answer the questions below.

| **First figure:** | |
| --- | --- |
| 1. Title |  |
| 1. What trends (results) are shown in this figure? |  |
| 1. What are the major conclusions (discussion) from this figure? |  |

| **Second figure:** | |
| --- | --- |
| 1. Title |  |
| 1. What trends (results) are shown in this figure? |  |
| 1. What are the major conclusions (discussion) from this figure? |  |

| **Table or image**: (or third figure if no table or image) | |
| --- | --- |
| 1. Title |  |
| 1. What trends (results) are shown in this figure? |  |
| 1. What are the major conclusions (discussion) from this figure? |  |

#### Read the methods.

Focus only on the design of the study and not the statistical analyses.

1. Create an outline of the main methods involved. The point of this exercise is to show you how to take the methods section, which is oftentimes very technical and confusing, and simplify it. Don’t worry if you don’t fully understand the techniques used, the skill you’re developing is being about to simplify and organize.

## Part IV. Annotated bibliography

*Using the information above, begin your annotated bibliography. More than a simple list of references, an annotated bibliography gives a brief description of each paper for future reference and allows you to keep track of relevant papers that you find. You will add two more papers for now.*

- Full reference in APA format:
- Keywords listed on paper (if available):
- Why is this source relevant to our project? (1-2 sentences)
- Describe the main conclusions of this paper (2-3 sentences):
