# Supplementary material for: Introducing a Novel Course-Based Undergraduate Research Experience Using Duckweed as a Model System
Source: Integr Org Biol. 2025 Dec 19;8(1):obaf049. doi: 10.1093/iob/obaf049 (PMC12802901; doi:10.1093/iob/obaf049)
Supplement: obaf049_Supplemental_Files [file obaf049_supplemental_files.zip › 07 Supplementary Materials/Supplementary Materials/18_Week04_RESOURCES_ExperimentalDesign.docx]

**Experimental Design: Duckweed CURE**

For this experiment we will be observing the effect that habitat size has on duckweed-microbe interactions. We are using habitat size as a preliminary effect to habitat fragmentation. We will be using a factorial design in which we change the size of the habitat (the test tube) and the microbial community added. At every bench there are 3 groups.

Each group will test one strain of duckweed (BAPL, PNLK, WDRD) and add all 3 of the microbial communities to their respected test tubes (Pictured below). This will be our first block. Our experiment will contain **3 blocks** for each group, we will also have a block of test tubes that only contain duckweed and a block of test tubes that only contain microbes.

Each week we will pipette some of the duckweed medium into microplates to measure the Optical Density. This allows us to measure the **biomass of the microbial community** within the test tube. We will also count the number of duckweed fronds in each test tube. This allows us to measure the **growth of** **the duckweed**. At the end of the experiment, you should be able to make inferences about habitat size, habitat fragmentation, duckweed growth, and plant-microbe interactions

**Each group will test **one** duckweed genotype with **three** habitat sizes and **three** microbial communities. You will have 3 replicates for each treatment, resulting in 9 test tubes total for each block (27 total test tubes) .


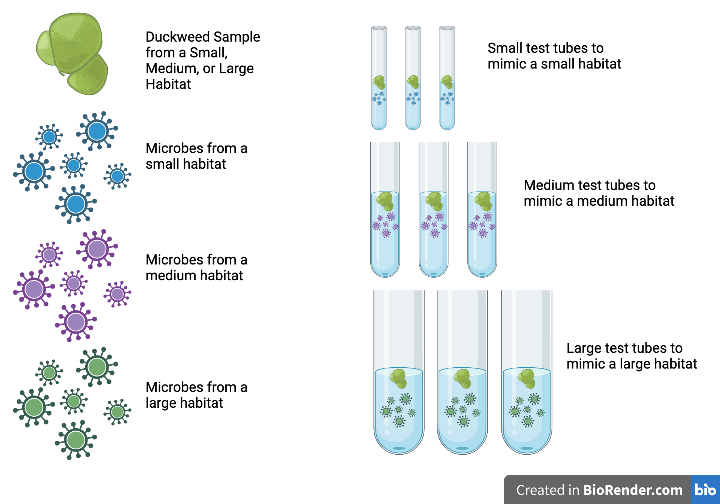


**Figure 1: Treatments Overview for block 1**


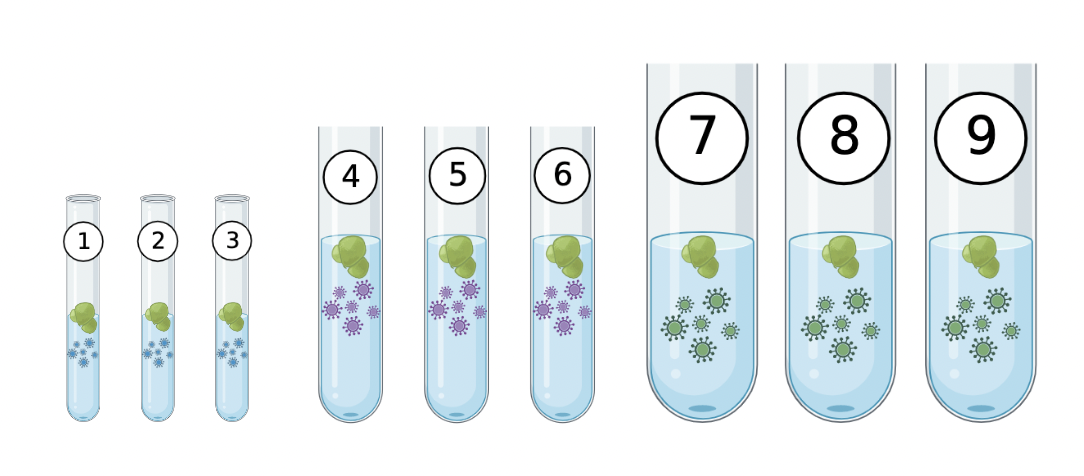


**Figure 2: Block 1 set-up (Duckweed + Microbes)**


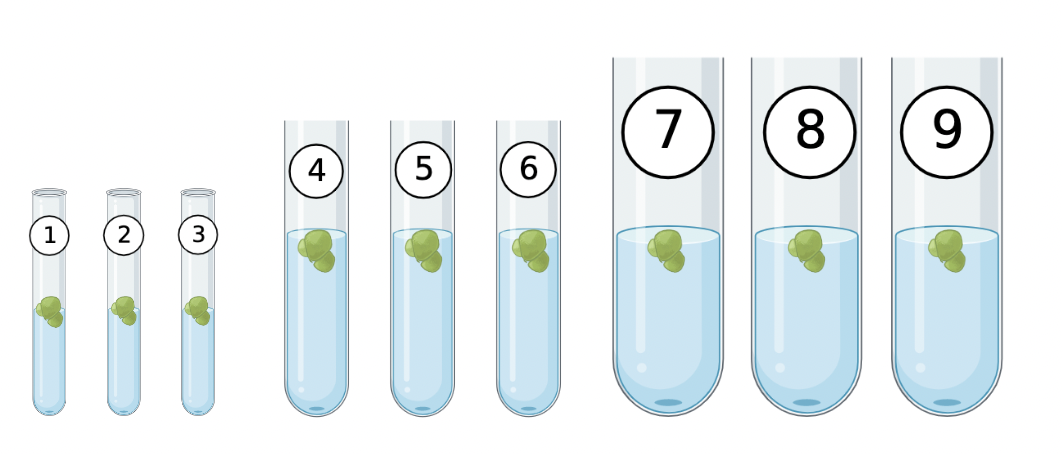


**Figure 3: Block 2 set-up (Duckweed only)**


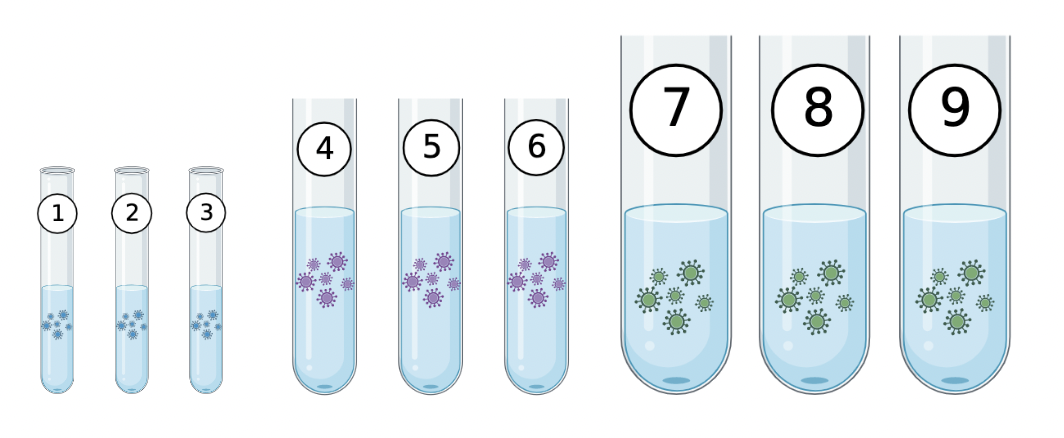


**Figure 4: Block 3 set-up (Microbes only)**
