# Supplementary material for: Introducing a Novel Course-Based Undergraduate Research Experience Using Duckweed as a Model System
Source: Integr Org Biol. 2025 Dec 19;8(1):obaf049. doi: 10.1093/iob/obaf049 (PMC12802901; doi:10.1093/iob/obaf049)

HOW IS TEMPERATURE INFLUENCING TURION GERMINATION?

MAJOR FOCUSES

Climate Change

Climate change encompasses the long-term alterations and shifts in global or regional climate patterns. A significant facet of this change is the phenomenon of "global warming," an observed increase in global temperatures over recent decades. This warming trend is attributed largely to human interventions, especially the emission of greenhouse gases like carbon dioxide (CO_2) and methane (CH_4) from activities such as burning fossil fuels, industrial processes, and deforestation. Once released into the atmosphere, these gases act like a blanket, trapping heat and thereby increasing Earth's average temperature. The consequences of this warming are multifaceted and interconnected: polar ice caps and glaciers melt, resulting in rising sea levels; weather patterns become more unpredictable and extreme; terrestrial and aquatic ecosystems shift, affecting biodiversity and habitats; and ocean temperatures rise, leading to coral bleaching and altered marine life behaviors. This intricate web of changes further amplifies challenges for both terrestrial and aquatic environments, emphasizing the crucial need to address the root causes of climate change.

This Study's Relation to Climate Change:

- Direct Correlation: The study directly correlates with the central issue of climate change - the rising global temperatures and their impact on biological life.
- Predictive Modeling: It contributes to predictive modeling about how ecosystems might change with ongoing climate shifts, which is vital for formulating environmental policies and conservation strategies.

WHY IS THIS IMPORTANT?

1. Understanding Plant Responses to Temperature Variations:

- Physiological Insights: The experiment offers insights into how a specific plant species responds to temperature changes. This understanding is crucial for predicting how plants might adapt or struggle as global temperatures fluctuate.
- Model for Other Species: Spirodela polyrhiza can serve as a model organism. Learning about its response to temperature changes can provide clues about the responses of other aquatic and terrestrial plants.

2. Ecological and Environmental Implications:

- Ecosystem Dynamics: Aquatic plants like Spirodela polyrhiza play vital roles in their ecosystems. Changes in their growth patterns due to temperature variations can have cascading effects on water quality, biodiversity, and the overall health of aquatic ecosystems.
- Indicator of Climate Change Effects: The way these plants respond to temperature changes can act as an indicator of how ecosystems are likely to be affected by climate change.

3. Climate Change Adaptation and Mitigation:

- Adaptation Strategies: Understanding the thermal tolerance and adaptability of plants helps in developing strategies for preserving and managing ecosystems under changing climatic conditions.
- Carbon Sequestration: Plants like Spirodela polyrhiza contribute to carbon sequestration. Understanding how their growth and survival are impacted by temperature can inform predictions about carbon capture potential in changing climates.

4. Educational and Research Value:

- Foundation for Future Research: This study can pave the way for more comprehensive research on plant responses to various aspects of climate change, such as altered precipitation patterns, increased CO2 levels, and more.

SPIRODELA POLYRHIZA

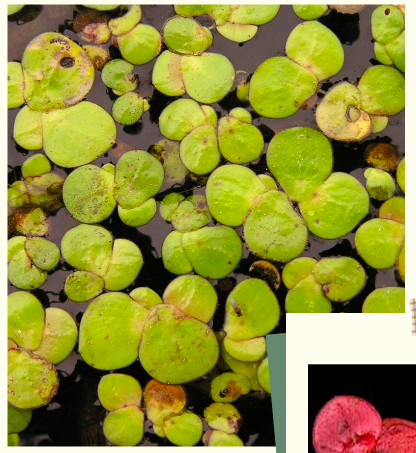

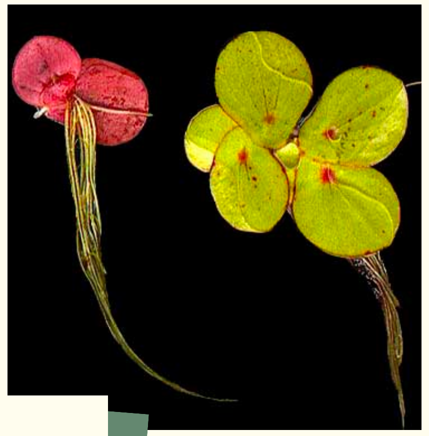

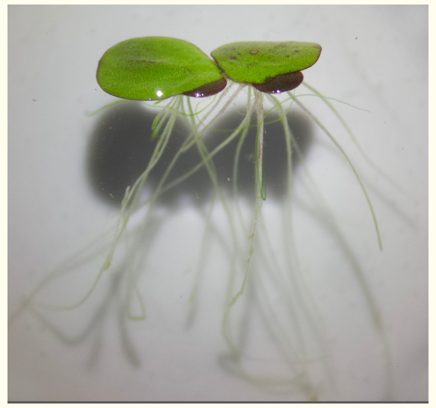

WHAT IS A TURION?

DEFINITION

Turions in Spirodela polyrhiza are specialized, overwintering buds that help the plant survive unfavorable conditions, particularly the cold winter months. They are a form of asexual reproduction.

Turions are dense, compact structures that form in the parent plant. They are typically rich in starch, which aids in their buoyancy and survival during dormant periods.

DORMANCY AND SURVIVAL MECHANISM

These turions detach from the parent plant and sink to the bottom of the water body as temperatures drop. This sinking behavior is a survival strategy that helps them avoid ice formation and damage during the winter. They remain dormant at the bottom throughout the cold season.

As the conditions become favorable in spring, turions germinate and float back to the surface. They then start to grow into new Spirodela polyrhiza plants, thereby continuing the lifecycle.

ECOLOGICAL IMPORTANCE

Turions play a crucial role in the life cycle of Spirodela polyrhiza and are an important adaptation for surviving in temperate climates. Their ability to sink and later rise allows the species to colonize and re-colonize habitats effectively.

CURRENT RESEARCH

Turions are often studied in botanical and ecological research due to their unique adaptation mechanisms and role in plant survival and reproduction.

Schedule Overview

1

Duckweed Prep

The first step in our experiment is to get familiar with duckweed! We will be using turions from a species of duckweed called Spirodela polyrhiza (Greater Duckweed). We will also perform serial dilutions to showcase the difference between axenic (clean) turions and turions in their natural environment! Naturally, duckweed and turions host a wide variety of microbes on their roots! Since these microbes can affect growth and development, we will try to keep our turions as clean as possible!

- Week 1: Pipetting Lesson
- Week 2: Microscopy of Duckweed & Measuring Growth
- Week 3: Microbial Plating
- Week 4: Photo Annotations

2

Experimental Setup

For your experiment, we will grow turions in a well plate. We will have 3 temperature groups: 40 degrees, 30 degrees, 20 degrees!
The 20 degrees group will be our **Control Group**, the 40 and 30 groups will be our **Experimental Groups**.

Independent Variable: Temperature
Dependent Variables: Turion Germination (Growth)

- Week 5: Experimental Setup - measure OD600

3

Data Collection

After our experiment is setup, every week we will count the number of duckweed fronds and take photos to measure coverage, coloration, and more!

- Week 5: Day 0 Data Collection
- Week 6, 8, 10: Data Collection
- Week 10: Experiment Breakdown and final data collection - measure OD600

KEY WORDS TO KNOW

- Climate Change
- Ecosystem
- Temparature
- Turion
- Adaptation
- Duckweed
- Germination
- Frond

LAB TECHNIQUES

- Microscopy
- Bunsen Burner usage
- Duckweed Bleaching
- Incubation
- Plant Growth Chambers
- Pipetting
- Photographic Documentation
- Sterile Techniques
- Monitoring Conditions
- Innoculating

EXPERIMENT OVERVIEW

We number each individual test tube to make data collection easier! If you know the number of the test tube, you can create a chart to record the duckweed fronds and microbial abundance for that specific test tube each week.

Did you know? You can number test tubes directly on the glass with sharpie marker! The sharpie doesn't stain the glass, and can be cleaned with alcohol. This is the best way to number replicates!

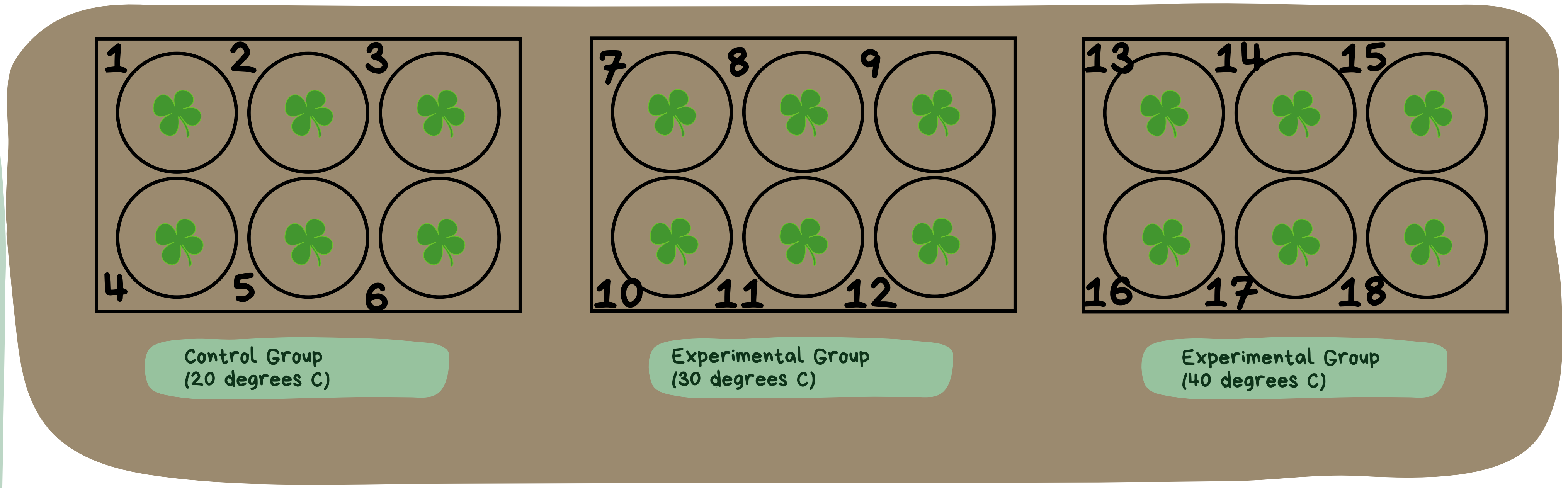

We will start by placing one turion in each well!

Know the difference between independent (the one you change), dependent (the one you measure), and controlled variables (the ones you keep constant)!

Weekly assessments will involve counting duckweed fronds and taking photographic documentations!

THIS EXPERIMENT HAS 3 TEMPERATURE GROUPS, WITH 6 REPLICATES, WHICH MEANS YOUR SAMPLE SIZE IS 18. 3 (GROUPS) X 6 (REPLICATES) = 18 SAMPLES

WEEK 1: PIPETTING BY DESIGN

This week we will be practicing our pipet skills by using a pipetting by design lab! This is just like a paint-by-numbers... but for scientists! If you follow the directions you'll create a something cool!

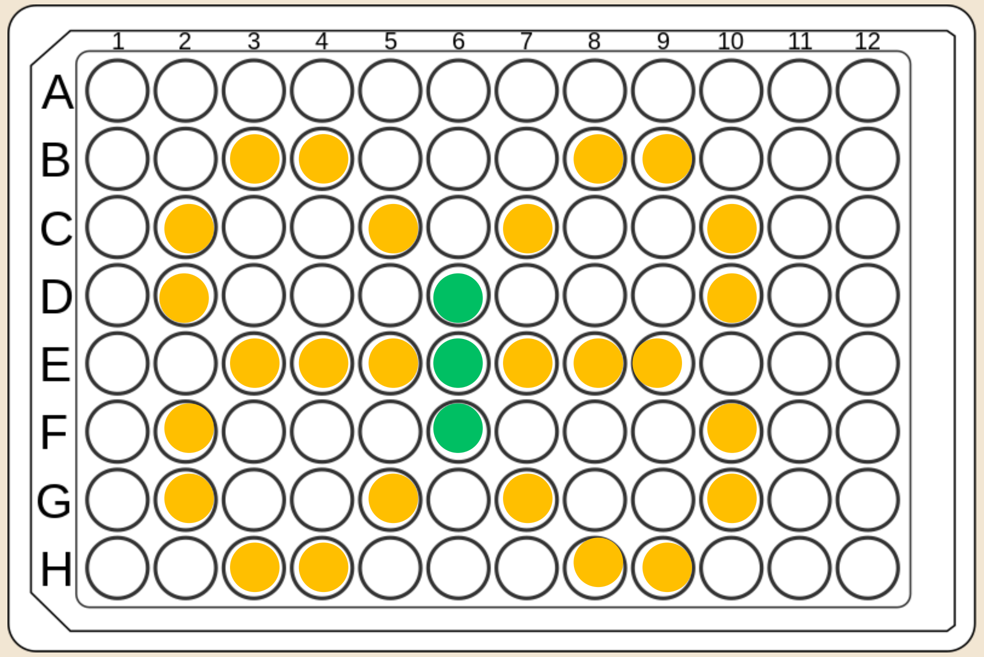

WEEK 2: MICROSCOPY & GROWTH MEASUREMENTS

Measuring root length and counting fronds in Spirodela polyrhiza helps in understanding its growth patterns and overall health, which is crucial for ecological studies and understanding plant biology.

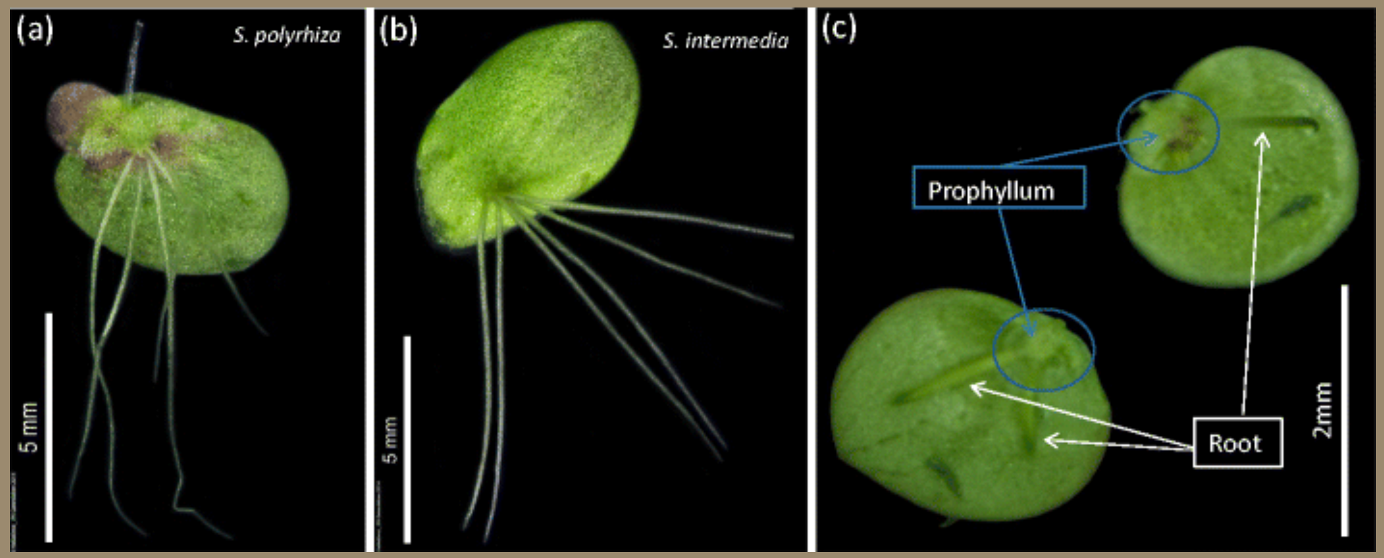

WEEK 3: PLATING MICROBES

Micorbes are an important part of the duckweed life cycle! However, they make things complicated for lab experiments. therefore, whenever we are doing research with duckweed, we like to use <u>axenic</u> duckweed. This means there are no microbes on the duckweed plants! We will plate a pond water sample and a sterile sample to show the difference between the two!

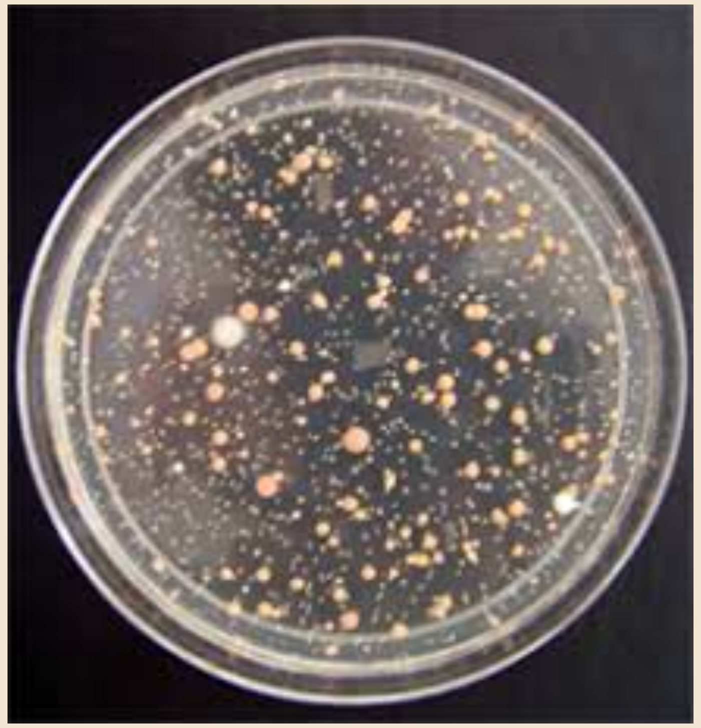

WEEK 4: PHOTO ANNOTATION

Taking photos is a great way to keep up with the growth process! We will practce with an online annotation tool to get you familar with measuring duckweed coverage and counting fronds!

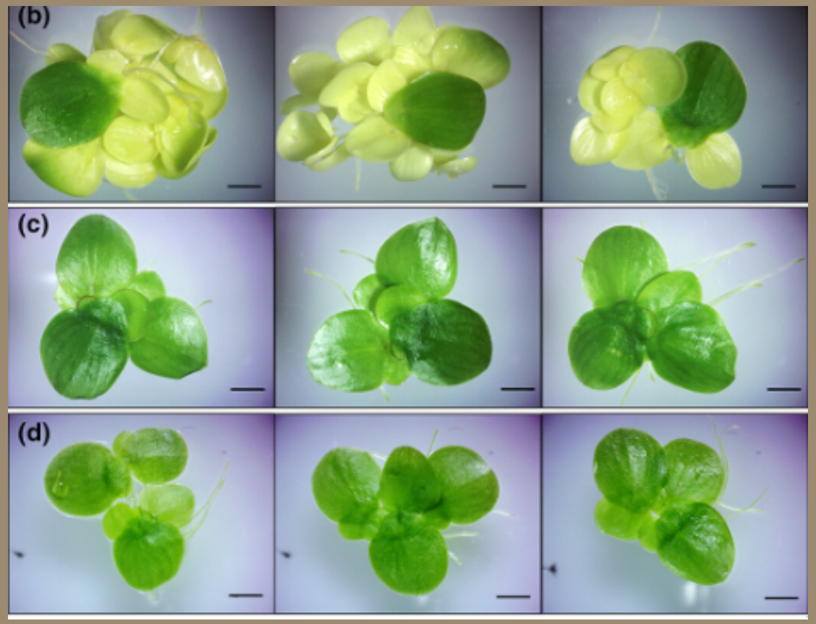

WEEK 5: EXPERIMENT SETUP

Keeping everything clean and germ-free during our experiment is super important. We don't want any stray bacteria from our hands or breath messing up our results. So, we use Bunsen burners, which are basically small gas-powered fires, to kill off any unwanted germs on our tools and test tubes. We also wear gloves and masks for extra protection. Gloves stop any bacteria on our hands from getting into our experiment, and masks catch any tiny droplets that might come out of our mouths when we talk or breathe. All this ensures we only study the microbes we want to and nothing else sneaks in to skew our results.

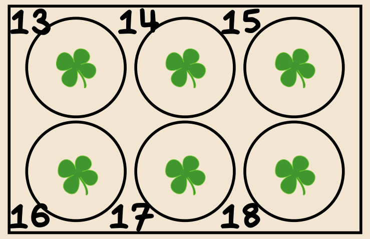

Experimental Group (40 degrees C)

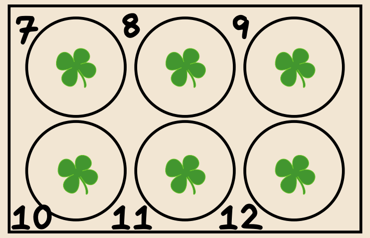

Experimental Group (30 degrees C)

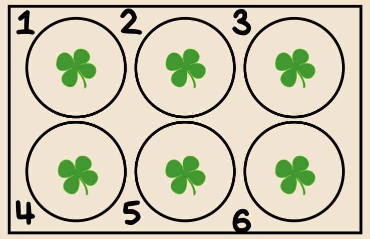

Control Group (20 degrees C)

WEEK 6 - 10 DATA COLLECTION

Throughout our experiment, we'll be collecting a bunch of data. To keep track of all this information, we'll be using Excel, a computer program that's great for organizing numbers and data into easy-to-read tables. Once we've recorded everything in Excel, we'll then move our data over to another program called JMP. JMP is a powerful tool that can sift through all our numbers and help us see if there's any patterns or interesting connections between the things we've been measuring. So, in short, Excel helps us collect our data in an organized manner, while JMP allows us to analyze and make sense of that data once the experiment concludes.

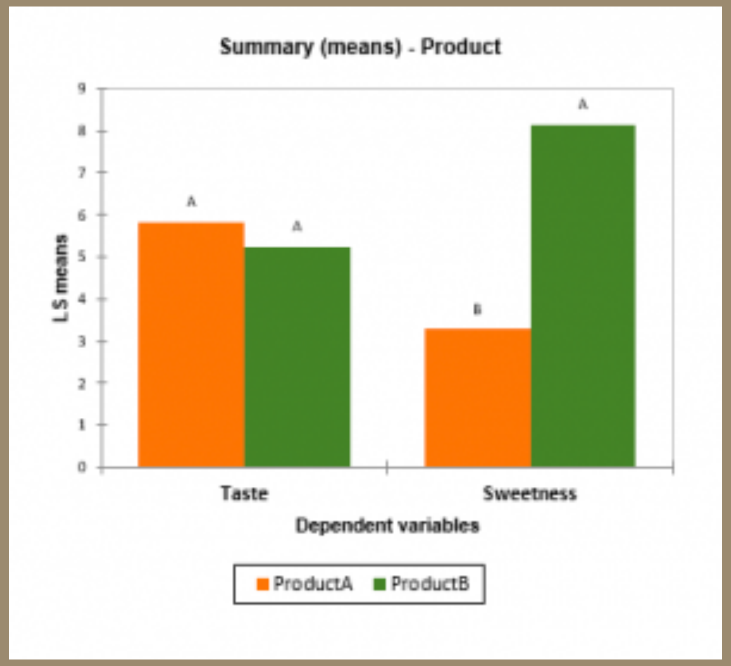

Supplement: obaf049_Supplemental_Files [file obaf049_supplemental_files.zip › 07 Supplementary Materials/Supplementary Materials/19_Week04_RESOURCES_ExperimentalDesignOverview.pdf]
