# Supplementary material for: Introducing a Novel Course-Based Undergraduate Research Experience Using Duckweed as a Model System
Source: Integr Org Biol. 2025 Dec 19;8(1):obaf049. doi: 10.1093/iob/obaf049 (PMC12802901; doi:10.1093/iob/obaf049)
Supplement: obaf049_Supplemental_Files [file obaf049_supplemental_files.zip › 07 Supplementary Materials/Supplementary Materials/20_Week04_THA_ExperimentOverviewQuestions.docx]

Turions, Experiment Overview Questions

BIOL 1503

Spring 2024

**Please use the Experiment Overview PDF, located on Moodle, to answer the following questions about turions and the experiment we plan to run throughout this course. Feel free to use other sources to find information, but please cite them at the end of each question. Please answer the questions in blue.**

1. What is Spirodela polyrhiza?
2. What are turions?
3. Why is it important to maintain a clean and germ-free environment during your experiment?​​
4. What software tools are used for data collection and analysis in the experiment? There are two.
5. What are the independent and dependent variables in the Spirodela polyrhiza experiment, and how are they related to the study of temperature effects on turion germination?​
6. Why is measuring root length and counting fronds important in understanding the growth patterns and overall health of duckweed?​​
7. Explain the difference between axenic (clean) turions and turions in their natural environment in the context of Spirodela polyrhiza growth.​
8. What are the independent and dependent variables in this experiment, and how are they related to the study of temperature effects on turion germination?​​.
9. Discuss the ecological importance of turions in the life cycle of Spirodela polyrhiza and their role in surviving temperate climates.​​
10. How does temperature influence turion germination in Spirodela polyrhiza, and what implications does this have for understanding climate change impacts?
