# Supplementary material for: Introducing a Novel Course-Based Undergraduate Research Experience Using Duckweed as a Model System
Source: Integr Org Biol. 2025 Dec 19;8(1):obaf049. doi: 10.1093/iob/obaf049 (PMC12802901; doi:10.1093/iob/obaf049)
Supplement: obaf049_Supplemental_Files [file obaf049_supplemental_files.zip › 07 Supplementary Materials/Supplementary Materials/22_Week04_PROTOCOL_StreakingMicrobialColonies.docx]

# Protocol: Microbial Streaking

## **Introduction**

This protocol describes the process for serial dilution and microbe plating. It is imperative that you practice sterile techniques during this lab. Be cognizant of what you and your equipment touch – do not allow pipette tips to make contact with anything other than what you are transferring. Sterilize your gloves often with 70% EtOH.

## **Materials**

| - Gloves - Goggles - Lab Coats - Masks | - Petri Dishes - Inoculating loop - Bunsen burner - Ethanol | - Gloves |
| --- | --- | --- |
|  |  | - Goggles |
| - Petri Dishes | - Inoculating loop | - Lab Coats |
|  | - Bunsen burner | - Masks |

## **Procedure**

1. Obtain an
2. Label the bottom of the plate with the color of the bacterial colony, your initials, bench #, and the date.
   1. EX: Yellow, JD, Bench 2, 02/20/2023
3. Sterilize your lab bench by spraying it down with 70% ethanol and wiping it down with a paper towel. Maintain sterility by working near a flame or bunsen burner.
4. Obtain the appropriate bacterial colony (choose one color)
5. Using a sterile loop touch the bacteria growing within the agar plate. (Only touch ONE COLOR at a time)
   1. Sterilize the loop by passing it through a flame, just be sure to allow enough time for the loop to cool before touching it to the bacteria.
6. Gently spread the bacteria over a section of the plate, as shown in the diagram above, to create streak #1.
   1. Hold your loop at an angle, the way you would hold a pencil, so that you can make a broad stroke. Only touch the surface of the plate, do NOT dig into the agar.
7. Using a freshly sterilized loop, drag through streak #1 and spread the bacteria over a second section of the plate, to create streak #2.
8. Using a sterilized loop, drag through streak #2 and spread the bacteria over the last section of the plate, to create streak #3.
9. Incubate plate with newly plated bacteria.

## **Clean-up**

- Return all items or discard in their proper receptacle. Gloves (only) go in the biohazard bag.
- Sterilize benchtops with EtOH and paper towels.
- Wash your hands well.
