# Supplementary material for: Introducing a Novel Course-Based Undergraduate Research Experience Using Duckweed as a Model System
Source: Integr Org Biol. 2025 Dec 19;8(1):obaf049. doi: 10.1093/iob/obaf049 (PMC12802901; doi:10.1093/iob/obaf049)
Supplement: obaf049_Supplemental_Files [file obaf049_supplemental_files.zip › 07 Supplementary Materials/Supplementary Materials/24_Week04_THA_KeyPapersStudentGuide.docx]

# **THA: Key Papers Student Guide**

*For this week’s lab, you will read through three papers that are key to your study of growing diatoms for biofuels. The purpose of this exercise is to provide you with valuable information that will help as you begin to write your paper. PDFs of all three papers can be found on Moodle. You can also use Google Scholar to locate them.*

Read all instructions so you do not lose points! Please make sure all answers are in blue.

**Flores-Renteria et al 2015**

**“Habitat Fragmentation can Modulate Drought Effects on the Plant-soil-microbial System in Mediterranean Holm Oak (Quercus ilex) Forests”**

*Use the guide below to read the paper.*

1. Find the important information to use for a citation by completing the table below.

| Author(s) |  |
| --- | --- |
| Date of publication |  |
| Title of article |  |
| Title of journal |  |
| Volume |  |
| Page number |  |
| Website |  |
| Date found (today’s date) |  |

1. Using the information in the table above, provide the full citation in APA format below. See the APA Format Guide on Moodle.

Read the abstract.

1. What is the purpose of this study?
2. What are the major results?
3. What are the keywords that the authors list for this paper? Note: They should be below the abstract.

Read the introduction.

1. Notice how the introduction begins with relevant background information that gives the reader important information to understand the study. What did you learn from reading it?
2. Outline the main topics discussed in the background information. Hint: Each paragraph is typically a new topic. Notice how topics begin broad and end narrow.
3. Notice how the introduction ends by being very specific about the study at hand. What are the research questions addressed in this study? What hypotheses and/or predictions are made? Not all papers have hypotheses, fyi.
   - Research Question:

- Hypotheses:

Find the results section.

*The most important parts of the results section are the figures, tables, and images. In scientific writing, figures, along with their captions, should be easy to understand, even when you do not thoroughly read the paragraphs.*

| **Figure 1** | |
| --- | --- |
| 1. Title |  |
| 1. What trends (results) are shown in this figure? |  |
| 1. What are the major conclusions (discussion) from this figure? |  |

| **Figure 2** | |
| --- | --- |
| 1. Title |  |
| 1. What trends (results) are shown in this figure? |  |
| 1. What are the major conclusions (discussion) from this figure? |  |

| **Figure 3** | |
| --- | --- |
| 1. Title |  |
| 1. What trends (results) are shown in this figure? |  |
| 1. What are the major conclusions (discussion) from this figure? |  |

## **Kiesewetter et al 2021**

## **“Microbiome-mediated effects of habitat fragmentation on native plant performance”**

*You can skim the* ***Introduction*** *and* ***Conclusion*** *of this paper to complete the questions below.*

1. Find the important information to use for a citation by completing the table below.

| Author(s) |  |
| --- | --- |
| Date of publication |  |
| Title of article |  |
| Title of journal |  |
| Volume |  |
| Page number |  |
| Website |  |
| Date found (today’s date) |  |

1. Using the information in the table above, provide the full citation in APA format below. See the APA Format Guide on Moodle.

Key Takeaways

1. What is habitat fragmentation and what negative effects does it cause?
2. Why is it important to study the effect of habitat fragmentation on plant-microbe interactions?
3. How do these microbial interactions influence the ecosystem?
4. How can we help mediate the negative effects of habitat fragmentation?
5. What future research should be done to understand the effects of habitat fragmentation?

**Obrien et al. 2020**

**“Mutualistic Outcomes Across Plant Populations, Microbes, and Environments in the Duckweed Lemna minor”**

1. Find the important information to use for a citation by completing the table below.

| Author(s) |  |
| --- | --- |
| Date of publication |  |
| Title of article |  |
| Title of journal |  |
| Volume |  |
| Page number |  |
| Website |  |
| Date found (today’s date) |  |

1. Using the information in the table above, provide the full citation in APA format below. See the APA Format Guide on Moodle.

Read the abstract.

1. What is the purpose of this study?
2. What are the major results?
3. What are the keywords that the authors list for this paper? Note: They should be below the abstract.

Read the introduction.

1. Notice how the introduction begins with relevant background information that gives the reader important information to understand the study. What did you learn from reading it?
2. Outline the main topics discussed in the background information. Hint: Each paragraph is typically a new topic. Notice how topics begin broad and end narrow.
3. Notice how the introduction ends by being very specific about the study at hand. What are the research questions addressed in this study? What hypotheses and/or predictions are made?
   - RQ:

- H/P:

Find the results section.

*The most important parts of the results section are the figures and tables. In scientific writing, figures, along with their captions, should be easy to understand, even when you do not thoroughly read the paragraphs.*

| **Figure 1** | |
| --- | --- |
| 1. Title |  |
| 1. What trends (results) are shown in this figure? |  |
| 1. What are the major conclusions (discussion) from this figure? |  |

| **Figure 2** | |
| --- | --- |
| 1. Title |  |
| 1. What trends (results) are shown in this figure? |  |
| 1. What are the major conclusions (discussion) from this figure? |  |

| **Figure 3** | |
| --- | --- |
| 1. Title |  |
| 1. What trends (results) are shown in this figure? |  |
| 1. What are the major conclusions (discussion) from this figure? |  |
| **Figure 4** | |
| 1. Title |  |
| 1. What trends (results) are shown in this figure? |  |
| 1. What are the major conclusions (discussion) from this figure? |  |

Experimental section

Briefly outline the methods described in this paper that we are using this semester: Bleaching Duckweed, Microbial Swaps, Structure the outline as needed for your reference later.

Key takeaways

1. What are some of the positve effects microbes have on duckweed?
2. Why is duckweed being used as a study system?
3. How are the methods in this study similar to the methods in your study?
4. Define Diffuse Mutualisms:

Part II. Annotated bibliography

*Using the information above, begin your annotated bibliography. More than a simple list of references, an annotated bibliography gives a brief description of each paper for future reference and allows you to keep track of relevant papers that you find. The key papers will be part of this, but you will add two more papers for now. These papers can be those that you found for the previous assignments if relevant.*

*Flores-Renteria et al 2015*

- Full reference in APA format:
- Keywords listed on paper (if available):
- Why is this source relevant to our project? (1-2 sentences)
- Describe the main conclusions of this paper (2-3 sentences):

Kiesewetter et al. 2021

- Full reference in APA format:
- Keywords listed on paper (if available):
- Why is this source relevant to our project? (1-2 sentences)
- Describe the main conclusions of this paper (2-3 sentences):

Obrien et al. 2020

- Full reference in APA format:
- Keywords listed on paper (if available):
- Why is this source relevant to our project? (1-2 sentences)
- Describe the main conclusions of this paper (2-3 sentences):

{New paper 1} *replace this line with the in-text citation as shown with the paper you found last week*

- Full reference in APA format:
- Keywords listed on paper (if available):
- Why is this source relevant to our project? (1-2 sentences)
- Describe the main conclusions of this paper (2-3 sentences):
