# Supplementary material for: Introducing a Novel Course-Based Undergraduate Research Experience Using Duckweed as a Model System
Source: Integr Org Biol. 2025 Dec 19;8(1):obaf049. doi: 10.1093/iob/obaf049 (PMC12802901; doi:10.1093/iob/obaf049)
Supplement: obaf049_Supplemental_Files [file obaf049_supplemental_files.zip › 07 Supplementary Materials/Supplementary Materials/25_Week05_PROTOCOL_DataCollectionDay0.docx]

# Protocol: Data Collection Day 0

## **Introduction**

This protocol describes the process for setting up the CURE Duckweed experiment to observe the effects of habitat fragmentation on plant-microbe symbiosis. It is imperative that you practice sterile techniques during this lab. Be cognizant of what you and your equipment touch – do not allow pipette tips to make contact with anything other than what you are transferring. Sterilize your gloves often with 70% EtOH.

## **Materials**

| - Gloves - Goggles - Lab Coats - Masks - Sterile Microplate | - Bunsen Burner - Ethanol - Sharpie - Notebook - Pen | - 13 mm test tubes with Hoagland's media (9) - 16 mm test tubes with Hoagland's media (9) - 25 mm test tubes with Hoagland's media (9) - Test tube racks for each test tube size - Axenic Duckweed |
| --- | --- | --- |

## **PROCEDURE**

## **Treatment: Duckweed + Microbes**

**Duckweed Inoculation**

1. Wash your hands thoroughly and don gloves and a mask.
2. Clean your lab bench and prep additional materials.
3. Spray ethanol onto the gloves before beginning in order to re-sterilize. Do not perform this step near an open flame. Set ethanol away from flame once completed.
4. You will need to coordinate with your bench for the remainder of the protocol
5. Each group will receive nine test tubes (three each: 13 mm, 16 mm, 25 mm)
6. Withing your benchtop, decide which group will begin with each size test tube.
7. Once you have chosen your beginning size, you will need to pick up the correct size test tube rack and the sterile test tubes containing Hoagland’s media.
8. You will use the axenic duckweed given to you by your instructor, to inoculate one cluster of duckweed into each of the 3 test tubes.
9. Repeat the **Duckweed Inoculation** process for all 3 sizes until you have 9 test tubes, 3 of each size containing one cluster of duckweed.
10. Make sure to flame the opening of the test tubes after removing the lid and before replacing the lid.
11. Label each test tube 1-9 as show below:


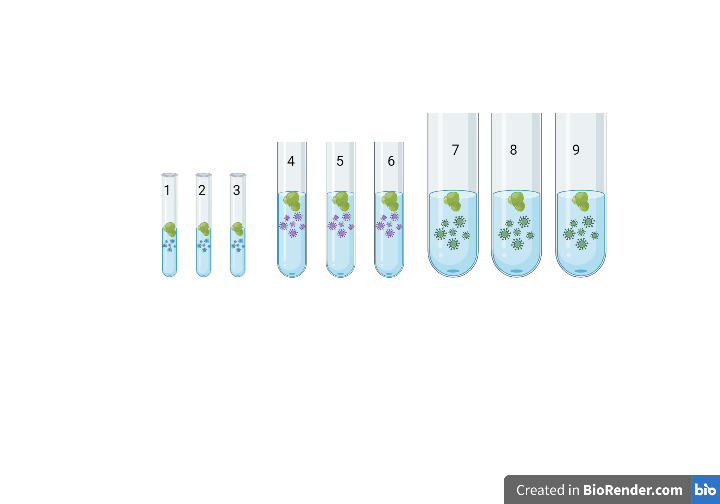


1. Place these test tubes on the rack. We will add the microbes **next week**.

## **Treatment: Duckweed only**

**Duckweed Inoculation**

1. Wash your hands thoroughly and don gloves and a mask.
2. Clean your lab bench and prep additional materials.
3. Spray ethanol onto the gloves before beginning in order to re-sterilize. Do not perform this step near an open flame. Set ethanol away from flame once completed.
4. You will need to coordinate with your bench for the remainder of the protocol
5. Each group will receive nine test tubes (three each: 13 mm, 16 mm, 25 mm)
6. Withing your benchtop, decide which group will begin with each size test tube.
7. Once you have chosen your beginning size, you will need to pick up the correct size test tube rack and the sterile test tubes containing Hoagland’s media.
8. You will use the axenic duckweed given to you by your instructor, to inoculate one cluster of duckweed into each of the 3 test tubes.
9. Repeat the **Duckweed Inoculation** process for all 3 sizes until you have 9 test tubes, 3 of each size containing one cluster of duckweed.
10. Label each test tube 10-18 as show below:


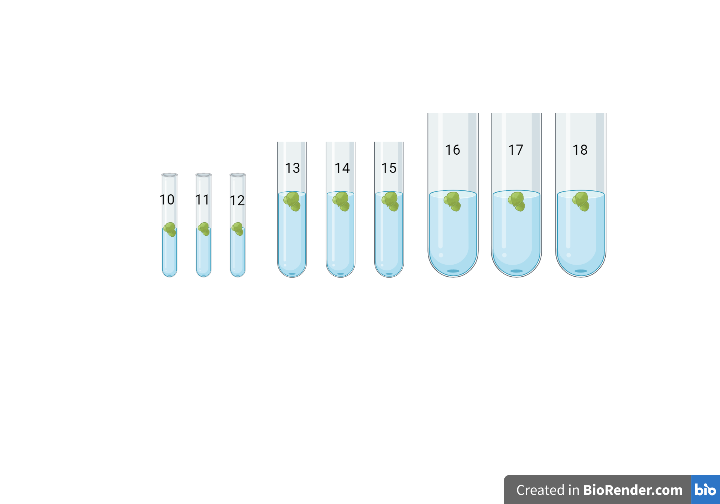


1. Place these test tubes on the rack. They will not be receiving any microbes.

## **PROCEDURE**

## **Treatment: Microbes Only**

**Duckweed Inoculation**

1. Wash your hands thoroughly and don gloves and a mask.
2. Clean your lab bench and prep additional materials.
3. Spray ethanol onto the gloves before beginning in order to re-sterilize. Do not perform this step near an open flame. Set ethanol away from flame once completed.
4. You will need to coordinate with your bench for the remainder of the protocol
5. Each group will receive nine test tubes (three each: 13 mm, 16 mm, 25 mm)
6. Withing your benchtop, decide which group will begin with each size test tube.
7. Once you have chosen your beginning size, you will need to pick up the correct size test tube rack and the sterile test tubes containing Hoagland’s media.
8. Label each test tube 19-27 as show below:


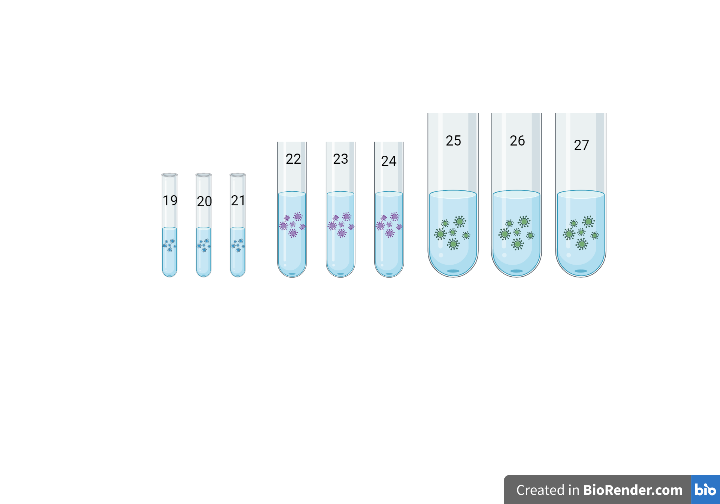


1. Place these test tubes on the rack. We will add the microbes **next week**.

**Data Collection**

1. Once you have finished setting up all 27 test tubes, you will need to sterilize your benchtop
2. To begin your data collection, you will need to count the number of duckweed fronds in each test tube.
3. You can use the excel template uploaded to Moodle to record your data.

**Clean-up**

- Return all items or discard in their proper receptacle. Gloves (only) go in the biohazard bag.
- Sterilize benchtops with EtOH and paper towels.
- Wash your hands well.
