# Supplementary material for: Introducing a Novel Course-Based Undergraduate Research Experience Using Duckweed as a Model System
Source: Integr Org Biol. 2025 Dec 19;8(1):obaf049. doi: 10.1093/iob/obaf049 (PMC12802901; doi:10.1093/iob/obaf049)
Supplement: obaf049_Supplemental_Files [file obaf049_supplemental_files.zip › 07 Supplementary Materials/Supplementary Materials/26_Week05_PROTOCOL_InoculationAndDataCollectionDay0.docx]

# Protocol: Turion Inoculation & Data Collection Day 0

## **Introduction**

This protocol describes the process for setting up the CURE Duckweed experiment to observe the effects of habitat fragmentation on plant-microbe symbiosis. It is imperative that you practice sterile techniques during this lab. Be aware of what you and your equipment touch – do not allow pipette tips to contact anything other than what you are transferring. Sterilize your gloves often with 70% EtOH.

## **Materials**

| - PPE: gloves, goggles, coat, mask - 3 - Sterile 6-Well Microplate - Bunsen Burner - Ethanol | - Sharpie - 1 Turion Pod - Inoculation Loop - Kim Wipes | - Phone to take photo - Laptop - 200 mL of plant growth Medium |
| --- | --- | --- |

## **Procedure:** *Turion Inoculation*

1. Wash your hands thoroughly and don gloves, goggles, a lab coat, and a mask.

#### *Experimental Setup*

1. Clean your lab bench and prep additional materials.
2. Spray ethanol onto the gloves before beginning to re-sterilize. Do not perform this step near an open flame. Set ethanol away from flame once completed.
3. Ask your instructor to turn on your Bunsen burner.
4. You will need 3 sterile microplates. These will be at the top of your bench and sealed to maintain sterility.
5. You will need to use a serological pipette to add **10 mL** of sterile plant medium into each well. You will have 18 wells.
   1. Keep the well plate top on when you are not pipetting to maintain sterility
6. Once the media is added, you may begin adding your turions into the well plates. To do this, you will need to use the inoculation loop.
   1. Sterilize the loop in the flame before and after touching the turions. Be sure to let the loop cool down before touching the turions to avoid scorching them.
7. Use the inoculation loop to add one turions into each well. You will need 18 turions total. Be careful to only add one.
   1. Keep the top on the well plate when you are not inoculating turions to maintain sterility

## *Data Collection*

1. Once you have finished setting up your experiment, sterilize your benchtop
2. To begin your data collection, take a photo of each well within the well plate. There are 3 well plates and 3 groups members, so everyone should record data for one plate.
3. Using ImageJ, count the number of fronds within each well.
4. Using ImageJ, measure the duckweed area for each well.
5. Use the excel template on Moodle to record your data.

## *Clean-up*

- Return all items or discard in their proper receptacle. Gloves (only) go in the biohazard bag.
- Sterilize benchtops with EtOH and paper towels.
- Wash your hands.
