# Supplementary material for: Introducing a Novel Course-Based Undergraduate Research Experience Using Duckweed as a Model System
Source: Integr Org Biol. 2025 Dec 19;8(1):obaf049. doi: 10.1093/iob/obaf049 (PMC12802901; doi:10.1093/iob/obaf049)
Supplement: obaf049_Supplemental_Files [file obaf049_supplemental_files.zip › 07 Supplementary Materials/Supplementary Materials/27_Week05_ICA_IntroductionOutline.docx]

# ICA: Outline an Introduction

## Part I. Introduction Outline

The introduction section of the paper that you will begin writing soon is one of the more challenging sections to develop. This section should introduce the biological topics that surround your study, while also pointing out the gaps in current literature and working to establish the importance of your study. The introduction begins very broad and ends narrow by stating your specific research questions and hypotheses.

While your written introduction section will start with the broadest topic and narrow at the end, many find it easiest to work backwards during the development stage. You can use concept mapping or basic outlining to pick apart what topics should be introduced in your study, as defined by your research questions.

## Study Specifics

1. Main research topic and why important to organism and beyond. This may go in 2^nd^ to last paragraph.

- Research topic:
- Importance of organism:
- Importance beyond organism (real world applications):

1. Null and alternative hypotheses. Make sure to include IV and DV in each. If you have multiple DVs, select the most important one. You will include mention of more DVs measured in other sections of paper.

- Research question:
- Null Hypothesis:
- Alternative Hypothesis:

1. Prediction. Recall that these are written as statements with direction (increase/decrease, faster/slower) and reasoning (support from literature with in-text citation).

- Prediction:
- Reasoning:

The prompts above will help you develop the **last two paragraphs** of your introduction. If there is more than one research question, you can separate your paragraphs based on the two studies.

## Introduction Outline

Organize what you completed above for the final paragraphs and fill in information to complete the paragraphs. Include in-text citations so that, later, you can easily locate where the information came from. You can use the papers that your group located last week and also find additional. Add as many bullet points as needed.

1. Paragraph 1 (broadest topic): *Give an overview of the demand for energy consumption and challenges, especially relating to sustainability and safety of fossil fuel i.e., drawbacks. What are some alternative solutions? (should include biofuels).* *In-text citations.*
2. Paragraph 2 (less broad): *Expand on biofuels. What is the importance of biofuels over fossil fuels? Include several examples. Introduce your model organism and why the organism is of interest as it relates to biofuels. Include other uses of microalgae.*
3. Paragraph 3 (more focused/narrow): *What is your research topic and why is it important to your organism in its natural environment and beyond? How are others attempting to address the problem involving your topic? Highlight drawbacks/limitations to the effective use as biofuels. What makes diatoms problematic for biofuel production?*
4. Paragraph 4 (more focused/narrow): *What are solutions to solving the problem mentioned in the paragraph above? What are some research gaps in the papers you reviewed? Include why the research is important.*
5. Paragraph 5 (your study in the classroom): *How will your research address the research gaps? What are your research questions?* State your hypothesis (use the term, “statistically significant,” or “significant” to show how you want the relationship between independent and dependent variables to be appreciated. State your prediction(s) based off your observation of previous related studies (so include citations). Keep in mind: Prediction gives direction to your study, unlike hypothesis which does not. Also, prediction must be based off your observation of previous related studies (so include citations)

Paragraph 5 still confusing

Need greater focus on the “reasoning” for predictions – past research in terms of results

***Once complete, allow your instructor to review your introduction outline and offer real-time feedback. Be sure to incorporate the feedback into your Formal Writing Assignment.***
