# Supplementary material for: Introducing a Novel Course-Based Undergraduate Research Experience Using Duckweed as a Model System
Source: Integr Org Biol. 2025 Dec 19;8(1):obaf049. doi: 10.1093/iob/obaf049 (PMC12802901; doi:10.1093/iob/obaf049)
Supplement: obaf049_Supplemental_Files [file obaf049_supplemental_files.zip › 07 Supplementary Materials/Supplementary Materials/28_Week05_RESOURCES_FWAWritingGuide.docx]

# LSU Department of Biological Sciences Writing Guide

Introductory Biology

***The most important rule: always keep the reader, your peers, in mind.***

### Title:

- Concisely convey what was done in the experiment.
- Include independent and dependent variables.
- Include the scientific name(s) of model organism(s).

### Abstract:

- Choose the most important information. What would you tell your peer about the experiment in a brief conversation?
- Include ~2-3 sentences from each section of lab report:
  - Introduction: importance of the study, research question, and prediction
  - Methods:
    - 1-2 sentences for data collection
    - 1-2 sentences for data analysis
  - Results: main trends (no specific values unless the value itself is a **vital** finding of the study)
  - Discussion: interpretations, big picture (real world application)

### Introduction:

- Present tense
- Background information: 1-2 paragraphs
  - Explain **important** topics surrounding the study.
  - Start broad and get more specific to your study.
- Narrow towards your study: 1-2 paragraphs
  - Introduce the problem or pattern to be studied.
  - Discuss your model organism(s) or study system(s).
  - Why is it an appropriate model organism(s) or study system(s)?
  - Why is the study important in the broader context of science and life?
- End narrow: 1 paragraph
  - What is the objective of your study?
  - Include hypotheses, and predictions with reasoning.

### Methods:

- Use third person, past tense, passive voice
  - Ex: ‘___ was measured…’
  - Do not reference your TA, lab partners, or yourself.
- Three main parts (**include as sub headers**):
  - *Experimental design*: 1 paragraph
    - how your experiment was set-up; # individuals & replicates
    - Assume the reader understands how to use equipment. Do not include steps like how to squeeze the pipette, collect termites from the container, etc.
  - *Data collection*: 1 paragraph per data collected
    - Diatoms study: 3 types of data collected (absorbance, biomass, [fucoxanthin])
    - what you measured and how you collected your data
  - *Data analysis*: 1 paragraph
    - Include appropriate data analysis, but do not explain what those analyses mean (do not explain trends).
    - Means with standard deviations; Scatterplot with trendline
    - Include what type of figures you developed (e.g. “means and standard deviations were plotted in a bar chart...”)
- Add reasoning to the beginning of sentences when necessary
  - Ex: ‘To increase accuracy, three replicates were used for each treatment.’; ‘To calculate concentration, the equation for the standard curve was used.’
- Write in your own words; do not copy straight from the protocol sheets
- Cite literature for methods obtained from previous studies
- Be concise

### Results:

- Use past tense
- Often the shortest section, but very important
- Do NOT discuss hypotheses or predictions here
- Use sub-headings to help group and organize data
- Four major points of a results section (may be combined for flow):
  - 1. Write results in terms of trends.
  - 2. Include important/meaningful values.
  - 3. State statistical significance if relevant.
  - 4. Reference figure(s).
  - Ex: ‘Plant height increased significantly with nutrient addition (p = 0.03, Figure 3).’
- Main components
  - Text: include facts only (no interpretations)
    - One paragraph per figure
    - Statistical results should be in parentheses
      - ANOVA Ex: ‘Average mass gain differed between the three populations (p= 0.003).’
      - T-test Ex: ‘The average mass gain of population B was significantly lower than average mass gain of Population A.’
      - Regression Ex: ‘The rate of mass gain in population A increased at a rate of 0.002 kg/month (p<0.0001, Figure 4). The rate of mass gain for population B, however, showed no significant growth over the course of the study (p=0.81, Figure 4).’
    - If the results were significant, indicate the *direction*.
      - Ex: ‘Average fitness and male size was correlated across population B (p=0.003, Figure 4). Larger males were *more fit*.’
  - Figures & Tables:
    - Must be referenced in the text by figure/table number
    - Title goes into caption (see below)
    - Caption Components: Figure/Table #. Title statement. Additional information.
      - *Number*: numerical order throughout document (Ex: Figure 1, Figure 2…Table 1, Table 2)
      - *Title statement*: general statement (not complete sentence)
      - *Additional information*: in complete sentence(s) to help stand alone
      - Be concise but include all relevant information.
      - Exact captions requirements will differ among courses.
    - Figure specifics:
      - Purpose: visually represent trends (scatterplot) and comparisons (bar graph; line graph)
      - Label axes with units
      - Caption location: below figure
    - Table specifics:
      - Purpose: when actual values are more important than trends, or when trends not present
      - Caption location: above table (“table top”)
      - Must reference in the text by table number; not related to figure numbering

### Discussion:

- Start narrow
  - Prediction and hypotheses
    - Restate trends and tie to prediction
    - Null hypothesis: reject or fail to reject
    - Alternative hypothesis: reject or support
  - Interpretation (Why?)
    - Make sense of your findings
    - Do not report values again unless absolutely necessary. P-values, F statistics, etc. are not necessary.
    - Do not falsify. If you did not find a difference between treatments, do not write about differences.
    - Support with literature; use in-text citations.
    - Unexpected results: When are unexpected results from experimenter error? Are errors genuine errors? If not, think of (and read about) some possible explanations for the unexpected results.
      - Do not be quick to discredit yourself. Think beyond human error. Is your study system the same as other studies (cite)? Are the methods the same (cite)? How might those differences affect results?
  - Limitations (be specific and logical). The scientific method has a number of limitations including:
    - Constrained by the extent of existing knowledge - Developing a hypothesis and designing an experiment is based on current human knowledge. However, until viruses were discovered many diseases could not be explained e.g. smallpox.
    - Design of experiment is limited to observation method and instrument - e.g. discovery of viruses depended on the discovery of the electron microscope. What methods would make study better?
    - Human error - e.g. mistakes can occur in recording observations or inaccurate use of measuring instrument.
    - Data interpretation - research findings are limited by human ability to interpret the results. Wrong interpretations can lead to wrong conclusions e.g. thalidomide was used to treat morning sickness in human pregnancy in 1950s. It was safely tested on many animals and then wrongly interpreted as safe for humans. However, the drug was not tested on embryo in womb. This caused limb deformities in babies. The drug was later withdrawn in 1961.
    - Is limited to the present - what is true now may not have been true in the past or in the future e.g. penicillin used to be effective against many bacteria but new strains have evolved that are resistant to penicillin. As changes occur, scientific theories may require updating or revision.
- End broad
  - Assign significance to your findings
  - Big picture - why is your study important outside of the classroom (i.e. who would be interested and why)
    - Relate your findings to your experimental question
    - Breadth of discussion should match breadth of introduction
  - Relate your study to real world applications. What is the big picture?
  - Include ideas for future studies that take it to the next level.
    - What future studies could you conduct that may build on your findings for the scientific community?
    - Make sure that your future study is a good research question.
    - **Note**: future studies do not include suggestions of “increase # of replicates” or basic technique improvements
- **edit to include more about null or non-significant results, what this means, and where to find literature

### References:

- Consult section-specific instructor for minimum number of references
- Use primary, reputable sources (Google Scholar, Web of Science)
- See course-specific rules for websites
- In-text citations must be in the Introduction, Methods, and Discussion sections
- Use consistent formatting for citations - APA
- Citations should contain author(s) names, year, article title, journal title, volume, and page numbers
- Format varies with course, just like it varies with journals.
