# Supplementary material for: Introducing a Novel Course-Based Undergraduate Research Experience Using Duckweed as a Model System
Source: Integr Org Biol. 2025 Dec 19;8(1):obaf049. doi: 10.1093/iob/obaf049 (PMC12802901; doi:10.1093/iob/obaf049)
Supplement: obaf049_Supplemental_Files [file obaf049_supplemental_files.zip › 07 Supplementary Materials/Supplementary Materials/31_Week05_ICA_AnnotatedBibliography.docx]

# Annotated bibliography

Continue to build your annotated bibliography in this working document. You will be asked to submit this document later. More than a simple list of references, an annotated bibliography gives a brief description of each paper for future reference and allows you to keep track of relevant papers that you find.

*Appenroth et al 1996*

- Full reference in APA format:
- Keywords listed on paper (if available):
- Why is this source relevant to our project? (1-2 sentences)
- Describe the main conclusions of this paper (2-3 sentences):

*Appenroth et al. 2002*

- Full reference in APA format:
- Keywords listed on paper (if available):
- Why is this source relevant to our project? (1-2 sentences)
- Describe the main conclusions of this paper (2-3 sentences):

*Dudley et al. 1987*

- Full reference in APA format:
- Keywords listed on paper (if available):
- Why is this source relevant to our project? (1-2 sentences)
- Describe the main conclusions of this paper (2-3 sentences):

** *replace this line with the in-text citation*

- Full reference in APA format:
- Keywords listed on paper (if available):
- Why is this source relevant to our project? (1-2 sentences)
- Describe the main conclusions of this paper (2-3 sentences):

** *replace this line with the in-text citation*

- Full reference in APA format:
- Keywords listed on paper (if available):
- Why is this source relevant to our project? (1-2 sentences)
- Describe the main conclusions of this paper (2-3 sentences):

** *replace this line with the in-text citation*

- Full reference in APA format:
- Keywords listed on paper (if available):
- Why is this source relevant to our project? (1-2 sentences)
- Describe the main conclusions of this paper (2-3 sentences):

** *replace this line with the in-text citation*

- Full reference in APA format:
- Keywords listed on paper (if available):
- Why is this source relevant to our project? (1-2 sentences)
- Describe the main conclusions of this paper (2-3 sentences):
