# Supplementary material for: Introducing a Novel Course-Based Undergraduate Research Experience Using Duckweed as a Model System
Source: Integr Org Biol. 2025 Dec 19;8(1):obaf049. doi: 10.1093/iob/obaf049 (PMC12802901; doi:10.1093/iob/obaf049)
Supplement: obaf049_Supplemental_Files [file obaf049_supplemental_files.zip › 07 Supplementary Materials/Supplementary Materials/32_Week06_PROTOCOL_MicrobialAdditionAndDataCollectionDay7.docx]

# Protocol: Microbial Addition and Data Collection – Day 7

## **Introduction**

This protocol describes the process for setting up the CURE Duckweed experiment to observe the effects of habitat fragmentation on plant-microbe symbiosis. It is imperative that you practice sterile techniques during this lab. Be cognizant of what you and your equipment touch – do not allow pipette tips to make contact with anything other than what you are transferring. Sterilize your gloves often with 70% EtOH.

## **Materials**

| - PPE: Gloves, goggles, coats, masks - Sterile Microplate - Bunsen Burner | - P10 or P100 micropipette - Sterile Pipette Tips - Ethanol |  |
| --- | --- | --- |

## **PROCEDURE: Microbial Addition**

1. Wash your hands thoroughly and don gloves and a mask.
2. Clean your lab bench and prep additional materials.
3. Spray ethanol onto the gloves before beginning in order to re-sterilize. Do not perform this step near an open flame. Set ethanol away from flame once completed.
4. Briefly vortex the microbial solution
5. Using a sterilized micropipette and sterile pipette tips, pipet 20 uL of the microbial solution into each test tube.
6. Vortex gently
7. Place test tubes back on the rack.

**Data Collection**

1. You will need to count the number of duckweed fronds in each test tube.
   1. Check with your instructor for approval before beginning
2. You can use the excel template uploaded to Moodle to record your data.
3. Once you have finished counting all duckweed fronds, you will need to sterilize your benchtop and obtain a sterile 96 well plate.
4. Vortex each test tube gently.
5. Pipette 100 uL of each test tube into the well plate using the following guide:


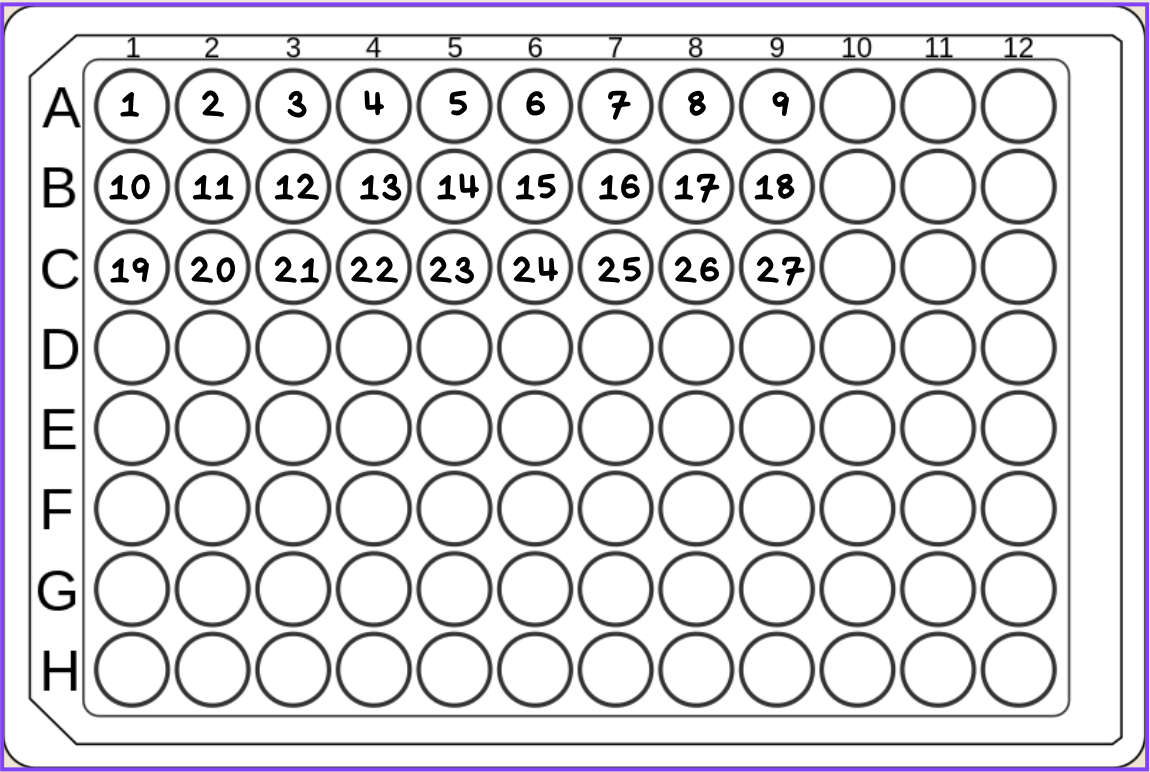


1. Place the 96 well plate into the Gen5 Microplate reader. It will automatically give you values for the Optical Density. You will need to record these values in your notebook and data table.

## **Clean-up**

- Return all items or discard in their proper receptacle. Gloves (only) go in the biohazard bag.
- Sterilize benchtops with EtOH and paper towels.
- Wash your hands well.
