# Supplementary material for: Introducing a Novel Course-Based Undergraduate Research Experience Using Duckweed as a Model System
Source: Integr Org Biol. 2025 Dec 19;8(1):obaf049. doi: 10.1093/iob/obaf049 (PMC12802901; doi:10.1093/iob/obaf049)
Supplement: obaf049_Supplemental_Files [file obaf049_supplemental_files.zip › 07 Supplementary Materials/Supplementary Materials/33_Week06_PROTOCOL_DataCollectionDay7.docx]

# Protocol: Data Collection Day 7

#### Introduction

This protocol describes the process of using ImageJ to calculate percentage area coverage of our duckweed samples. You will need a laptop to complete this protocol. We will be practicing taking quantitative measurements using imaging annotation software.

#### Preparation

1. Take photos of each well from your well plate. Make sure that you are paying attention to the well numbers. Take one photo for each well.
2. Navigate to the ImageJ home page (<https://imagej.net/ij/>) Search “ImageJ” in your browser if needed.
3. Select **‘Run ImageJ in Browser’**

#### Opening images in ImageJ

1. In ImageJ, click **‘File’** --> **‘Open’**. Then **‘Select Local File’**
2. Navigate to your Desktop and choose the image you saved.

#### Select a well to measure

1. Crop the photo so that only the well you wish to measure is in the frame.
2. Use your mouse to draw a square around the well you wish to measure.
3. Select **‘Image’** --> **‘Crop’**
4. Keep the edges of the well within the cropped image.
5. If you need to redo the crop, select ‘**Edit**’ --> ‘**Undo**’.

#### Counting Fronds

1. Count the number of duckweed fronds within each well using the **‘Multipoint’** tool
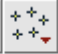
in the toolbar. Every time you click and add a point on each frond, the multitool will count the number of points.


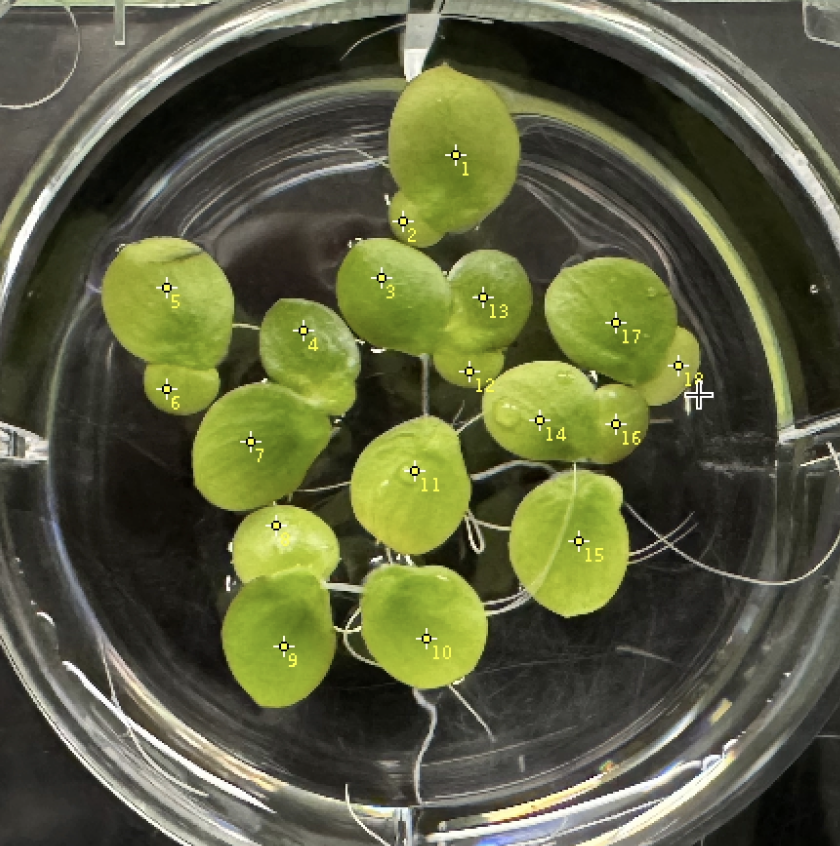


1. Record this number in your excel file under **‘Frond Count’.**
2. Compare your results with your group mates to ensure consistency.

#### Set your scale for measuring coverage

1. Use the ‘**Straight**’ tool
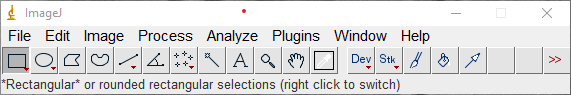
 in the toolbar to draw a line to represent the diameter of the well.
   1. Make sure you draw the line at an angle of 0.


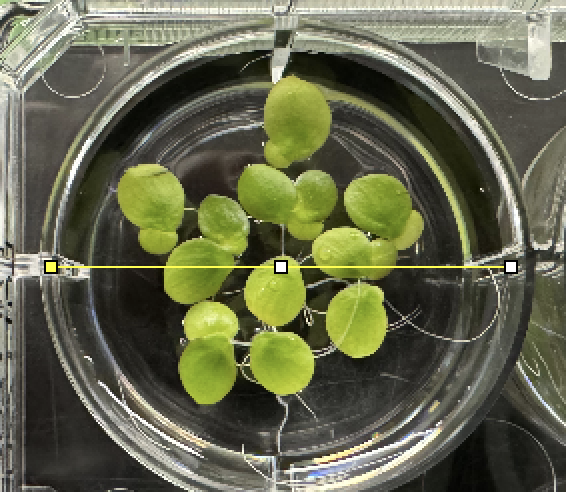


1. Select **‘Analyze’** -> **‘Set Scale’**
   1. Known Distance: 34.8￼
   2. Units: mm (millimeters)
2. ***Note: you will need to reset the scale for **every image** that you open in ImageJ.

#### Measure percent coverage

1. Use the **‘Freehand’** tool
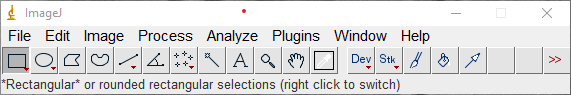
 to trace the outer edge of all the duckweed fronds.
2. Once you have outlined the entire mass of duckweed within the well, analyze the area by selecting **‘Analyze’ --> ‘Measure’**. Image J will automatically measure the area of your selection.
3. Record the measurement listed under **‘Area’** as **‘Duckweed Area’** in your data file. The area for each well has already been calculated.


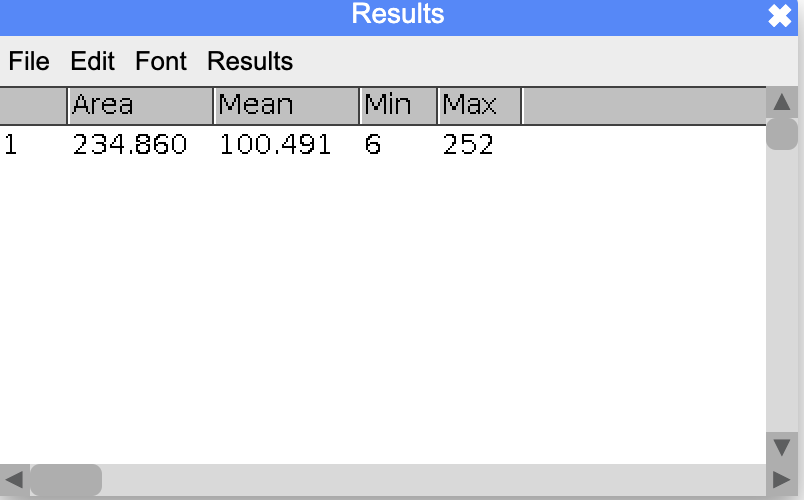


1. In excel, the column labeled **‘Percent Coverage’** should automatically update as you input the values.
   1. It is pre-set with the formula **Duckweed Area / Well Area * 100** to calculate the percentage.
2. Compare your results with your group mates to ensure consistency.

#### Repeat process for next image

1. Click the ‘X’ to close the current image – keep the ImageJ menu visible.
2. Repeat the protocol above, opening the next image file for the next well.
