# Supplementary material for: Introducing a Novel Course-Based Undergraduate Research Experience Using Duckweed as a Model System
Source: Integr Org Biol. 2025 Dec 19;8(1):obaf049. doi: 10.1093/iob/obaf049 (PMC12802901; doi:10.1093/iob/obaf049)
Supplement: obaf049_Supplemental_Files [file obaf049_supplemental_files.zip › 07 Supplementary Materials/Supplementary Materials/34_Week06_ICA_MethodologyDissectionDevelopment.docx]

# **ICA: Methodology Dissection & Development**

## (independent work; due via Moodle)

**Name**:

| **Text within Paper** | **Text Color** |
| --- | --- |
| Materials | **GREEN** |
| Experimental design (set-up of experiment) | **TEAL** |
| Data collection | **BLUE** |
| Data analysis | **PURPLE** |

Within each section below, you will see the guidelines listed from the Writing Guide, along with text from two key papers associated with this project. Using the color indicated, edit the text color to satisfy that guideline. As an example, some text has been highlighted for you. Use the remainder of the text to highlight key sentences to help form your own methods section.

## **Part I. Dissecting Relevant Methodologies**

## Paper:

O’Brien, A. M., Laurich, J., Lash, E., & Frederickson, M. E. (2020). Mutualistic outcomes across plant populations, microbes, and environments in the duckweed Lemna minor. Microbial ecology, 80, 384-397.

**Methods**

**Biological Materials**

We collected duckweed and associated microbes from ponds in the Greater Toronto Area in the summer of 2017 (Table S1). Our field sites likely vary in local zinc contamination due to their dispersed geographic location and proximity to urban inputs [52]. Moccasin Trail is urban and located 46 and 56 km from McCraney Pond (suburban) and Kelso (rural), respectively, which are 18 km from each other. Stoney Creek is rural, and the most isolated (132, 125, and 80 km from the others, alphabetically). We collected fronds of duckweed into 3 separate tubes (minimum 50 per tube), one each for duckweed culture, microbial culture, and microbial sequencing.

We transferred several individuals of field-collected live duckweed to growth media [63] in 500-mL glass jars, grew them to high density (≈ 500 plants per jar), and maintained stock lines from each site in a growth chamber with a cycle of 23 ◦C and 150 μmol/m2 lighting for 16 h followed by 18 ◦C and dark for 8 h. These lines are mixed samples from a population, and theoretically could contain multiple genotypes. However, since very little genetic diversity segregates in L. minor, especially within sites [28], we expect all individuals in a line from the same source site to have identical or nearly identical genotypes. Further, since duckweed from different locations are often distinguished by some genetic differentiation [28], lines isolated from different sites are likely to be distinct genotypes. We assume our lines reflect this most likely case and refer to them as “genotypes.”

Immediately after collection, we extracted microbes by pulverizing fresh tissue of 1 or 2 entire plants from each site, plating the slurry onto yeast mannitol medium agar plates, which we cultured at 29 ◦C for 5 days, and then stored at 4 ◦C. We used these cultures for experimental inocula. These inocula thus comprise the subset of the epiphytic and endophytic duckweed microbiomes that can live on yeast mannitol media. Beyond bacteria, other microbes such as fungi and diatoms are known to live on duckweed in the field [64, 65] and may also proliferate in our inocula. However, we expect the majority of taxa in the duckweed microbiome with large effects on duckweed to be bacteria, since these are responsible for many of the strong microbial effects on duckweed observed to date [33, 66–68]. Furthermore, while plant microbiomes can be functionally divided into external and internal fractions [32], the previously implicated taxa are likely to be external, or both external and internal [33, 66–68]. Therefore, we focused our efforts on bacteria in the duckweed microbiome, without differentiating endophytic and surface portions.

Bacterial communities of plants are notoriously diverse, especially when considering microbes colonizing external plant surfaces [32], and many bacterial taxa can fail to grow in standard laboratory culture [69]. We therefore considered it important to characterize the taxonomic diversity of the bacteria in our inocula and compare it to the diversity of bacteria present in the field. We extracted DNA from frozen field-collected L. minor tissue (stored frozen, ≈ 0.25 g of fresh tissue and adhered water) with DNeasy Powersoil (Qiagen) kits. We also extracted DNA from our yeast mannitol medium cultures to be used as inocula in the experiment using GenElute Bacterial Genomic DNA (Millipore-Sigma). We sent 10 ng of DNA of each field and culture sample to Genome Que ́bec (McGill University) for PCR amplification of 16s rDNA, normalization, and barcoded, paired-end, 250-base pair format sequencing on an Illumina MiSeq System. We sequenced the V3-V4 variable region with primers 341f-805r, since this region should show both good recovery and little bias for taxa found in plant microbiomes (Table S2, [70]). We received demultiplexed reads from the facility with adaptors pre- trimmed (an average of ≈ 100,000 reads per sample, ranging from 69,234 to 119,383) totalling ≈ 407 Mbp (mega base pairs). Sequence data from Moccasin field and culture samples are previously published (in review [57]), but the sequencing methods are the same as those for other samples. We processed reads with QIIME2 [71]; specifically, our pipeline cut adaptors (cutadapt), joined pairs (vsearch), and quality filtered (quality-filter) with default settings. We then used deblur to correct errors and assign amplicon sequence variants (ASVs), followed by the naive Bayesian classify- sklean pre-trained on the Greengenes database [72] to assign taxonomy to ASVs with a confidence of 70% or higher, and followed up with filter-taxa and filter-table to remove vascular plant mitochondrial and chloroplast reads, but we retained reads from non-bacterial microbes if they occurred (e.g., diatoms). We further analyzed results with R and package ade4 [73, 74] to compare the culturable and field- only portions of the microbiome, and to compare relative abundance of the culturable fraction (ASV in any culture; logged proportion) of bacterial families across inocula and field samples (at lower taxonomic levels, matrices are sparse).

**Experiment**

To test our hypothesis that duckweed fitness and phenotypes are altered interactively by abiotic and biotic factors, we experimentally manipulated environment (zinc), plant genotype, and microbial communities. Our three microbial treatments were as follows: none, added “home” microbes, and added “away” microbes from one of the other three populations. The particular “away” microbial community that was crossed with each duckweed source was selected randomly without replacement; specifically, randomization paired plants from Kelso with microbes from Moccasin, plants from McCraney with microbes from Kelso, plants from Moccasin with microbes from Stoney Creek, and plants from Stoney Creek with microbes from McCraney. We made a separate microbial inoculum from each site by taking a swab across the stored agar plate, adding to liquid yeast mannitol media, culturing in a shaker for 5 days at 30 ◦C and 200 rpm, and then diluting to control optical density across inocula. Because communities are made up of various species, the translation of optical density to cell count is inexact (see below), but inocula were approximately 200 cells/μL.

We placed each experimental plant into 2.5 mL of autoclaved growth media [63] in a 3.4-mL well of a 24-well plate. Before adding each plant, we removed surface microbes from the lab culture by dipping in 75% ethanol. Plants selected were approximately the same size, and all had one mature and one immature frond each. After adding plants to wells, 20 μL of the treatment-specific microbial inoculum was added to each well assigned to the inoculation treatment. Wells assigned to no inoculum treatment received 20 μL of sterile liquid culture media.

We crossed the 12 combinations of duckweed source and microbial source with low (0.86 μM) or high (3.44 μM) zinc water concentrations; four times the amount of ZnSO4 was added to the high compared to the low zinc treatment solution (a negligible increase of sulfate concentration). These concentrations of zinc represent natural [75] and elevated levels, such as those generated by waste discharge [76] or road runoff events [52, 75]. We repeated the full design over 10 replicates per treatment combination (source of microbes × source of duckweed × zinc level) for a total of 240 plants in 10 24-well plates. Replicates were assigned to wells at random, and as a result treatments were spatially interspersed within plates.

We photographed plates under a stationary camera at the beginning and end of the experiment. After taking the initial photograph, each plate was sealed with a gas-permeable membrane to prevent contamination among wells, and placed into a growth chamber set to the same conditions as above (23 ◦C and 150 μmol/m2 lighting for 16 h, 18 ◦C and dark for 8 h). After 10 days, we removed plates and photographed again to measure fitness and phenotypes (see Fig. S1). From the photographs, we manually counted the number of final fronds in each well, and used ImageJ [77] to measure the total pixel area of duckweed from start to finish, greenness of fronds (relative to blue and red), and the ratio of the total pixel area of the fronds in a well to the total perimeter of fronds in a well (aggregation) as a measure of the tendency of duckweed growth to remain attached in large units (Fig. S1).

We measured these response variables because of their links to fitness and conservation applications. Each duckweed frond is one potentially reproductive individual, and thus frond counts measure reproductive output, i.e., fitness. Final pixel area is tightly correlated to frond number (see “Results”), and may have two advantages as a measure of fitness: it meets assumptions of normality for statistical tests, and it incorporates frond size, which matters if larger fronds (individuals) are more likely to reproduce sooner or more often. Stress hastens daughter frond abscission rates in duckweed [78], i.e., reduced aggregation may be a sign of plant stress. More aggregated duckweed may also be more stable and dense on the water surface, which could increase shading of the water and reduce warming. Much local duckweed habitat in the sampling area is stormwater ponds, and reducing temperatures of the outflow water to streams is of concern for fish (e.g., [79–81]). Lastly, greenness should be a coarse indication of chlorophyll content [82, 83], and darker fronds may also be more effective at shading.

As a measure of microbial growth, we measured the optical density of suspended well solution, using starting growth media in the well as blank control. Plates were frozen and stored at − 20 ◦ C before optical density measures, so that microbial growth did not continue as measurements were taken. For a subsample of replicates (3 out of 10), we also measured microbial growth with an alternate method. Immediately after the end of the experiment (before freezing), we sampled 10 μL of well solution, diluted to 1 mL, and then plated 10 μL of the dilution onto petri dishes with yeast mannitol agar media and grew at 29 ◦C for 5 days. We then scored the number of colony-forming units on plates, as a measure of the total microbial growth. Some plates had colonies too numerous to accurately count, so we excluded those measurements (4 total) from analysis.

**Data Analysis**

To estimate interactive and independent effects of abiotic (zinc, E) and biotic (microbial community, C, duckweed genotype, G) factors, we analyzed data in R [73], using linear models in MCMCglmm (all models with 1,000,000 iterations, 1000 burn in, thinning by 100, unless otherwise noted [84]). We modelled change in duckweed pixel area, final frond number (all wells started with 2 fronds), optical density in wells, duckweed greenness, and duckweed aggregation as separate response variables. For all analyses, we determined whether differences were significant between specific treatments and contrasts using 95% highest posterior density intervals for the model- predicted means as calculated from the posterior distribution of parameter effects.

We first quantified the effect of inoculation with microbes on duckweed growth and microbial optical density to verify that manipulation of microbes was successful and that it positively affected duckweed. For optical density, we corrected for our minimum detection threshold (0.001), and took the natural log, since it was non-normally distributed, and fit this as the response variable to inoculum treatment in a linear model. We then subset all data to inoculated treatments for the remaining analyses below.

For each response variable, we tested whether main effects alone (G, C, E), two-way interactions (i.e., G × C, G × E), or three-way interactions (G × C × E) better described effects of host genotype, microbial community, and environment (zinc treatment). For each response variable, we determined the best model structure in stages. We first compared models with each main effect, each main effect and all 2-way interactions, and each main effect with all 2-way and 3-way effects. We further fit two versions of models with 2-way and 3-way effects, including interactions of microbial treatment and plant genotype where the source of each was either specified exactly, or categorized as “home” versus “away”. We selected the model structure with the lowest DIC [85]. Finally, we re-fit a simpler model with this structure, but removing non-significant terms. Frond number was treated as Poisson distributed (MCMC iterations increased to 10 million); all other variables were treated as Gaussian.

Finally, we investigated inter-relatedness between response variables. We asked whether associations exist between duckweed growth measures (plant fitness) and other duckweed traits. Similarly, we asked whether duck- weed growth was linked to optical density (total microbial growth, putatively linked to average microbial fitness across species) using treatment means; we expect positive corre- lations of fitness measures across partners in mutualistic interactions. We measured each association by again fitting linear models with MCMCglmm() as above for each pair of variables, with one as the dependent variable and the other as the independent variable. We also verified a relationship between colony-forming units (i.e., live cells, dependent variable) and optical density in the subset of wells where we measured both quantities. While both measures attempt to quantify microbial growth during the experiment, colony- forming units suffer from post-experiment competitive dynamics on petri plates, overgrowth obscuring some indi- vidual colonies, and data being laborious to collect. Optical density is not affected by post-experiment dynamics nor human counting error, but is unlikely to be equal across all microbe species (or even within species [86]), and may be influenced by dead cells. We transformed both variables with the natural log to meet normality assumptions, and for consistency with all previous analyses of optical density (see above).

## **Part II. Outlining your Methodology**

Use the methods below to outline the beginning of your Methodology section. Develop bullet points in your own words following the style mentioned in class. You will later turn these bullet points into paragraphs, so if you prefer to develop the sections below as paragraphs, you can do so. Appropriate sub-headers for your methodology have been included below.


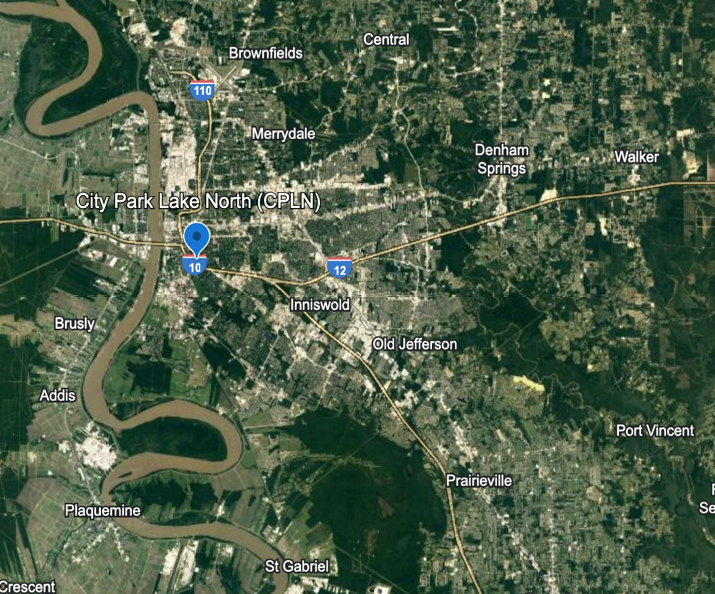

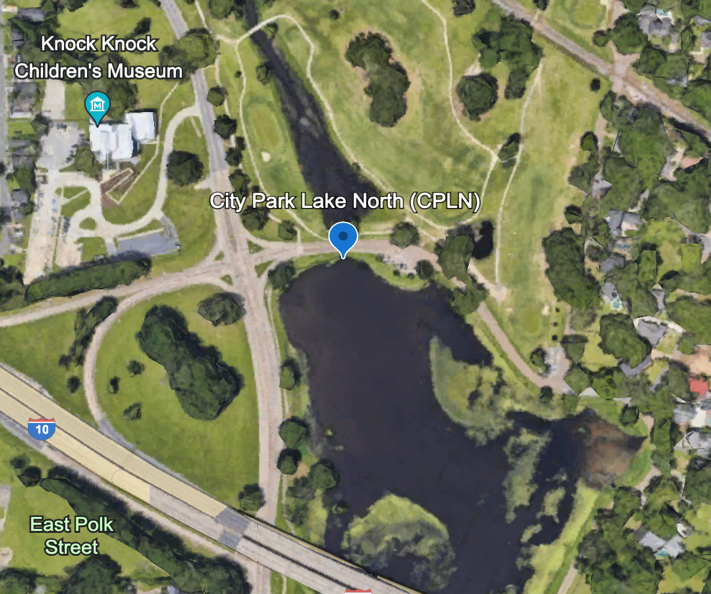


Figure 1: Map to show proximity to urban areas and site location of Duckweed sample used within this experiment.

**Methodology Outline**

- Biological Materials:
- Experimental Design:
- Data Collection:
