# Supplementary material for: Introducing a Novel Course-Based Undergraduate Research Experience Using Duckweed as a Model System
Source: Integr Org Biol. 2025 Dec 19;8(1):obaf049. doi: 10.1093/iob/obaf049 (PMC12802901; doi:10.1093/iob/obaf049)
Supplement: obaf049_Supplemental_Files [file obaf049_supplemental_files.zip › 07 Supplementary Materials/Supplementary Materials/36_Week07_PROTOCOL_DataCollectionDay14.docx]

# Protocol: Data Collection Day 14

## **Introduction**

This protocol describes the process for collecting data on both the duckweed and microbial treatments within the experiment. It is imperative that you practice sterile techniques during this lab. Be cognizant of what you and your equipment touch – do not allow pipette tips to make contact with anything other than what you are transferring. Sterilize your gloves often with 70% EtOH.

## **Materials**

| - Gloves - Goggles - Lab Coats - Masks | - P10 or P100 micropipette - Sterile Pipette Tips - Bunsen Burner | - Experimental treatments - Test tube racks - Ethanol - Sterile Microplate |
| --- | --- | --- |

**Data Collection**

#### Exercise I.

1. Retrieve your experimental test tubes from the grow racks.
2. To begin your data collection, count the number of duckweed fronds in each test tube.
   1. Check with your instructor for approval before beginning.
3. Use the excel template on Moodle to record your data.

#### Exercise II

1. Sterilize your benchtop and obtain a sterile 96 well plate.
2. Vortex each test tube gently.
3. Pipette 100 uL of each test tube into the well plate using the following guide:


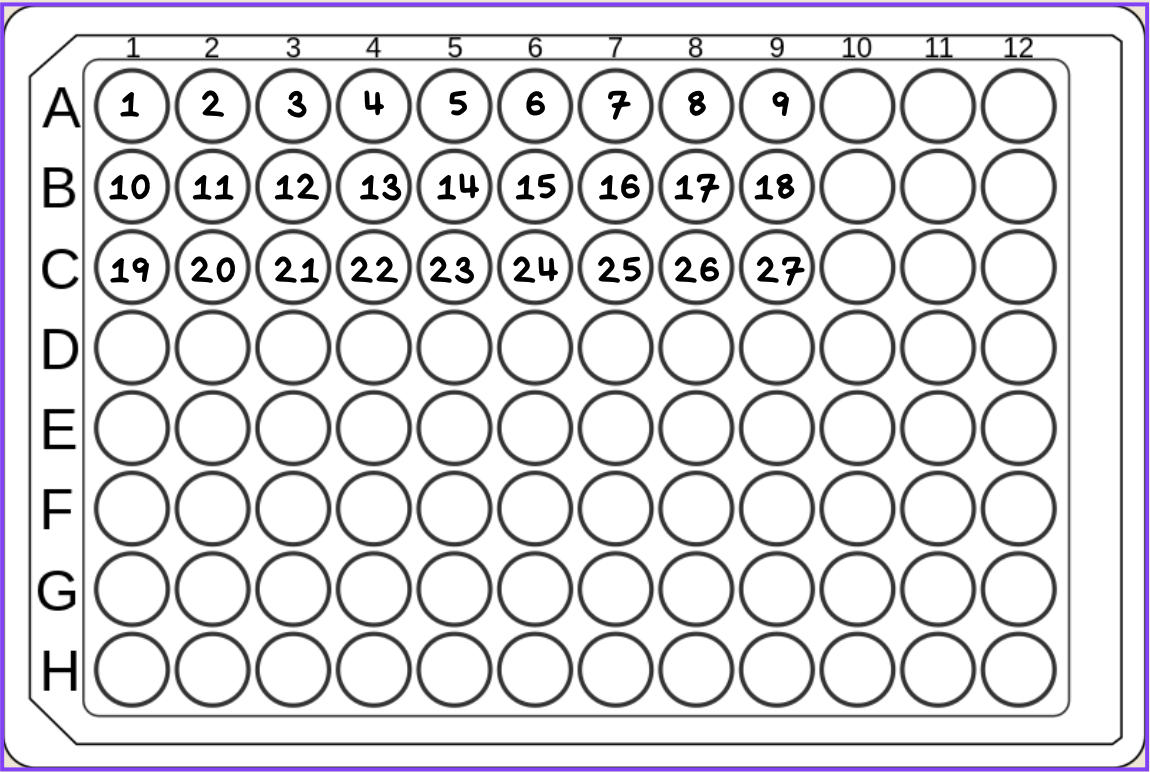


## **Clean-up**

- Return all items or discard in their proper receptacle. Gloves (only) go in the biohazard bag.
- Sterilize benchtops with EtOH and paper towels.
- Wash your hands well.
