# Supplementary material for: Introducing a Novel Course-Based Undergraduate Research Experience Using Duckweed as a Model System
Source: Integr Org Biol. 2025 Dec 19;8(1):obaf049. doi: 10.1093/iob/obaf049 (PMC12802901; doi:10.1093/iob/obaf049)
Supplement: obaf049_Supplemental_Files [file obaf049_supplemental_files.zip › 07 Supplementary Materials/Supplementary Materials/37_Week08_RESOURCES_ExcelStatstisticsTutorial.docx]

**Statistics using Excel**

1. Complete for both in-situ & ex-situ
2. Go to the figures that you created and decide which ones you will include in your final paper. NOTE: you can use nonsignificant figures in your paper if you think the information is interesting. Just be sure to write in a way that shows you understand the limitations of that model.
3. Follow the steps to determine significance, depending on what type of graph (below)
4. Make sure that you’re analyzing RAW data and not averages
5. Set-up your raw data as directed in the Excel Graphing Set-up document
6. Round to two significant figures to report as p-value in paper

**BAR CHART**: means & custom standard deviation on figure

- Significance: ANOVA p-value, adjusted p-values from t-tests
  - If p < 0.05, statistically significant
  - If p > 0.05, NOT statistically significant
- See here for info: <https://www.stattutorials.com/EXCEL/EXCEL_ANOVA.html>
- To determine if significance among groups, choose *ANOVA: Single Factor* from Data Analysis under Data tab
- If only two bars in bar chart, the ANOVA p-value is the only p-value you need
  - ANOVA p-value will determine if the two data sets are significant from each other
- How to write in results section (based on output below): "Diameter at breast height was significantly greater for trees in isolated populations than in non-isolated populations (F_1, 14_=18.29, p=0.00077)."


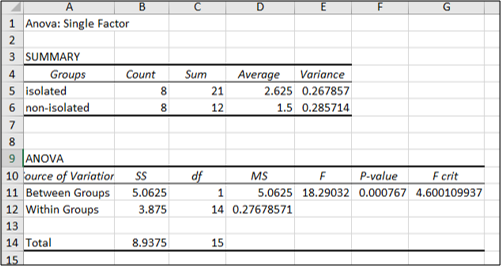


- If three or more bars in bar chart, choose *T-test: Two-Sample Assuming Unequal Variances* analysis in ToolPak
  - Use if you have three or more categorical variables along x-axis
  - Determines significance between two categorical variables; gives p-value
  - Must run a t-test on each pair of data sets separately
  - Gather p-values from output using the p-value from P(T<=t) two-tail
  - Must multiply each p-value by the total number of tests run to get adjusted p-value
  - Bonferonni adjustment - more conservative p-value due to several independent tests that don’t consider each other
    1. Calculate p-values of each individual t-test
    2. Determine how many t-tests you ran
    3. Multiply each p-value by the total # of tests run - this is your adjusted p-value for each pairwise test
    4. Round to two significant figures to report as p-value in paper
  - Also capture: df, t-Stat (use absolute value)
- How to write in results section: “The number of dead branches within canopies of live oaks was significantly greater when the infestation rating of *Tillandsia recurvata* was three than when it was one (t_4_=6.52, p=0.0029).”


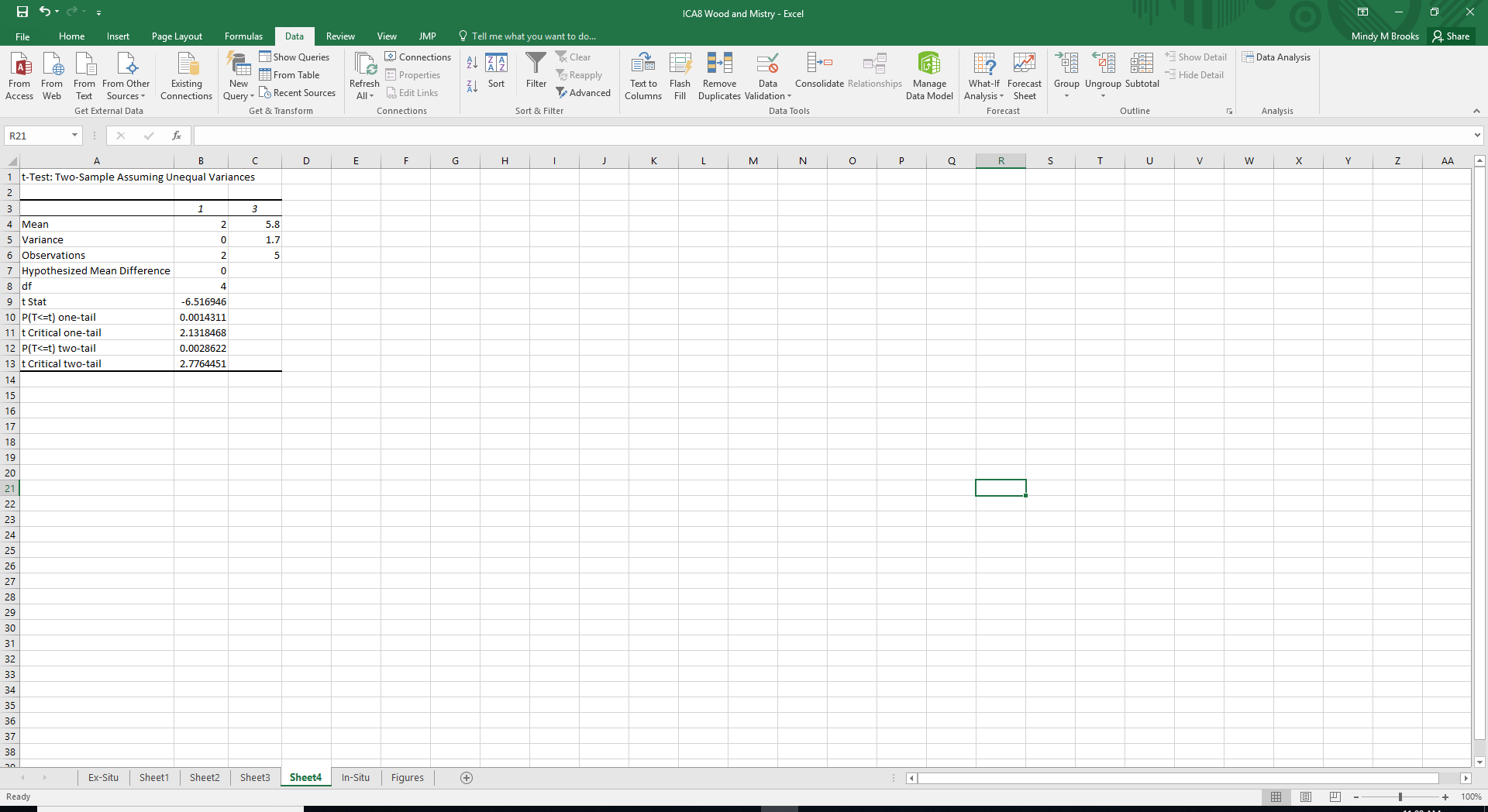


**SCATTERPLOT**: trendline, equation of line, R^2^ value on figure

- Significance: p(F)
- *Regression* analysis in ToolPak
  - Get p-value from Significance F
  - Also capture: equation of line (from graph), both df values, adjusted R^2^
- How to write in results section: “As the light intensity increased beneath the canopy of crepemyrtle trees, the infestation of *Tillandsia recurvata* decreased (F_1, 7_ = 8.25, p=0.024, y=1.722x + 0.87). Light intensity accounted for 47.55% of the variation found in the infestation rating (adjusted R^2^=0.4755).”


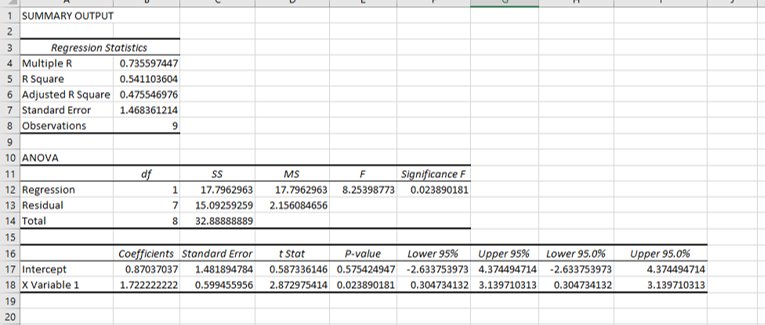


**LINE**: means & custom standard deviation

- You can use the t-test to determine significance between two data sets from a line graph, for example:
  - raw data for the high and medium groups at Day 21.
  - raw data for high group at Days 21 & 2.

Downloading the Analysis ToolPak:

- Most: <https://support.office.com/en-us/article/load-the-analysis-toolpak-in-excel-6a63e598-cd6d-42e3-9317-6b40ba1a66b4>
