# Supplementary material for: Introducing a Novel Course-Based Undergraduate Research Experience Using Duckweed as a Model System
Source: Integr Org Biol. 2025 Dec 19;8(1):obaf049. doi: 10.1093/iob/obaf049 (PMC12802901; doi:10.1093/iob/obaf049)
Supplement: obaf049_Supplemental_Files [file obaf049_supplemental_files.zip › 07 Supplementary Materials/Supplementary Materials/38_Week08_RESOURCES_JMPProtocolDataAnalysis.docx]

# JMP Protocol: Data Analysis

# Analyses of duckweed growth, and microbial concentration

## **Import your Data to JMP**

Each of you will graph four figures for this exercise: two to explore duckweed growth and two to explore microbial abundance. You will use your data sheet provided on. Moodle.

1. From Moodle, download the document ICA: Data Analysis and save to your computer. You will copy your graphs and statistical data into here.
2. Open JMP. If prompted for license, select the SID license file saved on your desktop.
3. From JMP Starter window (Mac) or JMP Home Window (PC), import your **Diatom Data** to JMP
   1. Be sure to use the **Desktop app** of Excel and not OneDrive.
   2. Click File, Open [Diatom Data]
   3. Click Import
4. Data opens in JMP
5. If there are dots at the bottom of the first 3 columns, right click on the row number (furthest left column) and choose “Delete Rows”

## **Opening Data in JMP**

1. To open your data in JMP, you will first need tocopy your data file. In the copy data file, delete the mean cells on the side of your excel file. So you will only have the columns with all of your data.
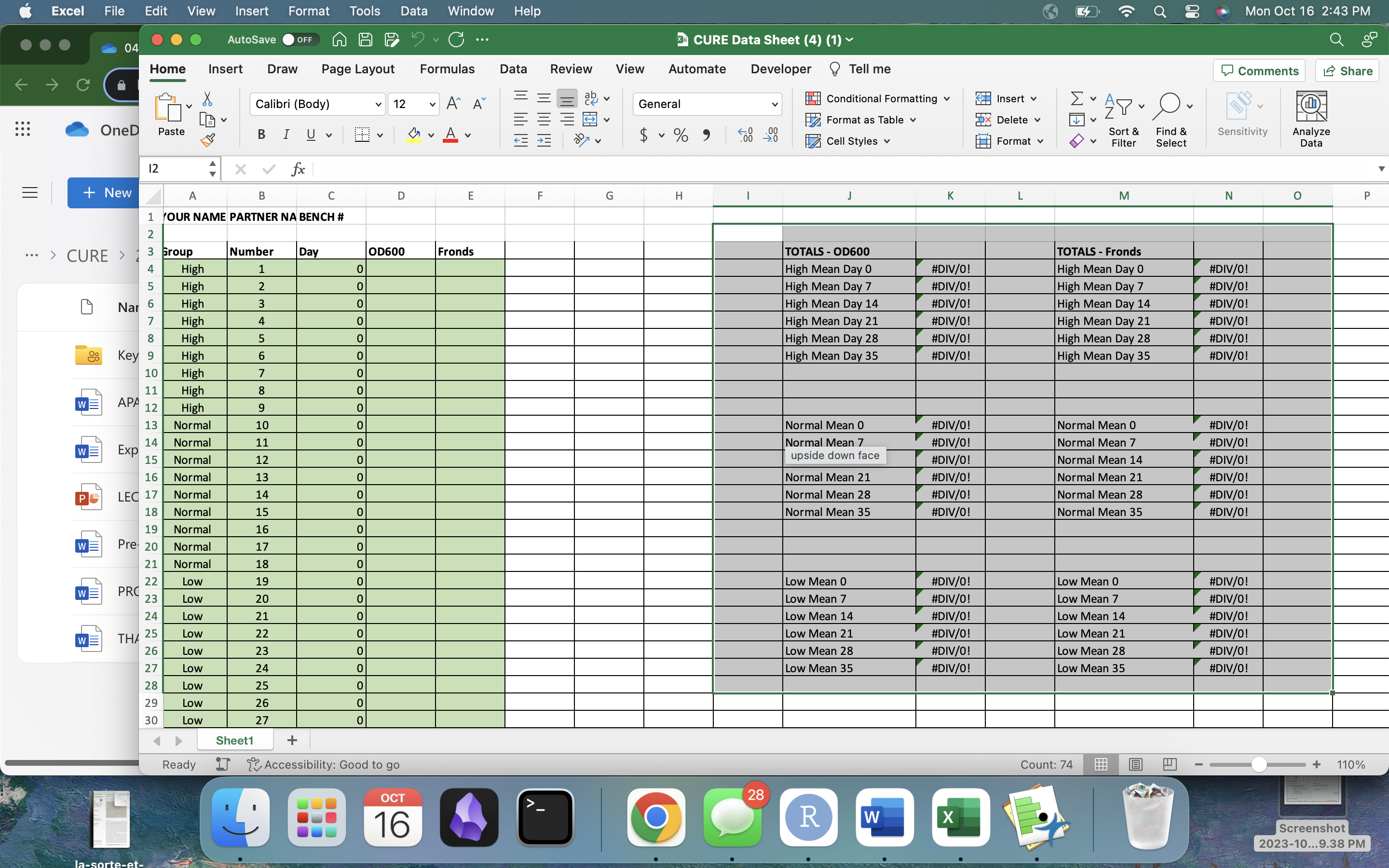


** Delete the gray highlighted selection, but save the values. Do not delete this section in the original data file. Only the copied file.

1. Select “Open” in JMP and import your data file


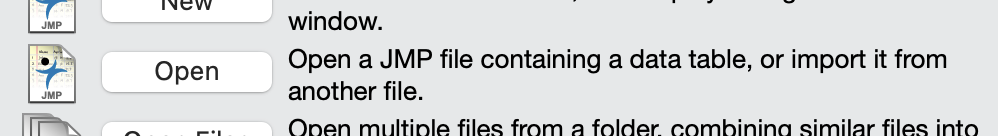


1. Adjust the data file so your headings are at the top using the following numbers. The data will Start with Row 4:


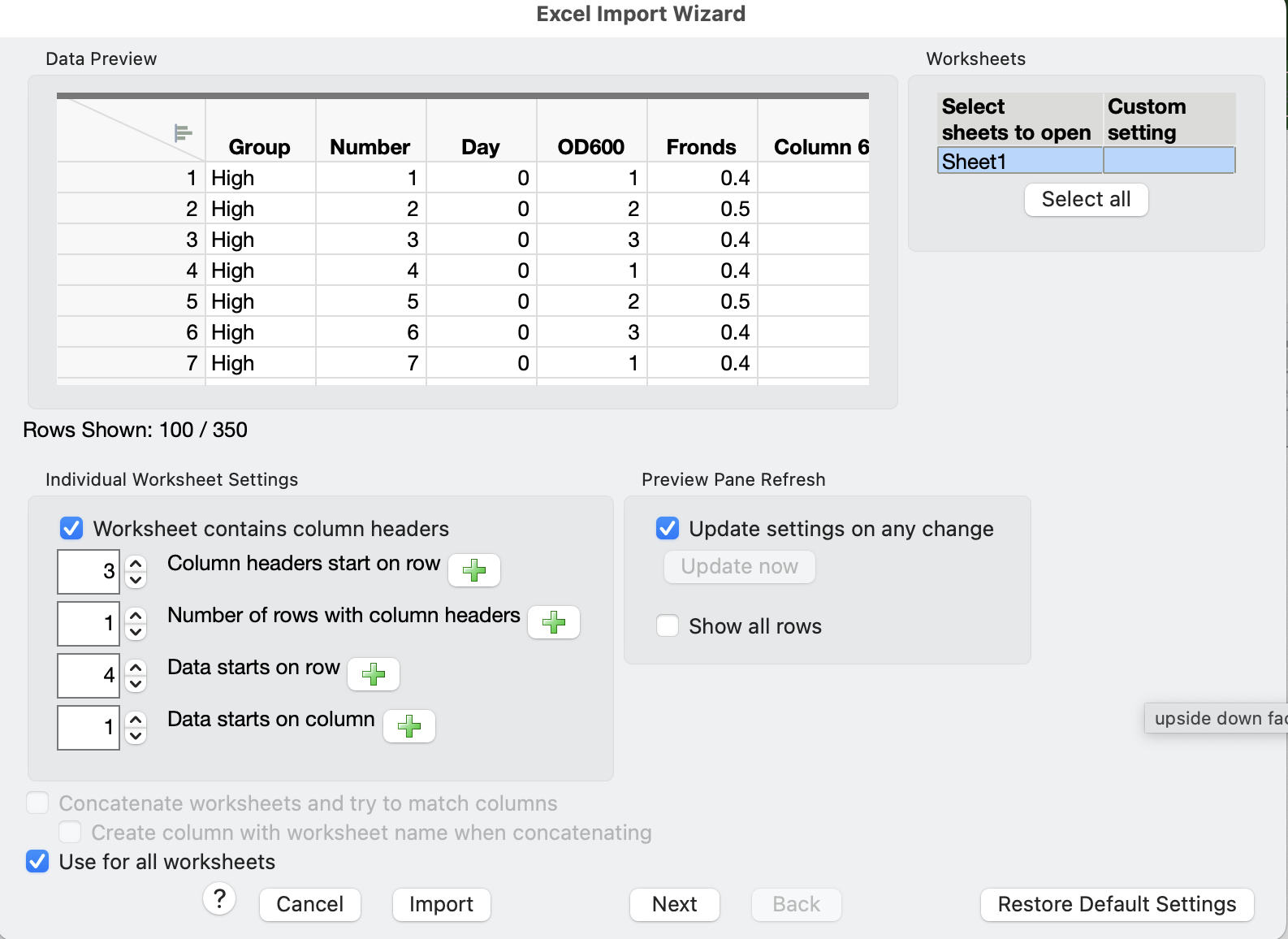


1. Press NEXT, The data will end at row 165.

##
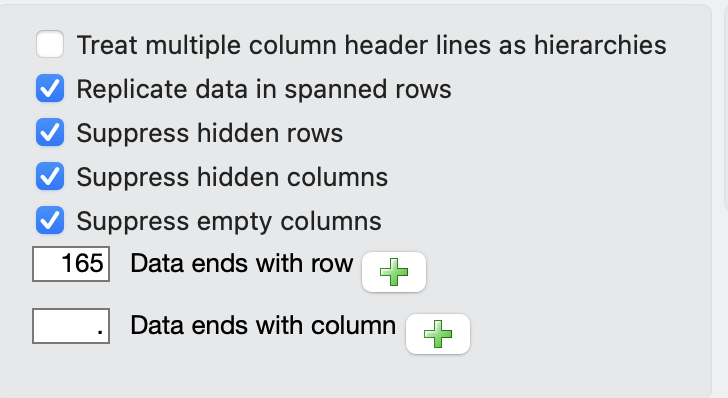


1. Press IMPORT

## **Part I. Scatterplot with linear trendline: OD600 over time**

In this analysis, you will plot raw data points for your chosen temperature intensity study, along with a trendline. The purpose of this figure it to show you how each treatment changed over time – you are not comparing treatments with this analysis. The slope of the trendline tells you the rate at which the treatment changed, as well as the direction (+ or -). You will find the p-value associated with the slope of the trendline, which indicates if the slope is significantly different from zero.

1. From imported data window, select Graph and then Graph Builder
2. Select scatter plot with linear trend line
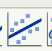
 (don’t deselect the scatterplot
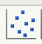
 )
3. Under **Variables**:
   - Drag Day to bottom X-axis
   - Drag Observed Absorbance (OD600) to Y-axis
   - Drag Group to Overlay *(top right corner)*
4. Under **Statistics on the top banner:**
   - Select Equation
   - Unselect Fit
   - Select Caption Box for all data points & lines. The legend may automatically generate and be visible to the right of the screen. To check, you can select the red arrow next to Graph Builder and ensure that “Show Legend” is checked.
5. Remove the title above graph, click & delete text
6. Change the font of the axes and axes label by:
   - Right click on axes, click axis settings then font and change font to 10 and click bold.
   - Right click on axes title, then font and again change font to 10 and bold.
7. Click Done
8. If need to change something after clicking Done:
   - Click red arrow next to Graph Builder & check Show Control Panel
9. If you change something that you cannot undo, the easiest thing to do is start over.
10. To paste the graph into a Word document
    - Right click on graph, Edit, Copy Graph
    - Open Word & paste
11. Repeat this process for **Fronds**

## **Part II. Line graph of means and standard error (ANOVA and t-tests)**

In this analysis, you will compare treatments to each other. Similar to the scatterplot, you will plot all three treatments, but instead of raw data, JMP will plot the means and standard errors. By plotting means and standard error, you can then run statistical analyses to determine if treatments are significantly different at each reading (day 0, 7, 14, 28).

1. Return to your data window within JMP
2. Select Graph and then Graph Builder
3. Select the Line Graph
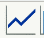

4. Under **Variables**:
   - Drag Day to bottom X-axis
   - Drag OD600 to Y-axis (left hand side of graph)
   - Drag Group to Overlay (top right corner)
5. Check legend for all data points & lines. The legend may automatically generate and be visible to the right of the screen. To check, you can select the red arrow next to Graph Builder and ensure that “Show Legend” is checked.
6. Remove the title above graph, click & delete text
7. To add error bars, select error interval and then standard error.
8. Change the font of the axes and axes label by:
   - Right click on axes, click axis settings then font and change font to 10 and click bold.
   - Right click on axes title, then font and again change font to 10 and bold.
9. Click Done
10. If need to change something after clicking Done:
    - Click red arrow next to Graph Builder & check Show Control Panel
11. If you change something that you cannot undo, the easiest thing to do is start over.
12. To paste the graph into a Word document
    - Right click on graph, Edit, Copy Graph
    - Open Word & paste

#### Analysis of Variance (absorbance means)

The p-value associated with an analysis of variance, or ANOVA, will tell you if there are any significant differences among your three treatments. Since the ANOVA doesn’t tell us which treatments are different from each other, you will run a Student’s t-test to determine which treatments are statistically different or statistically the same. The Student’s t-test uses alphabetic letters to decipher which treatments are same or different. If the letters are the same, the treatments are not significantly different from each other.

1. From main menu at top: Analyze --> Fit Y by X
2. Model Specification window opens.
3. Under **Select Columns**:
   - Drag OD600 to Y, Response
   - Drag Group to X, Factor
   - Drag Day to By
   - Click OK
4. “Oneway Analysis of OD600 by Replicates” will open
5. Click the red arrow & select the following for Time = 0
   - Means/Anova
   - Means and Std Dev
   - Compare Means --> select Each Pair, Student’s t
6. Tables & figures will open below
7. Collapse the following by clicking the grey arrow:
   - Summary of Fit
   - Confidence Quantile
   - LSD Threshold Matrix
   - Ordered Differences Report
8. Repeat #s 27-29 for the “Oneway Analysis of OD600 by Replicates” for Days 7, 14, & 28
9. Collect your Data.

- **Means & Std Dev**:
  - Record Mean & Std Error for each treatment in **Table 1**.
  - Find this on ‘means oneway anova’.
- **Analysis of Variance, Prob > F column**: Record p-values in **Table 2**.
  - If p-value > 0.05, the treatments are not significantly different from each other
  - If p-value ≤ 0.05, at least one treatment is significantly different from the others
- **Connecting Letters Report**: Record the letter associated with each treatment in **Table 3**.
  - If treatments share letters, they are not significantly different from each other
  - If treatments have different letters, they are significantly different from each other

1. NOTE: only include Student’s T-test in your writing assignments if the ANOVA p-value shows significance.
2. **Repeat for Fronds**
