# Supplementary material for: Introducing a Novel Course-Based Undergraduate Research Experience Using Duckweed as a Model System
Source: Integr Org Biol. 2025 Dec 19;8(1):obaf049. doi: 10.1093/iob/obaf049 (PMC12802901; doi:10.1093/iob/obaf049)
Supplement: obaf049_Supplemental_Files [file obaf049_supplemental_files.zip › 07 Supplementary Materials/Supplementary Materials/40_Week08_ICA_FiguresAndTables.docx]

# ICA: Results Figures and Tables

Your FWA1 included a full Introduction and Methodology, along with a partial References. In Part I and II of this guide, you will develop a Results section for FWA2 with your current data. Below, you will include your figures, tables, complete captions, and trends seen within the figures. Trend sentences are usually the first of each paragraph within the Results section; thus, you are essentially developing your topic sentences for each paragraph. Recall from the Writing Guide that each figure should have its own paragraph. You will need to develop these paragraphs for FWA2.

In Part III of this ICA, you will continue building your methodology by developing the final subsection of your methods – Data Analysis. For this section, include a summary of analyses that you performed but do not include any of the actual results or trends.

## **Part I. Results – Figures**

1. Line Graph: Percent Coverage between Treatments over time

Copy & paste your figure and add a complete caption below.

Include 1-2 sentences that state general trends found (note: trends do not go into the caption – this will be the first sentence of your results paragraph to describe this figure).


1. Line Graph: Number of Fronds between Treatments over time

Copy & paste your figure and add a complete caption below.

Include 1-2 sentences that state general trends found (note: trends do not go into the caption – this will be the first sentence of your results paragraph to describe this figure).


1. Box-Plot: Percent Coverage between Treatments

Copy & paste your figure and add a complete caption below.

Include 1-2 sentences that state general trends found (note: trends do not go into the caption – this will be the first sentence of your results paragraph to describe this figure).


1. Box-Plot: Number of Fronds between Treatments

Copy & paste your figure and add a complete caption below.

Include 1-2 sentences that state general trends found (note: trends do not go into the caption – this will be the first sentence of your results paragraph to describe this figure).


## **Part II. Results – Tables of Descriptive Statistics**

## ANOVA

Interpreting an ANOVA: A two-way ANOVA is used when you have two independent categorical variables and you want to understand their individual effects as well as the interaction effect on a dependent variable. From the data and context you've provided, it seems you have the following:

- Treatment (categorical): Different treatments applied in your experiment.
- Day (categorical): The time points at which measurements are taken can be treated as categorical if you are interested in the specific day effects rather than the trend over time.
- Number of Fronds or Percent Coverage (continuous): These are the dependent variables being measured.

The reasons for performing a two-way ANOVA on this data would be:

- To determine if there is a statistically significant effect of Treatment on the dependent variable (e.g., Number of Fronds or Percent Coverage).
- To determine if there is a statistically significant effect of Day on the dependent variable.
- To determine if there is an interaction between Treatment and Day. An interaction effect would mean that the effect of Treatment on the dependent variable changes depending on which Day it is, or vice versa.

If the ANOVA finds a significant interaction effect, this suggests that the impact of the treatment is not consistent across the different days. For example, a treatment might be more effective on earlier days and less effective later, or vice versa.

In summary, a two-way ANOVA is appropriate when you want to understand the effects of two independent categorical variables on a dependent variable and particularly when you're interested in whether there's an interaction between these two independent variables.

1. ANOVA: Percent Coverage between Treatments

Copy & past your table and add a complete caption above.

Include 1-2 sentences about the statistics found.

1. ANOVA: Number of Fronds between Treatments

Copy & past your table and add a complete caption above.

Include 1-2 sentences about the statistics found.

## TUKEY TEST

Interpreting the Tukey Tests: The 'reject' column in the results of the Tukey HSD (Honest Significant Difference) test indicates whether the null hypothesis for the pairwise comparison can be rejected or not. “True” means that the null hypothesis (that there is no difference between the group means) is rejected, indicating that there is a statistically significant difference between the group means at the chosen significance level (in this case, α = 0.05). “False “means that the null hypothesis is not rejected, indicating that there is not a statistically significant difference between the group means at the chosen significance level.

1. Tukey Test: Percent Coverage between Treatments

Copy & paste your table and add a complete caption above.

1. Tukey Test: Percent Coverage between Days

Copy & paste your table and add a complete caption above.

1. Tukey Test: Number of Fronds between Treatments

Copy & paste your table and add a complete caption above.

1. Tukey Test: Number of Fronds between Days

Copy & paste your table and add a complete caption above.

## **Part III. Methods – Data Analysis Subsection**

Methodology

**Data analysis** *(develop a paragraph that describes your analyses but not your results)*
