# Supplementary material for: Introducing a Novel Course-Based Undergraduate Research Experience Using Duckweed as a Model System
Source: Integr Org Biol. 2025 Dec 19;8(1):obaf049. doi: 10.1093/iob/obaf049 (PMC12802901; doi:10.1093/iob/obaf049)
Supplement: obaf049_Supplemental_Files [file obaf049_supplemental_files.zip › 07 Supplementary Materials/Supplementary Materials/41_Week08_ICA_DataAnalysisReport.docx]

# ICA: Data Analysis

## (independent work)

Your FWA1 will include a full Introduction and Methodology, along with a partial Results and References. In Part I of this ICA, you will finish building your methodology by compiling relevant pictures of your CURE project – these can include the set-up on the grow room, pictures of you and your partner taking measurements, etc. Pictures are considered figures, thus your first picture will be Figure 1. Likewise, all figures (i.e., pictures) should have a complete caption. You will also develop the final subsection of your methods – Data Analysis. Remember that this is independent work.

In Part II, you will develop a partial Results section for FWA1 (a full Results section will be included in FWA2 later). Below, you will include your three graphs, complete captions, and trends seen within the figures. Trend sentences are usually the first of each paragraph within the Results section; thus, you are essentially developing your topic sentences for each paragraph. Recall from the Writing Guide that each figure should have its own paragraph. Instead of a full paragraph for each figure, FWA1 will include the trend sentences only. The rest of the paragraph will be developed for FWA2.

## Part I. Methods – Data Analysis

Methodology

**Experimental design (***Insert pictures taken of set-up; include a caption for each picture)*

**Data collection** *(Insert your best picture for each process; include a caption for each picture)*

**Tables and figures representing data do not belong in this section.

*Optical Density*

*Duckweed Growth*

**Data analysis** *(develop a paragraph that describes your analyses but not your results)*

## Part II. Results – Figures & Trends

#### Line graph of means and standard error within each treatment: OD600 readings

Copy & paste (no screenshots) your figure and add a complete caption below.

Include 1-2 sentences that state general trends found (note: trends do not go into the caption – this will be the first sentence of your results paragraph to describe this figure).


What are some important values and comparisons that should be made in your paper?


#### Line graph of means and standard error between each treatment: OD600 readings

Copy & paste (no screenshots) your figure and add a complete caption below.

Include 1-2 sentences that state general trends found (note: trends do not go into the caption – this will be the first sentence of your results paragraph to describe this figure).


What are some important values and comparisons that should be made in your paper?


#### Line graph: duckweed growth within each treatment

Copy & paste (no screenshots) your figure and add a complete caption below.

Include 1-2 sentences that state general trends found (note: trends do not go into the caption – this will be the first sentence of your results paragraph to describe this figure).


What are some important values and comparisons that should be made in your paper?


#### Line graph: duckweed growth between each treatment

Copy & paste (no screenshots) your figure and add a complete caption below.

Include 1-2 sentences that state general trends found (note: trends do not go into the caption – this will be the first sentence of your results paragraph to describe this figure).


What are some important values and comparisons that should be made in your paper?
