# Supplementary material for: Introducing a Novel Course-Based Undergraduate Research Experience Using Duckweed as a Model System
Source: Integr Org Biol. 2025 Dec 19;8(1):obaf049. doi: 10.1093/iob/obaf049 (PMC12802901; doi:10.1093/iob/obaf049)
Supplement: obaf049_Supplemental_Files [file obaf049_supplemental_files.zip › 07 Supplementary Materials/Supplementary Materials/43_Week09_ICA_DissectingResultsAndDiscussion.docx]

# **ICA: Dissecting Results & Discussion**

**Name**:

In this guide, you will pick apart a paper about turion development in two aquatic carnivorous plants. Within each section below, you will see the guidelines listed from the Writing Guide, along with text from the Adamec 2023 paper. Using the color indicated, edit the text color within the paper’s text below that satisfy that guideline.

## **RESULTS**

| **Text within Paper** | **Text Color** |
| --- | --- |
| Trends | **GREEN** |
| Important values from figures | **TEAL** |
| Reference to figures | **BLUE** |
| Significance | **PURPLE** |

Induced shoot apices in both experimental species were maturating during the expected course of decreasing mean, minimum, and maximum temperatures in September and October (Table S1) but the turions fully maturated and detached as late as the end of October (UA) or at the beginning of November (AV), when the minimum water temperature reached to 4°C to 6°C.

The aim of the investigation of young trap-free leaves/shoots of both species was to find changes of some physiological markers during turion development. Gasometric measurements revealed some differences in young leaves/shoots between both species. In AV, the shoot RD decreased significantly from that in the controls as early as Stage I and then was constant, while in UA, the foliar RD decreased significantly as late as Stage II (Figure 2a). Consistently in both species, PN values in the same young organs were maximal in the summer growing controls and at turion Stage I and were declining by 44–59% at further Stages II and III (Figure 2b). The shoot content of chl. a + b was the highest or the same at Stage I during turion development in AV but was almost constant from the summer controls to Stage II in UA and declined afterwards (Figure 2c,d). In both species, the content of carotenoids was the same during turion development. The shoot N and P contents in AV were the highest in plants at turion Stage II (Figure 3). However, in UA, the foliar N and P content was significantly the highest in growing control plants and declined consistently during next turion development. Shoot DMC mildly increased at Stage I and then was constant in AV, while it decreased at Stage III in UA leaves (Figure 4). Taken together, all results indicate consistently that in UA, young leaves enter senescence during turion maturation earlier and their N and P stores are reutilized and allocated to maturing turions more effectively than in AV. In both species, the DMC increased gradually from ~11% in the apices of summer-growing plants to ~26% in Stage III turions (Figure 4). In AV shoots, no significant linear correlation was found between PN and chl. a + b (r = .32, p = .14, n = 23) and between PN and RD during turion development (r = .15, p = .49, n = 23); however, both correlations were significant in UA leaves (r = .80, p = .0001, n = 23; r = .74, p = .0001, n = 23, respectively). In both species, PN correlated with neither N nor P contents in shoots/leaves.


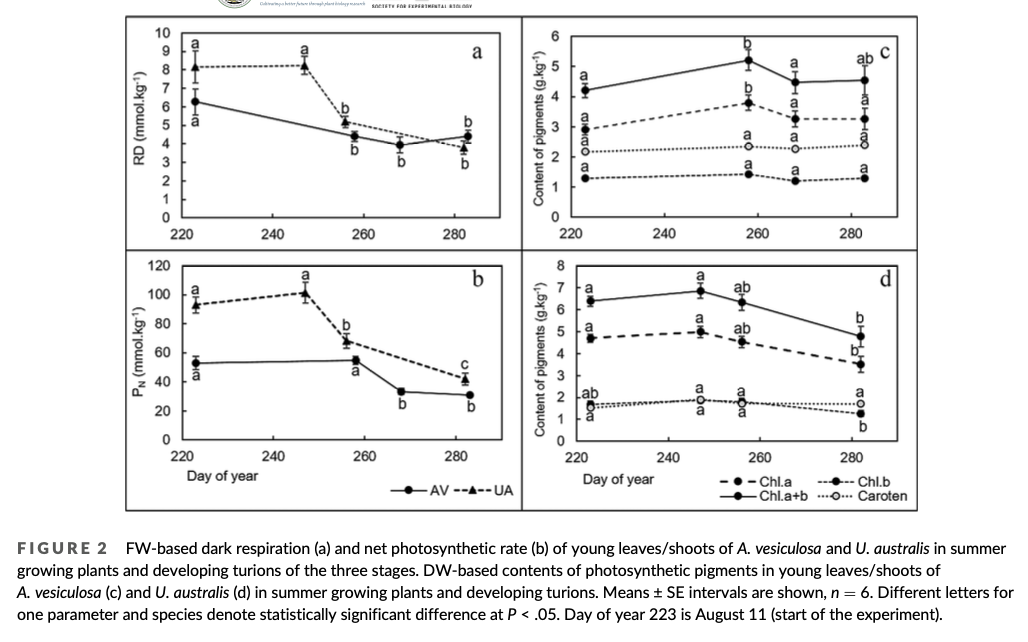


Cytokinin profiles in shoot apices developing from summer growing plants to mature turions revealed considerable differences between both species (Tables 1, S2, and S3). In AV, the total content of CKs (tZ, cZ, DHZ, and iP) as well as of biologically active forms (free bases + ribosides) did not differ significantly between shoot apices of summer growing plants and developing or mature turions. The prevailing CK types in AV developing turions were tZ and DHZ. The content of tZ culminated at Stage II and was significantly the lowest in mature turions, while that of DHZ was gradually rising during the turion development; in contrast, the iP content was steeply decreasing. Surprisingly, in AV apices, the proportion of active CK forms was relatively constant (55–73%) during turion development. In UA, the content of both tZ and DHZ decreased significantly at Stage III and in mature turions, while the trends of the cZ and iP contents were ambiguous and non-significant (Table 1); iP was always the dominant CK type. The total CK content was relatively stable, but that of active forms was gradually decreasing from 148 ± 15 nmol kgdw1 in summer growing plants to 10 ± .1 nmol kgdw1 in mature turions. Therefore, the percentage of active CK forms was markedly decreasing during turion development (from 7.0 to .48%). In UA, the content of active CK forms was thus 3.4–40 times lower than that in AV at the same developmental stages even though the total CK contents pre- vailed 2–3.6 times in UA.


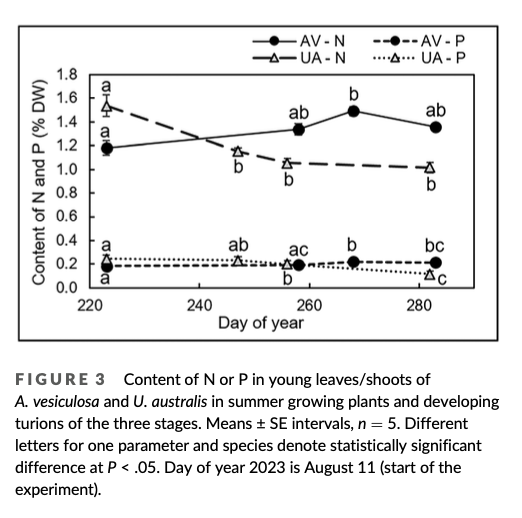


Auxin profiles in shoot apices developing from summer growing plants to mature turions in both species markedly differed from CK profiles (Tables 2, S2, and S3). In AV, the contents of the four auxin forms—indole-3-acetic acid (IAA), 2-oxindole-3-acetic acid (oxIAA), indole-3-acetyl aspartic acid (IAAsp), and indole-3-acetyl-β-1-O- D-glucose (IAGlu)—and their total content were significantly the lowest (except for IAGlu) either in developing turions at stage III or in mature turions. The IAGlu content was always minimal and below the limit of determination. The proportion of IAA as the only active auxin form to the total auxin content was increasing from summer growing plants, culminated significantly at Stage III but was followed by a steep decline below the limit of determination in mature turions. Overall, in AV, the IAA proportion was only within .014–3.2%. The ABA content in AV shoot apices was below the limit of detection in summer growing plants but was gradually rising—the rise started at Stage I and culminated in mature turions (13,204 ± 1,200 nmol kgdw1; Table 2).


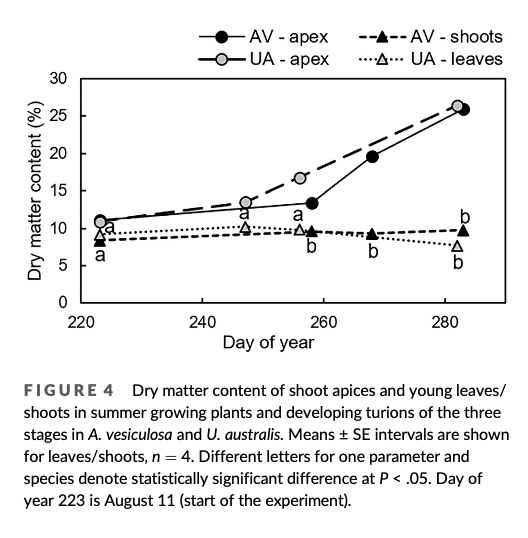


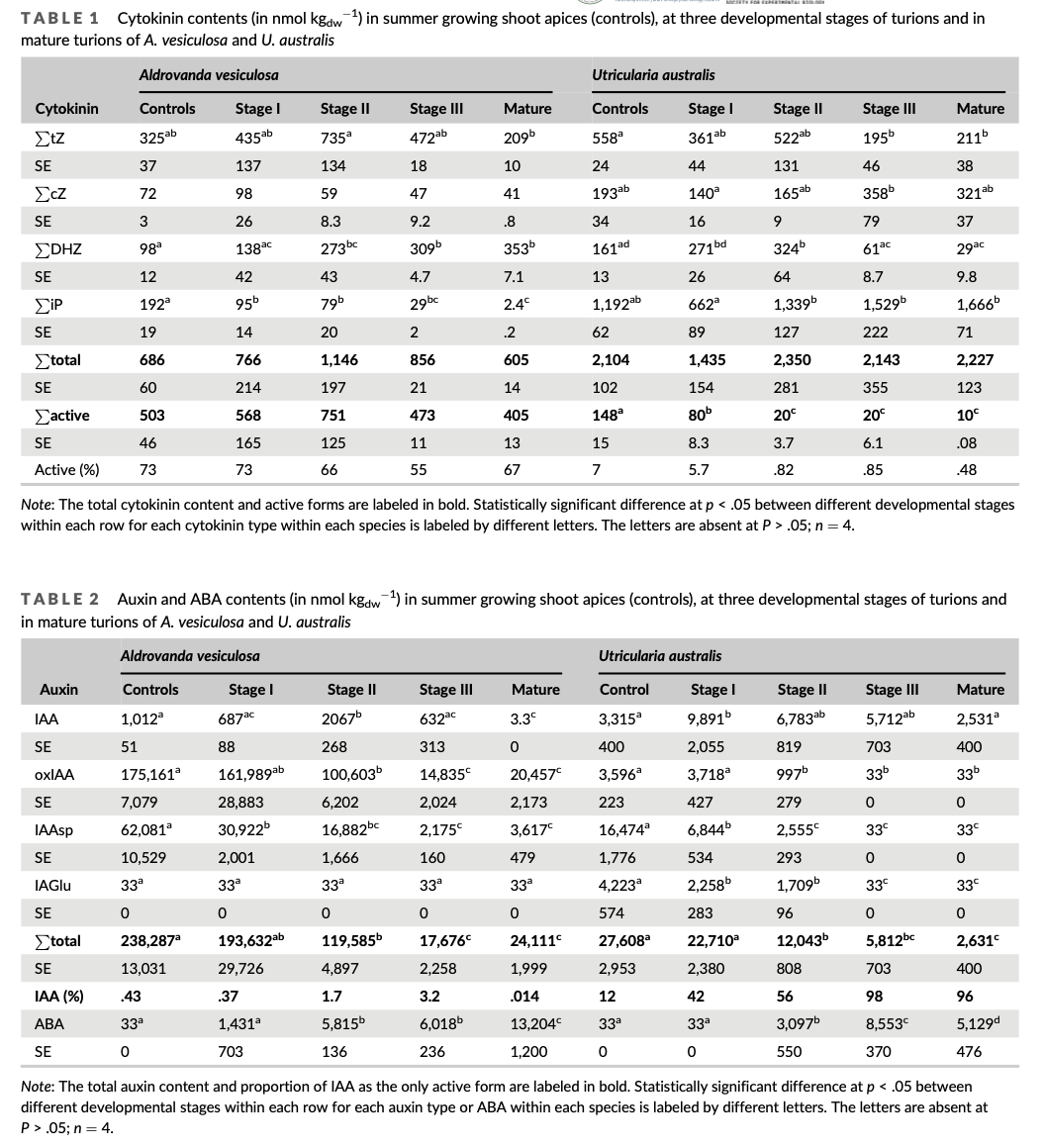


In UA shoot apices during turion development, auxin profiles (both contents and trends) differed considerably from those in AV (Table 2). In UA, IAA and IAAsp were the dominant auxin forms, at least in the growing plants and at Stages I and II. The IAA content peaked significantly at Stage I and was then decreasing back to the level found in summer growing plants in mature turions. Both oxIAA, IAAsp, and IAGlu exhibited the highest contents in summer growing plants, the contents were decreasing steeply during the next three developmental stages so that at Stage III, and in mature turions, their contents were below the limit of detection. The total auxin content decreased about 10 times between summer growing plants and mature turions. Due to the relatively high IAA content in all developmental stages (2,531–9,891 nmol kgdw1) in UA shoot apices, and also due to simultaneous declining of the total auxin con- tent, the IAA proportion to the total auxin content was gradually rising from 12% in summer growing plants to 96–98% at the two last stages of turion development. The ABA content in UA shoot apices was below the limit of detection in summer growing plants and also at Stage I but was greatly increased in all the following developmental stages (3,097–8,553 nmol kgdw1) and culminated at stage III (Table 2).

## **DISCUSSION**

| **Text within Paper** | **Text Color** |
| --- | --- |
| **Restating Trends/Hypotheses** | **GREEN** |
| **Interpretations** | **PURPLE** |
| **In-text Citations** | **ORANGE** |
| **Big Picture** | **RED** |
| **Limitations** | **BLUE** |
| **Future Directions** | **GREY** |

The main aim of the paper was to find changes in phytohormone pro- files in shoot apices of two aquatic carnivorous plants during the gradual transition from the summer growth to mature, innately dormant turions. It should be noted that once a plant is induced by environ- mental factors to form turions, this developmental stage is irreversible by changing the environmental factors and has the character of the all-or-none rule (Adamec, 2018a; Winston & Gorham, 1979a, 1979b). As turions represent quite contrasting organs (both morphologically and physiologically) in comparison to summer growing shoot apices, it can be expected that the transition between both “stable” states shall include profound changes in the profiles of phytohormones such as CKs, auxins, and ABA in the developing shoot apices towards future turions, but also in the remaining, dying annual shoots (Adamec, 2018a; Sˇimura et al., 2016; Winston & Gorham, 1979b). The same conclusion can also be drawn from external applications of ABA or cytokinin kinetin to some aquatic plants (mainly Spirodela polyrhiza) to induce turion formation or to revert it (Chaloupková & Smart, 1994; Smart et al., 1995; Smart & Trewavas, 1983; Weber & Noodén, 1976) and also from first genomic or transcriptomic studies on turions (Li et al., 2022; Pasaribu et al., 2023; Wang et al., 2014).

We have chosen three discrete arbitrary stages of immature tur- ion development (Figure 1 and Table S1) in order to subdivide the gradual turion development into three separate periods and/or processes. These stages include the gradual switch from the summer unterminated, continuous shoot growth to shoot growth cessation and are characterized by a marked and gradual rise in the DMC in the developing turions. The shortest period between two successive stages was 9 days; this might be long enough to expect marked changes both in young trap-free shoots/leaves (metabolic processes) and in phytohormone profiles in developing turions. Although the chosen three stages were comparable morphologically in both species, there was a great difference in the cessation of growth of new leaf nodes between both species: in A. vesiculosa, the growth ceased as early as at Stage I, while it continued partly even at Stage II in U. australis.

PN and RD in young shoots/leaves were the highest in summer growing plants and at stage I in both species (Figure 2a,b), and the values of both parameters were comparable with those measured in both species in summer growing plants (cf. Adamec, 1997, 2013). Afterwards, PN, RD, and also chl. a + b content decreased more steeply in UA than in AV. A quite opposite pattern of tissue N and P content in senescing leaves/shoots between both species (Figure 3) indicates a very low N and P reutilization efficiency in AV as com- pared to that in UA during turion development, although Adamec (2000) suggested a very effective N and P reutilization in AV during turion development. The relatively high PN and RD values in young leaves of both species during all stages of turion maturation may con- firm that supply of carbohydrates as future reserve substances to maturating turions from photosynthesis of young leaves/shoots is much more important than reutilization of sugars from senescing shoots and that young leaves are still metabolically very active, even at the last stages of turion development without any new leaf growth (cf. Adamec, 1997, 2000, 2013). In AV, there is no apparent relationship between the time-course of PN, RD, pigment, and N and P con- tent in young shoots and the turion content of the inhibitory ABA. While in young UA leaves, the time-course of photosynthetic pigments and PN and RD agreed with that of the turion ABA content.

Numerous reports describe the role of phytohormones in plant development (Davies, 2004); however, cytokinins and auxins in storage organs (except for roots) have been studied only a few times even in land plants; to our knowledge, no studies on these phytohormones in storage organs in aquatic plants have been published so far. Kara et al. (1997) studied the distribution of phytohormones in the roots of radish plants and concluded that both IAA and CKs were involved in the initiation and formation of the storage organ (regardless of light quality). Moreover, CKs appeared also to stimulate the assimilate flow to developing storage tissues (Kara et al., 1997). Similar results were obtained for changes in endogenous levels during the growth cycle of Curcuma alismatifolia (Hongpakdee et al., 2010). Off-season conditions induced a decrease in the photosynthetic rate and increases in ABA and tZR contents in various organs at different growth stages, leading to depressed shoot growth and an increase in rhizome numbers.

Our results confirmed a mild, transient increase of the tZ type and of total and active CKs in the middle of turion development in AV. In UA turions, the contents of all active CK forms gradually but markedly decreased though the total content was nearly constant (Table 1). It is evident that developing and maturating turions in aquatic plants represent organs possessing a very strong sink for both mineral nutrients and organic reserve substances, similar to seeds (e.g., Gonzalez-Lemes et al., 2023; Liang et al., 2023). In line with this, it is well known that CKs function in the regulation of sink–source relationships, both in source and sink organs, and in the activation of the genetic program of development (Ron’zhina, 2009). They stimulate the source function of leaves by stimulating leaf expansion, increasing net photosynthesis and by changing the balance between transportable and storage forms of photoassimilates (increase of sucrose and reduction of starch synthesis). Moreover, CKs also stimulate the sink strength and the activity and incorporation of soluble organic substances into insoluble polymeric compounds (starch, structural polysaccharides, and proteins).

A number of studies have confirmed that IAA content is extremely important for plant development and is influenced by the rate of its biosynthesis, transport, and metabolic inactivation (Kondhare et al., 2021). In several tuber and storage-root crops, a common trend has been observed: IAA contents in tubers or developing storage roots are high during the early developmental stages of the belowground storage organs so that they could induce cell divisions. However, IAA contents drop during the later stages of development suggesting that high IAA content is essential only for the onset of storage organ formation, whereas its low content is required during the late processes of storage organ thickening (Kondhare et al., 2021). Our results confirmed a very similar trend in aquatic carnivorous plants: the content of free IAA as the only active auxin form is increasing gradually during turion development up to a certain stage, reaching the peak contents at Stage II in AV and at Stages I and II in UA, and then dropping in mature turions (in AV down to zero, Table 2). On the other hand, the contents of inactive auxin metabolites are decreasing consistently in both species during turion development. However, this is the first report on the role of auxins in turion development and should be confirmed by subsequent studies.

The crucial role of another phytohormone, ABA, in turion development mainly in S. polyrhiza has been known for a long time (e.g., Li et al., 2022; Smart et al., 1995; Smart & Trewavas, 1983; Wang et al., 2014; Weber & Noodén, 1976; Winston & Gorham, 1979b). Smart et al. (1995) quantified ABA contents in normally growing S. polyrhiza fronds and in those induced to form turions by an exogenous ABA supply. The comparison of these values allowed the authors to judge whether the frond ABA content associated with turion induction after the ABA supply could be attained by an endogenous syn- thesis and accumulation of ABA. However, the susceptibility of turion induction by an ABA supply may be rather low in more robust aquatic plants (cf. Best, 1979; Weber & Noodén, 1976). In a novel transcriptomic study on S. polyrhiza turions formed after ABA treatment, Wang et al. (2014) confirmed profound changes in gene expression in tur- ions. In turions, a total of 208 genes exhibited four times more increased expression as compared to growing fronds, while 154 genes exhibited markedly reduced expression. In this species, turion formation thus represents very complex developmental changes on the genomic level comparable, e.g., with flowering (see also Pasaribu et al., 2023).

The formation of bulbils, considered another type of storage organ, is an important agronomic trait also found in yams. In a land- race of water yam (Dioscorea alata), which rarely forms bulbils, Hamaoka et al. (2023) investigated the effect of ABA on bulbil formation on the basis of changes in the sink–source relationships in response to a waterlogging stress. ABA treatment of leaf axils enhanced bulbil formation in unstressed plants, suggesting that increased ABA content is one of the factors that initiate bulbil formation. Also in our study, the ABA content increased from almost zero by 2 orders of magnitude during turion development and culminated in nearly mature or mature turions in both species (Table 2), thus confirming the central role of ABA in turion development and maintaining of the innate dormancy. In developing turions in UA at Stage I though, the ABA content was still nearly zero (Figure 1 and Table 2). It may indicate that increase of endogenous ABA content is not regulated by the turion induction alone but can occur at more advanced developmental stages (Stage II) before the growth of new leaves has ceased.

Overall, the data obtained in our study fully confirmed the importance of three phytohormone classes (ABA, auxins, and CKs) in the regulation of developmental stages of turions as overwintering and storage organs in two unrelated species of aquatic carnivorous plants. However, our results based on analyzing the endogenous con- tents of the phytohormones in developing turions should be con- firmed by future studies based, for example, on exogenous treatment by phytohormones or comparing the hormone profiles in true dormant turions and non-dormant winter shoot apices in some relative species or elucidating the hormonal cross-talk between gradually developing turions and senescing leafy shoots. These further experiments should confirm, for example, the role of cytokinins and other plant hormones in regulation of sink-source relationships during tur- ion development as well as their crosstalk, as these mechanisms were previously studied only in land but not yet aquatic plants. As Aldrovanda and Utricularia spp. shoots exhibit a marked physiological and growth polarity and their growing shoot apices represent a strong sink for N, P and organic substances (Adamec, 2018b), one can also ask whether the sink–source relationships in developing tur- ions are the same as those in growing shoots.
