# Supplementary material for: Introducing a Novel Course-Based Undergraduate Research Experience Using Duckweed as a Model System
Source: Integr Org Biol. 2025 Dec 19;8(1):obaf049. doi: 10.1093/iob/obaf049 (PMC12802901; doi:10.1093/iob/obaf049)
Supplement: obaf049_Supplemental_Files [file obaf049_supplemental_files.zip › 07 Supplementary Materials/Supplementary Materials/44_Week09_THA_DevelopingResultsAndDiscussion.docx]

# THA: Develop Results & Discussion

# (submit one per student; independent)

You can consult with your lab partner for this ICA, but each of your responses should be independently developed. Your captions can be the same. Use the Writing Guide under Resources on Moodle to help you complete this document.

## Results

*Based on feedback from ICA: Data Analysis, include a revised caption and trend sentence/s for each of your figures. These should be in paragraph form. The final portion of your results paragraphs will include statements and comparisons of important values, as well as significance. You can bullet these or attempt a first version paragraph.*

#### *Choose a graph that best represents percent coverage*

Copy your line graph:

Revised caption for this figure:

What are the general trends for this figure (1-2 sentences)?

What are some important values and comparisons that should be made in your paper? Include significance.

#### *Choose a graph that best represents frond growth*

Copy your bar graph of means:

Revised caption for this figure:

What are the general trends for this figure (1-2 sentences)?

What are some important values and comparisons that should be made in your paper? Include significance.

## Discussion

*Compile the information that will go into your discussion section. Discuss with your lab partner but be sure that all content is independent and your intellectual property. Leniency will not be given for plagiarism, even if unintended.*

Restate hypotheses & predictions.

- Null:
- Alternative:
- Prediction/s:

Discuss support or rejection of each hypothesis (recall rules about not “supporting” the null)?

- Percent Coverage:
- Number of Fronds:

Interpret your data – explain the WHY of your findings – using common language (not technical like in results).

What are some limitations of your study? What other things could be influencing your data? See slide from PPT.

What is the big picture of your study? Why should someone outside of the classroom care?

What are the real-world applications of your study?

If you were to take the results from your study and expand upon them, how would you do so? Note: think critically; additional replicates or more light intensity values will not suffice here.
