# Supplementary material for: Introducing a Novel Course-Based Undergraduate Research Experience Using Duckweed as a Model System
Source: Integr Org Biol. 2025 Dec 19;8(1):obaf049. doi: 10.1093/iob/obaf049 (PMC12802901; doi:10.1093/iob/obaf049)
Supplement: obaf049_Supplemental_Files [file obaf049_supplemental_files.zip › 07 Supplementary Materials/Supplementary Materials/45_Week10_PROTOCOL_LakeStudy.docx]

# **Protocol: Lake Study**

**Read carefully:**

LSU EHS requires full PPE (person protection equipment) when working with LSU lake water, as it can and does contain pathogens that could make you ill. Full PPE for this lab includes gloves, goggles, surgical grade face coverings, and laboratory coats. While handling your collection containers and slides, ensure that you are not touching your face, phone, pen/pencil, etc. Re-sanitize your gloves regularly by squirting 70% EtOH into your gloves and allowing them to dry. Do this especially if your gloves come in contact with the lake water.

After observing your specimens, follow the clean-up procedure listed below. Remove your gloves safely to avoid contamination and dispose of them in the biohazard bag. By participating in this lab, you are agreeing to everything outlined above. Should you handle lake water without proper PPE, you will be asked to leave the lab.

## **Stereoscope use**

1. **Before you begin, wipe contact areas (ocular lenses, nosepiece, and focus knobs) with an alcohol pad.**
2. Make sure the cap is secure on your bottle and gently agitate (shake) your sample.
3. Pour a small amount of your sample into a petri dish and place on the stereoscope. It is good to get some particulates in your sample, as microorganisms can be found within them.
4. Turn on the light source (back of the scope) and use the light settings on the right side. You can choose different settings for the light to come from above (REFL), below (TRANS, DI, DF), or both (REFL/TRANS, DI/TRANS/REFL).
5. Look through the eyepiece. Notice that ocular lenses are listed as 10x magnification.
6. Use the coarse adjustment knob to focus on the image. You may need to fully rotate to bring it into focus.
7. Use the fine adjustment knob to fully focus your image. You should be able to see a clear image of your specimen.
8. Notice the numbers on the fine adjustment knob range 0.7-4.5. Multiply this # by 10 (magnification from ocular) to get the total magnification. Total magnification should be listed under any images in your presentation.

## **Compound microscope – wet mounts**

1. **Before you begin, wipe contact areas (ocular lenses, nosepiece, and focus knobs) with an alcohol pad.**
2. Ensure microscope is in starting position: stage lowest, 4x objective lens in position.
3. Obtain a glass slide, cover slip, and transfer pipette from the supply bench.
4. Draw-up a specimen from your sample using a transfer pipette and place a drop at the center of the slide.
5. **At an angle**, place the cover slip against the slide over your specimen. Note: the slip is glass and will break.
6. Place the slide on the stage and secure it with the slide holder – the holder secures the slide from the side.
7. Using the x/y knobs, position the slide so that the sample is directly above the stage opening.
8. Turn-on the light source and look through the eyepiece. Adjust the diaphragm to a comfortable light intensity.
9. Use the coarse adjustment knob to move the stage as **high** as it will go (ensure you are on 4x).
10. While looking through the ocular, use the coarse adjustment to move the stage down until image is in focus.
11. Adjust power intensity knob for the best viewing and the fine focus knob to bring it into perfect focus.
12. Ensure that your specimen is directly in the center of your field of view using the x/y knob on the stage.
13. The y and x-axis knobs located below stage can be used to move the slide to search for more specimens.
14. Slowly move up to the next level of magnification: the 10X objective.
15. Adjust the power intensity, fine focus knob, and diaphragm accordingly until it focuses perfectly.
16. **Note:** **Do not use the coarse focus knob when using 10x or 40x objective lens;** it will break the lens and slide.
17. Slowly move up to the next level of magnification, 40X, if desired. Not all samples need to be amplified this much.
18. Adjust the power intensity, fine focus knob, and diaphragm accordingly. **Note: do not use the coarse focus.**
19. Take pictures and videos for your presentation. Be sure to sanitize your gloves before touching your phone.
20. A Freshwater Microorganism Identification Guide can be found at your bench for organism identification.
21. Repeat steps using your or another’s sample.
22. **Note:** Glass slides and cover slips can be wiped & re-used to view different samples.

## **Clean up:**

- Squirt bleach into your sample and pour water down sink (**ensure plastic screen is in place over drain**).
- Specific discard container:
  - transfer pipette into labeled discard container
  - glass coverslip in the SHARPS DISCARD container at bench
  - wipe your slide with a paper towel and place into the ethanol bath for sterilization
- Regular trash:
  - particulates from sample (collect into paper towel from sink if needed)
  - collection tube/bottle
  - face mask (remove gloves first – see below)
- Dispose of gloves in the biohazard bag. Wash hands and wrists thoroughly.
- Wipe goggles with alcohol pads before returning to the goggles bin
- Adjust objective lens to the lowest magnification and the stage at its lowest setting & switch off the microscopes.
