# Supplementary material for: Introducing a Novel Course-Based Undergraduate Research Experience Using Duckweed as a Model System
Source: Integr Org Biol. 2025 Dec 19;8(1):obaf049. doi: 10.1093/iob/obaf049 (PMC12802901; doi:10.1093/iob/obaf049)
Supplement: obaf049_Supplemental_Files [file obaf049_supplemental_files.zip › 07 Supplementary Materials/Supplementary Materials/46_Week10_ICA_PrintoutForLakeSamplingObservations.docx]

# **Field Study: Lake Sampling & Observations**

## Part I. Water sample collection

1. Obtain gloves to wear for collection. Be mindful not to touch your face or phone during Collection.
2. Choose a spot along the edge of the lake that is accessible to collect water.
   1. SPREAD OUT FROM YOUR CLASSMATES!
3. Dip your bottle to fill halfway with water + vegetative particulates. The critters live in the particulates!
4. Secure the lid to your collection container and place into the secondary container (e.g. plastic bag).
5. Remove your gloves properly to avoid contamination (video shown in class) & dispose with your instructor.
6. Sterilize your hands and wrists with ethanol spray bottle – water can harbor pathogenic bacteria and viruses!
7. When able to access a bathroom, wash hands and wrists thoroughly.

## Part II. Observations

- Take at least three quality pictures of your collection site - you will use these in your Oral Report.
  - Ex: entire ecosystem pano, collection site, nearby influences (plants, parking lot, pipes, pollution sources)
- Describe the ecosystem you chose (e.g. location, brief description).
- Create two four-member food chains using words and arrows
  - *Ex: hydrilla --> grass carp --> largemouth bass --> great blue heron --> red-tailed hawk*
  - Be specific about the species. Simply saying carp, bass, or hawk is insufficient.
  - If you are unable to observe a full food chain yourself, begin by identifying an organism you see and developing the food chain using Google. Be sure that the organisms you find are local to your area and that you are using credible sources.
- Note how two biotic, two abiotic, and two human activities could influence your collection site. Focus on your collection site, but you can also discuss your larger ecosystem if relevant. The discussion can be bulleted but should be descriptive and connect your organisms to the factors discussed. Possible influencing factors:
  - Depth of water
  - Sunlight availability
  - Shade from trees/bushes
  - Aquatic vegetation: floating, submerged, rooted beneath water, growing along bank and outward, etc
  - Connected to another water body or ditch
  - Nearby parking lot, building, other urban source
  - Turbidity – cloudiness; how much sediment is in water column

## Part III. Presentation

- Why is sampling microorganisms from water bodies important? (1 slide)
  - Who does this and why?
  - What sort of information does the diversity of aquatic organisms provide?
- Collection site (~2 slides)
  - Pictures of where samples were collected (1 per unique location)
  - Food chains (1 per student)
  - Biotic, abiotic influences (4 each)
  - Human influences (3)
- Specimens (1 slide per student):
  - Pictures/videos of specimens observed in class
  - Describe specimens: autotroph/heterotroph; magnification; size; behavior; etc.
  - Relate to your collection site – did environmental characteristics influence what you found?
