# Supplementary material for: Introducing a Novel Course-Based Undergraduate Research Experience Using Duckweed as a Model System
Source: Integr Org Biol. 2025 Dec 19;8(1):obaf049. doi: 10.1093/iob/obaf049 (PMC12802901; doi:10.1093/iob/obaf049)
Supplement: obaf049_Supplemental_Files [file obaf049_supplemental_files.zip › 07 Supplementary Materials/Supplementary Materials/47_Week10_ICA_LakeStudyAlternativeAssignment.docx]

ICA: Lake Sampling and Observations Alternative Assignment

1. Describe abiotic, biotic, and human influences (two each).
2. Discuss your thoughts on the health of this system, based on the influences listed above as well as the organisms found. Remember that one sign of a healthy system is diversity of organisms in a sample.
3. Why is the health of an aquatic ecosystem important to humans?


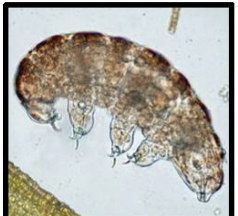

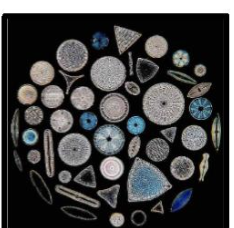

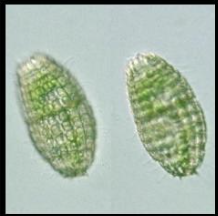


|  |  |  |  |
| --- | --- | --- | --- |
| Ocular lens mag | 10x | 10x | 10x |
| Objective lens mag | 40x | 10x | 4x |
| Total Magnification |  |  |  |
| Organism ID |  |  |  |
| Organism color |  |  |  |
| Motile or non-motile? |  |  |  |
| Heterotrophic or autotrophic? |  |  |  |
| Organism’s interesting characteristics |  |  |  |
