# Supplementary material for: Introducing a Novel Course-Based Undergraduate Research Experience Using Duckweed as a Model System
Source: Integr Org Biol. 2025 Dec 19;8(1):obaf049. doi: 10.1093/iob/obaf049 (PMC12802901; doi:10.1093/iob/obaf049)
Supplement: obaf049_Supplemental_Files [file obaf049_supplemental_files.zip › 07 Supplementary Materials/Supplementary Materials/48_Week11_FORMS_PosterPartnerContract.docx]

# **CURE Poster - Partner Contract**

You will work with your bench mates to develop a scientific poster for your CURE study. When designing and developing your poster, be sure to choose the best pieces and parts from each partner’s paper, rather than working independently. Your poster, along with delivery, will be worth 15% of your total grade in this course. Use all available resources to build a great poster.

*To work with lab partners on these assignments, you must agree to the following*:

- Lab partners must contribute equally to poster development.
- A document of partner contributions must be used to track each partner’s contribution.
  - This will be submitted with your poster file.
- Lab partners are equally responsible for submitted work.
- You must communicate with your lab partner regularly and promptly.
  - You must respond to communication from your lab partner within 6 hours.
- Partners may not work together on any other assignment.
- Partners that do not contribute equally to the development of the poster may lose the privilege of delivering the poster and be asked to develop a poster independently.

By typing your name below, you acknowledge that you understand and will adhere to the policies listed above for working with your lab partner. If you fail to adhere to these guidelines, the instructor will direct you to independently develop a poster for submission. Should you choose to work alone for your poster, you can indicate that below and submit this contract independently.

*Partners*:

This document must be electronically signed (name and date typed in the fields) and submitted through the Poster Partner Contract Turnitin link on Moodle before you leave class**.**

|  |  |  |
| --- | --- | --- |
| Name (electronic signature) |  | Date |
