# Supplementary material for: Introducing a Novel Course-Based Undergraduate Research Experience Using Duckweed as a Model System
Source: Integr Org Biol. 2025 Dec 19;8(1):obaf049. doi: 10.1093/iob/obaf049 (PMC12802901; doi:10.1093/iob/obaf049)
Supplement: obaf049_Supplemental_Files [file obaf049_supplemental_files.zip › 07 Supplementary Materials/Supplementary Materials/49_Week11_RESOURCES_PosterTips.docx]

**CURE POSTER TIPS**

**Organization:**

- Poster must be landscape orientation and set to a custom slide size (Width 48”, Height 36”)
- Poster will be in 3 columns – use templates
- Make sure all columns are ALIGNED - will make the poster look much more visually pleasing
- Must have white space between text boxes, tables, diagrams, and border of poster - otherwise will look cramped
- Separate each section (abstract, introduction, methods, etc) into text boxes so each can stand out on its own

**Overall:**

- After complete, make sure your poster will open properly (maintains formatting) on another computer
- Don’t include too much information on poster - it can be visually overwhelming
- Remember this poster is not to stress you out! You all have done the work for this through formal writings. Simply summarize your work and put it into a big poster. IT’S NOT AS INTIMIDATING AS IT SEEMS.
- Plenty of example templates available online to give you poster ideas

**Images & Figures:**

- Introduction: consider using a picture of your model organism
- Methodology:
  - use pictures of you doing wet labs or set-up of study
  - consider creating a flowchart to explain the flow of methods or visual to explain more technical methods
- Results:
  - mainly composed of figures with little text (more below)
  - include picture of final product if appropriate

**Text:**

- Make sure your text is easy to read!! If people are having trouble with it, they will be less inclined to read your poster.
- Title: >75 pt (should be seen from 4-5 ft away)
- Headings: 30-60 pt
- Text: 18-24 pt
- Don’t use a variety of fonts. Keep it down to max 2-3 fonts.
- Don’t cram too much information into a small space.

**Color:**

- Don’t use too many colors doesn’t reflect organization (3 colors should be max)
- The most important tip: make sure colors are compatible! (color wheel: opposites work well)
- The first thing that will draw a person to your poster is if it’s visually pleasing
- There are plenty of online resources that can help you find compatible color schemes
- Background should be white or light color
- If background is dark, make sure text is light

**Title:**

- Should be the biggest text on poster
- Be able to grab audience’s attention
- As succinct as possible but specific at the same time
- Remember to italicize names of microbes

**Abstract (optional):**

- 150-200 words
- Should be directly below title
- Include research problem (the “why”), methods (“how”), results, and implications for the future
- SHOULD BRIEFLY ANSWER WHY, WHAT, HOW
- Do not use bullet points/it’s usually written in paragraph form

**Introduction:**

- The main reason for an introduction is to answer “why did I do this experiment” or why is this study important
- Below abstract
- Can use bullet points of fragmented sentences (avoid full sentences for bullets)
- Remember to cite your sources in this section because this is where you will talk about the majority of your sources

**Methods**:

- Flowchart/Visual can be used to summarize the methods (SUGGESTED!)
- Can be directly under introduction or can be the start to the second column
- Bullet list/numbered list
- Include controls (+/-), independent/dependent variables

**Results**:

- Probably largest section of poster
- MUST INCLUDE CHARTS/GRAPHS/DIAGRAMS – most important data
- All tables and figures must have captions
- two-four figures
- Should mostly be image-based; try to use very few words
- Summarize the most important data

**Discussion**:

- Placed in third column
- Bulleted list
- “take home” points/what did I learn from this experiment
- Also answers the “why” did this happen
- Explain the meaning of the results
- Elaborate on any unanticipated outcomes
- Must include applications to real world
- Future applications
- How research on this can be further improved

**References**:

- Below discussions
- Depends on TA which style of referencing should be used but for science, APA is the go-to
- Usually smaller font that the rest of the text

**Acknowledgements**:

- LSU College of Science
- Department of Biological Sciences
- Your instructor
- Mindy Brooks
- Additional resources, as advised by your instructor
