# Supplementary material for: Introducing a Novel Course-Based Undergraduate Research Experience Using Duckweed as a Model System
Source: Integr Org Biol. 2025 Dec 19;8(1):obaf049. doi: 10.1093/iob/obaf049 (PMC12802901; doi:10.1093/iob/obaf049)
Supplement: obaf049_Supplemental_Files [file obaf049_supplemental_files.zip › 07 Supplementary Materials/Supplementary Materials/50_Week11_RESOURCES_PosterTemplatesStandardSize.pptx]

## Slide 1
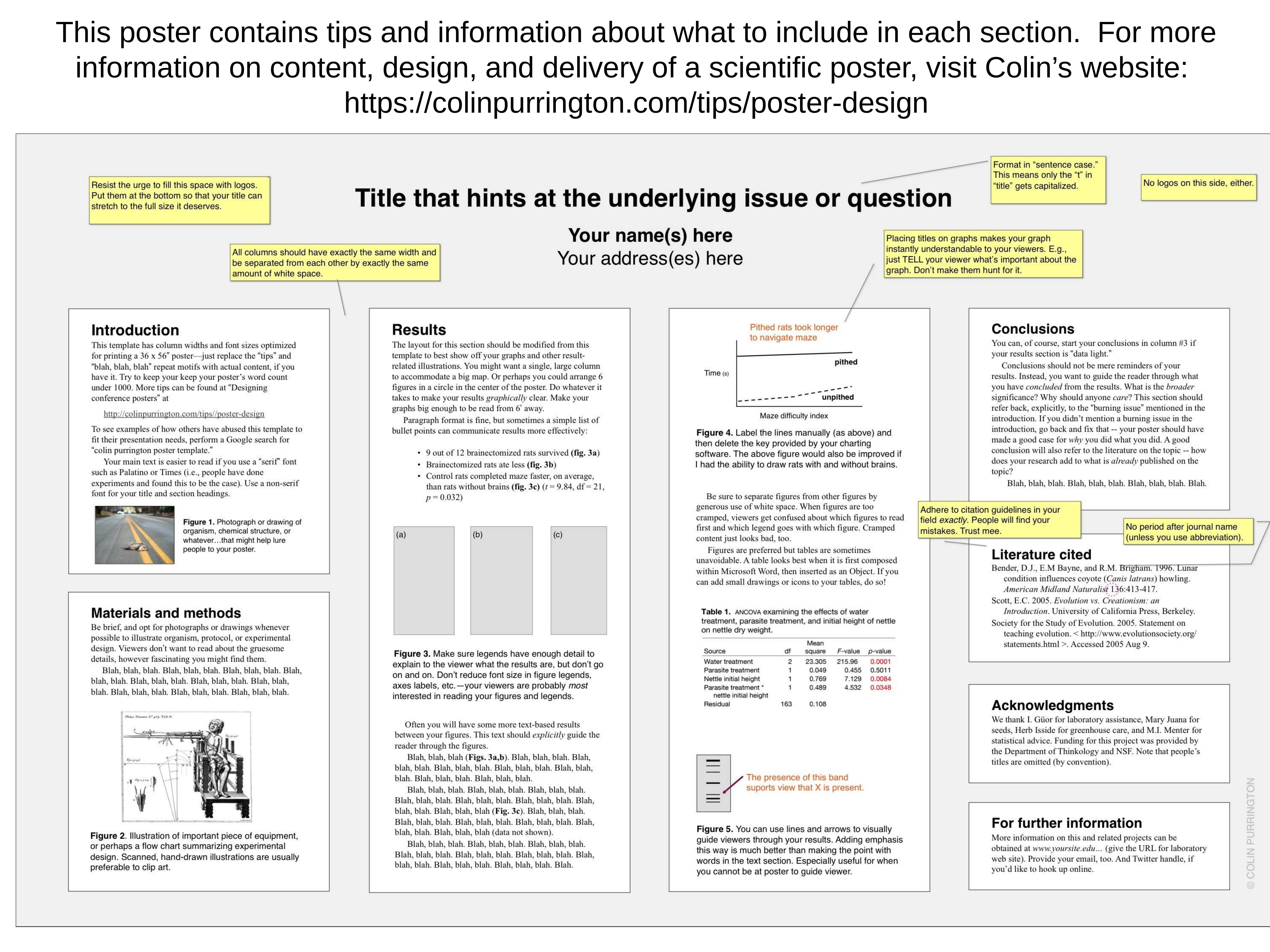

This poster contains tips and information about what to include in each section. For more information on content, design, and delivery of a scientific poster, visit Colin’s website:
https://colinpurrington.com/tips/poster-design

## Slide 2
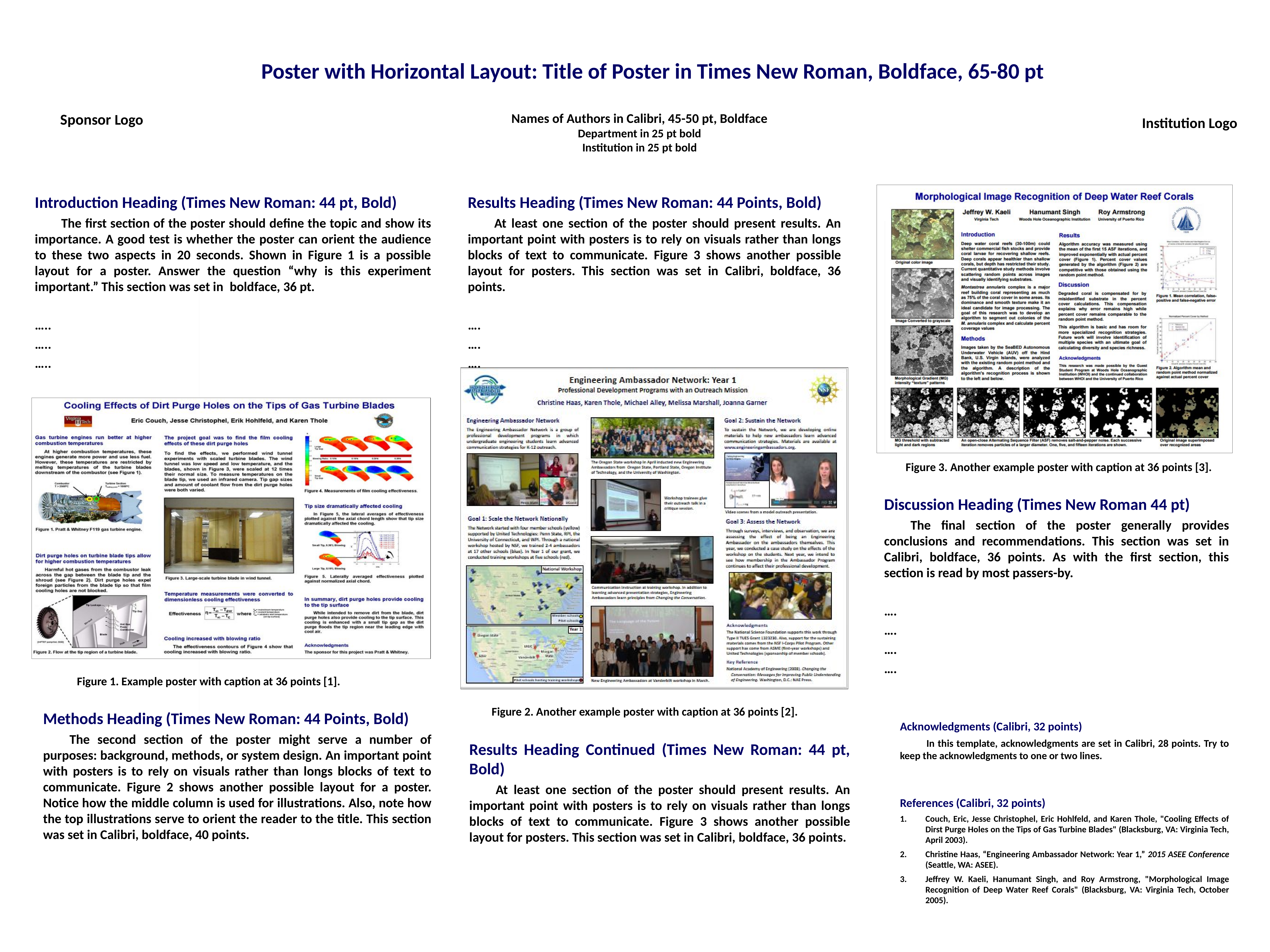

Poster with Horizontal Layout: Title of Poster in Times New Roman, Boldface, 65-80 pt
Sponsor Logo
Names of Authors in Calibri, 45-50 pt, Boldface
Department in 25 pt bold
Institution in 25 pt bold
Institution Logo
Introduction Heading (Times New Roman: 44 pt, Bold)
	The first section of the poster should define the topic and show its importance. A good test is whether the poster can orient the audience to these two aspects in 20 seconds. Shown in Figure 1 is a possible layout for a poster. Answer the question “why is this experiment important.” This section was set in  boldface, 36 pt.
…..
…..
…..
Results Heading (Times New Roman: 44 Points, Bold)
	At least one section of the poster should present results. An important point with posters is to rely on visuals rather than longs blocks of text to communicate. Figure 3 shows another possible layout for posters. This section was set in Calibri, boldface, 36 points.
….
….
….
Figure 3. Another example poster with caption at 36 points [3].
Discussion Heading (Times New Roman 44 pt)
	The final section of the poster generally provides conclusions and recommendations. This section was set in Calibri, boldface, 36 points. As with the first section, this section is read by most passers-by.
….
….
….
….
Figure 1. Example poster with caption at 36 points [1].
Figure 2. Another example poster with caption at 36 points [2].
Methods Heading (Times New Roman: 44 Points, Bold)
	The second section of the poster might serve a number of purposes: background, methods, or system design. An important point with posters is to rely on visuals rather than longs blocks of text to communicate. Figure 2 shows another possible layout for a poster. Notice how the middle column is used for illustrations. Also, note how the top illustrations serve to orient the reader to the title. This section was set in Calibri, boldface, 40 points.
Acknowledgments (Calibri, 32 points)
	In this template, acknowledgments are set in Calibri, 28 points. Try to keep the acknowledgments to one or two lines.
Results Heading Continued (Times New Roman: 44 pt, Bold)
	At least one section of the poster should present results. An important point with posters is to rely on visuals rather than longs blocks of text to communicate. Figure 3 shows another possible layout for posters. This section was set in Calibri, boldface, 36 points.
References (Calibri, 32 points)
Couch, Eric, Jesse Christophel, Eric Hohlfeld, and Karen Thole, "Cooling Effects of Dirst Purge Holes on the Tips of Gas Turbine Blades" (Blacksburg, VA: Virginia Tech, April 2003).
Christine Haas, “Engineering Ambassador Network: Year 1,” 2015 ASEE Conference (Seattle, WA: ASEE).
3.	Jeffrey W. Kaeli, Hanumant Singh, and Roy Armstrong, "Morphological Image Recognition of Deep Water Reef Corals" (Blacksburg, VA: Virginia Tech, October 2005).

## Slide 3
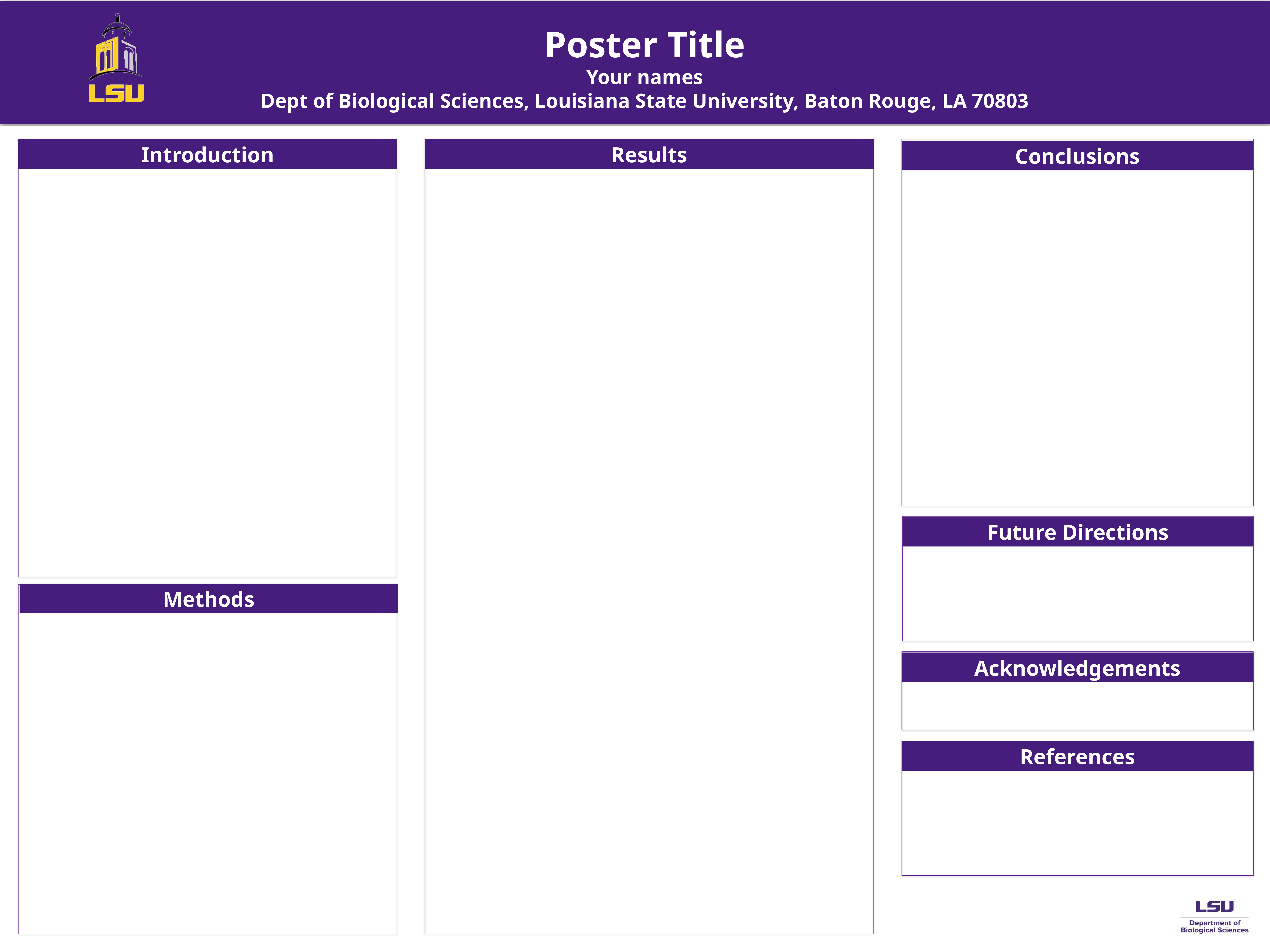

Poster Title
Your names
Dept of Biological Sciences, Louisiana State University, Baton Rouge, LA 70803
Introduction
Results
Conclusions
Future Directions
Methods
Acknowledgements
References

## Slide 4
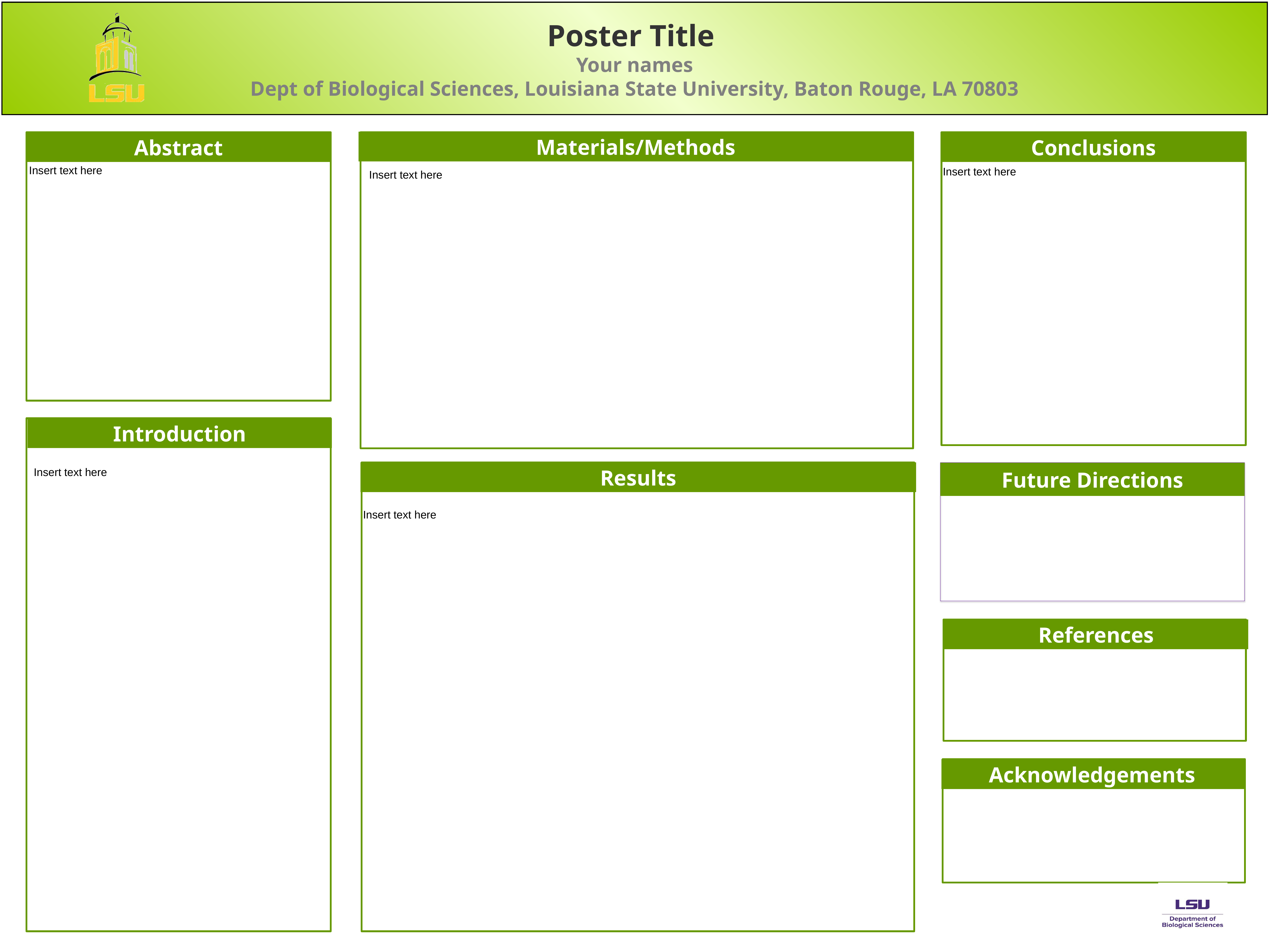

Poster Title
Your names
Dept of Biological Sciences, Louisiana State University, Baton Rouge, LA 70803
Materials/Methods
Abstract
Conclusions
Insert text here
Insert text here
Insert text here
Introduction
Results
Future Directions
Insert text here
Insert text here
References
Acknowledgements

## Slide 5
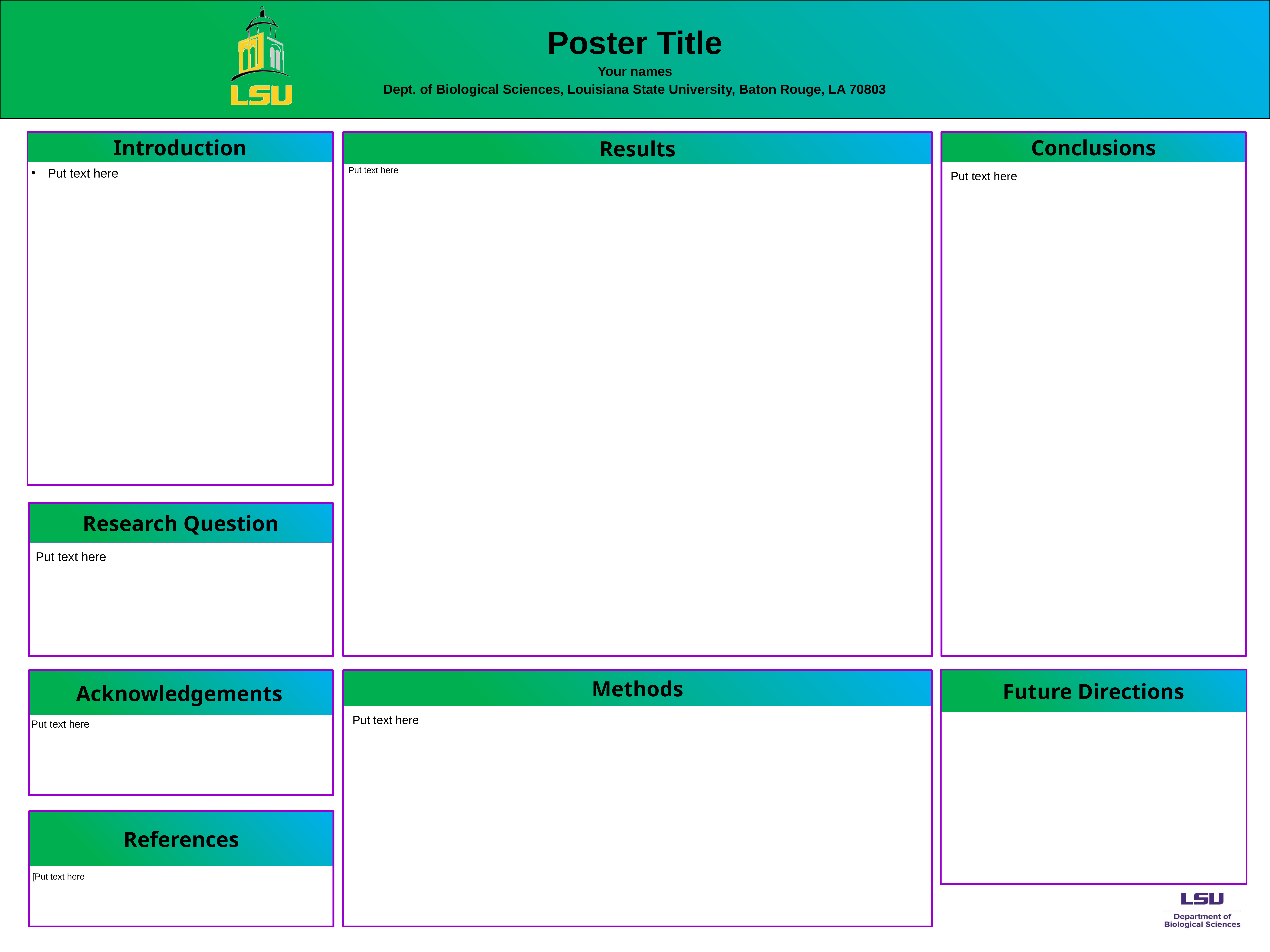

Poster Title
Your names
Dept. of Biological Sciences, Louisiana State University, Baton Rouge, LA 70803
Results
Introduction
Conclusions
Put text here
Put text here
Put text here
B.
A.
D.
E.
Research Question
Put text here
Future Directions
Methods
Acknowledgements
Put text here
Put text here
References
[Put text here

## Slide 6
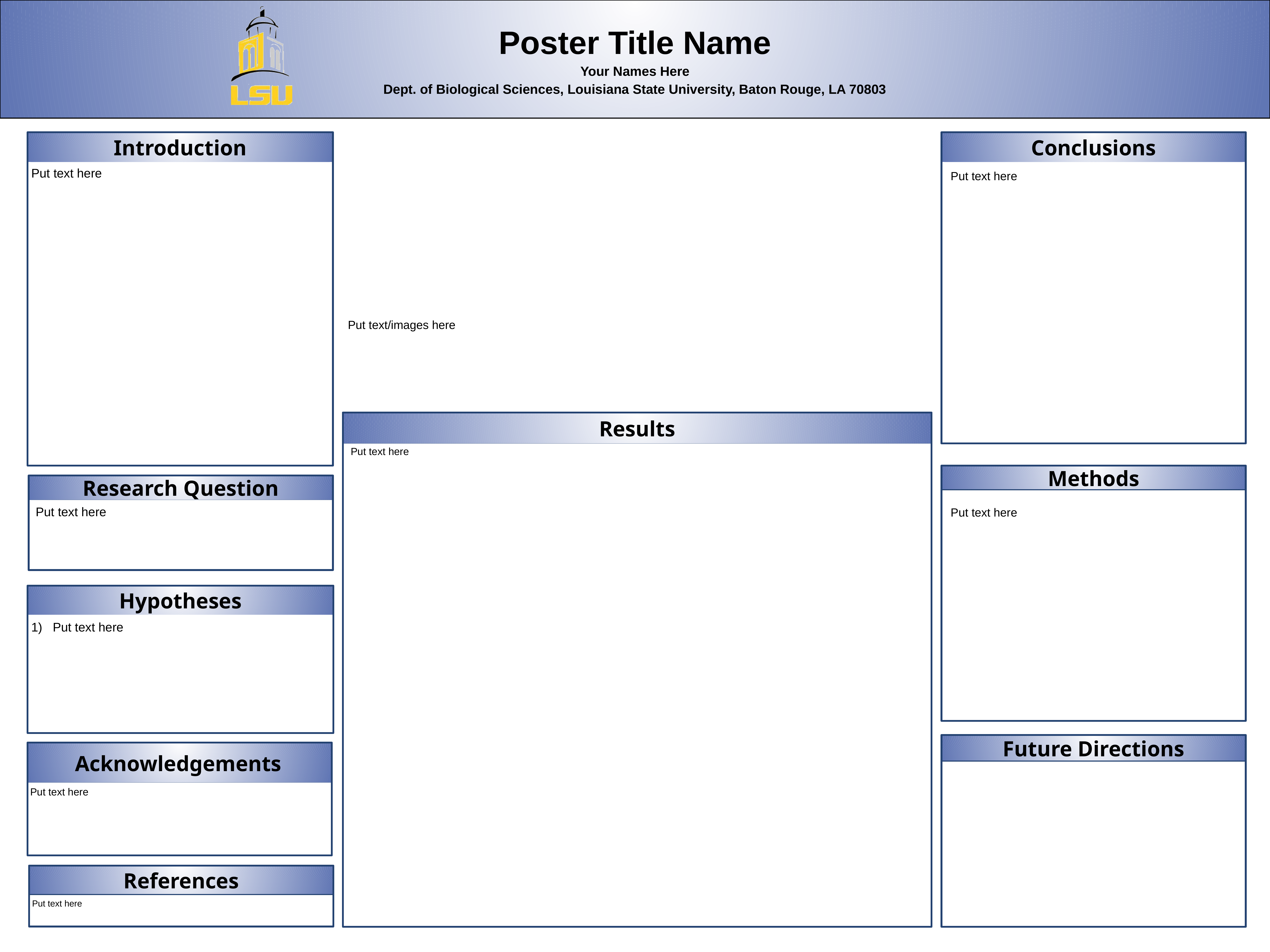

Poster Title Name
Your Names Here
Dept. of Biological Sciences, Louisiana State University, Baton Rouge, LA 70803
Introduction
Conclusions
Put text here
Put text here
B.
A.
D.
Put text/images here
E.
Results
Put text here
Methods
Research Question
Put text here
Put text here
Hypotheses
Put text here
Future Directions
Acknowledgements
Put text here
References
Put text here

## Slide 7
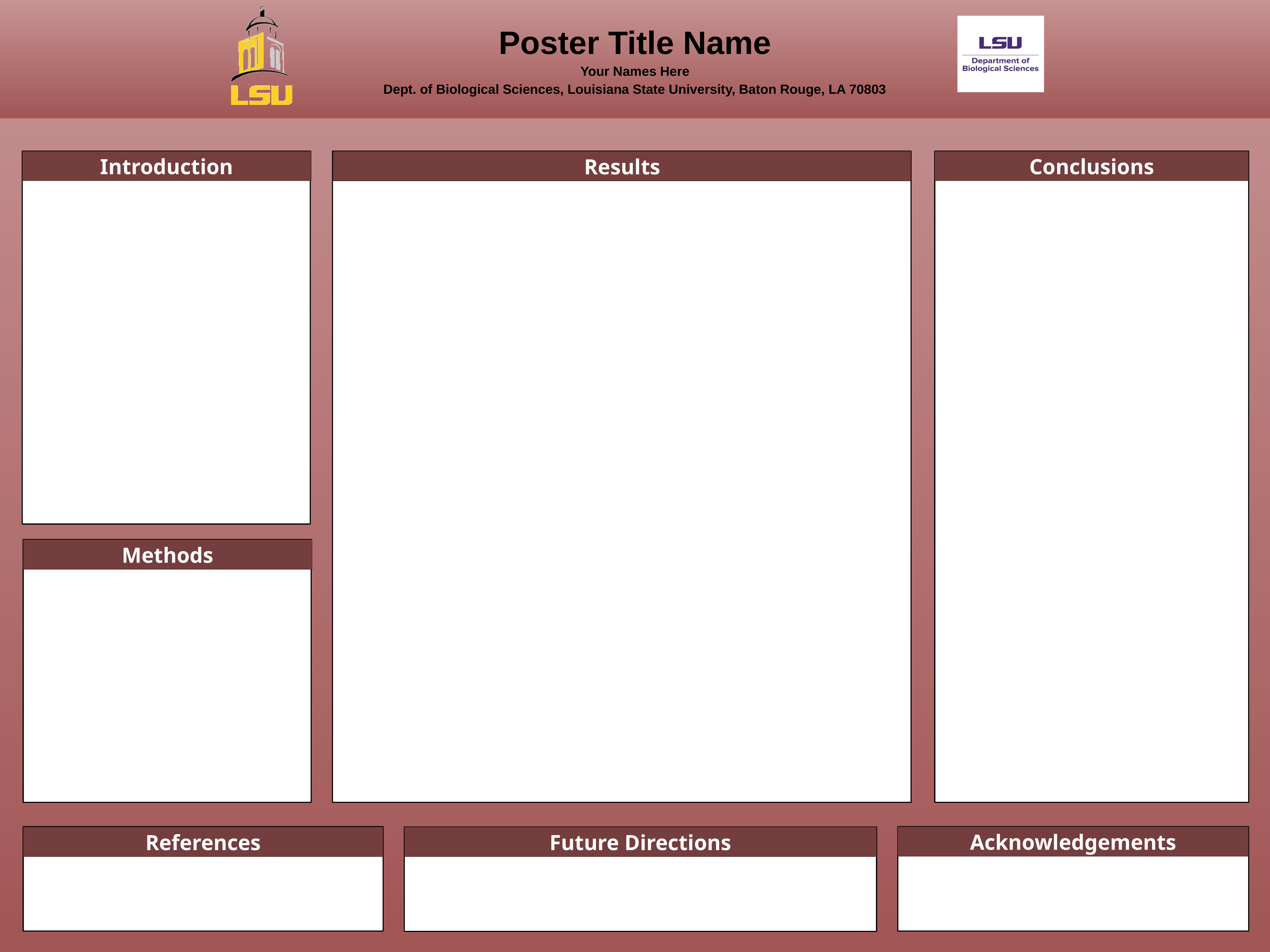

Poster Title Name
Your Names Here
Dept. of Biological Sciences, Louisiana State University, Baton Rouge, LA 70803
Introduction
Conclusions
Results
Methods
Acknowledgements
References
Future Directions

## Slide 8
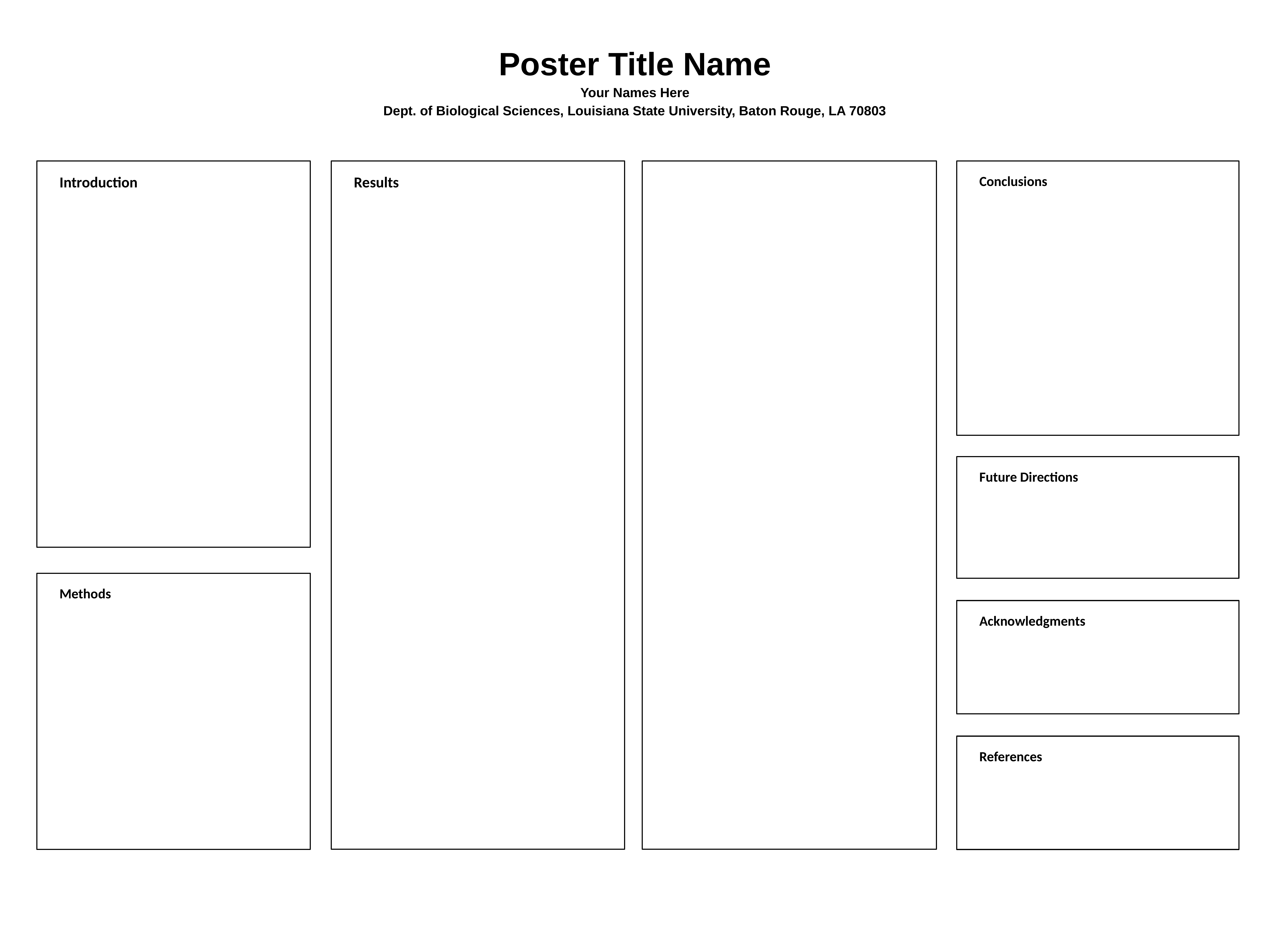

Poster Title Name
Your Names Here
Dept. of Biological Sciences, Louisiana State University, Baton Rouge, LA 70803
Introduction
Results
Conclusions
Future Directions
Methods
Acknowledgments
References
