# Supplementary material for: Introducing a Novel Course-Based Undergraduate Research Experience Using Duckweed as a Model System
Source: Integr Org Biol. 2025 Dec 19;8(1):obaf049. doi: 10.1093/iob/obaf049 (PMC12802901; doi:10.1093/iob/obaf049)
Supplement: obaf049_Supplemental_Files [file obaf049_supplemental_files.zip › 07 Supplementary Materials/Supplementary Materials/51_Week11_ICA_HallwayPosterCritiques.docx]

**Scientific Posters - Hallway Critiques**

*Instructions for reviewer: You will score two posters from the hallways of Life Sciences (floors 2 and up, old or new section). Complete all ratings and include comments for at least half of the categories.*

Poster #1 Title:

Location:

What’s your first impression?

1 – beginner, 2 – needs improvement, 3 – succinct, meets requirements, 4 – mastery

| **Appearance** | **Rating** | **Comments** |
| --- | --- | --- |
| Title is clear, concise, descriptive, and interesting |  |  |
| Poster is well organized and easy to follow; Appropriate headings used |  |  |
| Text readable from an appropriate distance (3-6 feet); Font selection & size appropriate |  |  |
| Graphics relevant & prominent, enhance poster, high quality (i.e. not pixilated) |  |  |
| Balance among text, figures, and white space. Not cluttered |  |  |
| **Content** |  |  |
| Purpose of study is obvious, stated succinctly, and includes the significance of the work |  |  |
| Background is included to place work into larger context of what is known |  |  |
| Key assumptions, objectives, hypotheses stated |  |  |
| Clear and concise description of methods provided |  |  |
| Main result stated outcome of work, address hypothesis, and visualized with graphics |  |  |
| Conclusions stated clearly, supported by results, pertain to current knowledge |  |  |
| Acknowledgements and literature cited included |  |  |
| Free of grammatical errors, unnecessary details, visuals |  |  |

**Additional comments**:

Poster #2 Title:

Location:

What’s your first impression?

1 – beginner, 2 – needs improvement, 3 – succinct, meets requirements, 4 – mastery

| **Appearance** | **Rating** | **Comments** |
| --- | --- | --- |
| Title is clear, concise, descriptive, and interesting |  |  |
| Poster is well organized and easy to follow; Appropriate headings used |  |  |
| Text readable from an appropriate distance (3-6 feet); Font selection & size appropriate |  |  |
| Graphics relevant & prominent, enhance poster, high quality (i.e. not pixilated) |  |  |
| Balance among text, figures, and white space. Not cluttered |  |  |
| **Content** |  |  |
| Purpose of study is obvious, stated succinctly, and includes the significance of the work |  |  |
| Background is included to place work into larger context of what is known |  |  |
| Key assumptions, objectives, hypotheses stated |  |  |
| Clear and concise description of methods provided |  |  |
| Main result stated outcome of work, address hypothesis, and visualized with graphics |  |  |
| Conclusions stated clearly, supported by results, pertain to current knowledge |  |  |
| Acknowledgements and literature cited included |  |  |
| Free of grammatical errors, unnecessary details, visuals |  |  |

**Additional comments**:

**Biggest takeaways to implement for your CURE poster:**
