# Supplementary material for: Introducing a Novel Course-Based Undergraduate Research Experience Using Duckweed as a Model System
Source: Integr Org Biol. 2025 Dec 19;8(1):obaf049. doi: 10.1093/iob/obaf049 (PMC12802901; doi:10.1093/iob/obaf049)
Supplement: obaf049_Supplemental_Files [file obaf049_supplemental_files.zip › 07 Supplementary Materials/Supplementary Materials/52_Week13_ICA_PeerEvaluationsIn-ClassPosterPresentations.docx]

# **Name:**

# **ICA: In-Class Poster Presentations – Peer Evaluations**

## Part I: Evaluate three posters presented in class today. In Part II, you will ask questions of two additional posters (different than the three below).

| **Student names:** |  |  |  |
| --- | --- | --- | --- |
| **Criteria** | **Poster 1 Rating** | **Poster 2 Rating** | **Poster 3 Rating** |
| **Organization**: Poster is well organized and easy to follow; Appropriate headings used | 1  2 3  4 5 6  7 8 9 10 | 1  2 3  4 5 6  7 8 9 10 | 1  2 3  4 5 6  7 8 9 10 |
| **Formatting**: Text readable from an appropriate distance (3-6 feet); Font selection pleasing | 1  2 3  4 5 6  7 8 9 10 | 1  2 3  4 5 6  7 8 9 10 | 1  2 3  4 5 6  7 8 9 10 |
| **Graphics**: Graphics relevant & prominent, enhance poster, high quality (i.e. not pixilated) | 1  2 3  4 5 6  7 8 9 10 | 1  2 3  4 5 6  7 8 9 10 | 1  2 3  4 5 6  7 8 9 10 |
| **White space**: Balance among text, figures, and white space; Not cluttered | 1  2 3  4 5 6  7 8 9 10 | 1  2 3  4 5 6  7 8 9 10 | 1  2 3  4 5 6  7 8 9 10 |
| **Purpose of study**: Obvious, stated succinctly, and includes the significance of the work | 1  2 3  4 5 6  7 8 9 10 | 1  2 3  4 5 6  7 8 9 10 | 1  2 3  4 5 6  7 8 9 10 |
| **Methods**: Clear and concise description of methods provided | 1  2 3  4 5 6  7 8 9 10 | 1  2 3  4 5 6  7 8 9 10 | 1  2 3  4 5 6  7 8 9 10 |
| **Results**: Main result stated outcome of work and visualized with graphics to enhance understanding | 1  2 3  4 5 6  7 8 9 10 | 1  2 3  4 5 6  7 8 9 10 | 1  2 3  4 5 6  7 8 9 10 |
| **Conclusions**: Stated clearly, supported by results, pertain to current knowledge | 1  2 3  4 5 6  7 8 9 10 | 1  2 3  4 5 6  7 8 9 10 | 1  2 3  4 5 6  7 8 9 10 |
| **Presenter completeness**: Detail and depth is appropriate | 1  2 3  4 5 6  7 8 9 10 | 1  2 3  4 5 6  7 8 9 10 | 1  2 3  4 5 6  7 8 9 10 |
| **Presenter enthusiasm/energy**: Shows interest through tone and energy | 1  2 3  4 5 6  7 8 9 10 | 1  2 3  4 5 6  7 8 9 10 | 1  2 3  4 5 6  7 8 9 10 |

Part II: Choose **two posters** (other than those evaluated above) to ask questions after the presentation. Questions should be thoughtful and elicit critical thinking from the presenters about the study they performed.

#### **Poster #4**:

Question asked (brief):

Student reply (brief):

Did you feel that the student/s answered the question that you asked?

Explain if you feel that the student/s have a good understanding of their study based on their response.

#### **Poster #5**:

Question asked (brief):

Student reply (brief):

Did you feel that the student/s answered the question that you asked?

Explain if feel that the student/s have a good understanding of their study based on your question.

#### Part II. Which posters do you feel best represent your section for the LSU CURE Poster Session? Include your top two choices.

|  | **Choice 1** | **Choice 2** |
| --- | --- | --- |
| **Student last names:** |  |  |
