# Supplementary material for: Introducing a Novel Course-Based Undergraduate Research Experience Using Duckweed as a Model System
Source: Integr Org Biol. 2025 Dec 19;8(1):obaf049. doi: 10.1093/iob/obaf049 (PMC12802901; doi:10.1093/iob/obaf049)
Supplement: obaf049_Supplemental_Files [file obaf049_supplemental_files.zip › 07 Supplementary Materials/Supplementary Materials/53_Week13_RESOURCES_In-ClassPosterPresentations.docx]

In-class Poster Presentation Rubric

LSU CURE

| Introduction | 20 |  |
| --- | --- | --- |
| Elevator pitch touches on most important parts of study |  | /5 |
| Begins with the broadest possible context based on audience |  | /5 |
| Clearly identifies niche/gap in research field & addresses how authors intend to occupy gap/fill niche |  | /5 |
| Central research question is clearly stated |  | /5 |
| Methods | 10 |  |
| Establishes authors' credibility and demonstrates an understanding of the experiment |  | /2 |
| Does not include unnecessary information that distracts reader |  | /2 |
| Key components of data collection and analyses are emphasized |  | /6 |
| Results | 20 |  |
| Author uses figures & statistics to emphasize biological findings of experiment |  | /20 |
| Discussion | 20 |  |
| Interprets results to answer central research question and relates back to literature |  | /5 |
| Addresses all main concepts introduced in introduction section & does not bring up new main concepts |  | /2 |
| Considers possible weaknesses/limitations of study but doesn't stretch too far |  | /3 |
| Relates to literature and considers broader implications beyond scope of experiment |  | /5 |
| Specifically explains future directions given results of experiment |  | /5 |
| Poster Style | 20 |  |
| Color scheme: text clearly visible & not obscured by background; clean, simple design |  | /5 |
| Overall Clarity & Organization: main points clearly and concisely presented, level appropriate to audience, sequence logical, smooth transitions, easy to follow |  | /5 |
| Headers are used appropriately to identify sections |  | /2 |
| Font is large enough to be readable 6 feet away from the poster |  | /3 |
| The reader is considered throughout the paper. Only information relevant to story is included. |  | /5 |
| Poster Presentation | 10 |  |
| All group members present with roughly equal speaking time |  | /5 |
| Research and poster content clearly explained and presented to class, not read directly from poster |  | /5 |
|  |  |  |
| Total Score |  | /100 |

Instructor notes:
