# Supplementary material for: Introducing a Novel Course-Based Undergraduate Research Experience Using Duckweed as a Model System
Source: Integr Org Biol. 2025 Dec 19;8(1):obaf049. doi: 10.1093/iob/obaf049 (PMC12802901; doi:10.1093/iob/obaf049)
Supplement: obaf049_Supplemental_Files [file obaf049_supplemental_files.zip › 07 Supplementary Materials/Supplementary Materials/54_ARTIFACT_FWA2Spring23.pdf]

Impact of Fragmentation on Duckweed-Microbe Symbiosis

Introduction

Habitat fragmentation is the dispersion and erosion of habitual land for plants and animals (Kiesewetter et al., 2021). Habitat fragmentation is a threat to organisms' biodiversity and their function in ecosystems (Flores-Renteria et al., 2015). Fragmentation decreases biodiversity by shrinking organisms' environments, resulting in smaller spaces to breed and live. Fragmentation is a very common farming practice in the Mediterranean. Since the start of this farming practice, biodiversity of birds, butterflies and microorganisms have significantly decreased. Also, it has hindered many plants' ability to pollinate and disperse seeds (Flores-Renteria et al, 2015).

The effects that fragmentation has on plant-microbe symbiosis is a very under researched topic, but the few studies that researched this interaction did not come to one consensus on the effects of fragmentation on plant-microbe symbiosis (Weigand et al., 2005). Most research has studied the impact that habitat fragmentation has on animals and microbial interactions. By studying the impacts of fragmentation on plant-microbe symbiosis, we can achieve a better understanding on how fragmentation alters an ecosystem's biodiversity and function (Kiesewetter et al., 2021). Microbes are bacteria that vary in their ability and help sustain the fitness of organisms in harsh environments. This can be seen in the brighter color and larger area occupied by plants that interact with microbes, compared to plants that do not have plant microbe interactions. Plant-microbe symbiosis is a beneficial process for both organisms. Microbes can perform a multitude of tasks such as either absorbing heavy metals before they reach an organism or attracting heavy metals to support the organism; it depends on the demand of the

organism. In return, the plant provides the microbes with carbohydrates to keep performing and living. (O'Brien et al., 2020).

This study researches the impact that fragmentation has on plant micro symbiosis, with microbial and duckweed interactions. Lemna minor, duckweed, is a small, beneficial plant that lives in rivers and lakers. Many individuals view duckweed as a harmful, invasive species, but it is not. Duckweed cleans bodies of water by absorbing phosphorous and nitrogen (*What is Duckweed?)*. Duckweed cannot perform this responsibility on its own; it has the help of microbes. Duckweed was chosen as the studied organism because it is very easy to grow and collect (O'brien et al., 2020). Microbes are very valuable in duckweed growth and function, so studying and understanding how fragmentation can impact this interaction is very beneficial for duckweed health, in order to clean and maintain bodies of water.

For this study, the null hypothesis is that altering fragmentation size will not significantly impact the effectiveness of plant microbe symbiosis; the alternative hypothesis is that altering fragmentation size will significantly impact the effectiveness of plant-microbe symbiosis. It is predicted that the duckweed-microbial interactions in smaller habitats will not be as functionable or healthy compared to the duckweed and microbes that interacted in larger habitats because of the past observation that smaller fragmentation decreased the biodiversity and reproductivity of animals and plants in the Mediterranean (Flores-Renteria et al., 2015).

Methods

Experimental Design:

For the experiment, duckweed must not contain any initial microbes**,** so 6 colonies of duckweed were cleansed in 100 mL of water, 100 mL of 25% bleach, and 100 mL of water again using sterile techniques (O'Brien et al., 2020). To obtain the microbes for the study, 0.5 mL of water from the lake that the duckweed and microbes lived in was pipetted into 4.5 mL of sterile water. This mixture was then used to make a grand total of five different dilutions of solution. 10 μL of each of these solutions and the original stock solution were pipetted and streaked in individual plates. To isolate, or purify, one colony of microbes, one color of microbes were streaked in a plate by using sterile techniques. These microbes grew to later be inoculated in the test tubes.

The purpose of this study is to research the impact that varying fragmentation sizes has on the impact on plant-microbe symbiosis. For this study, WDRD duckweed from a small habitat size, was used to experiment. The three test groups for this experiment were communities of duckweed and microbes, duckweed only, and microbes only. These three test groups were inoculated in three varying test tube sizes (16 mm, 19 mm, and 25 mm) to stimulate different fragmentation sizes. Each treatment of duckweed had 3 replicates for each test tube size to increase accuracy in data. On day 0, duckweed was inoculated in test tubes that required them. Microbes were not added to their respective test tubes until day 7. Every 7 days, the total number of green and white fronds was counted and the microbial biomass for each test tube was recorded. This process was repeated every 7 days, for a total of 28 days.

Data Collection:

On day 0, one colony of duckweed was inoculated in the test tubes that required them. After inoculation, the number of green fronds in each test tube was counted and recorded. On day 7, microbes were added to test tubes that required them. The number of green and white fronds was counted and recorded for each test tube. 100 mL of solution from every test tube, was pipetted into one opening of a well 96 plate and then placed in the spectrometer for a microbe density reading (Weigand et al., 2005). This process of frond counting and a microbial density reading was repeated every 7 days, for a total of 28 days.

Data Analysis:

After all data was imputed into a spreadsheet, four graphs were plotted to compare ODU and total frond count. The first two graphs that were created and compared were the ODU of microbes only and ODU of duckweed and microbes. The final two graphs were the total frond count of duckweed and duckweed and microbes. For every graph, a linear fit line was generated and graphed. After the linear fit line was added, an r^2 value was calculated to compare how well the trend line fit the data collected.

Results

This experiment aimed to discover the relationship between habitat fragmentation and plant-microbe symbiosis. Habitat fragmentation was induced by using 3 different test tube sizes,

but it was not the only variable manipulated in this experiment. The treatment group was another variable manipulated and used to research the relationship. To measure the impact of habitat fragmentation, the microbial absorbance (ODU) was used as a measure of microbe density between each treatment for every test tube size by using a spectrometer. As seen in Figure 1, the general trend for the small test tube size was that microbe density increased slightly over 28 days.

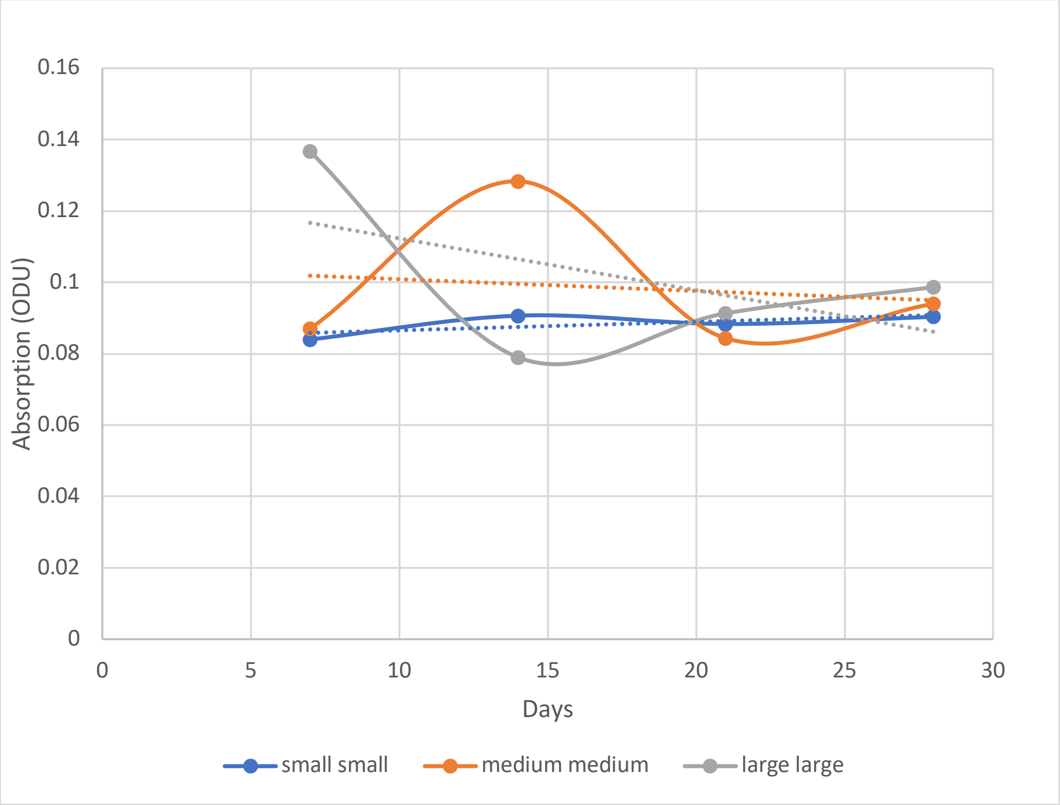

Figure 1. Microbe Treatment ODU.

The scatter plot displays the average ODU for the microbe only treatment in three test tube sizes over 28 days. For each curve, a linear fit line and r^2 value was calculated to determine how well the trend line fit the data.

The equation for the small test tube trend line was $y = 0.0002x + 0.0842$ with an r^2 value of 0.49.

Next, the trend line for the medium sized test tube was graphed using the equation $y = -0.0003x + 0.1042$ with an r^2 value of 0.0213. From this trend line, the microbial absorption for the

medium test tube decreased over 28 days. Finally, the line $y = -0.0015x + 0.1268$ had an r^2 value of 0.2787. Based off this trend line, the microbial absorbance from the large test tube decreased over time.

To compare the microbial absorbance between treatments, the average ODU for the duckweed and microbe treatment was graphed. As seen in Figure 2, each test tube size had a trend line graphed and a r^2 value calculated to display how well the trend line fit the data.

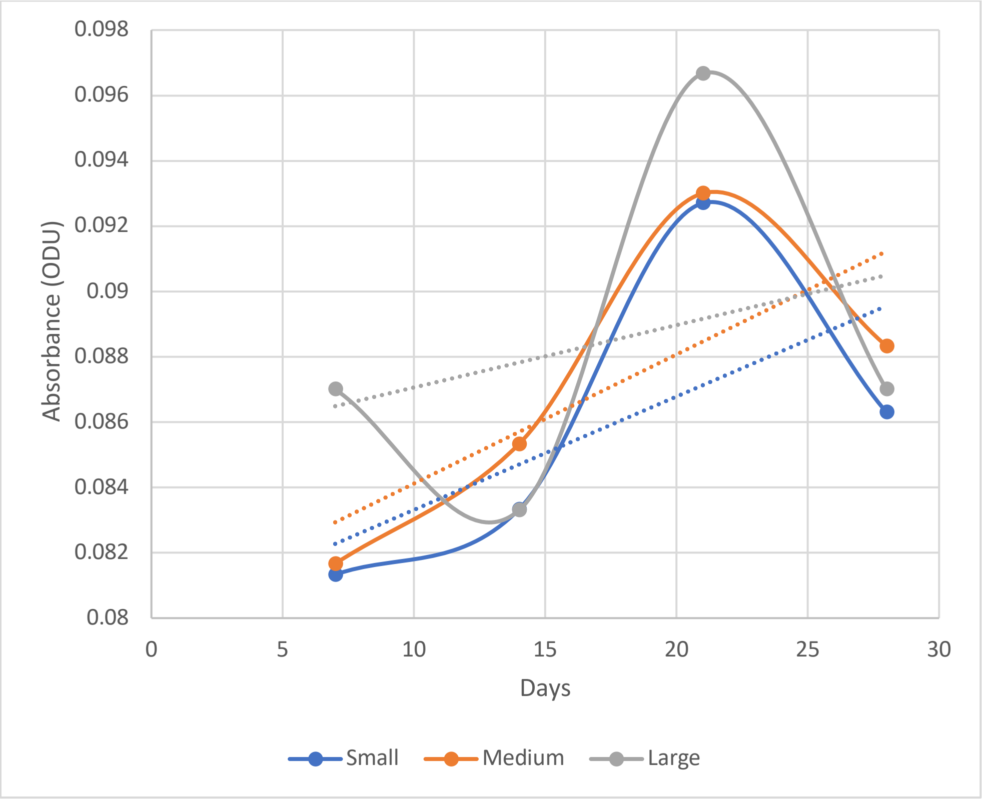

Figure 2. Duckweed and Microbes ODU.

The scatter plot displays the average ODU for the duckweed and microbe treatment in three test tube sizes over 28 days. For each curve, a linear fit line and r^2 value was calculated to determine how well the trend line fit the data.

The small test tube's trend line was graphed using the equation $y = 0.0003x + 0.0798$ and a calculated r^2 value of 0.3989. Then, the medium test tube's trend line was graphed by the equation $y = 0.0004x + 0.0802$ and a computed r^2 value of 0.5546. Finally, the trend line for the

large test tube was plotted with the equation y = 0.0002x + 0.0852 and a derived r^2 value of 0.0909. Based off the trend lines, the microbial absorbance increased over 28 days for every test tube size group.

The next measurement for the effects of habitat fragmentation was the total healthy frond count for each treatment. The duckweed only test group was first graphed to have a baseline comparison of duckweed growth with the microbe and duckweed treatment. In Figure 3, a trend line was graphed for each test tube size group to observe the trend for each fragmentation size.

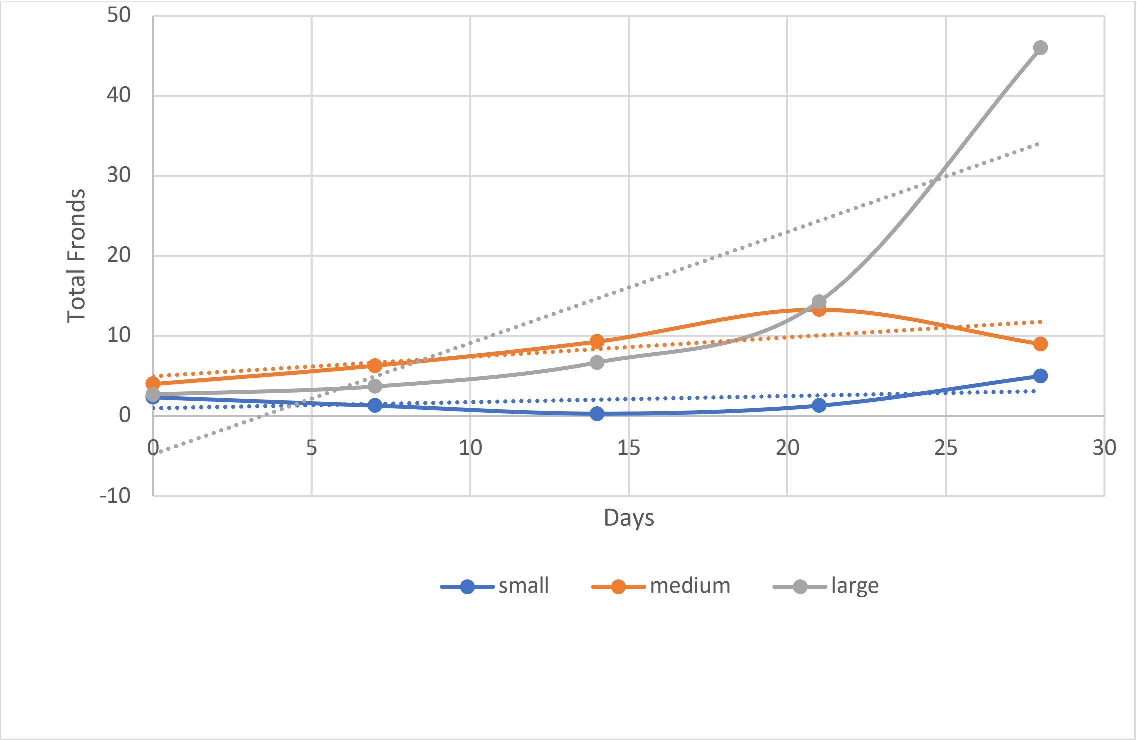

Figure 3. Duckweed Total Fronds.

The scatter plot displays the average total healthy front count for the duckweed treatment in 3 test tube sizes over 28 days. For each curve, a linear fit line and r^2 value was calculated to determine how well the trend line fit the data.

For the small test tube, a trend line equation of y = 0.0762x + 0.98 was graphed with an r^2 value of 0.2193. Afterwards, an equation of y = 0.2429x + 4.98 with an r^2 value of 0.5904 was graphed for the trend line of the medium size test tube. Finally, a trend line was graphed for the largest

test tube. The trend line equation was $y = 1.3886x - 4.76$ and an r^2 value of 0.7218. Based on all three trend lines, the total health frond count for every test tube the duckweed only treatment increased, but it is important to note that they all increased at different rates.

Finally, the total frond count for the duckweed and microbes solution was graphed and compared to the growth of the total fronds in the duckweed only treatment. For the duckweed and microbe treatment, the average number of healthy fronds for each test tube was graphed.

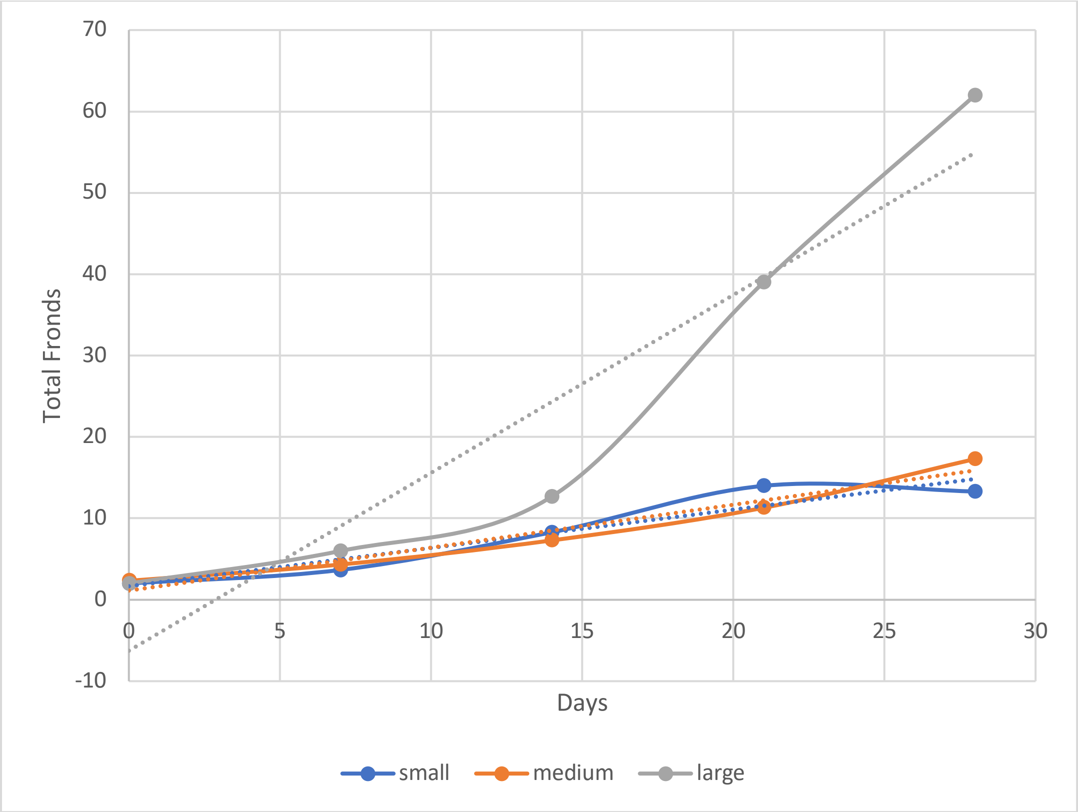

Figure 4. Microbe and Duckweed Total Fronds.

The scatter plot displays the average total healthy frond count for the duckweed treatment in 3 test tube sizes over 28 days. For each curve, a linear fit line and r^2 value was calculated to determine how well the data fits to the trend line.

For the small test tube, the line $y = 0.4705x + 1.6667$ was graphed with an r^2 value of 0.9142. Next, the medium size test tube had a trend line graphed by the equation $y = 0.5271x + 1.1333$ with an r^2 value of 0.9592. Finally, the large test tube's trend line was graphed using the equation $y = 2.1857x - 6.26$ and a computed r^2 value of 0.8989. Using these three trend lines, a trend was identified for each test tube size. As seen in Figure 4, each test tube had an increase in the total

number of fronds over a span of 28 days, but some test tubes increased by a faster rate than others.

Discussion

In this study, our research looked to identify any relationships between fragmentation size and the function ability of plant-microbe symbiosis. The null hypothesis for this experiment was that if the habitat fragmentation size were altered, then there would be no significant change in the function ability of plant-microbe symbiosis; the alternative hypothesis was that if habitat fragmentation size was changed, then there would be a significant change in the function ability of plant-microbe symbiosis. Based on past studies, it was predicted that a smaller fragmentation size would hinder the ability of plant-microbe symbiosis because the smaller fragmentation hinders the ability for a plant to disperse seeds and reproduce (Flores-Renteria et al, 2015). The first observation made during the study was the symbiotic relationship between plants and microbial density. In Figure 1, the microbial ODU decreased for every test tube size in the microbe only treatment, while in Figure 2, the microbial density increased for each fragmentation size of the duckweed and microbe treatment. The final observation was made viewing the total growth of health fronds in each treatment. The final trend was observed in Figures 3 and 4. Figure 3 displayed the total frond growth for duckweed only treatment. In this figure, the largest test tube resulted in the most duckweed grown, while the smallest tube resulted in the least amount of duckweed grown. Figure 4 graphed the total frond count for the duckweed and microbe treatment. In this figure, the largest test tube resulted in the most duckweed grown, while the smallest test tube resulted in the least amount of duckweed grown. Based off Figure 4, the null hypothesis was rejected, and the alternative hypothesis was supported because as the test tube changed size, the growth of the fronds significantly changed.

Plant-microbe symbiosis is a beneficial interaction for both the plant and microbes. During plant microbe symbiosis, the microbes increase a plants tolerance level of harsh conditions by improving the nutritional and water flow to plants and in return the plant supplies the microbes with an abundance of carbohydrates to maintain their performance (Khatabi et al., 2019). In Figure 1, the microbe could not benefit from this interaction, so the microbial density decreased over time, but in Figure 2 the microbes benefitted from the symbiotic relations causing an increase in microbial density over time. Figure 3 and 4 measured the total growth of healthy fronds of duckweed between the duckweed only and the duckweed and microbes' treatment. In Figure 3, the final frond number for the medium and small test tube were both around 7, while the final frond count for the large test tube was approximately 46. In Figure 4, the final frond count for the medium and small size test tube was approximately 15, while the find frond count for the large test tube was roughly 62. The duckweed in Figure 4 resulted in a higher total frond count because they could benefit from the symbiotic relationship between microbes and duckweed, while the duckweed in Figure 3 could not. In Figure 4, the duckweed grew at different rates because of how fragmentation impacted the relationship between the duckweed and microbes. The duckweed in the largest test tube (least fragmentation) experienced the greatest growth of duckweed, as seen in the trend line, and the duckweed in the smallest test tube (highest fragmentation) experienced the smallest amount of growth, as seen in the trend line. The higher fragmented area (smallest test tube) resulted in the smallest total frond growth because in highly fragmented areas microbes struggle to survive and reach their plant to interact and support the plant (Kiesewetter et al., 2023).

In this experimental design, there were a number of limitations that could have had a potential influence on the data. The first limitation was the duckweed used. This experiment used

WDRD which is a type that lives in small habitats. It is possible that duckweed from other habitat sizes would not respond the same way that the WDRD did in this experiment. The next limitation was the microbes present in each test tube. Each test size had its respective microbes, so the small test tubes had the small microbes, medium test tubes had medium microbes, and the large test tubes had the large microbes. WDRD is a small habitat duckweed, but it is possible that the duckweed might behave and grow differently with different sized microbes. The final limitation of this experiment is time. The data was only collected for 28 days; this is a very short amount of time to come to a definitive conclusion.

Plant-microbe symbiosis is a very beneficial interaction for humans. Our ecosystem is continually ramping up the dangers to plants such as global warming, erosion, and pollution. If plants cannot withstand harsh environments, then it will be very difficult for them to survive soon, but this symbiotic relationship benefits both plants and microbes and in the end, humans. In future experiments, the days tested should continue over 6 months to observe is this trend continues over a longer period. Because global warming is a major threat to plants today, future studies should expand upon this data by testing how plant-microbe symbiosis is impacted by different temperatures.

References

Kiesewetter, Kasey. N., & Afkhami, Michelle. (2021) Microbe-mediated effects of habitat fragmentation on plant performance. *New Phythol, 232.* (4), 1823-1838. 10.1111/nph.17595. doi: .1007/s00248-019- 01452-1. Epub 2020 Mar 2. PMID: 32123959.

Kiesewetter, K.N., Otano, L. and Afkhami, M.E. (2023), Fragmentation disrupts microbial effects on native plant community productivity. *Journal of Ecology*, *111.* (4), 761-772. https://doi- org.libezp.lib.lsu.edu/10.1111/1365-2745.14097

Khatabi, B., Gharechahi, J., Ghaffari, M. R., Liu, D., Haynes, P. A., McKay, M. J., Mirzaei, M., Salekdeh, G. H., Plant–Microbe Symbiosis: What Has Proteomics Taught Us?. *Proteomics*, *19.* (16). doi: 10.1002/pmic.201800105.

O'Brien, Annan M., Lins, Tiago, M., Yang, Yamin., Frederickson, Megan. E., Sinton, David., & Rochman, Chelsea. M. (2022). A Common Contaminant Shifts Impact of Climate Change on a Plant-Microbe Mutualism: Effects of Temperature, CO2, and Leachate from Tire Wear Particles. *Environ Res. 203,* 1-41. doi.org/10.1101/2020.05.19.105098.

O'Brien, Anna. M., Laurich, Jason., Lash, Emma., & Frederickson, Megan. E. (2020). Mutualistic Outcomes Across Plant Populations, Microbes, and Environments in the Duckweed Lemma minor. *Microb Ecol. 80.* (2), 384-397.

Flores-Renteria, Dulce., Curiel Yuste, Jorge., Rincon, Ana., Brearley, Francis. Q., Garcia-Gil, Juan Carlos., & Valladares, Fernando. (2015). Habitat Fragmentation can Modulate Drought Effects on the Plant-soil- microbial System in

Mediterranean Holm Oak (Quercus ilex) Forests. *Microb Ecol, 69.* Doi: 798-812. 10.1007/s00248-015-0584-9.

What is Duckweed? YouTube (2020). Retrieved March 5, 2023, from https://www.youtube.com/watch?v=rjFqqxAlk5I.

Wiegand, T., Revilla, E., & Moloney, K. (2005) Effects of Habitat Loss and Fragmentation on Population Dynamics. *Conservation Biology, Volume 19.* (Issue 1), 108-121.
