# Supplementary material for: Introducing a Novel Course-Based Undergraduate Research Experience Using Duckweed as a Model System
Source: Integr Org Biol. 2025 Dec 19;8(1):obaf049. doi: 10.1093/iob/obaf049 (PMC12802901; doi:10.1093/iob/obaf049)
Supplement: obaf049_Supplemental_Files [file obaf049_supplemental_files.zip › 07 Supplementary Materials/Supplementary Materials/55_ARTIFACT_FWA2Fall23.pdf]

How does temperature affect the growth of duckweed, *Lemna minor*, and its microbial communities?

Abstract: The impact of climate change and its rising temperatures affects the growth of duckweed and their microbial communities. The benefits and drawbacks of increased temperature on duckweed growth and microbial composition can vary depending on the environment that the duckweed inhabits. Furthermore, the interactions between biodiversity and ecosystem services, such as bioremediation and agriculture, are important for the ecosystem. 27 replicates of duckweed were obtained for OD600 and frond growth: 1-9 (high temperature), 10-18 (room temperature), and 19-27 (low temperature). The raw data from the 27 replicates were put into Excel and JMP to gain access to the mean and standard error values of the treatments. The OD600 for high treatment increased over 28 days while the frond growth only increased from day 14 to day 21. The OD600 on day 28 showed a statistical difference for the high treatment, and the frond growth showed a statistical difference on day 28 between the low and high treatments. This study emphasizes the potential applications of duckweed in various industries, such as biopharmaceuticals, agriculture, biofuel production, and phytoremediation. The need for further research includes examining how different temperatures and controlled environments affect duckweed's ability to remove pollutants.

Introduction

The ongoing effects of climate change heavily impact duckweed and its microbial communities due to its relations with an organism's metabolism and biochemical functions. Temperature acts as a catalyst in speeding up metabolic rates in microorganisms (Zhou *et al.*, 2016). Climate change can be detrimental to the ecosystem surrounding these microbial communities because the ecosystems' services are not as optimal. The services that the

ecosystem provides for the environment can range from decontaminating toxins to agricultural practices, which are beneficial for humans and livestock (O'Brien *et al.,* 2020). Without biodiversity, the ecosystem in which microorganisms live will collapse (Zhou *et al,.* 2016). Therefore, climate change causes a chain of reactions to follow that can eventually lead to a loss of biodiversity.

The relationship between temperature and plant-microbe interactions is important because the growth of microorganisms is affected. Microbes play a role in interfering with a plant's responses to its changing environment (Ishizawa *et al.,* 2017). Duckweed, for example, is subjected to temperature changes in its environment, and an increase in temperature will also increase the growth of duckweed (Ishizawa *et al.,* 2017). The correlation between temperature and the growth of duckweed will alter the composition of microbial communities in soil or aquatic environments (O'Brien *et al,.* 2020). These plant-microbe interactions are advantageous for the environment because duckweed *Lemna minor* has many functions, such as bioremediation, agricultural practices, and bioenergy production (O'Brien *et al,.* 2020). *L. minor's* role in bioremediation includes the process of detoxifying contaminants like zinc that pollute the water in its environment. The reason for the increase in the growth of microbial communities is that it also consists of bacteria that may be resistant to the effects of zinc and may even use it as a source of food (O'Brien *et al.,* 2020). Moreover, duckweed is also beneficial for agricultural practices and bioenergy production because it serves as a protein-rich food source for livestock and an alternative source of energy.

Climate change and its effects on the growth of duckweed, *Lemna minor*, is important for the natural environment and beyond because it plays a critical role in biodiversity as well as genetic diversity (Stewart *et al*., 2021). Moreover, temperature does not have a positive effect on

the growth of duckweed, and there can be a loss in microorganisms that live in the same ecosystem as the duckweed (Ishizawa *et al.,* 2017). The mutual relationship that temperature provides for duckweed can be beneficial yet harmful to the ecosystem; Excessive duckweed growth can potentially eliminate other aquatic microorganisms in the ecosystem, resulting in a loss of biodiversity (Ishizawa *et al.,* 2017). Scientists are attempting to address this issue by conducting experiments with the microorganisms that share an ecosystem with duckweed (Zhou *et al.*, 2016).

The research in this experiment will address these gaps by testing how temperature affects duckweed growth by determining how the duckweed media responds to environmental influences. The research question being tested in this experiment is how rising levels of climate change affect the growth of *Lemna minor* and its microbial communities. Moreover, there will be a statistically significant change in the growth of duckweed when subjected to temperature. The reasoning behind this is that temperature does not have any negative impact on the growth of duckweed because an increase in temperature will also increase the growth rate of microbes in the duckweed (Zhou *et al.,* 2016).

Methods

Biological Materials

The duckweed medium was obtained from a lake by the TA. To prepare the duckweed to reach an axenic state, 5 replicates were obtained for the duckweed to be bleached. To transfer the duckweed from the falcon tube into the beakers, a loop was sterilized with the Bunsen burner. First, the duckweed was transferred from the falcon tube to the water solution. Second, the duckweed from the water solution was transferred to the bleach solution for 10-20 seconds.

Lastly, the duckweed from the bleach solution was rinsed when transferred to the other solution of DI water.

For serial dilutions for the microbial plating, 5 test tubes were obtained and dispensed with 4.5 mL of sterile water. The tubes were labeled for the dilution of the duckweed. To start the dilution of duckweed, 0.5 microliters of water were extracted from the Falcon Tube of duckweed, and it was dispensed into a -1 dilution test tube. After pressing the plunger of the micropipette 20 times into the test tube, 0.5 microliters of the solution were extracted and dispensed into the -2 dilution. The dilution process was continued until the -5 dilution test tube. The 5 dilution tubes were then vortexed for a few seconds. After, 5 petri dishes were obtained. 10 microliters of the solution were extracted from each of the dilution tubes. They were dispensed into their respective petri dish. After each use of the dilution tube extraction, the pipette was replaced. The solution was dispensed in the middle of the petri dish and was spread along the whole petri dish with the spreader. After each use of the spreader in every petri dish, it was sterilized with ethanol. The samples were then put into the incubator for growth.

The duckweed from the petri dish was purified through microbial streaking. The petri dishes were obtained from the incubator. A sterile loop touched the bacteria from the red, yellow, and white cultures and was streaked 4 times onto a new petri dish, separately. In between those 4 increments, the loop was sterilized through the flame.

Experimental Design

To measure the growth of the duckweed, the duckweed was inoculated. 27 replicates were obtained, and 1 cluster of duckweed was extracted with a sterile loop. Each replicate was flamed for sterilization before and after replacing the cap. Each set of 9 replicates was grown at

different temperatures. Replicates 1-9 were grown at high temperature. Replicates 10-18 were grown at room temperature. Replicates 19-27 were grown at low temperature.

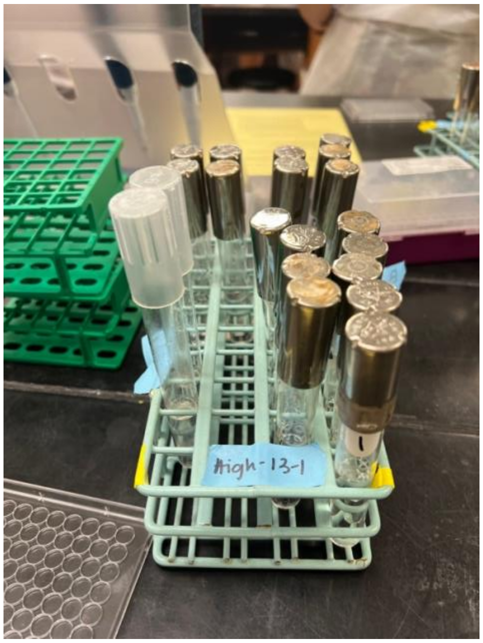

Figure 1. Duckweed replicates 1-9 grown under high temperatures. The duckweed in the replicates was put at 40 degrees Celsius to measure the effects of temperature on the growth of duckweed.

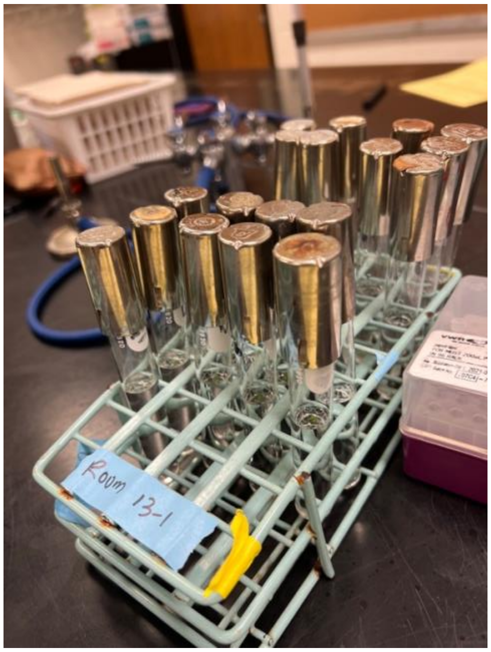

Figure 2. Duckweed replicates 10-18 grown under room temperature. The duckweed in the replicates was put at 20 degrees Celsius to measure the effects of temperature on the growth of duckweed.

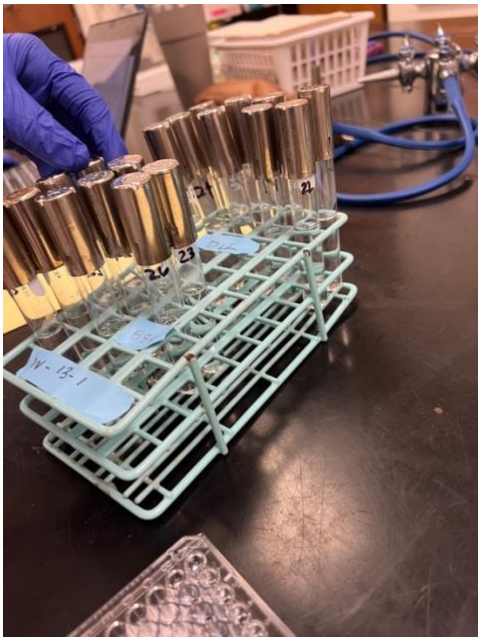

Figure 3. Duckweed replicates 19-27 grown under low temperature. The duckweed in the replicates was put at 30 degrees Celsius to measure the effects of temperature on the growth of duckweed.

Data Collection

The raw data was obtained from the number of fronds in each of the 27 replicates. The fronds were counted for days 0, 7, 14, 21, and 28. The data was put into the Excel Data Sheet and the 96 Well Plate Reader to gain access to the mean for the optical density and duckweed growth. The data from the 27 replicates from days 0, 7, 21, and 28 were put into the OD600 to measure the growth of the duckweed.

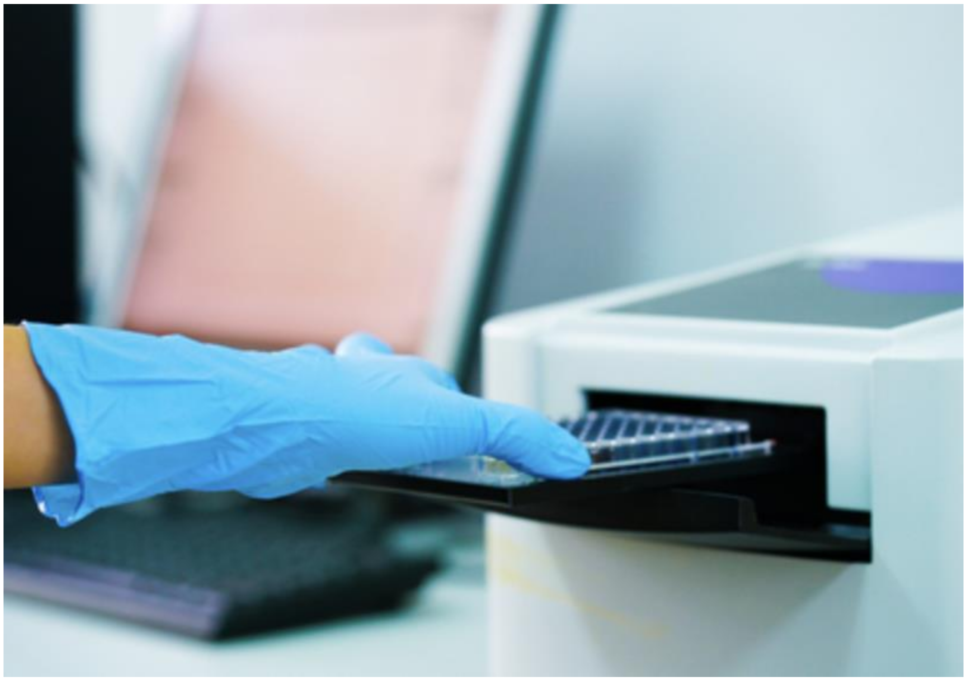

Figure 4. Duckweed replicates 1-9, 10-18, and 19-27 inserted into 96 Well Plate Reader for OD600 readings. The OD600 readings help determine the output from days 0, 7, 14, 21, and 28.

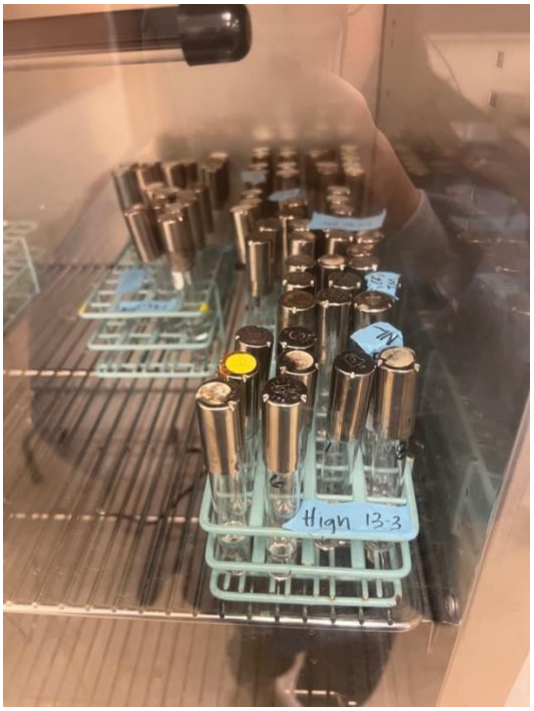

Figure 5. Duckweed replicates 1-9, 10-18, and 19-27 placed into incubator of high, room, and low temperatures, respectively. The growth of the duckweed at each of the 3 temperatures will determine which highly affects growth.

Data Analysis

The raw data obtained from OD600 and the growth of duckweed from the number of fronds were listed in the Excel Data Sheet to gain access to data regarding the mean and standard error for the line graph from OD600 and frond growth. The raw data was collectively organized by the specified temperatures of high, room, and low temperatures and their duration of days 0, 7, 14, 21, and 28. The Excel Data Sheet also gained access to the trendline from the scatterplots from OD600 and frond growth. JMP was the program used to compute the raw data with the means of oneway ANOVA, the mean, and the standard errors from the line graphs.

Results

The OD600 output for low and normal temperatures through the duration of 28 days stayed almost constant with no increase in OD600 (see **Figure 6)**. However, the OD600 output for high temperature through the duration of 28 days increased. The mean between the normal and low temperatures was closer in numbers than compared to that of the high temperature at 0.12556 (see **Figure 6)**. The OD600 of the high temperature on day 14 was 0.122554 and increased to 0.169776 on day 21. Meanwhile, the room and low temperatures on day 21 were 0.091608 and 0.092605, respectively (see **Figure 6**). On day 28, there was a statistical difference between all 3 treatments because the whiskers did not overlap (see **Figure 6**).

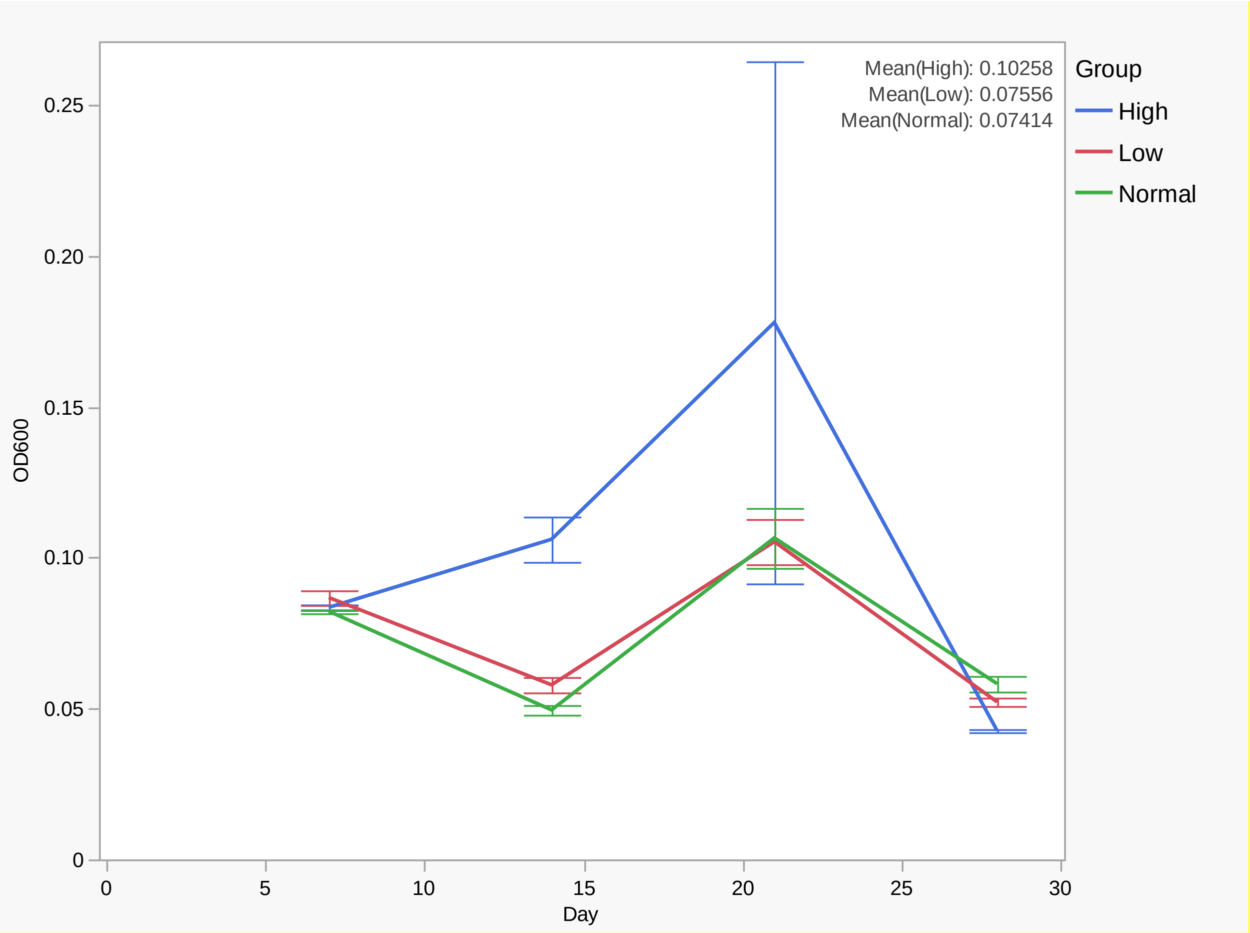

Figure 6. The mean and standard error of OD600 at high, room, and low temperatures on days 7, 14, 21, and 28. The OD600 for the temperature and day are found in the graph and can be calculated to find the specific output of OD600.

The mean and standard error of the high-temperature treatments increased from day 7 to day 28 in **Table 1**. The low temperature mean and standard error had a slightly lower value than the normal temperature treatment (see **Table 1**). The standard error of the high and low temperatures was the same at 0.05046, which showed that there was no statistical difference (see **Table 1**). However, the standard error of the room temperature was higher by 0.5046, which showed that there was a statistical difference as seen in **Table 1**. The standard error for all 3 treatments was the same on day 14 at 0.00466 and showed no statistical difference (see **Table 1**).

Table 1. Days 7, 14, 21, and 28 mean and standard error values of OD600 treatments. The mean and standard error of the high, room, and low treatments are calculated by ANOVA according to their specified temperatures.

**Treatments**	**Mean/SD**	**Letter**	**Temperature**
**Day 7**			
High	0.083556/0.00155	A, B	40°C
Low	0.086778/0.00155	A	30°C
Normal	0.082222/0.00155	B	20°C
**Day 14**			
High	0.106111/0.00466	A	40°C
Low	0.057889/0.00466	B	30°C
Normal	0.049556/0.00466	B	20°C
**Day 21**			
High	0.178000/0.05046	A	40°C
Low	0.105333/0.05046	A	30°C
Normal	0.106556/0.5046	A	20°C
**Day 28**			
High	0.042667/0.00173	C	40°C
Low	0.052222/0.00173	B	30°C
Normal	0.058222/0.00173	A	20°C

The frond growth starting on day 7 for the low temperature decreased from 3.2 to 0 on day 21 in **Figure 7**. There is no statistical difference in frond growth on day 14 and day 21 between the room and low temperatures because the whiskers overlap each other. The frond growth on day 28 for high temperature was relatively, yet slightly lower than that of day 21 (see **Figure 7**). Over, 21 days frond growth at high temperatures decreased from 2.5 to 1.9. The frond growth mean of low temperature on day 7 at 3.2 was significantly greater than the high temperature of 1.3 on day 7 (see **Figure 7**). The room temperature mean on day 7, however, was 2.75. The low temperature mean on day 28 was 0.48876 while the high temperature mean on day 28 was 1.133764 (see **Figure 7**). The whiskers on the line graph did not overlap, which showed that there was a statistical difference between these 2 treatments.

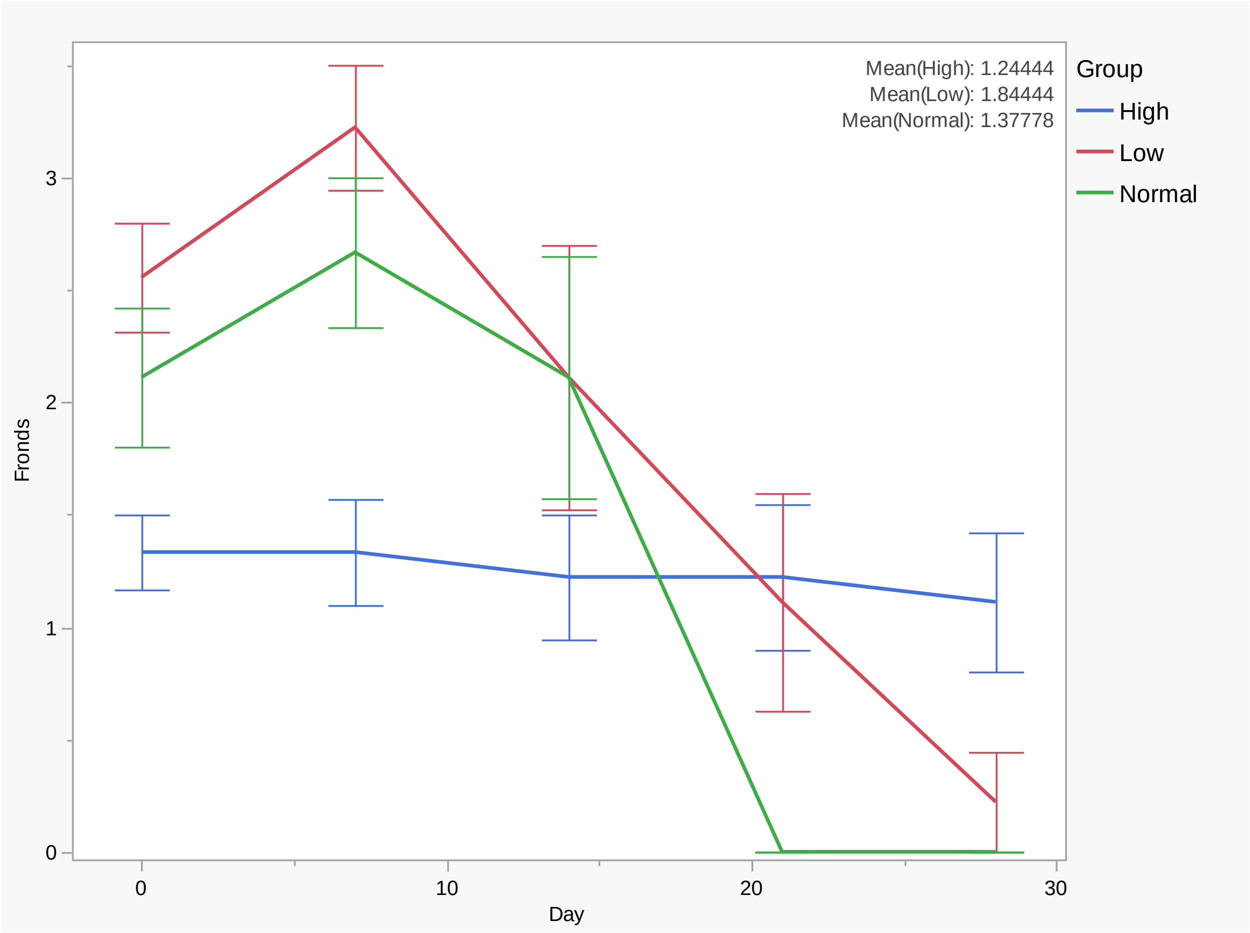

Figure 7. The high, room, and low temperature means of frond growth through the duration of 28 days. The mean was highly affected by the different temperatures on frond growth over 28 days.

The mean of the frond growth on day 7 of the low treatment was significantly higher than the other 2 treatments, which results in a significant difference as seen in **Table 2**. There is more of an increase in frond growth for the low-temperature treatment than compared to the high-temperature treatment (see **Table 2**). The standard error of the 3 treatments on days 0, 7, and 14 are all 0.24637, 0.28509, and 0.48750, respectively (see **Table 2**); This resulted in no significant change in frond growth. The mean for day 21 and day 28 were both and showed no statistical difference as seen in **Table 2**.

Table 2. Days 7, 14, 21, and 28 mean and standard error values of frond growth treatments. The mean and standard error of the high, room, and low treatments are calculated by ANOVA according to their specified temperatures.

Treatments	Mean/SD	Letter	Temperature
**Day 0**			
High	1.33333/0.24637	B	40°C
Low	2.55556/0.24637	A	30°C
Normal	2.11111/0.24637	A	20°C
**Day 7**			
High	1.33333/0.28509	B	40°C
Low	3.22222/0.28509	A	30°C
Normal	2.66667/0.28509	A	20°C
**Day 14**			
High	1.22222/0.48750	A	40°C
Low	2.11111/0.48750	A	30°C
Normal	2.11111/0.48750	A	20°C
**Day 21**			
High	1.22222/0.33641	A	40°C
Low	1.11111/0.33641	A	30°C
Normal	0.00000/0.33641	B	20°C
**Day 28**			
High	1.11111/0.21990	A	40°C
Low	0.22222/0.21990	B	30°C
Normal	0.00000/0.21990	B	20°C

Discussion

The effects of temperature will have no significant change on the growth of duckweed. On the other hand, the effects of temperature will have a significant change on the growth of duckweed. Temperature does not have any negative impact on the growth of duckweed because an increase in temperature will also increase the growth rate of microbes in the duckweed (Zhou *et al.*, 2016). From the results of the experiment, the duckweed growth from the fronds fails to reject the null hypothesis because there is no significant change in the effects of temperature on the growth of duckweed. The low-temperature treatment had a spike in increase on day 7 but stayed almost constant from there. The standard error for all 3 treatments overlapped each other and showed no statistical difference in frond growth. However, the microbial growth (OD600)

results support the alternative hypothesis because, on day 28, there was a statistical difference between all 3 treatments with no whiskers overlapping.

Since the standard error numbers overlapped in the OD600 for the 3 treatments on day 7, there was no statistical difference, resulting in the rejection of the alternative hypothesis. However, the normal treatment standard error for OD600 was significantly higher than the high and low treatments, resulting in the support of the alternative hypothesis. One reason behind these results depends on the environment that the duckweed was obtained from; The growth of duckweed can both have positive and negative effects based on the bacterial communities that surround it in freshwater biosystems (Ishizawa *et al*., 2017). Moreover, since the standard error numbers overlapped and had the same number for frond growth for the 3 treatments on days 0, 7, 14, and 21, this resulted in a rejection of the alternative hypothesis. The normal treatment on day 21 for the mean at 0 was lower compared to the high and low treatments, showing a statistical difference, and failing to reject the alternative hypothesis. There were a few unexpected results regarding the OD600 and frond growth because both the graphs and the tables showed a peak in growth for OD600 and frond growth but decreased by day 28. The study system and methods may not be the same as this experiment because the environment that the duckweed was obtained from was in contact with other bacterial microbes that could have affected its growth (Ishizawa *et al.,* 2017). Ishizawa et al. (2017) conducted their experiments by isolating the bacterial communities in which the duckweed grew and yielded promotive and inhibitory results.

There are some limitations that can be applied to this experiment. One limitation is human error because there could have been a miscalculation with counting the number of fronds after inoculation or an error in micro-pipetting the correct number of media. Another limitation is the constraint of existing knowledge, such as research that may have been overlooked before

testing out the experiment and learning what other environmental effects the duckweed grew in were easily subjected to. The accuracy of the results may not fully be correct due to errors in interpreting raw data and graphs. Also, the sampling techniques could have led to errors in the experiment if the vortex was not used or the wrong amount of medium was dispensed into the well plate when gathering OD600 data.

The big picture of this study is to determine how well duckweed can play into everyday human productibility. This can apply to biopharmaceutical purposes, agricultural remediation, biofuel production, and bioremediation (Ishizawa *et al.,* 2017). Someone outside the classroom should care because it may affect their lives when it comes to what their jobs entail and their health systems. In the oil industry, duckweed plays a huge role in phytoremediation, such as decontaminating water pollutants or even oil spills (O'Brien *et al.,* 2020). Someone working in the agricultural industry should care because duckweed helps improve nutrient management, livestock feed, and wastewater treatment (Stewart *et al*., 2021). They can use the growth of duckweed as a feedstock for animals due to its source of protein (Stewart *et al*., 2021). Furthermore, for future studies, these results can be expanded upon by taking each of the duckweed from the high, room, and low temperatures and putting them in 3 different controlled environments to test how efficiently each one can contribute to removing water pollutants in lakes or other bodies of water. Not only that but 3 samples of the same water pollutant should be obtained and dispensed with the duckweed to collect the data on the removal of the pollutant.

References

Ishizawa, H., Kuroda, M., Morikawa, M. *et al.* Evaluation of environmental bacterial communities as a factor affecting the growth of duckweed *Lemna minor* . *Biotechnol Biofuels* 10, 62 (2017). https://doi.org/10.1186/s13068-017-0746-8

O'Brien, A.M., Laurich, J., Lash, E. *et al.* Mutualistic Outcomes Across Plant Populations, Microbes, and Environments in the Duckweed *Lemna minor*. *Microb Ecol* **80**, 384–397 (2020). https://doi.org/10.1007/s00248-019-01452-1

Stewart, J. J., Adams, W. W., López-Pozo, M., Doherty Garcia, N., McNamara, M., Escobar, C. M., & Demmig-Adams, B. (2021, June 12). *Features of the Duckweed Lemna That Support Rapid Growth under Extremes of Light Intensity*. MDPI. https://doi.org/10.3390/cells10061481.

Zhou, J., Deng, Y., Shen, L., Wen, C., Yan, Q., Ning, D., Qin, Y., Xue, K., Wu, L., He, Z., Voordeckers, J. W., Nostrand, J. D. V., Buzzard, V., Michaletz, S. T., Enquist, B. J., Weiser, M. D., Kaspari, M., Waide, R., Yang, Y., & Brown, J. H. (2016, July 5). *Temperature mediates continental-scale diversity of microbes in forest soils*. Nature News. https://www.nature.com/articles/ncomms12083/
