# Supplementary material for: Introducing a Novel Course-Based Undergraduate Research Experience Using Duckweed as a Model System
Source: Integr Org Biol. 2025 Dec 19;8(1):obaf049. doi: 10.1093/iob/obaf049 (PMC12802901; doi:10.1093/iob/obaf049)
Supplement: obaf049_Supplemental_Files [file obaf049_supplemental_files.zip › 07 Supplementary Materials/Supplementary Materials/56_ARTIFACT_FWA2Spring24.pdf]

Turion Germination at Varying Temperatures for *Spirodela polyrhiza* Over Time

Abstract:

Several plants such as *Spirodela polyrhiza* are dependent on certain temperatures to be able to grow, so as global warming continues to worsen and temperatures continue to rise, plant species could die off due to a lack of cool temperatures needed to germinate and grow. Because temperatures are inevitably going to rise, it is important to study the effects of different temperatures on plants thus making the research question of "how do differing temperatures affect the growth and development of *Spirodela polyrhiza*" an important one. It was predicted that as temperatures increase, the growth of *Spirodela polyrhiza* will decrease because the plant needs cooler temperatures to germinate (Appenroth et al., 2002). In this experiment, 10 mL of sterile plant medium were pipetted into three trays of six petri dishes and stored at three different temperatures of 20 degrees, 30 degrees, and 40 degrees. Photos of the frond growth over several different weeks were then uploaded to and analyzed with ImageJ and placed into an excel sheet. It was found that the fronds had the most percent coverage over time at thirty degrees and the most number of fronds at twenty degrees. The fronds grew the least at the high temperature of forty degrees. In this experiment, it was found that fronds grow the best at room temperature and they are unable to survive and grow when the temperatures reach forty degrees. This is a problem because if climate change continues to raise the water temperatures to this point, plants like *Spirodela polyrhiza* will be unable to continue to grow.

Introduction:

The average temperature of the earth is rising at a rapid rate right now, increasing the temperature of both the air and the water (Dietz et al., 2020). This is due to the issue of global warming, which is caused by increased carbon emissions being released into the atmosphere (Dietz et al., 2020). As the temperature in the air rises, it in turn raises the temperature in the water and land. (Dietz et al., 2020) As time goes on, the water will continue to get warmer if nothing is done to stop climate change, affecting the plants and animals that live in these waters.

Certain organisms have adapted their survival methods around the average temperatures of their environment. The organism *Spirodela polyrhiza* is an example of an organism that does this. *Spirodela polyrhiza* is a form of duckweed that forms turions as a survival mechanism and way of reproducing (Appenroth et al., 1996). Turions are a survival mechanism of the *Spirodela polyrhiza* that allows them to survive the colder months by having the turions drop to the bottom of a body of water in order to avoid the harsh conditions on the surface and rise to the top of the water when the weather gets warmer to bloom (Appenroth et al., 1996). In this study the effect of varying temperatures on turion growth is observed. Cooler temperatures are needed for the germination of several types of plants, including *Spirodela polyrhiza* (Appenroth et al., 2002). The turions that duckweed forms have a unique way of germinating that is dependent on cooler temperatures coming in the winter (Appenroth et al, 2002). If the temperatures rise and the cooler months do not get cold, it could affect the way turions germinate, possibly making their germination less successful.

Although preventing the waters that duckweed grows in from warming is an implausible solution to this problem, learning the ways to stimulate turion growth in different conditions like increasing phosphate levels and high photon fluorescence is a tangible way to help these plants

survive global warming (Dudley et al., 1987). Several studies have been conducted on the effects of some varying conditions on turion growth; however, how specific temperatures affect growth with other variables remaining constant has yet to be studied (Dudley et al., 1987). The research that others have done is important to see what has already been discovered in the area of interest and to determine the gaps to see what still needs to be studied.

In this study, the gaps in research are addressed with the main research question of how do different temperatures affect the growth of turions from *Spirodela polyrhiza*. The null hypothesis for this experiment is that differing temperatures will not have a significant effect on the growth and development of turions for *Spirodela polyrhiza* over time. The alternative hypothesis for this experiment is that differing temperatures will have a significant effect on the growth and development of turions for *Spirodela polyrhiz*a over time. The prediction for this experiment is that as temperatures increase, turion growth for *Spirodela polyrhiza* will begin to slow down because lower temperatures aid the formation of turions (Appenroth et al., 2002).

Methods

Experimental Design

To begin the experiment, three empty test tubes were sterilized, and sterile techniques were maintained throughout the experiment. 4.5 mL of clean water was then obtained and put into the three sterilized test tubes. Then, 0.5 mL of pond water was placed into the first, least diluted, test tube labels 10-1 and mixed. The solution was then diluted further to the -3 point. 10 ul of the solution were then plated and spread in separate petri dishes along with a control group of clean water and a sample of pure pond water. Then, 10mL of sterile plant medium was pipetted into each of the 18 wells, and one frond was placed in each well.

Data Collection:

Photos of the eighteen wells were then taken and uploaded to ImageJ. Using the ImageJ program, photos of the wells were analyzed. In order to get accurate results, each well was analyzed separately. Then, the multipoint tool was used to count each frond individually. The scale was then set for each well using the known distance of 34.8 mm. After, the freehand tool was used to measure the area of the duckweed fronds. The area, percent coverage, and number of fronds were recorded in an excel sheet for each well.

Data Analysis:

The python coding program was used to obtain line graphs and box plots showing percent coverage over time and number of fronds among treatments. The graphs compared the three different treatment temperatures of 20, 30, and 40 degrees over 28 days with the mean values being recorded every seven days. Additionally, six different tables were obtained with two two-way ANOVAS and four Tukey tests. The first two-way ANOVA test analyzed the effect of the day, treatment, and the interaction of the day and treatment on the percent coverage and generated p-values. The second table compared the effect of the day, treatment, and interaction of the day and treatment on the number of fronds and generated a p-value.

Results:

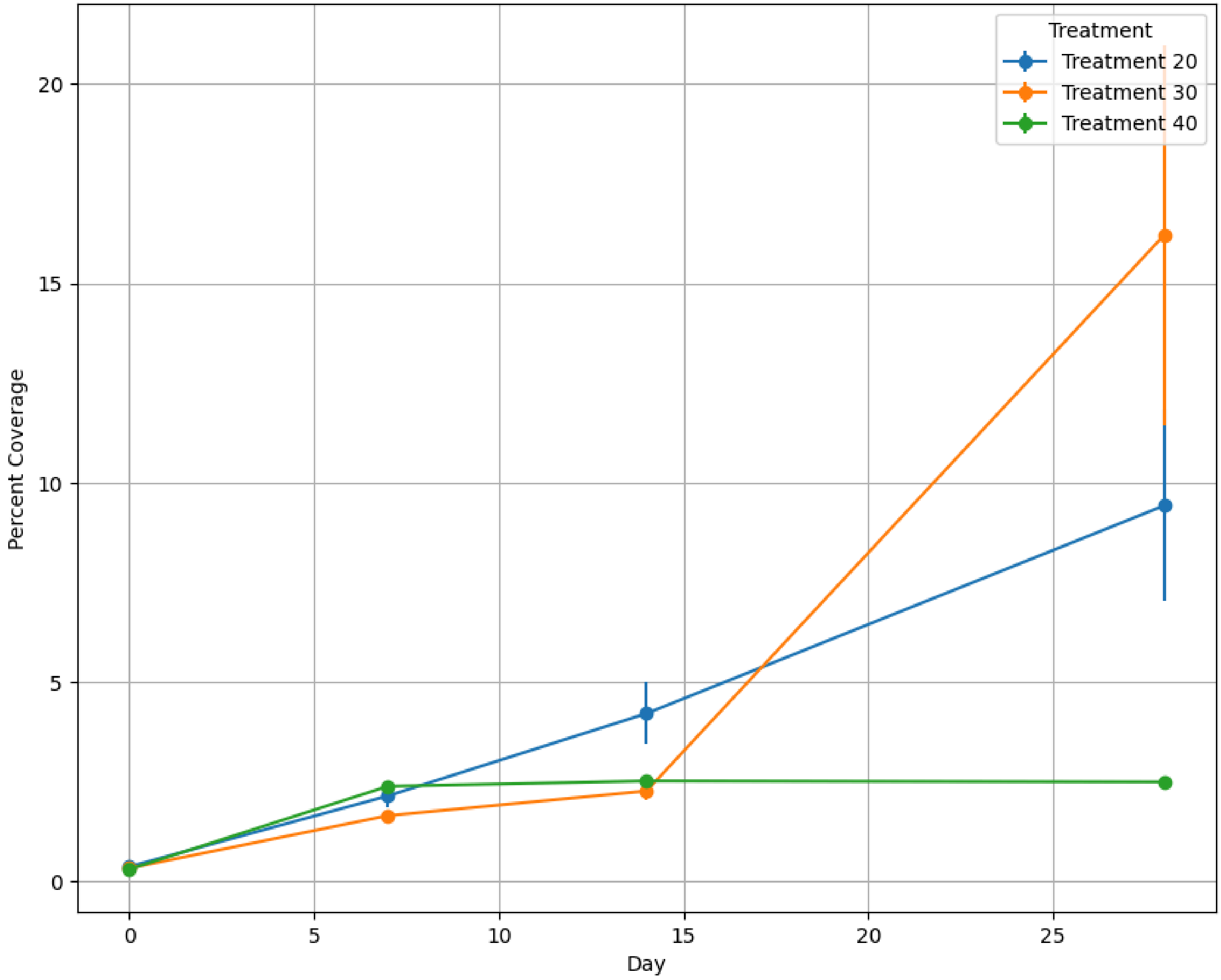

Figure 1. Line graph of percent coverage of turions for three different treatment group temperatures (20 degrees, 30 degrees, and 40 degrees) over time (a span of 28 days). The mean and standard error bars are displayed.

In figure 1, the treatments for 20 and 30 degrees continued to grow the whole time whereas the treatment for 40 degrees stopped increasing after day 7. Treatment 30 had the most percent

coverage by day 28, and treatment 40 had the least. After day 14, treatment 30 had a very high increase and passed up the other two treatments. There is no significance among the treatments because the standard error bars overlap. The rate of increase in percent coverage showed no significant increase or difference among treatments (p=2.5, table 1)

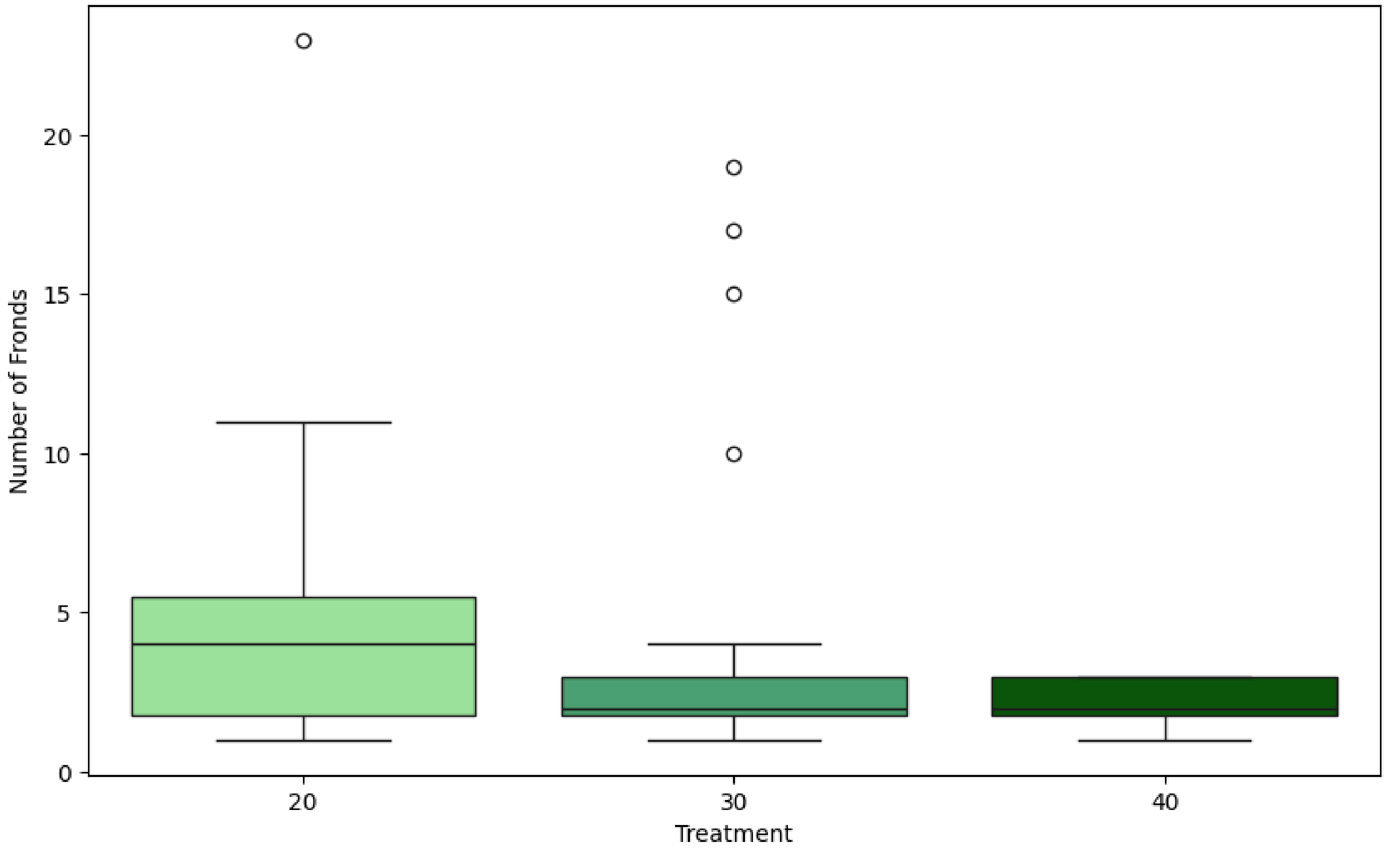

Figure 2. Boxplot of the number of fronds among three different temperature treatment groups (20 degrees, 30 degrees, and 40 degrees). Standard error bars and median bars are displayed.

In figure 2, As the temperature got warmer, the number of fronds got smaller. Treatment 20 had the most number of fronds, and treatment 40 had the least number of fronds. Treatment 30 and 40 had similar numbers of fronds. There is no significance among treatments because the

standard error bars overlap. The three treatments showed no significant difference among treatments for the number of fronds (p=6.41, figure 2)

Discussion:

The prediction that as temperatures increase, turion growth for *Spirodela polyrhiza* will begin to slow down because lower temperatures aid the formation of turions was supported through this experiment (Appenroth et. al., 2002). The null hypothesis that differing temperatures will have no significant effect on the growth and development of *Spirodela polyrhiza* over time for percent coverage was failed to be rejected, and the alternative hypothesis that differing temperatures will have a significant effect on *Spirodela polyrhiza* over time was rejected because the anova P- value was higher than 0.05. Therefore, it cannot be proven with our current data that the changing temperatures did have a significant effect on the percent coverage of fronds over time. The null hypothesis for the number of fronds was failed to be rejected, and the alternative hypothesis was rejected because the p-value was higher than 0.05. Therefore, we cannot prove with our data that changing temperatures did have a significant effect on the number of fronds over time. The 20 degree treatment grew the most fronds and had a consistent increase in percent coverage because it was a good temperature that the fronds were able to thrive in. At a certain point, the 40 degree treatment stopped increasing in percent coverage and grew the least number of fronds because the turions could not grow and died at this high temperature.

One possible limitation to this experiment could be improper sterilization techniques and human error using image J to measure frond growth. These errors could provide a false report of some results and mess up the validity of the study. Another major limitation could be the differing temperature of the room that could affect the growth of the control group that is

supposed to be at 20 degrees. The fluctuation in the temperature in the room could significantly affect the growth of the turions meant to be grown at 20 degrees.

The big picture of this study is the effects that rising temperatures have on different plant growth, including *Spirodela polyrhiza*. Plants are suited to germinate and grow at a certain temperature, so increasing the temperatures that they are exposed to has a significant and detrimental effect on their growth. The real-world application to the study is climate change. As climate change continues to worsen, the temperatures rise beyond control. This could have a horrible effect on plants and plant growth in the future as water and air temperatures warm.

References:

Appenroth, K.J., Teller, S. & Horn, M. Photophysiology of turion formation and germination in Spirodela polyrhiza . Biol Plant 38, 95–106 (1996). https://doi.org/10.1007/BF02879642

Appenroth, K.J. (2002). Co‐action of temperature and phosphate in inducing turion formation in Spirodela polyrhiza (Great duckweed). Plant, Cell & Environment, 25(9), 1079-1085. https://doi.org/10.1046/j.

Cordero EC, Centeno D, Todd AM (2020) The role of climate change education on individual lifetime carbon emissions. PLoS ONE 15(2): e0206266. https://doi.org/10.1371/journal .pone .0206266.

Dietz, T., Shwom, R. L., & Whitley, C. T. (2020). Climate change and society. Annual Review of Sociology, 46, 135-158. https://doi.org/10.1146/annurev-soc-121919-054614.

Dudley, J. L. (1987). Turion formation in strains of Lemna minor (6591) and Lemna turionifera (6573, A). Aquatic botany, 27(2), 207-215. https://doi.org/10.1016/0304-3770 (87)90069-6.
