# Supplementary material for: Introducing a Novel Course-Based Undergraduate Research Experience Using Duckweed as a Model System
Source: Integr Org Biol. 2025 Dec 19;8(1):obaf049. doi: 10.1093/iob/obaf049 (PMC12802901; doi:10.1093/iob/obaf049)
Supplement: obaf049_Supplemental_Files [file obaf049_supplemental_files.zip › 07 Supplementary Materials/Supplementary Materials/57_ARTIFACT_PosterSpring23.pptx]

## Slide 1
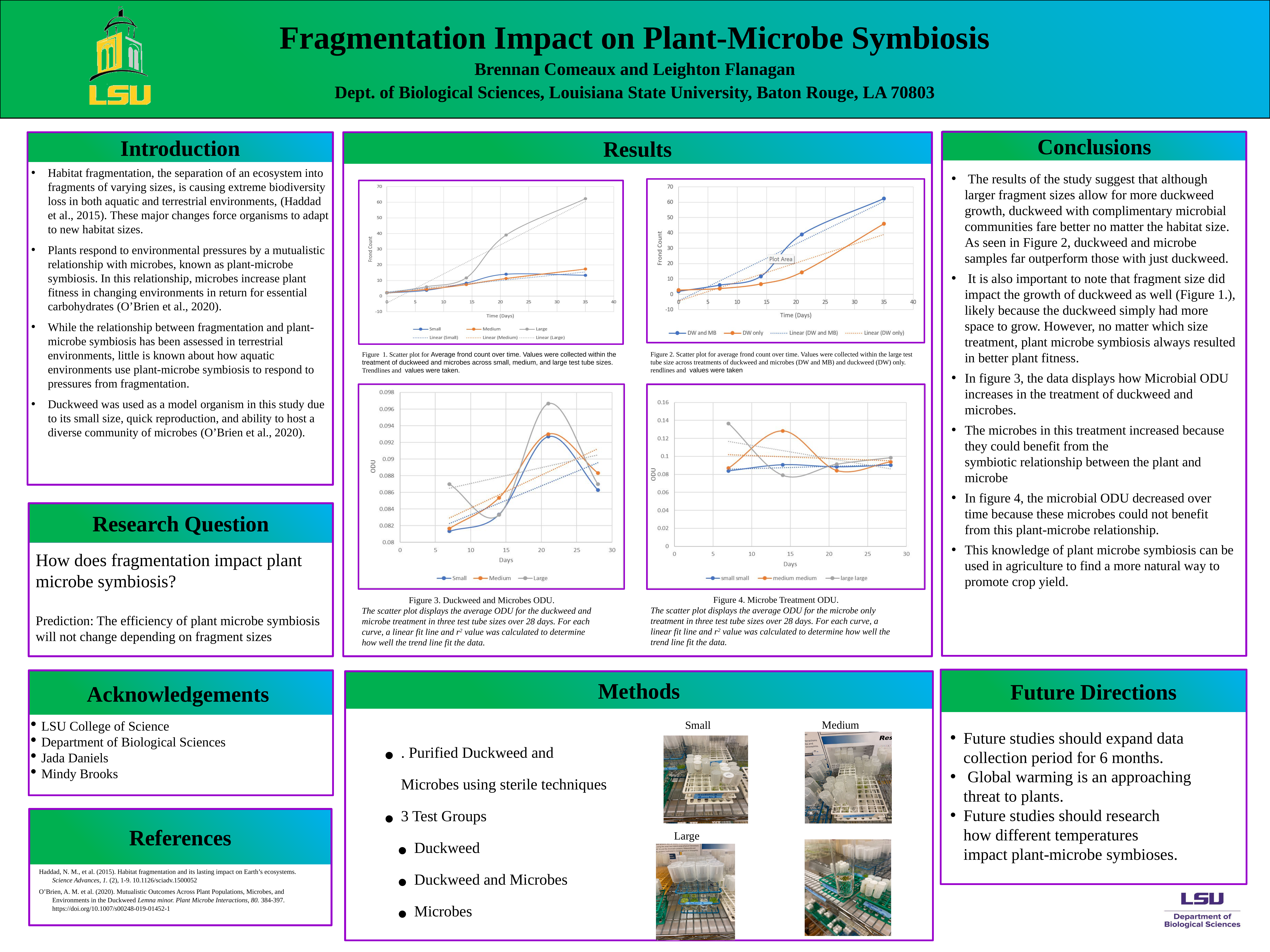

Fragmentation Impact on Plant-Microbe Symbiosis
Brennan Comeaux and Leighton Flanagan
Dept. of Biological Sciences, Louisiana State University, Baton Rouge, LA 70803
Conclusions
Results
Introduction
Habitat fragmentation, the separation of an ecosystem into fragments of varying sizes, is causing extreme biodiversity loss in both aquatic and terrestrial environments, (Haddad et al., 2015). These major changes force organisms to adapt to new habitat sizes.
Plants respond to environmental pressures by a mutualistic relationship with microbes, known as plant-microbe symbiosis. In this relationship, microbes increase plant fitness in changing environments in return for essential carbohydrates (O’Brien et al., 2020).
While the relationship between fragmentation and plant-microbe symbiosis has been assessed in terrestrial environments, little is known about how aquatic environments use plant-microbe symbiosis to respond to pressures from fragmentation.
Duckweed was used as a model organism in this study due to its small size, quick reproduction, and ability to host a diverse community of microbes (O’Brien et al., 2020).
 The results of the study suggest that although larger fragment sizes allow for more duckweed growth, duckweed with complimentary microbial communities fare better no matter the habitat size. As seen in Figure 2, duckweed and microbe samples far outperform those with just duckweed.
 It is also important to note that fragment size did impact the growth of duckweed as well (Figure 1.), likely because the duckweed simply had more space to grow. However, no matter which size treatment, plant microbe symbiosis always resulted in better plant fitness.
In figure 3, the data displays how Microbial ODU increases in the treatment of duckweed and microbes.
The microbes in this treatment increased because they could benefit from the symbiotic relationship between the plant and microbe
In figure 4, the microbial ODU decreased over time because these microbes could not benefit from this plant-microbe relationship.
This knowledge of plant microbe symbiosis can be used in agriculture to find a more natural way to promote crop yield.
B.
D.
E.
Research Question
How does fragmentation impact plant microbe symbiosis?
Prediction: The efficiency of plant microbe symbiosis will not change depending on fragment sizes
Figure 4. Microbe Treatment ODU.
The scatter plot displays the average ODU for the microbe only treatment in three test tube sizes over 28 days. For each curve, a linear fit line and r2 value was calculated to determine how well the trend line fit the data.
Figure 3. Duckweed and Microbes ODU.
The scatter plot displays the average ODU for the duckweed and microbe treatment in three test tube sizes over 28 days. For each curve, a linear fit line and r2 value was calculated to determine how well the trend line fit the data.
Future Directions
Acknowledgements
LSU College of Science
Department of Biological Sciences
Jada Daniels
Mindy Brooks
Methods
Medium
Small
Future studies should expand data collection period for 6 months.
 Global warming is an approaching threat to plants.
Future studies should research how different temperatures impact plant-microbe symbioses.
. Purified Duckweed and Microbes using sterile techniques
3 Test Groups
Duckweed
Duckweed and Microbes
Microbes
References
Large
Haddad, N. M., et al. (2015). Habitat fragmentation and its lasting impact on Earth’s ecosystems. Science Advances, 1. (2), 1-9. 10.1126/sciadv.1500052
O’Brien, A. M. et al. (2020). Mutualistic Outcomes Across Plant Populations, Microbes, and Environments in the Duckweed Lemna minor. Plant Microbe Interactions, 80. 384-397. https://doi.org/10.1007/s00248-019-01452-1
