# Supplementary material for: Introducing a Novel Course-Based Undergraduate Research Experience Using Duckweed as a Model System
Source: Integr Org Biol. 2025 Dec 19;8(1):obaf049. doi: 10.1093/iob/obaf049 (PMC12802901; doi:10.1093/iob/obaf049)
Supplement: obaf049_Supplemental_Files [file obaf049_supplemental_files.zip › 07 Supplementary Materials/Supplementary Materials/58_ARTIFACT_PosterFall23.pptx]

## Slide 1
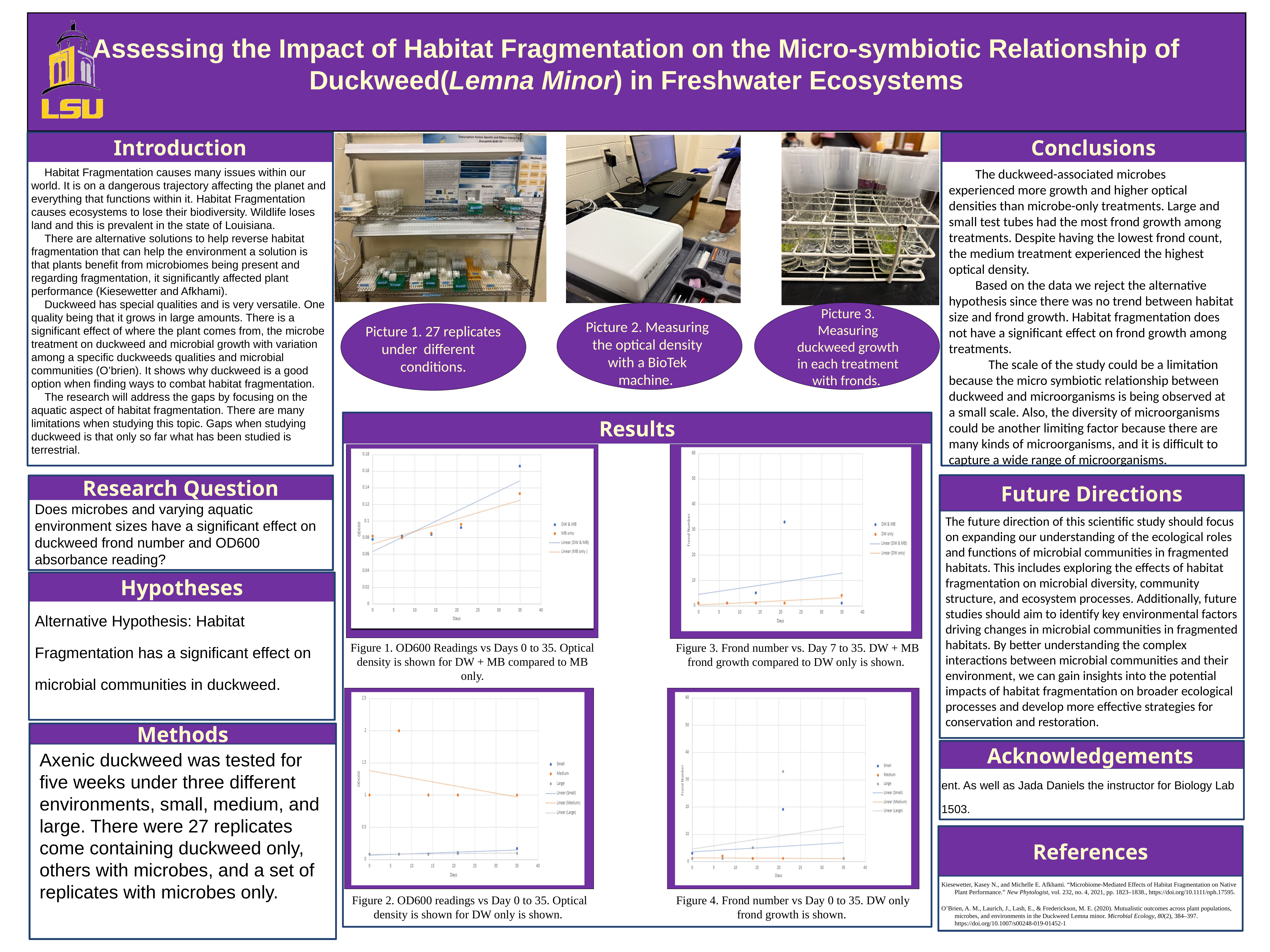

Assessing the Impact of Habitat Fragmentation on the Micro-symbiotic Relationship of Duckweed(Lemna Minor) in Freshwater Ecosystems
Introduction
Conclusions
Habitat Fragmentation causes many issues within our world. It is on a dangerous trajectory affecting the planet and everything that functions within it. Habitat Fragmentation causes ecosystems to lose their biodiversity. Wildlife loses land and this is prevalent in the state of Louisiana.
There are alternative solutions to help reverse habitat fragmentation that can help the environment a solution is that plants benefit from microbiomes being present and regarding fragmentation, it significantly affected plant performance (Kiesewetter and Afkhami).
Duckweed has special qualities and is very versatile. One quality being that it grows in large amounts. There is a significant effect of where the plant comes from, the microbe treatment on duckweed and microbial growth with variation among a specific duckweeds qualities and microbial communities (O’brien). It shows why duckweed is a good option when finding ways to combat habitat fragmentation.
The research will address the gaps by focusing on the aquatic aspect of habitat fragmentation. There are many limitations when studying this topic. Gaps when studying duckweed is that only so far what has been studied is terrestrial.
	The duckweed-associated microbes experienced more growth and higher optical densities than microbe-only treatments. Large and small test tubes had the most frond growth among treatments. Despite having the lowest frond count, the medium treatment experienced the highest optical density.
	Based on the data we reject the alternative hypothesis since there was no trend between habitat size and frond growth. Habitat fragmentation does not have a significant effect on frond growth among treatments.
      The scale of the study could be a limitation because the micro symbiotic relationship between duckweed and microorganisms is being observed at a small scale. Also, the diversity of microorganisms could be another limiting factor because there are many kinds of microorganisms, and it is difficult to capture a wide range of microorganisms.
B.
A.
D.
Picture 3. Measuring duckweed growth in each treatment with fronds.
Picture 2. Measuring the optical density with a BioTek machine.
Picture 1. 27 replicates under different conditions.
E.
Results
Research Question
Future Directions
Does microbes and varying aquatic environment sizes have a significant effect on duckweed frond number and OD600 absorbance reading?
The future direction of this scientific study should focus on expanding our understanding of the ecological roles and functions of microbial communities in fragmented habitats. This includes exploring the effects of habitat fragmentation on microbial diversity, community structure, and ecosystem processes. Additionally, future studies should aim to identify key environmental factors driving changes in microbial communities in fragmented habitats. By better understanding the complex interactions between microbial communities and their environment, we can gain insights into the potential impacts of habitat fragmentation on broader ecological processes and develop more effective strategies for conservation and restoration.
Hypotheses
Alternative Hypothesis: Habitat Fragmentation has a significant effect on microbial communities in duckweed.
Figure 1. OD600 Readings vs Days 0 to 35. Optical density is shown for DW + MB compared to MB only.
Figure 3. Frond number vs. Day 7 to 35. DW + MB frond growth compared to DW only is shown.
Methods
Acknowledgements
ent. As well as Jada Daniels the instructor for Biology Lab 1503.
Axenic duckweed was tested for five weeks under three different environments, small, medium, and large. There were 27 replicates come containing duckweed only, others with microbes, and a set of replicates with microbes only.
References
Kiesewetter, Kasey N., and Michelle E. Afkhami. “Microbiome‐Mediated Effects of Habitat Fragmentation on Native Plant Performance.” New Phytologist, vol. 232, no. 4, 2021, pp. 1823–1838., https://doi.org/10.1111/nph.17595.
O’Brien, A. M., Laurich, J., Lash, E., & Frederickson, M. E. (2020). Mutualistic outcomes across plant populations, microbes, and environments in the Duckweed Lemna minor. Microbial Ecology, 80(2), 384–397. https://doi.org/10.1007/s00248-019-01452-1
Figure 4. Frond number vs Day 0 to 35. DW only frond growth is shown.
Figure 2. OD600 readings vs Day 0 to 35. Optical density is shown for DW only is shown.

## Slide 2
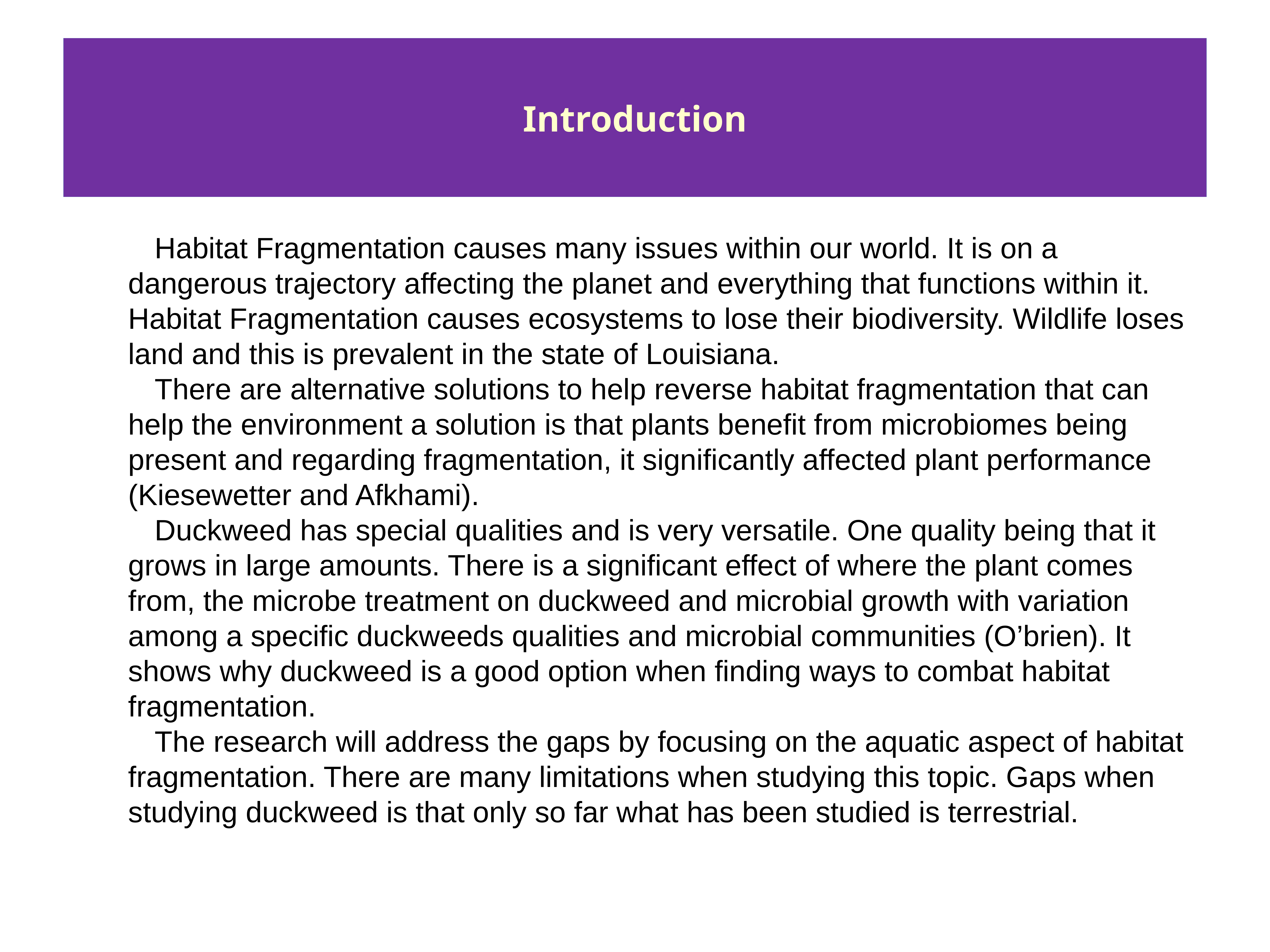

# Introduction
	Habitat Fragmentation causes many issues within our world. It is on a dangerous trajectory affecting the planet and everything that functions within it. Habitat Fragmentation causes ecosystems to lose their biodiversity. Wildlife loses land and this is prevalent in the state of Louisiana.
 	There are alternative solutions to help reverse habitat fragmentation that can help the environment a solution is that plants benefit from microbiomes being present and regarding fragmentation, it significantly affected plant performance (Kiesewetter and Afkhami).
	Duckweed has special qualities and is very versatile. One quality being that it grows in large amounts. There is a significant effect of where the plant comes from, the microbe treatment on duckweed and microbial growth with variation among a specific duckweeds qualities and microbial communities (O’brien). It shows why duckweed is a good option when finding ways to combat habitat fragmentation.
	The research will address the gaps by focusing on the aquatic aspect of habitat fragmentation. There are many limitations when studying this topic. Gaps when studying duckweed is that only so far what has been studied is terrestrial.

## Slide 3
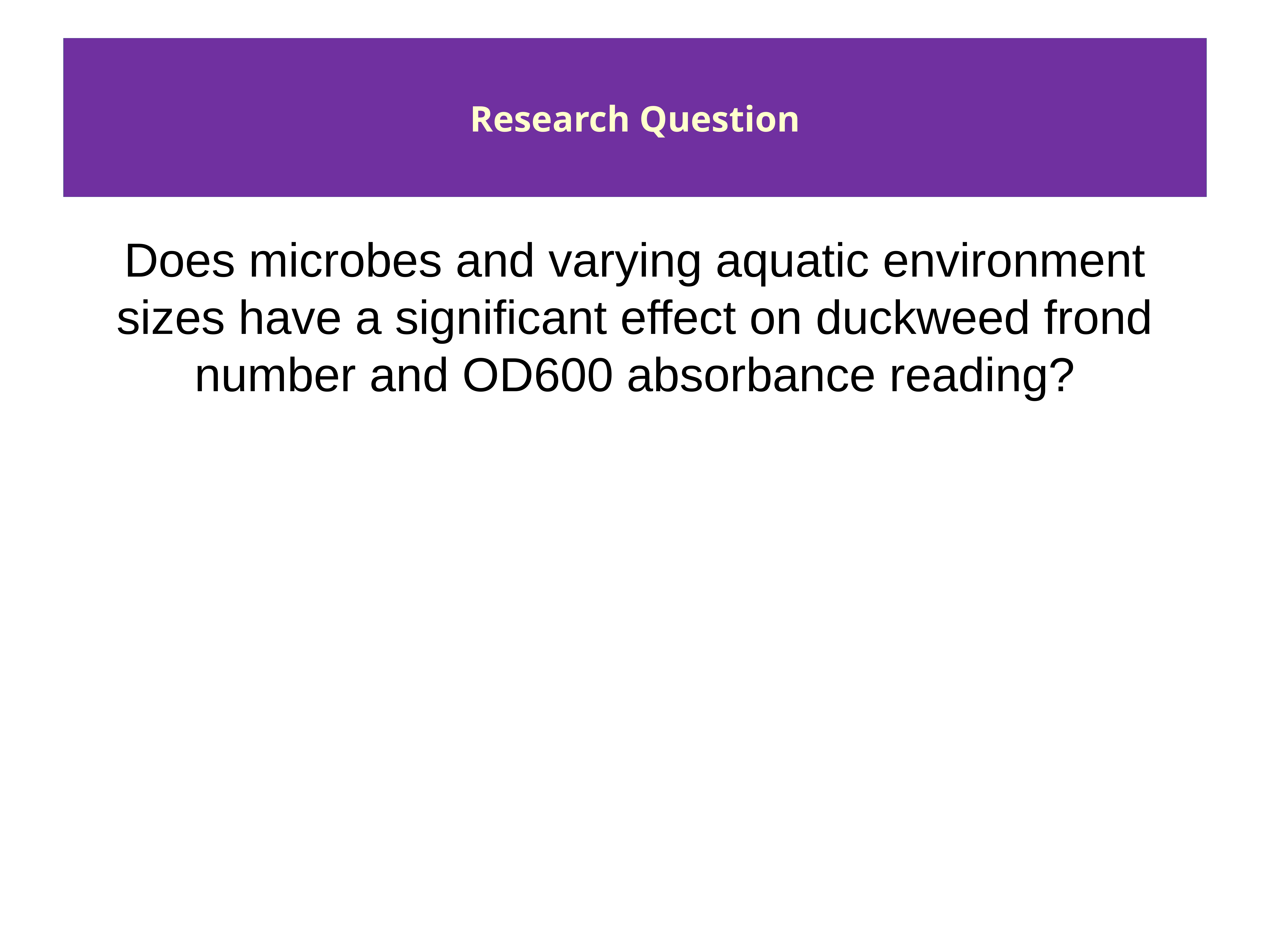

# Research Question
Does microbes and varying aquatic environment sizes have a significant effect on duckweed frond number and OD600 absorbance reading?

## Slide 4
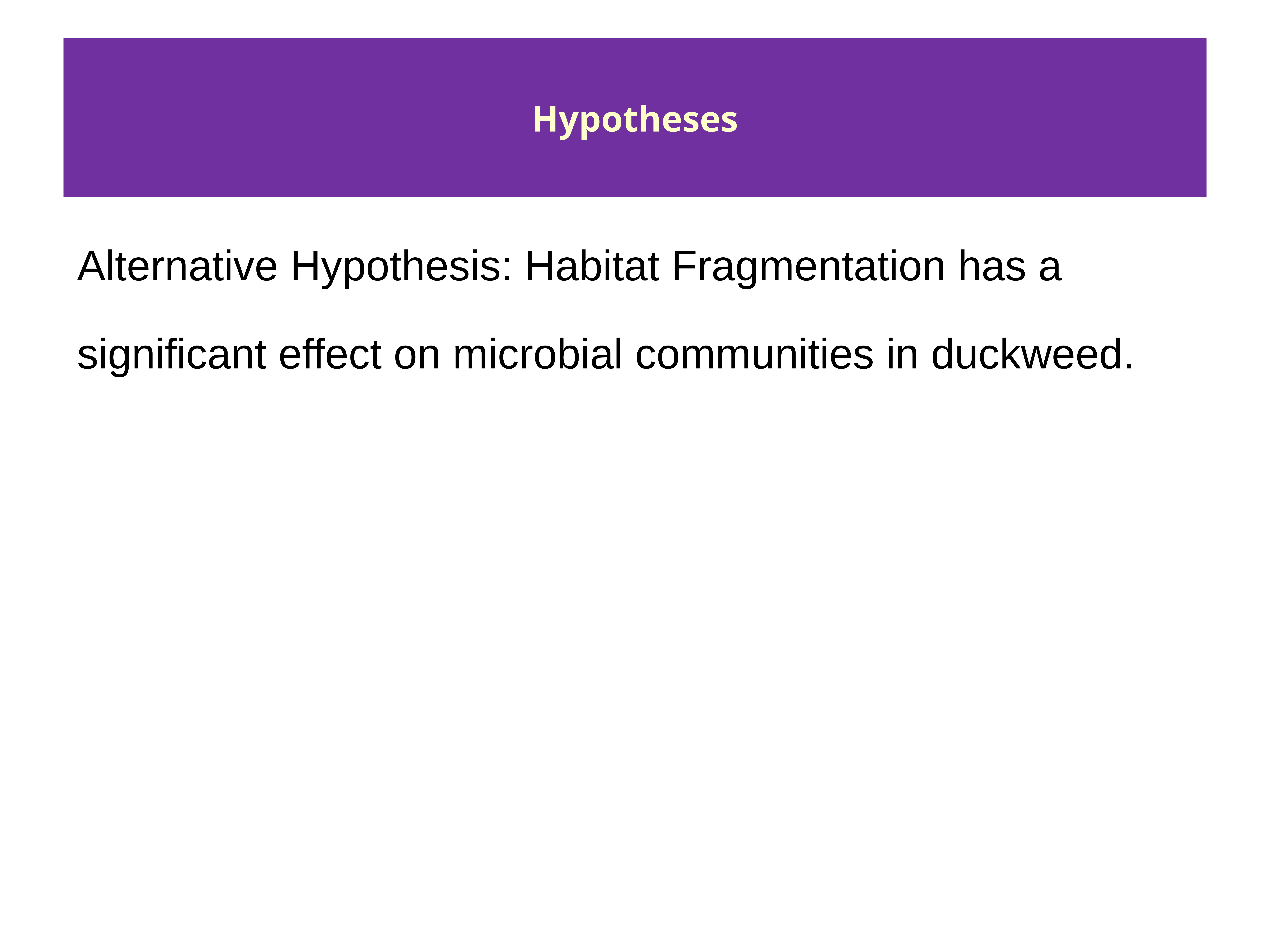

# Hypotheses
Alternative Hypothesis: Habitat Fragmentation has a significant effect on microbial communities in duckweed.

## Slide 5
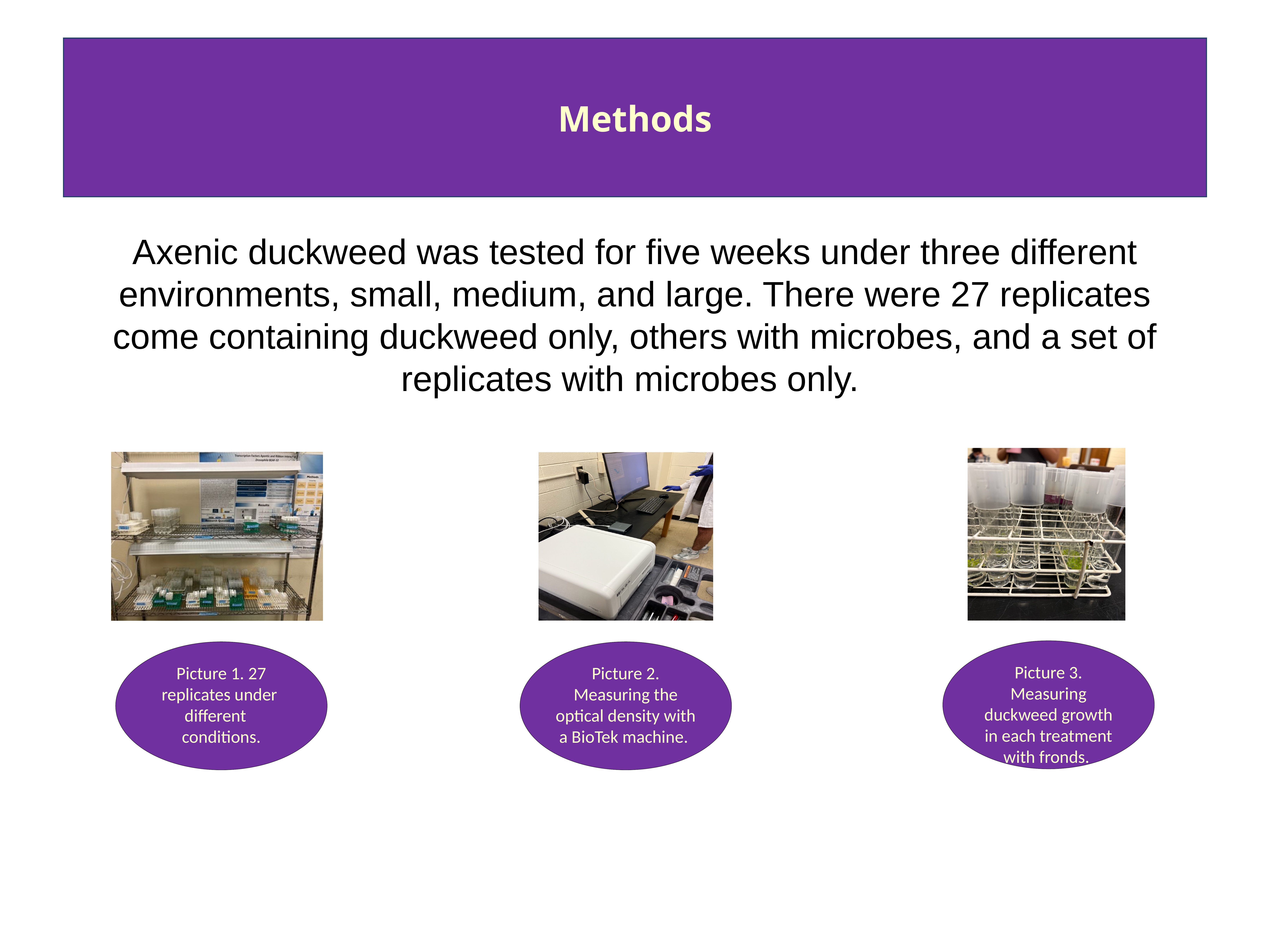

# Methods
Axenic duckweed was tested for five weeks under three different environments, small, medium, and large. There were 27 replicates come containing duckweed only, others with microbes, and a set of replicates with microbes only.
Picture 3. Measuring duckweed growth in each treatment with fronds.
Picture 1. 27 replicates under different conditions.
Picture 2. Measuring the optical density with a BioTek machine.

## Slide 6
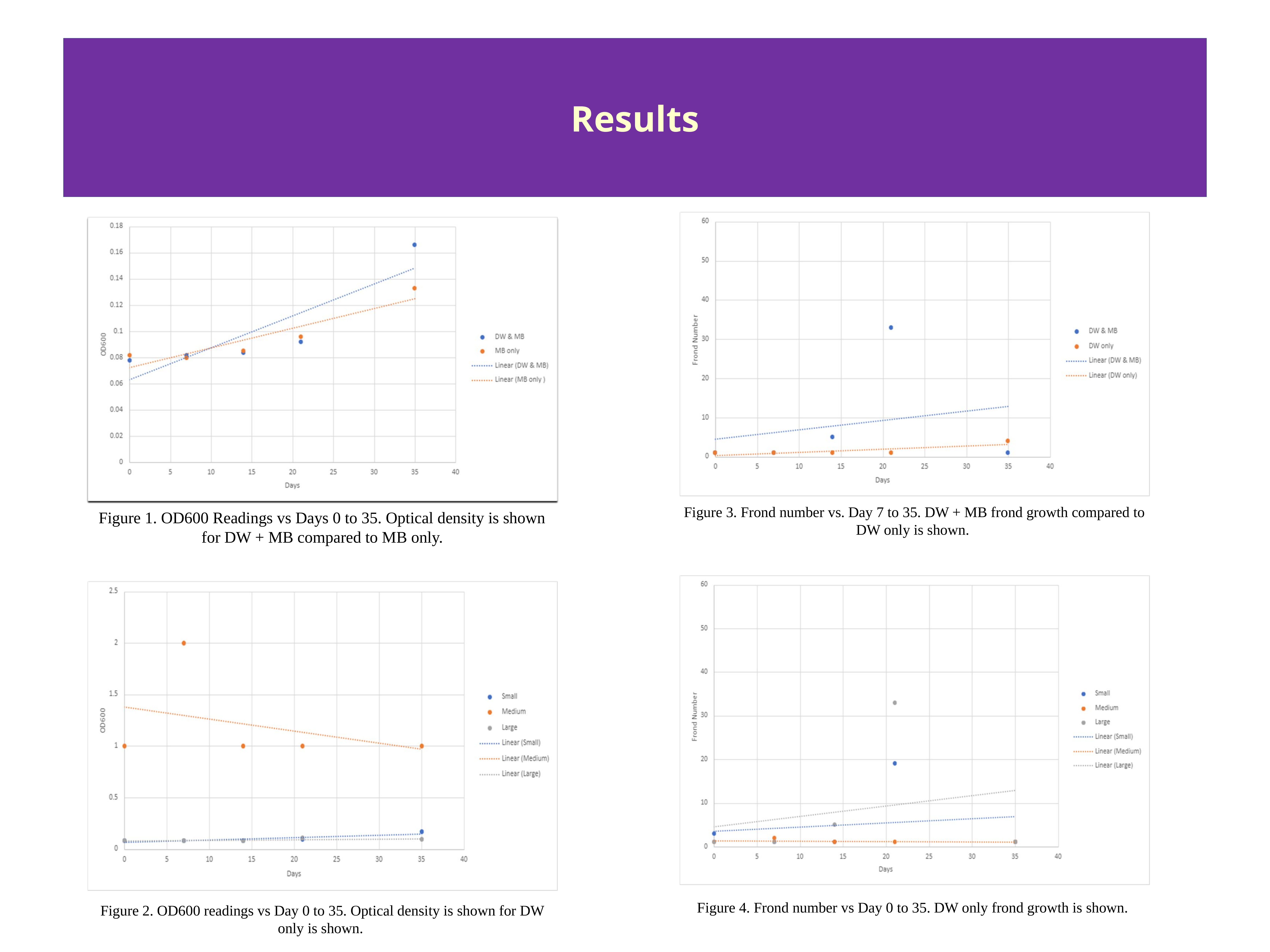

# Results
Figure 3. Frond number vs. Day 7 to 35. DW + MB frond growth compared to DW only is shown.
Figure 1. OD600 Readings vs Days 0 to 35. Optical density is shown for DW + MB compared to MB only.
Figure 4. Frond number vs Day 0 to 35. DW only frond growth is shown.
Figure 2. OD600 readings vs Day 0 to 35. Optical density is shown for DW only is shown.

## Slide 7
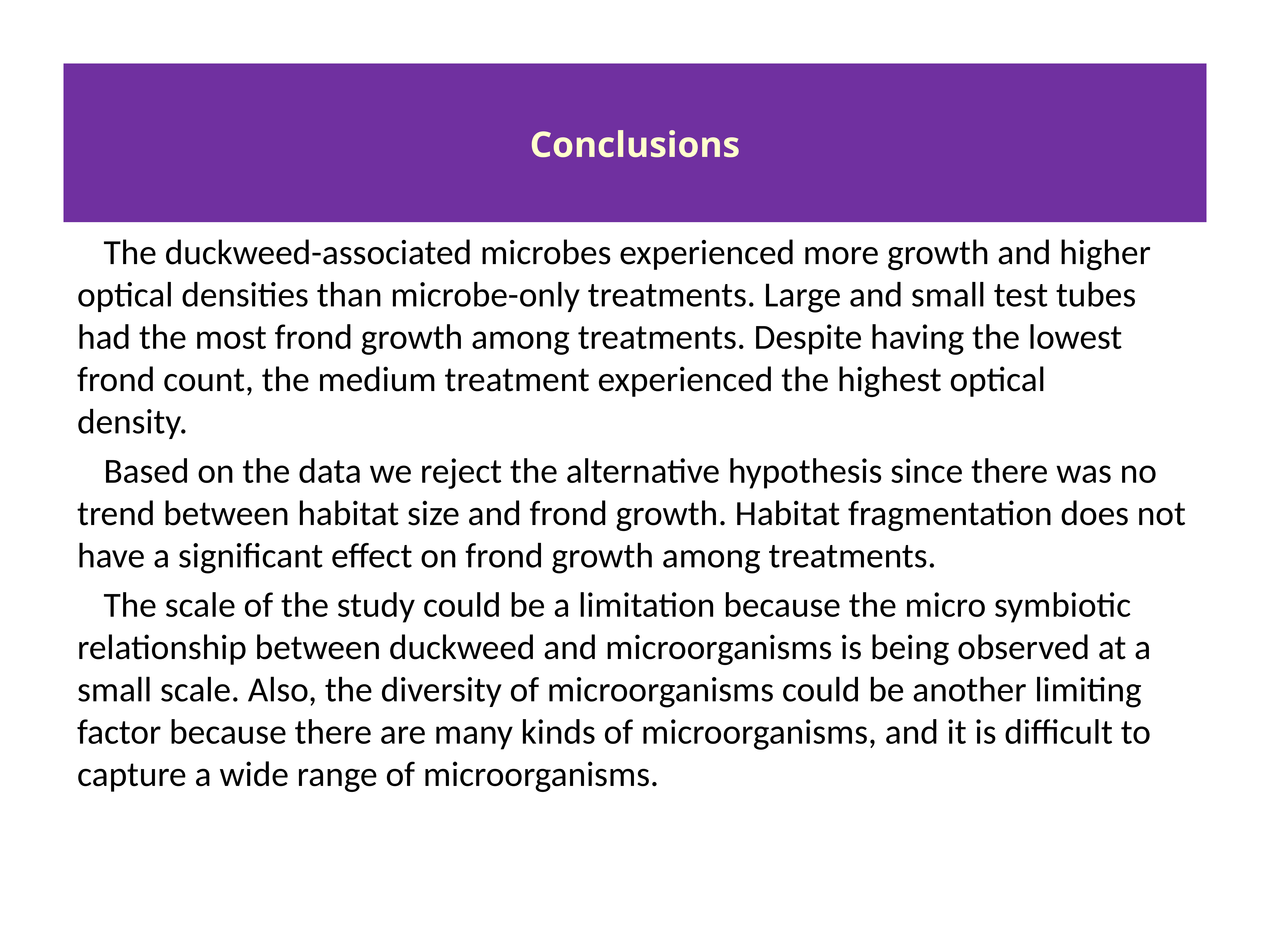

# Conclusions
	The duckweed-associated microbes experienced more growth and higher optical densities than microbe-only treatments. Large and small test tubes had the most frond growth among treatments. Despite having the lowest frond count, the medium treatment experienced the highest optical density.
	Based on the data we reject the alternative hypothesis since there was no trend between habitat size and frond growth. Habitat fragmentation does not have a significant effect on frond growth among treatments.
	The scale of the study could be a limitation because the micro symbiotic relationship between duckweed and microorganisms is being observed at a small scale. Also, the diversity of microorganisms could be another limiting factor because there are many kinds of microorganisms, and it is difficult to capture a wide range of microorganisms.

## Slide 8
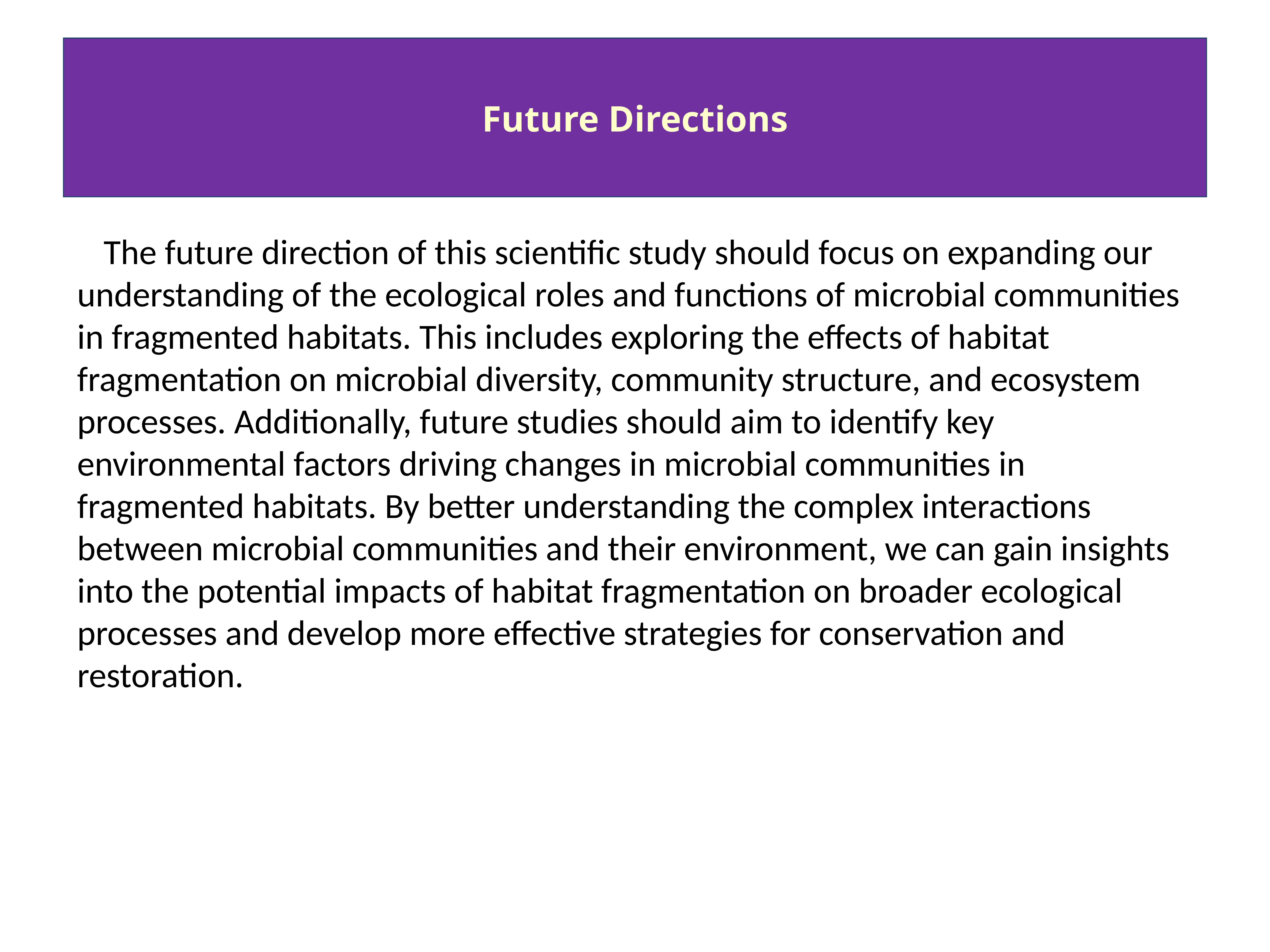

# Future Directions
	The future direction of this scientific study should focus on expanding our understanding of the ecological roles and functions of microbial communities in fragmented habitats. This includes exploring the effects of habitat fragmentation on microbial diversity, community structure, and ecosystem processes. Additionally, future studies should aim to identify key environmental factors driving changes in microbial communities in fragmented habitats. By better understanding the complex interactions between microbial communities and their environment, we can gain insights into the potential impacts of habitat fragmentation on broader ecological processes and develop more effective strategies for conservation and restoration.

## Slide 9
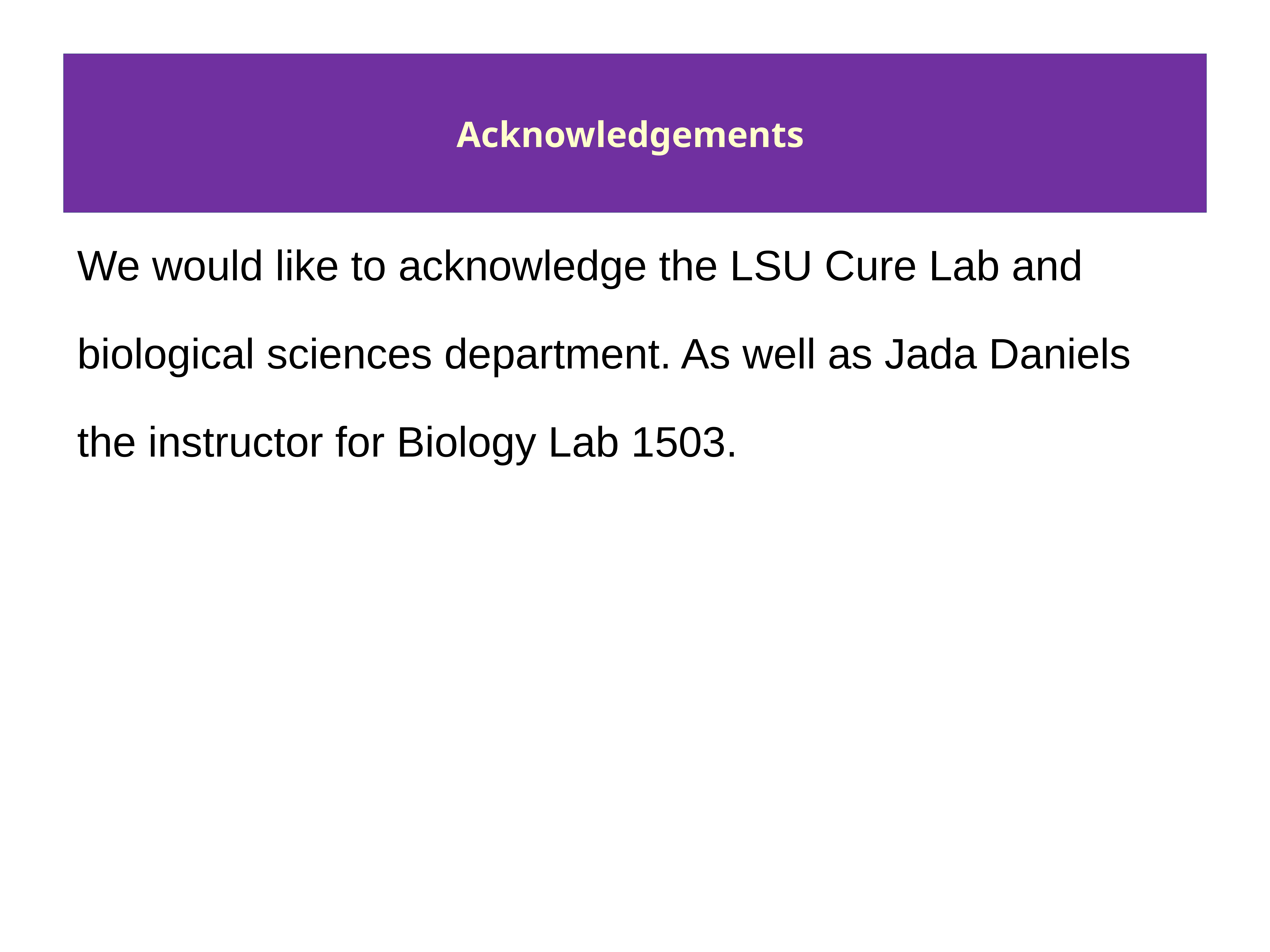

# Acknowledgements
We would like to acknowledge the LSU Cure Lab and biological sciences department. As well as Jada Daniels the instructor for Biology Lab 1503.

## Slide 10
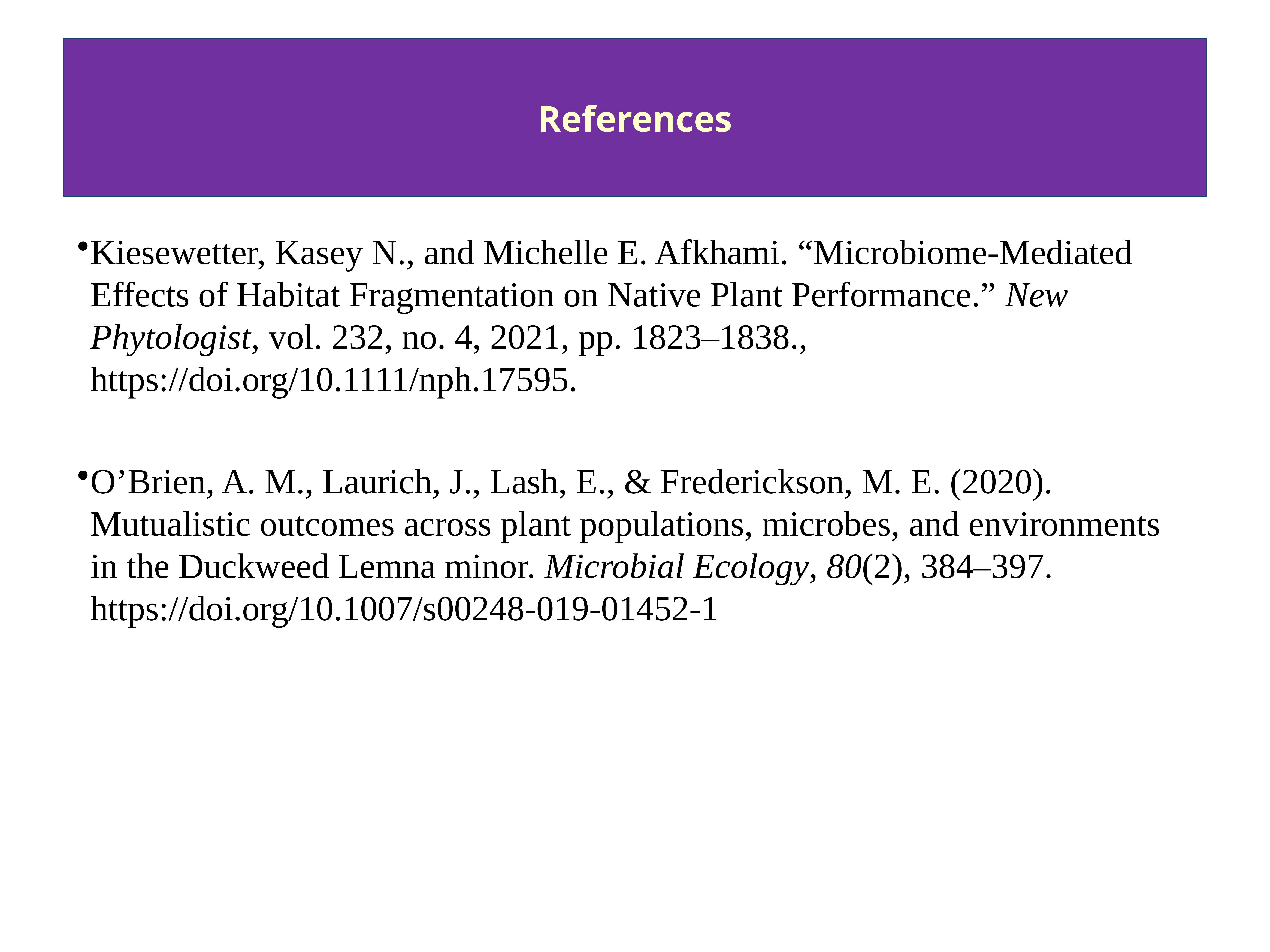

# References
Kiesewetter, Kasey N., and Michelle E. Afkhami. “Microbiome‐Mediated Effects of Habitat Fragmentation on Native Plant Performance.” New Phytologist, vol. 232, no. 4, 2021, pp. 1823–1838., https://doi.org/10.1111/nph.17595.
O’Brien, A. M., Laurich, J., Lash, E., & Frederickson, M. E. (2020). Mutualistic outcomes across plant populations, microbes, and environments in the Duckweed Lemna minor. Microbial Ecology, 80(2), 384–397. https://doi.org/10.1007/s00248-019-01452-1

## Slide 11
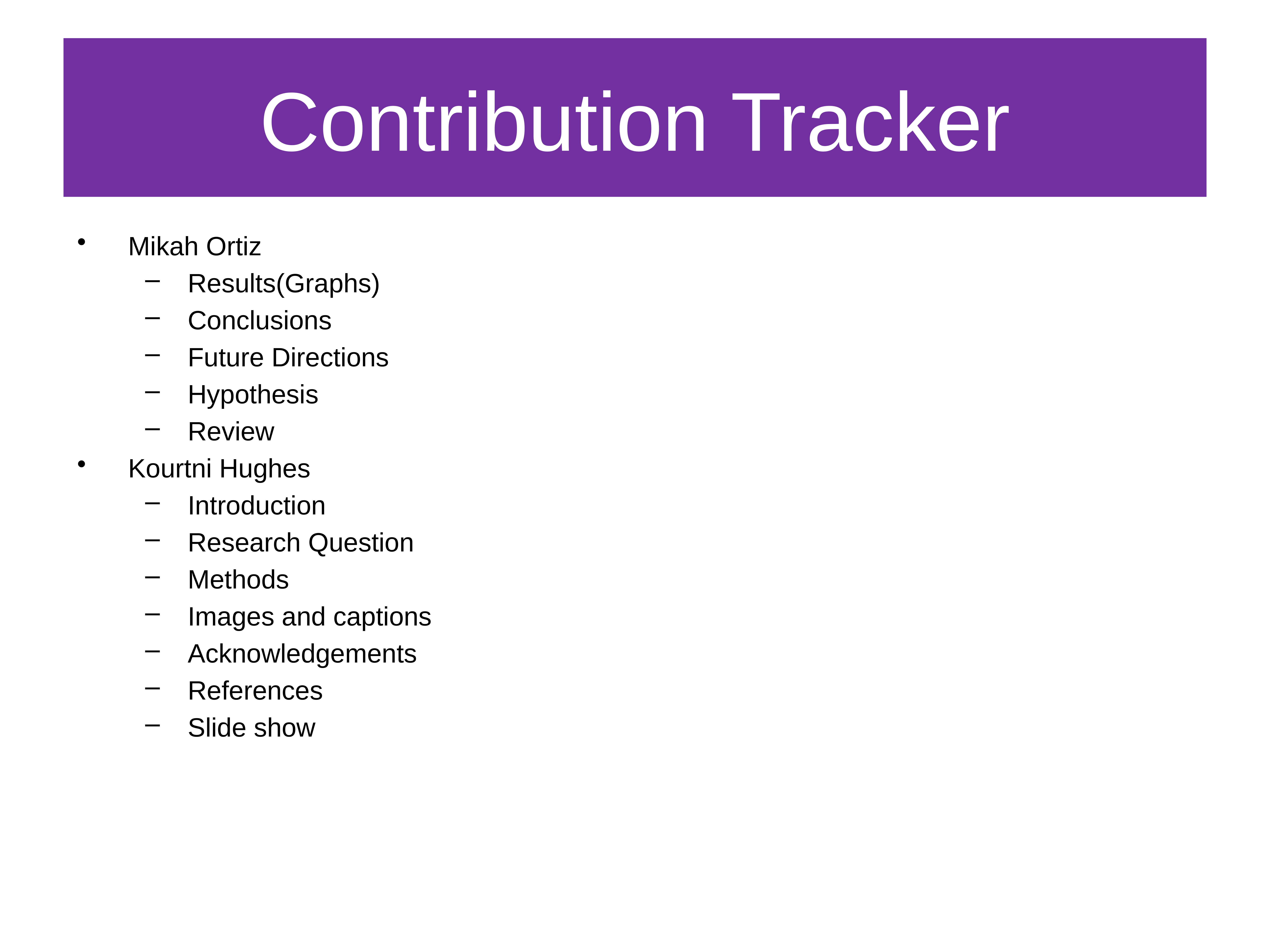

# Contribution Tracker
Mikah Ortiz
Results(Graphs)
Conclusions
Future Directions
Hypothesis
Review
Kourtni Hughes
Introduction
Research Question
Methods
Images and captions
Acknowledgements
References
Slide show
