# Supplementary material for: Introducing a Novel Course-Based Undergraduate Research Experience Using Duckweed as a Model System
Source: Integr Org Biol. 2025 Dec 19;8(1):obaf049. doi: 10.1093/iob/obaf049 (PMC12802901; doi:10.1093/iob/obaf049)
Supplement: obaf049_Supplemental_Files [file obaf049_supplemental_files.zip › 07 Supplementary Materials/Supplementary Materials/59_ARTIFACT_PosterSpring24.pptx]

## Slide 1
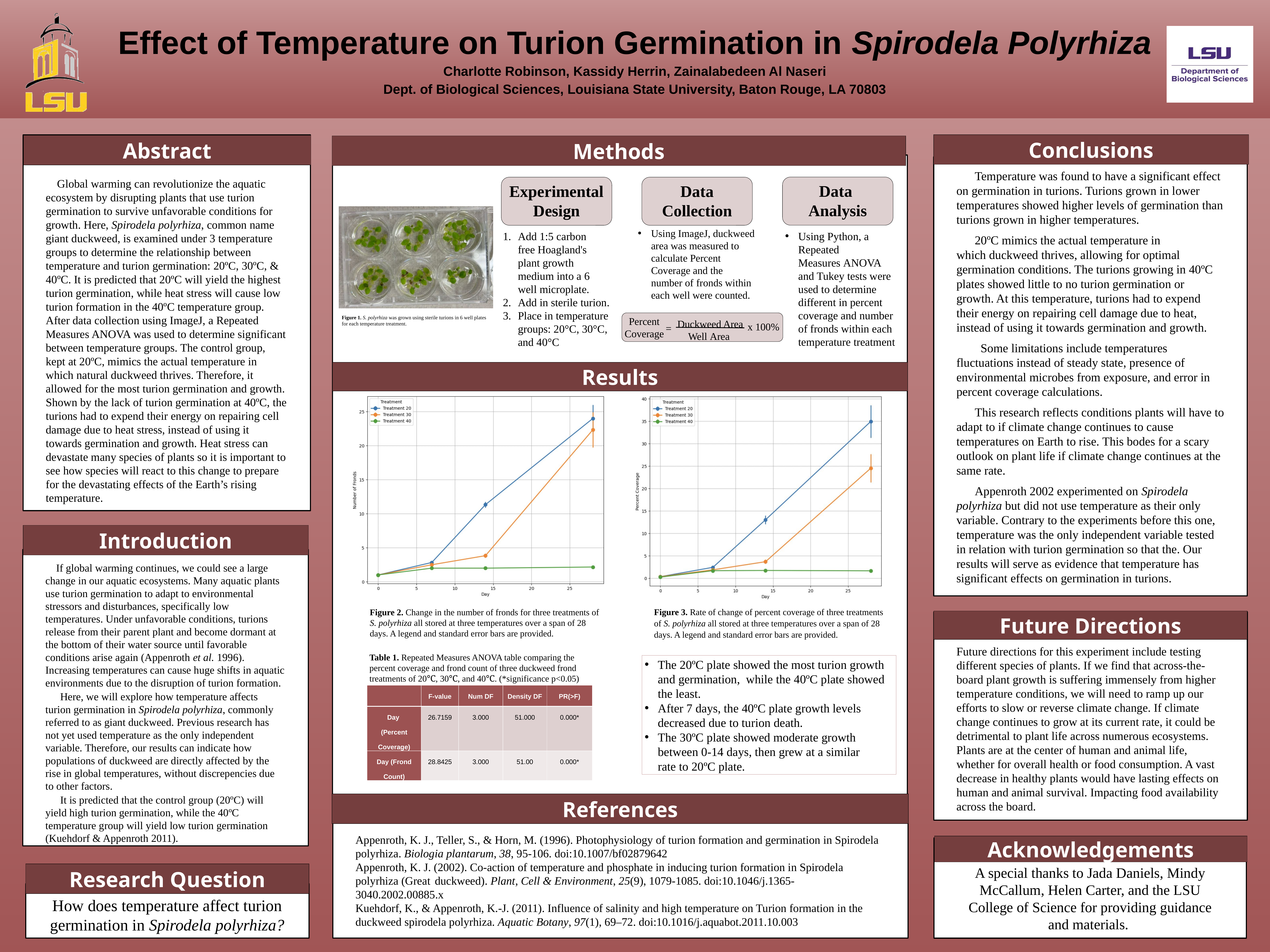

Effect of Temperature on Turion Germination in Spirodela Polyrhiza
Charlotte Robinson, Kassidy Herrin, Zainalabedeen Al Naseri
Dept. of Biological Sciences, Louisiana State University, Baton Rouge, LA 70803
Conclusions
 Global warming can revolutionize the aquatic ecosystem by disrupting plants that use turion germination to survive unfavorable conditions for growth. Here, Spirodela polyrhiza, common name giant duckweed, is examined under 3 temperature groups to determine the relationship between temperature and turion germination: 20ºC, 30ºC, & 40ºC. It is predicted that 20ºC will yield the highest turion germination, while heat stress will cause low turion formation in the 40ºC temperature group. After data collection using ImageJ, a Repeated Measures ANOVA was used to determine significant between temperature groups. The control group, kept at 20ºC, mimics the actual temperature in which natural duckweed thrives. Therefore, it allowed for the most turion germination and growth. Shown by the lack of turion germination at 40ºC, the turions had to expend their energy on repairing cell damage due to heat stress, instead of using it towards germination and growth. Heat stress can devastate many species of plants so it is important to see how species will react to this change to prepare for the devastating effects of the Earth’s rising temperature.
Abstract
Methods
	Temperature was found to have a significant effect on germination in turions. Turions grown in lower temperatures showed higher levels of germination than turions grown in higher temperatures.
	20ºC mimics the actual temperature in which duckweed thrives, allowing for optimal germination conditions. The turions growing in 40ºC plates showed little to no turion germination or growth. At this temperature, turions had to expend their energy on repairing cell damage due to heat, instead of using it towards germination and growth.
  Some limitations include temperatures fluctuations instead of steady state, presence of environmental microbes from exposure, and error in percent coverage calculations.
	This research reflects conditions plants will have to adapt to if climate change continues to cause temperatures on Earth to rise. This bodes for a scary outlook on plant life if climate change continues at the same rate.
	Appenroth 2002 experimented on Spirodela polyrhiza but did not use temperature as their only variable. Contrary to the experiments before this one, temperature was the only independent variable tested in relation with turion germination so that the. Our results will serve as evidence that temperature has significant effects on germination in turions.
Data
Analysis
Data Collection
Experimental Design
Using ImageJ, duckweed area was measured to calculate Percent Coverage and the number of fronds within each well were counted.
Using Python, a Repeated Measures ANOVA and Tukey tests were used to determine different in percent coverage and number of fronds within each temperature treatment
Add 1:5 carbon free Hoagland's plant growth medium into a 6 well microplate.
Add in sterile turion.
Place in temperature groups: 20°C, 30°C, and 40°C
Figure 1. S. polyrhiza was grown using sterile turions in 6 well plates for each temperature treatment.
Percent
Coverage
Duckweed Area
Well Area
x 100%
=
Results
Introduction
 If global warming continues, we could see a large change in our aquatic ecosystems. Many aquatic plants use turion germination to adapt to environmental stressors and disturbances, specifically low temperatures. Under unfavorable conditions, turions release from their parent plant and become dormant at the bottom of their water source until favorable conditions arise again (Appenroth et al. 1996). Increasing temperatures can cause huge shifts in aquatic environments due to the disruption of turion formation.
	Here, we will explore how temperature affects turion germination in Spirodela polyrhiza, commonly referred to as giant duckweed. Previous research has not yet used temperature as the only independent variable. Therefore, our results can indicate how populations of duckweed are directly affected by the rise in global temperatures, without discrepencies due to other factors.
	It is predicted that the control group (20ºC) will yield high turion germination, while the 40ºC temperature group will yield low turion germination (Kuehdorf & Appenroth 2011).
Figure 3. Rate of change of percent coverage of three treatments of S. polyrhiza all stored at three temperatures over a span of 28 days. A legend and standard error bars are provided.
Figure 2. Change in the number of fronds for three treatments of S. polyrhiza all stored at three temperatures over a span of 28 days. A legend and standard error bars are provided.
Future Directions
Future directions for this experiment include testing different species of plants. If we find that across-the-board plant growth is suffering immensely from higher temperature conditions, we will need to ramp up our efforts to slow or reverse climate change. If climate change continues to grow at its current rate, it could be detrimental to plant life across numerous ecosystems. Plants are at the center of human and animal life, whether for overall health or food consumption. A vast decrease in healthy plants would have lasting effects on human and animal survival. Impacting food availability across the board.
Table 1. Repeated Measures ANOVA table comparing the percent coverage and frond count of three duckweed frond treatments of 20℃, 30℃, and 40℃. (*significance p<0.05)
The 20ºC plate showed the most turion growth and germination,  while the 40ºC plate showed the least.
After 7 days, the 40ºC plate growth levels decreased due to turion death.
The 30ºC plate showed moderate growth between 0-14 days, then grew at a similar rate to 20ºC plate.
| | F-value | Num DF | Density DF | PR(>F) |
| --- | --- | --- | --- | --- |
| Day  (Percent Coverage) | 26.7159 | 3.000 | 51.000 | 0.000\* |
| Day (Frond Count) | 28.8425 | 3.000 | 51.00 | 0.000\* |
References
Appenroth, K. J., Teller, S., & Horn, M. (1996). Photophysiology of turion formation and germination in Spirodela 	polyrhiza. Biologia plantarum, 38, 95-106. doi:10.1007/bf02879642
Appenroth, K. J. (2002). Co‐action of temperature and phosphate in inducing turion formation in Spirodela 	polyrhiza (Great 	duckweed). Plant, Cell & Environment, 25(9), 1079-1085. doi:10.1046/j.1365-	3040.2002.00885.x
Kuehdorf, K., & Appenroth, K.-J. (2011). Influence of salinity and high temperature on Turion formation in the 	duckweed spirodela polyrhiza. Aquatic Botany, 97(1), 69–72. doi:10.1016/j.aquabot.2011.10.003
Acknowledgements
A special thanks to Jada Daniels, Mindy McCallum, Helen Carter, and the LSU College of Science for providing guidance and materials.
Research Question
How does temperature affect turion germination in Spirodela polyrhiza?
