# Supplementary material for: Introducing a Novel Course-Based Undergraduate Research Experience Using Duckweed as a Model System
Source: Integr Org Biol. 2025 Dec 19;8(1):obaf049. doi: 10.1093/iob/obaf049 (PMC12802901; doi:10.1093/iob/obaf049)
Supplement: obaf049_Supplemental_Files [file obaf049_supplemental_files.zip › 07 Supplementary Materials/Supplementary Materials/60_ARTIFACT_TableS1.docx]

**Table S1**. Summary of student-generated statistical analyses across three semesters of the Duckweed CURE. Each semester addressed a distinct ecological question using duckweed (Lemna minor or Spirodela polyrhiza) and associated microbial communities. Reported values represent student-conducted analyses based on class datasets. Statistical tests (ANOVA and repeated-measures ANOVA) were chosen for their accessibility in an introductory biology context and are presented here to illustrate the scope and interpretation of student-generated results rather than to draw formal statistical conclusions.

| **Semester** | **Experimental Focus** | **Dependent Variable(s)** | **Independent Variable(s)** | **Statistical Test** | **Statistical Results** | **Interpretation** | **Figure Reference** |
| --- | --- | --- | --- | --- | --- | --- | --- |
| **Spring 2023** | Effect of habitat size on duckweed–microbe interactions | Duckweed frond count; microbial density (OD600) | Habitat size (small, medium, large) | One-way ANOVA with post-hoc Tukey tests | **Frond count:** F = 23.97, *p* < 0.001; large > small (*p* < 0.001), large > medium (*p* < 0.001), small ≈ medium (*p* = 0.950). **OD600:** F = 2.32, *p* = 0.102. | Duckweed growth increased with habitat size; microbial density did not differ among treatments. | Fig. 1a–b |
| **Fall 2023** | Effect of temperature on *Lemna minor* and its microbial community | Duckweed frond count; microbial density (OD600) | Temperature (20 °C, 30 °C, 40 °C); Time (weeks) | Repeated-measures ANOVA | **Frond count:** F = 1.22, *p* = 0.322; **Time:** F = 1.34, *p* = 0.301; **Interaction:** F = 0.29, *p* = 0.958. **OD600:** F = 0.94, *p* = 0.419; **Interaction:** F = 0.57, *p* = 0.750. | No significant effects of temperature or time on growth or microbial density; plants at 40 °C survived but did not form new fronds. | Fig. 2a–b |
| **Spring 2024** | Effect of temperature on *Spirodela polyrhiza* turion germination | Germination rate; frond count; surface coverage | Temperature (20 °C, 30 °C, 40 °C) | One-way ANOVA | **Percent coverage:** F = 58.63, p < 0.001; 30 °C > 40 °C (p < 0.001), 20 °C > 40 °C (p < 0.001), 20 °C ≈ 30 °C (p = 0.912). | Temperature increased germination rate and surface coverage in axenic cultures. | Fig. 3 |
