# Supplementary material for: Introducing a Novel Course-Based Undergraduate Research Experience Using Duckweed as a Model System
Source: Integr Org Biol. 2025 Dec 19;8(1):obaf049. doi: 10.1093/iob/obaf049 (PMC12802901; doi:10.1093/iob/obaf049)
Supplement: obaf049_Supplemental_Files [file obaf049_supplemental_files.zip › 07 Supplementary Materials/Supplementary Materials/61_ARTIFACT_TableS2.docx]

# Supplementary Tables: Student Statistical Analyses

## Table 1. Summary of student-generated results from the Spring 2023 Duckweed CURE experiment examining habitat size effects on duckweed–microbe interactions.

| Variable Measured | Independent Variable | Statistical Test | Results (F, p) | Interpretation | Figure |
| --- | --- | --- | --- | --- | --- |
| Duckweed frond count | Habitat size (small, medium, large) | One-way ANOVA with post-hoc Tukey tests | F = 23.97, p < 0.001 | Duckweed growth increased with habitat size; large habitats supported significantly greater frond production than small or medium habitats. | Fig. 1a |
| Microbial density (OD₆₀₀) | Habitat size (small, medium, large) | One-way ANOVA | F = 2.32, p = 0.102 | Microbial density did not differ significantly across habitat sizes, suggesting stability in microbial abundance across treatments. | Fig. 1b |

## Table 2. Summary of student-generated results from the Fall 2023 Duckweed CURE experiment examining temperature effects on Lemna minor and its microbial community.

| Variable Measured | Independent Variable | Statistical Test | Results (F, p) | Interpretation | Figure |
| --- | --- | --- | --- | --- | --- |
| Duckweed frond count | Temperature (20°C, 30°C, 40°C); Time (weeks) | Repeated-measures ANOVA | F = 1.22, p = 0.322 (treatment); F = 1.34, p = 0.301 (time); F = 0.29, p = 0.958 (interaction) | Duckweed growth did not differ significantly among temperature treatments; plants at 40°C survived but produced fewer fronds. | Fig. 2a |
| Microbial density (OD₆₀₀) | Temperature (20°C, 30°C, 40°C); Time (weeks) | Repeated-measures ANOVA | F = 0.94, p = 0.419 (treatment); F = 0.57, p = 0.750 (interaction) | Microbial density remained consistent across temperatures, indicating short-term thermal resilience of the microbial community. | Fig. 2b |

## Table 3. Summary of student-generated results from the Spring 2024 Duckweed CURE experiment examining temperature effects on Spirodela polyrhiza turion germination.

| Variable Measured | Independent Variable | Statistical Test | Results (F, p) | Interpretation | Figure |
| --- | --- | --- | --- | --- | --- |
| Surface coverage / Frond count | Temperature (20°C, 30°C, 40°C) | One-way ANOVA | Percent coverage: F = 58.63, p < 0.001; 30 °C > 40 °C (p < 0.001), 20 °C > 40 °C (p < 0.001), 20 °C ≈ 30 °C (p = 0.912). | Growth and surface coverage increased with temperature under axenic conditions, indicating enhanced physiological response at higher temperatures. | Fig. 3 |
