# Supplementary material for: Introducing a Novel Course-Based Undergraduate Research Experience Using Duckweed as a Model System
Source: Integr Org Biol. 2025 Dec 19;8(1):obaf049. doi: 10.1093/iob/obaf049 (PMC12802901; doi:10.1093/iob/obaf049)
Supplement: obaf049_Supplemental_Files [file obaf049_supplemental_files.zip › 07 Supplementary Materials/Supplementary Materials/35_Week06_ICA_PeerReviewGuidelinesAndPeerMark.docx]

# ICA: Peer Review Guidelines for FWA1

#### Read the following carefully:

In this assignment, you will peer review two of your peers’ papers. The purpose of this assignment is to not only allow you to offer/receive valuable feedback, but to also gather ideas for how to improve your own writing. Use the prompts to evaluate the papers, but also make note that the questions asked will guide how your revised FWA1 will be graded.

To receive full credit for this assignment, you must offer insightful and valuable feedback to each question. Peer reviews are due by 11:59 pm CST on **the evening of your class**. After this, you will be able to access the reviews of your paper – you should end up with two reviews of your paper.

**Note: You may need to disable pop-up blockers to access reviews. If you find that Turnitin or PeerMark are behaving oddly, close out all browsers and start again.**

Directions:

1. Click on the FWA1 Turnitin submission link on Moodle.
2. Locate the PeerMark assignment, right-click on the green paper symbol.

3. (Agree to the Turnitin terms if necessary.)
4. If Turnitin asks you to sign-in or you have trouble with the application, close out your browser and begin again.
5. Under “Review’, click ‘Start Peer Review’.
6. To answer a free response question, click in the text box below the question and type in your response. The word minimum for the response is listed below the text box.
7. To highlight and add comments, click TOOLS in the top left-hand corner of the screen.
   a. Select the highlighter option (second from top) and choose a color.
   b. Click and drag your curser to indicate the portion you are highlighting.
   c. Add a comment to indicate what you’re referring to and click SAVE.
   d. Comments should be constructive, respectful, and informative.
8. If you need to save your work for later review, click the SAVE icon.
9. After you are finished and the top status bar is at 100%, click SUBMIT icon.
   a. If you answered all the questions and are still not at 100%, make sure you met the word minimum for all the short answer questions.
   b. A red asterisk will be next to any prompts that do not satisfy the minimum word requirement.
10. Repeat steps for your second paper (complete two reviews total).

#### Accessing your reviews

Once the due date for Peer Reviews has passed, you can access your reviews by returning to the PeerMark Assignment. Open your first review and use the arrows in the top right to navigate through your reviews. See screenshot below.

#### Grading:

Your grade for this assignment will be based on the following:

- Valuable and respectful feedback offered for each prompt in two papers.
- Highlights and comments are correct and valuable.
- Submitted on time.

After submitting, your grade may automatically display as 100%. This grade is subject to change, based on the quality of your feedback as determined by your instructor. If you receive poor feedback, contact your instructor.

# **PeerMark Questions – FWA1**

#### *Title*

1. From the title alone, list the dependent & independent variables, as well as the model organism.
2. A title should be descriptive of the study, yet concise. How can this student improve their title?

#### *Introduction*

3. Outline the intro topics in the order they are mentioned. Note: each paragraph should be a different topic.
4. Did the topics move from broad to narrow?
5. Is every introduction paragraph distinct in that each describes a different topic? Give one example of a paragraph that includes more than one topic.
6. Topic sentences are used to summarize ideas and should be found at the beginning of each paragraph. Highlight and comment on a strong topic sentence and which paragraph it is found. Make note if the topic sentence is not at the beginning.
7. Highlight and comment on one paragraph that has a weak or missing topic sentence. How can the sentence be improved?
8. List two transition words that were used in the introduction. Transition words can be used between sentences or at the beginning of a paragraph.
9. What research questions did the student aim to answer? You can shorthand them here.
10. Are the in-text citations from credible or non-credible sources? How do you know? List one credible source used.
11. Highlight and comment on one hypothesis and associated prediction that is unclear or not concise.
12. Choose one prediction with reasoning. How does the reasoning relate back to the background information?

#### *Methods*

13. List two examples of passive voice used in the methods section.
14. Highlight two examples of the student not using passive voice (if applicable).
15. List the subsections and subheadings used to organize the method section.
16. Do the subsections directly relate to the hypotheses stated in the introduction?
17. Are the paragraphs separated so that each describes a different topic?
18. Highlight and comment on one paragraph with unnecessary information.
19. Results should not be presented in the methods section. Give one example of where results were included

#### *References*

20. Are the references properly formatted: hanging indent & alphabetical? Give one example of a properly cited reference.
21. Do the references appear to be in APA format?
22. Glance through the references – do you see incorrect inclusion or exclusion of pertinent information? Give author last name if so.

#### *General Writing*

23. Provide overall feedback that includes at least one positive comment and one respectful critique.
